# An atlas of substrate specificities for the human serine/threonine kinome

**DOI:** 10.1038/s41586-022-05575-3

**Published:** 2023-01-11

**Authors:** Jared L. Johnson, Tomer M. Yaron, Emily M. Huntsman, Alexander Kerelsky, Junho Song, Amit Regev, Ting-Yu Lin, Katarina Liberatore, Daniel M. Cizin, Benjamin M. Cohen, Neil Vasan, Yilun Ma, Konstantin Krismer, Jaylissa Torres Robles, Bert van de Kooij, Anne E. van Vlimmeren, Nicole Andrée-Busch, Norbert F. Käufer, Maxim V. Dorovkov, Alexey G. Ryazanov, Yuichiro Takagi, Edward R. Kastenhuber, Marcus D. Goncalves, Benjamin D. Hopkins, Olivier Elemento, Dylan J. Taatjes, Alexandre Maucuer, Akio Yamashita, Alexei Degterev, Mohamed Uduman, Jingyi Lu, Sean D. Landry, Bin Zhang, Ian Cossentino, Rune Linding, John Blenis, Peter V. Hornbeck, Benjamin E. Turk, Michael B. Yaffe, Lewis C. Cantley

**Affiliations:** 1 Meyer Cancer Center, Weill Cornell Medicine, New York, NY USA; 2 Department of Medicine, Weill Cornell Medicine, New York, NY USA; 3 Englander Institute for Precision Medicine, Institute for Computational Biomedicine, Weill Cornell Medicine, New York, NY USA; 4 Department of Physiology and Biophysics, Weill Cornell Medicine, New York, NY USA; 5 Tri-Institutional PhD Program in Computational Biology & Medicine, Weill Cornell Medicine, Memorial Sloan Kettering Cancer Center and The Rockefeller University, New York, NY USA; 6 Weill Cornell Graduate School of Medical Sciences, Cell and Developmental Biology Program, New York, NY USA; 7 Department of Medicine, Division of Hematology/Oncology, Columbia University Irving Medical Center, New York, NY USA; 8 Herbert Irving Comprehensive Cancer Center, Columbia University Irving Medical Center, New York, NY USA; 9 Computer Science and Artificial Intelligence Laboratory, Massachusetts Institute of Technology, Cambridge, MA USA; 10 Center for Precision Cancer Medicine, Koch Institute for Integrative Cancer Biology, Departments of Biology and Biological Engineering, Massachusetts Institute of Technology, Cambridge, MA USA; 11 Department of Pharmacology, Yale School of Medicine, New Haven, CT USA; 12 Department of Chemistry, Yale University, New Haven, CT USA; 13 Institute of Genetics, Technische Universität Braunschweig, Braunschweig, Germany; 14 Department of Pharmacology, Rutgers Robert Wood Johnson Medical School, Piscataway, NJ USA; 15 Department of Biochemistry and Molecular Biology, Indiana University School of Medicine, Indianapolis, IN USA; 16 Division of Endocrinology, Weill Cornell Medicine, New York, NY USA; 17 Department of Genetics and Genomic Sciences, Icahn School of Medicine at Mount Sinai, New York, NY USA; 18 Department of Biochemistry, University of Colorado, Boulder, CO USA; 19 SABNP, Univ Evry, INSERM U1204, Université Paris-Saclay, Evry, France; 20 Department of Investigative Medicine, Graduate School of Medicine, University of the Ryukyus, Nishihara-cho, Japan; 21 Department of Developmental, Molecular and Chemical Biology, Tufts University School of Medicine, Boston, MA USA; 22 Department Of Bioinformatics, Cell Signaling Technology, Danvers, MA USA; 23 Rewire Tx, Humboldt-Universität zu Berlin, Berlin, Germany; 24 Department of Pharmacology, Weill Cornell Medicine, New York, NY USA; 25 Department of Biochemistry, Weill Cornell Medicine, New York, NY USA; 26 Divisions of Acute Care Surgery, Trauma, and Surgical Critical Care, and Surgical Oncology, Department of Surgery, Beth Israel Deaconess Medical Center, Harvard Medical School, Boston, MA USA; 27 Surgical Oncology Program, National Cancer Institute, National Institutes of Health, Bethesda, MD USA

**Keywords:** Kinases, Cellular signalling networks, Bioinformatics, Phosphorylation

## Abstract

Protein phosphorylation is one of the most widespread post-translational modifications in biology^1,2^. With advances in mass-spectrometry-based phosphoproteomics, 90,000 sites of serine and threonine phosphorylation have so far been identified, and several thousand have been associated with human diseases and biological processes^3,4^. For the vast majority of phosphorylation events, it is not yet known which of the more than 300 protein serine/threonine (Ser/Thr) kinases encoded in the human genome are responsible^3^. Here we used synthetic peptide libraries to profile the substrate sequence specificity of 303 Ser/Thr kinases, comprising more than 84% of those predicted to be active in humans. Viewed in its entirety, the substrate specificity of the kinome was substantially more diverse than expected and was driven extensively by negative selectivity. We used our kinome-wide dataset to computationally annotate and identify the kinases capable of phosphorylating every reported phosphorylation site in the human Ser/Thr phosphoproteome. For the small minority of phosphosites for which the putative protein kinases involved have been previously reported, our predictions were in excellent agreement. When this approach was applied to examine the signalling response of tissues and cell lines to hormones, growth factors, targeted inhibitors and environmental or genetic perturbations, it revealed unexpected insights into pathway complexity and compensation. Overall, these studies reveal the intrinsic substrate specificity of the human Ser/Thr kinome, illuminate cellular signalling responses and provide a resource to link phosphorylation events to biological pathways.

## Abstract (web summary)

Analysis of the kinase activity of 300 protein Ser/Thr kinases reveals that the substrate specificity of the kinome is substantially more diverse than expected and is driven extensively by negative selectivity

## Main

Phosphorylation of proteins at serine, threonine, tyrosine and histidine residues controls nearly every aspect of eukaryotic cellular function^1,2,5,6^. Misregulation of protein kinase signalling commonly results in human disease^7^. Deciphering the cellular roles of any protein kinase requires the elucidation of its downstream effector substrates. However, the majority of kinase–substrate relationships that have been published to date involve a relatively small number of well-studied protein kinases, while few, if any, substrates have been identified for the majority of the approximately 300 human protein Ser/Thr kinases within the human kinome^8–10^. This lack of knowledge of kinase–substrate relationships limits the interpretation of large mass spectrometry (MS)-based phosphoproteomic datasets, which to date have collectively reported over 200,000 Ser and Thr phosphorylation sites on human proteins^3,4,11–13^. The specific kinases that are responsible for these phosphorylation events have been reported for less than 4% of these sites^3^, substantially limiting the understanding of cellular phosphorylation networks.

Well-studied Ser/Thr kinases are generally known to recognize specific amino acid residues at multiple positions surrounding the site of phosphorylation^14–17^. This short linear motif, which is characteristic of a given protein kinase, ensures fidelity in signalling pathways regulating phosphorylation at a given Ser or Thr residue. Knowledge of kinase-recognition motifs can facilitate the discovery of new substrates, for example, by scanning phosphoproteomics data for matching sequences. However, to date, phosphorylation-site sequence motifs are known for only a subset of the human protein Ser/Thr kinome. We have previously described combinatorial peptide library screening methods that enable the rapid determination of specificity for individual kinases based on phosphorylation of peptide substrates^18,19^. Here we apply those methods to experimentally determine the optimal substrate specificity for the large majority of the human Ser/Thr kinome, characterize the relationship between kinases on the basis of their motifs, and computationally use these data to identify the protein kinases that are likely to phosphorylate any site identified using MS or other techniques. Finally, we show how this information can be applied to capture complex changes in signalling pathways in cells and tissues after genetic, pharmacological, metabolic and environmental perturbations.

## Profiling kinase substrate specificity

**Fig. 1:**
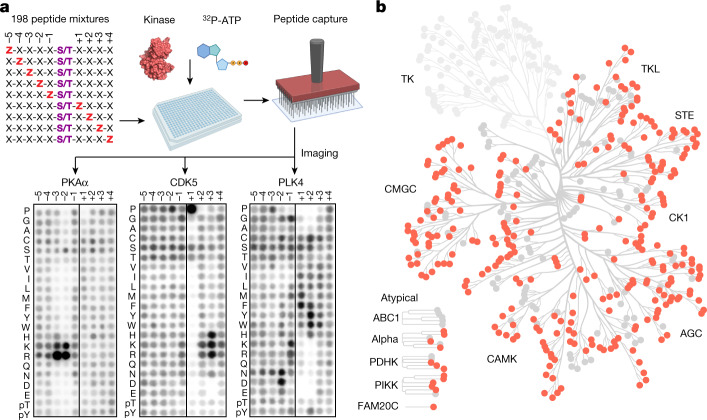
Profiling the substrate specificity of the human serine/threonine kinome. **a**, Experimental workflow for the PSPA analysis and representative results. The schematic was created using BioRender. Z denotes fixed positions containing one of the 20 natural amino acids, or either phosphorylated Thr (pThr) or phosphorylated Tyr (pTyr). X denotes unfixed positions containing randomized mixtures of all natural amino acids except Ser, Thr and Cys. Darker spots indicate preferred residues. **b**, Dendrogram of the human protein kinome, highlighting the Ser/Thr kinases analysed in this study.

Substrate-recognition motifs across the human Ser/Thr kinome were determined by performing a positional scanning peptide array (PSPA) analysis. We used a previously reported combinatorial peptide library that systematically substitutes each of 22 amino acids (20 natural amino acids plus phosphorylated Thr and phosphorylated Tyr) at nine positions surrounding a central phospho-acceptor position containing equivalent amounts of Ser and Thr^19^ (Fig. 1a). Using purified recombinant kinase preparations, we successfully obtained phosphorylation-site motifs for 303 Ser/Thr kinases, covering every branch of the human Ser/Thr kinase family tree as well as a collection of atypical protein kinases (Fig. 1b, Supplementary Fig. 1 and Supplementary Tables 1 and 2). Profiling of the large majority of these kinases, including 83 understudied ‘dark’ kinases, was lacking^8^.

**Fig. 2:**
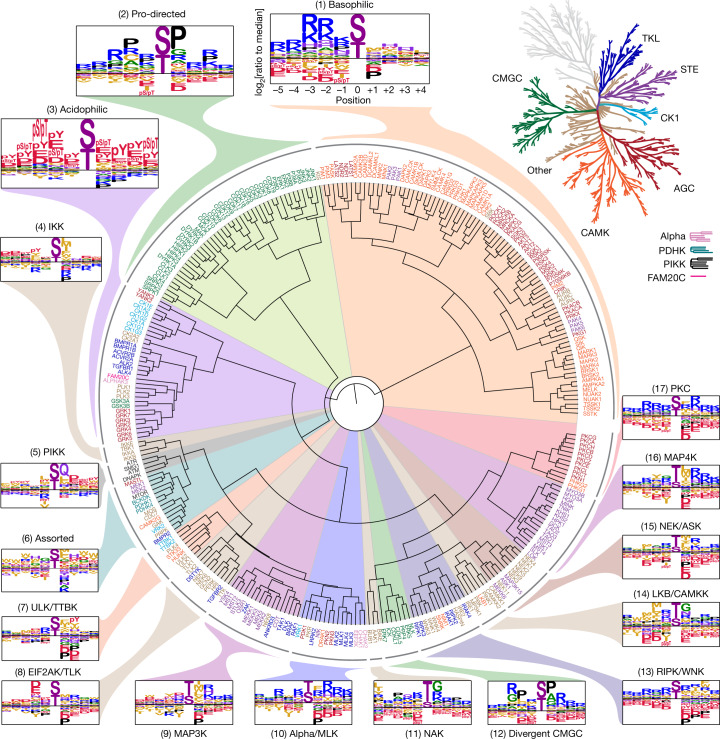
Phosphorylation-site motif tree of the human Ser/Thr kinome. Hierarchical clustering of 303 Ser/Thr kinases on the basis of their amino acid motif selectivity  (PSSMs). Kinase names are colour labelled according to their phylogenetic relationships (top right)^2^.

Position-specific scoring matrices (PSSMs) derived from quantified PSPA data were analysed by hierarchical clustering to compare kinase substrate motifs across the kinome (Fig. 2 and Supplementary Table 2). As expected, kinases sharing substantial sequence identity displayed a high degree of similarity in their optimal substrate motifs. However, we found many cases in which clustering by PSSM did not strictly recapitulate the evolutionary phylogenetic relationships between kinases inferred from their primary sequences (Fig. 2). Instead, members of most of the major kinase groups were distributed throughout the dendrogram, reflecting numerous examples in which kinases with low overall sequence identity have converged to phosphorylate similar optimal sequence motifs. For example, we found that a number of distantly related kinases (in the YANK, CK1 and 2, GRK and TGFβ receptor families) converged to phosphorylate similar sequence motifs despite their disparate locations on the kinome tree (Fig. 2 (cluster 3)).

**Extended Data Fig. 1:**
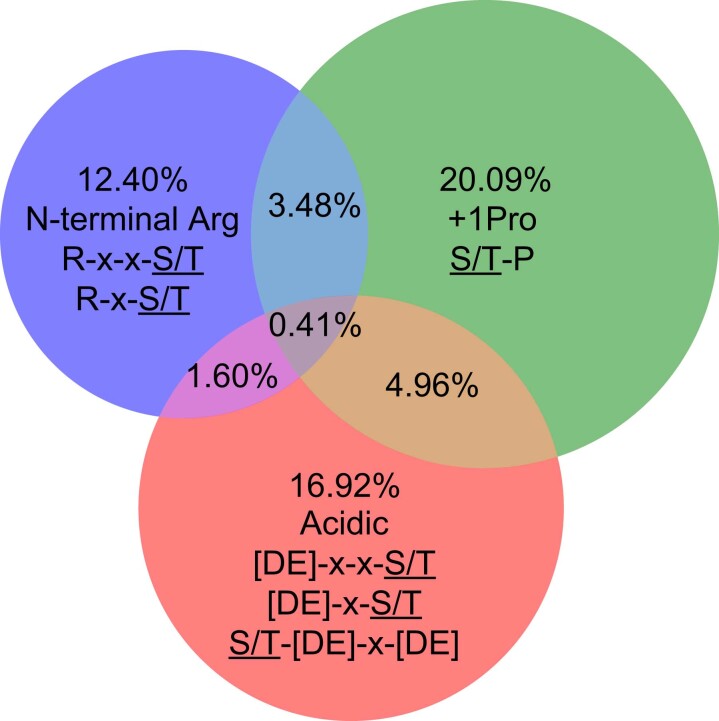
Representation of phosphorylation site motifs in the human serine and threonine phosphoproteome. Venn diagram representation of the percentages of three prominent Ser/Thr kinase motif features, pertaining to Clusters 1, 2, and 3 in Fig. 2, across 82,735 human serine and threonine phosphorylation sites confidently identified in mass spectrometry experiments^4^. The phosphorylated residues in the logos are represented as S/T.

**Extended Data Fig. 2:**
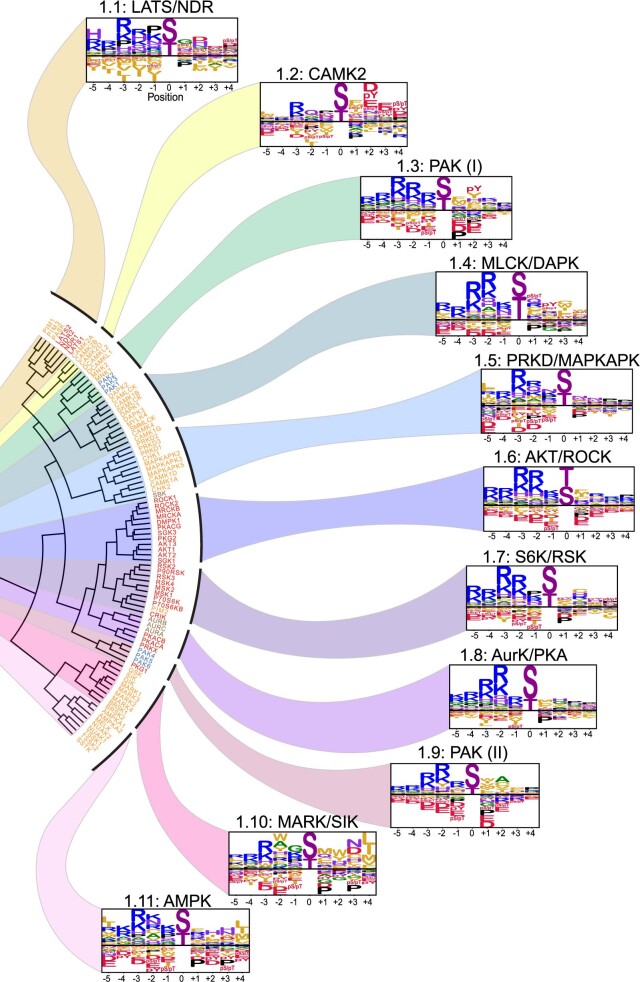
Subcategorization of the basophilic kinases of Cluster 1. Subcategorization of Cluster 1 from Fig. 2 into 11 motif classes.

**Extended Data Fig. 3:**
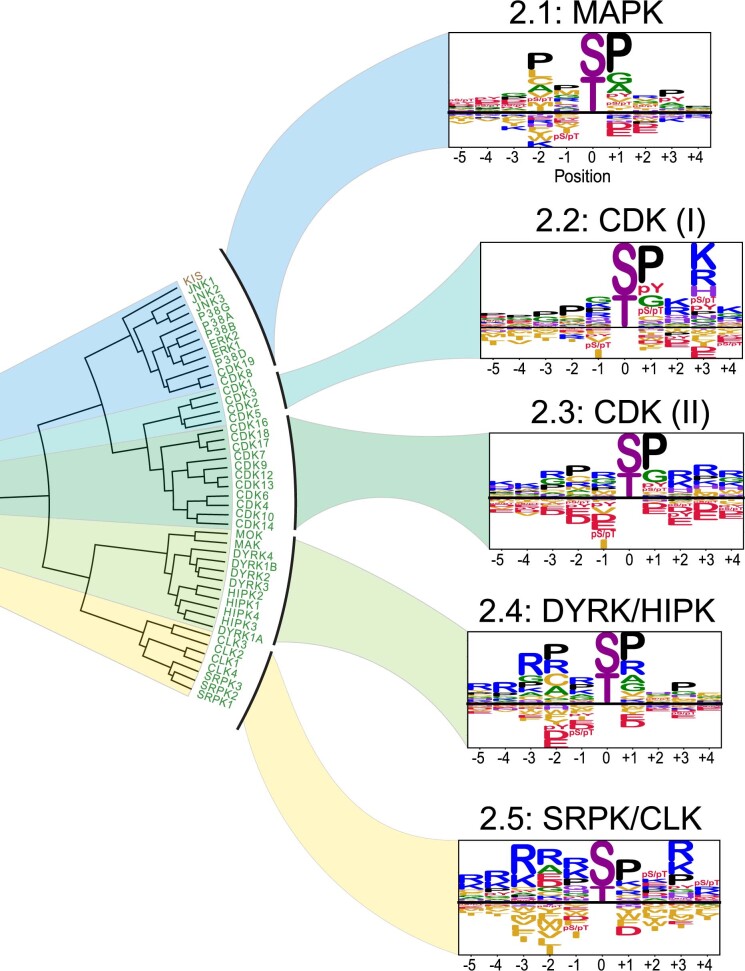
Subcategorization of the proline-directed kinases of Cluster 2. Subcategorization of Cluster 2 from Fig. 2 into 5 motif classes.

**Extended Data Fig. 4:**
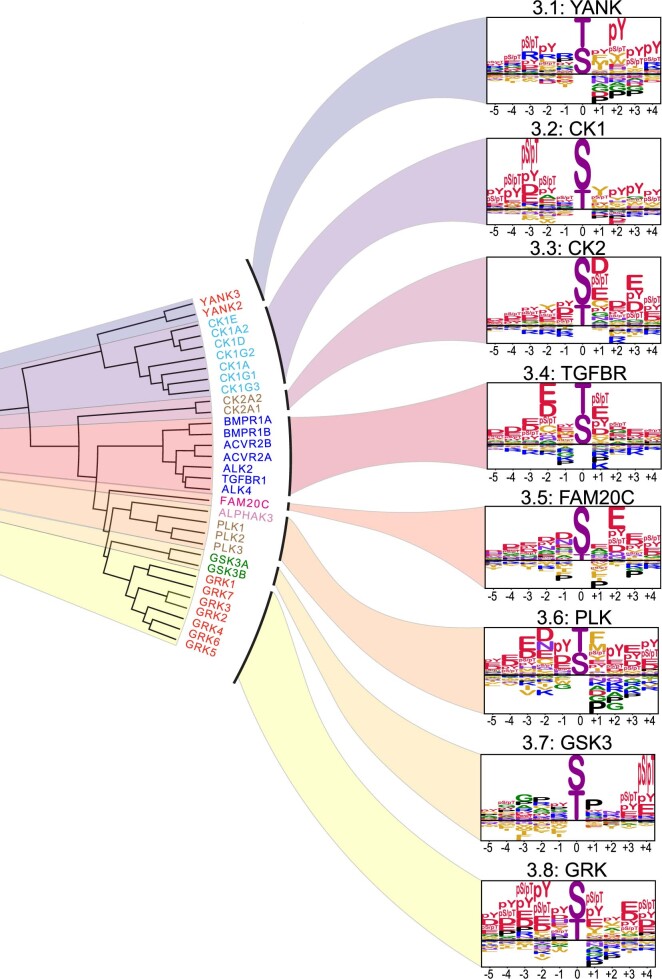
Subcategorization of the acidophilic kinases of Cluster 3. Subcategorization of Cluster 3 from Fig. 2 into 8 motif classes.

**Extended Data Fig. 5:**
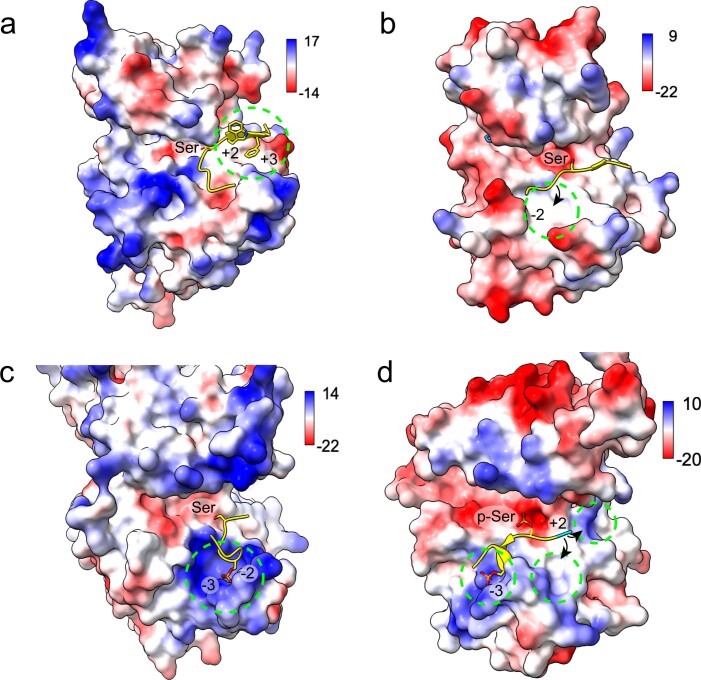
Structural models of kinase–substrate complexes. **a**, Synthetic peptide from its complex with PAK4 (PDB: 2Q0N) modelled onto WNK3 (PDB: 5O26). Dotted circle highlights a shallow hydrophobic pocket accommodating a +3 Phe residue. **b**, GSK3 peptide from its complex with AKT2 (PDB: 1O6L) modelled onto CAMKK2 (PDB: 2ZV2). Circle indicates a hydrophobic pocket that could accommodate a −2 aliphatic residue. **c**, Monophosphorylated peptide from p63 bound to CK1δ (PDB: 6RU6) modelled onto GRK2 (PDB: 1YM7). Circle shows positive surface potential in the vicinity of the −2 and −3 pSer residues. **d**, p63 peptide bound to CK1δ (PDB: 6RU8) was modelled onto YANK1 (PDB: 4FR4) showing potential binding sites for −3 and +2 phosphorylated residues. Surface electrostatics are represented with Coulombic potential values were computed in ChimeraX and represented by scale bars (kcal/mol·e).

Overall inspection of sequence motifs associated with various branches of the motif-based dendrogram revealed that approximately 60% of the Ser/Thr kinome could be represented by simple assignment to one of three previously observed motif classes: selectivity for basic residues N terminal to the phosphorylation site (cluster 1; Fig. 2); directed by a proline residue at the +1 position (cluster 2); or a general preference for negatively charged (acidic and phosphorylated) residues at multiple positions (cluster 3)^15,20,21^. Notably, more than half of all of the reported phosphorylation sites observed by MS could be assigned to one of these three signatures (Extended Data Fig. 1). However, each of these motif classes could be further subcategorized on the basis of selectivity both for and against distinct sets of residues at other positions, reflecting considerable diversity within these clusters (Extended Data Figs. 2–4). The remaining approximately 40% of the Ser/Thr kinome comprised many smaller groups that displayed unique sequence determinants (Fig. 2; clusters 4–17). For example, motifs for the PIKK family kinases (ATM, ATR, DNA-PK and SMG1) clustered into a group that primarily selected a Gln residue at the +1 position (cluster 5), consistent with previous studies^22,23^. Notably, several clusters displayed shared consensus motifs that have not been well recognized previously, such as the group including the IRAK, IRE, WNK, SNRK and RIP kinases (cluster 13), of which the substrate motifs contained basic residues both N and C terminal to the phosphorylation site with dominant selection for aromatic residues at the +3 position. As another example, the kinases LKB1, CAMKK, PINK1 and PBK (cluster 14) primarily recognized hydrophobic residues N terminal to the phosphorylation site in combination with selection for turn-promoting residues (Gly or Asn) at the +1 position. Structural modelling of kinase–peptide complexes revealed complementary features within the kinase catalytic clefts that are probably responsible for the recognition of these motifs (Extended Data Fig. 5a,b).

An important and less generally recognized feature that dominated the clustering was strong negative selection against either positively or negatively charged residues at distinct positions within a motif, suggesting that electrostatic filtering strongly influences kinase substrate selection throughout the kinome^24^. We identified additional classes of amino acids, such as hydrophobic residues, that are selected against by a variety of kinases. These trends suggest that substrate avoidance has a fundamental role in dictating correct kinase–substrate interactions^25,26^.

Unexpectedly, we observed that many kinases (129 out of 303) selected either a phosphorylated Thr or a phosphorylated Tyr as the preferred amino acid in at least one position within the motif (Supplementary Fig. 1; where selectivity for phosphorylated Ser was assumed to be equivalent to phosphorylated Thr). In addition to kinases of which the dependence on phospho-priming was previously known (GSK3, CK1 and CK2 families; cluster 3), this phenomenon was particularly evident for the GRK- and YANK-family kinases (Extended Data Fig. 4), both of which have complementary basic residues within their catalytic domains (Extended Data Fig. 5c,d). Notably, individual GRK-family members showed unique and specific selection for the location of the phosphorylated Thr or phosphorylated Tyr residue within their substrate peptides. GRKs are best known for their role in the desensitization of G-protein-coupled receptors (GPCRs), whereby multisite phosphorylation induces the binding of arrestin proteins to inhibit signalling^27,28^. Our findings suggest that the capacity for only seven GRKs to differentially regulate 800 distinct GPCRs probably involves a complex interplay between initial sequence-specific phospho-priming of GPCRs by other Ser/Thr and Tyr kinases, followed by a second level of specificity resulting from GRK-dependent phosphorylation and subsequent recognition by a small number of β-arrestins.

**Extended Data Fig. 6:**
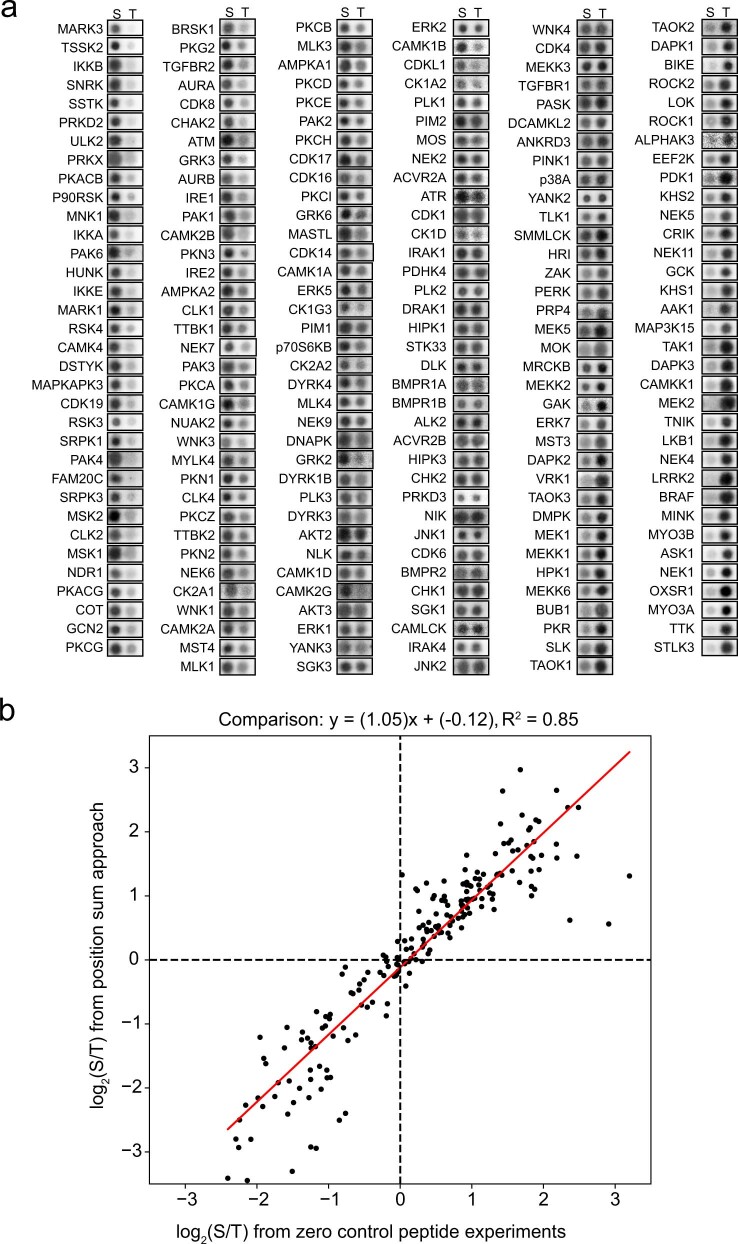
Profiling phospho-acceptor specificity. **a**, *in vitro* phosphorylation assays with recombinant kinases and substrate peptides containing either serine or threonine phospho-acceptors. Results shown for 208 recombinant Ser/Thr kinases. **b**, Correlation plot of the experimental results in (**a**) with the position sum approach applied in this study to score Ser/Thr phospho-acceptor preference.

**Extended Data Fig. 7:**
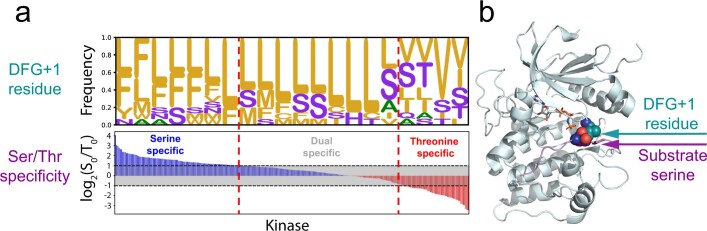
Global analysis of the relationship between the DFG+1 amino acid and preference for the serine versus threonine phospho-acceptor. **a**, Bottom, relative preferences for Ser or Thr phospho-acceptor residues for each kinase, arranged in order of decreasing Ser/Thr selectivity. Top, frequency of amino acids at the DGF+1 positions of corresponding kinases (bin size: 15 kinases). **b**, Structural illustration of the proximity between the DFG+1 residue and substrate phospho-acceptor residue, shown with the AKT1 kinase domain bound to substrate (GSK3β) peptide (pdb 1O6K).

Features of substrate-recognition motifs across the entire kinome could be structurally rationalized on the basis of the presence of specificity-determining residues at particular positions within the kinase catalytic domain^29–32^, leading to both expected and unexpected findings. For example, we found that half of the kinases display some degree of selectivity for either a Ser or a Thr as the phospho-acceptor residue (Extended Data Figs. 6 and 7). Consistent with our previously published observations^33^, Ser or Thr phospho-acceptor site preference strongly correlated with the identity of the ‘DFG+1’ residue (that is, the residue immediately after the canonical Asp-Phe-Gly motif within the kinase activation loop), with bulky residues (Phe, Trp, Tyr) at this position in Ser-selective protein kinases and β-branched residues (Val, Ile, Thr) at this position in Thr-selective kinases. However, for some DFG+1 residues, Ser versus Thr selectivity was unexpectedly context dependent. For example, a Leu residue at the DFG+1 position was observed in both Ser-selective and dual-specificity kinases, whereas a DFG+1 Ala residue resulted in a preference for Thr phosphorylation in the context of some kinases (for example, the mitogen-activated protein kinase kinase kinases (MAP3Ks)), but a preference for Ser specificity in others (the IκB kinases). These observations, notable only within the context of the complete Ser/Thr kinome, indicate that additional residues beyond the previously established DFG+1 position can influence Ser/Thr specificity in a context-dependent manner.

## Annotation of the human phosphoproteome

**Fig. 3:**
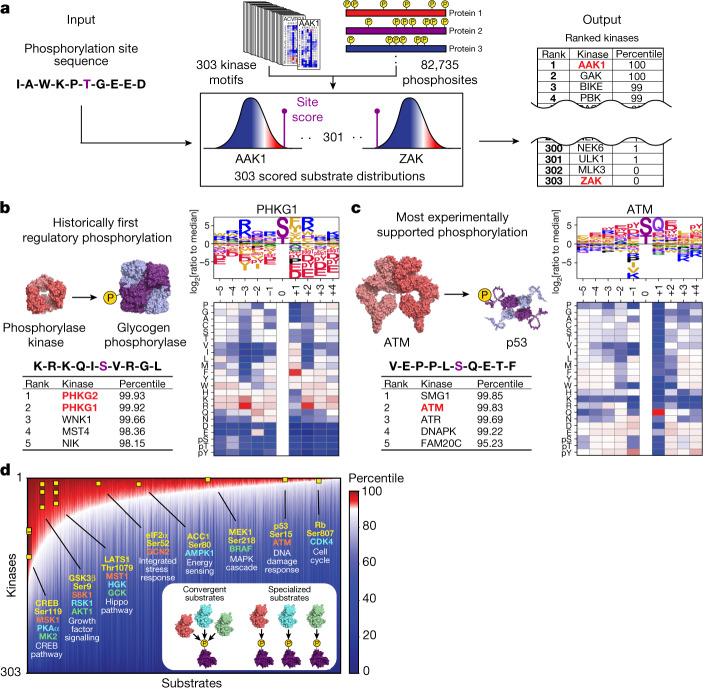
Phosphorylation motifs for the human Ser/Thr kinome enable comprehensive scoring and annotation of the human phosphoproteome. **a**, Schematic of the substrate-scoring process^4^. **b**, Results for Ser15 on glycogen phosphorylase alongside PSSM and the substrate motif logo of its established kinase glycogen phosphorylase kinase. **c**, The results for Ser15 of p53 alongside its established kinase ATM. **d**, Annotation of the human Ser and Thr phosphoproteome by percentile scores from 303 Ser/Thr kinases performed as shown in **a**. A total of 89,752 phosphorylation sites that were identified using high-throughput approaches^4^ and/or low-throughput approaches^3^ were sorted along the *x*-axis by their numbers of kinases with percentile scores higher than 90. On the *y*-axis, kinase percentile scores were sorted by rank separately for each site and represented in the heat map. Examples of well-studied kinase–substrate relationships are highlighted (yellow squares). Inset: phosphorylation sites on the left end of the plot scored favourably for many kinases, whereas sites on the right end scored favourably for fewer kinases.

**Extended Data Fig. 8:**
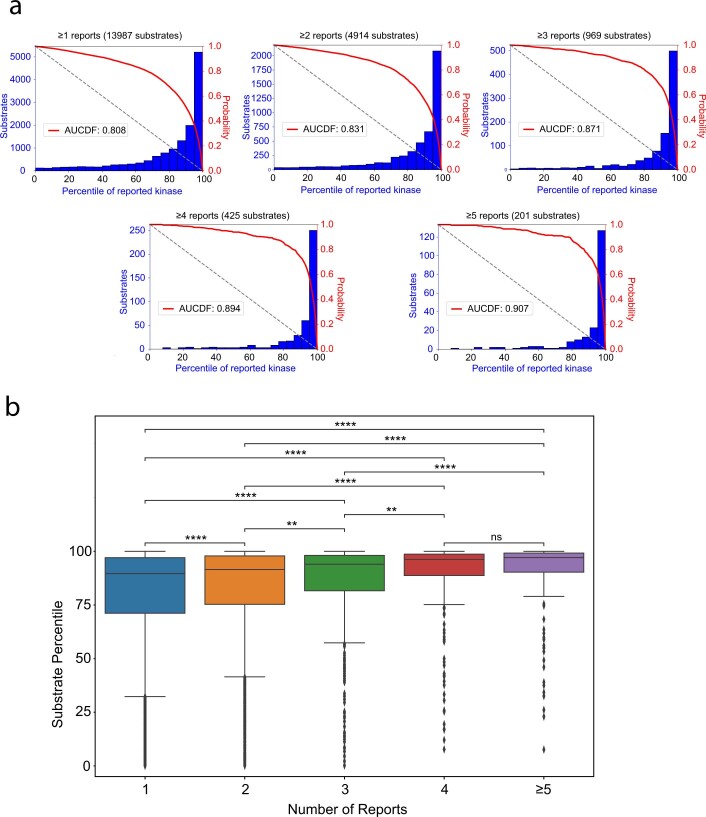
Global performance analysis of substrate percentile scores for their literature-annotated kinases. **a**, Percentile-score distributions of substrates for their literature-annotated kinases (AUCDF= area under the cumulative distribution function). **b**, Percentile-score of literature-annotated kinase–substrate pairs as a function of number of reports. Higher number of reports correlates with more favourable percentile-scores between the reported kinase and its substrate. n = 9,073, n = 3,945, n = 544, n = 224, and n = 201 for kinase–substrate relationships with 1, 2, 3, 4, and 5 or more reports, respectively. Statistical analyses were performed using Double-sided Mann-Whitney U-test. Box minima=25th percentile, centre=50th percentile, maxima=75th percentile. Whiskers extend from the box maxima or minima to the largest or smallest value no further than 1.5 x interquartile range. (ns p > 0.05, * p ≤ 0.05, ** p ≤ 10^−3^, *** p  ≤ 10^−4^, **** p ≤ 10^−5^).

**Extended Data Fig. 9:**
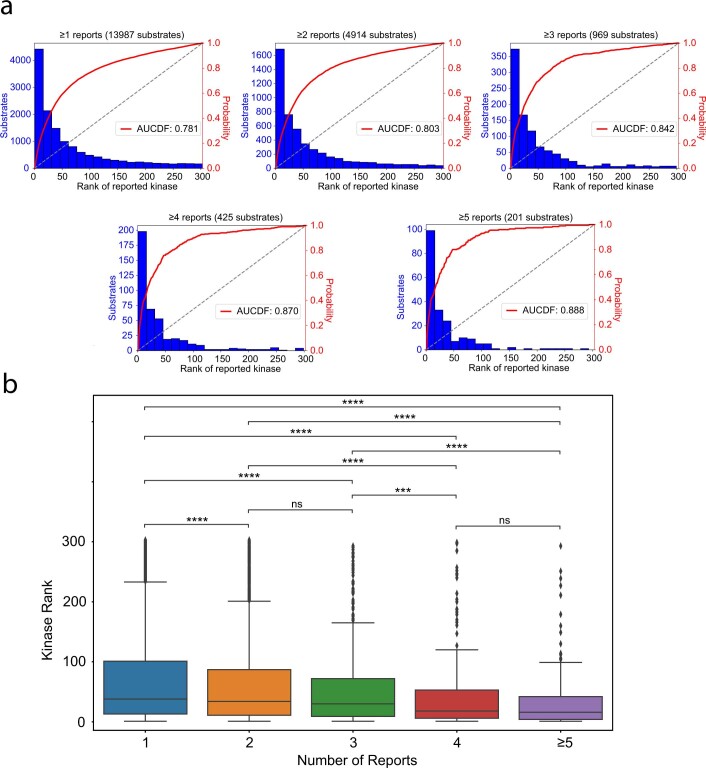
Global performance analysis of kinase ranks for their literature-annotated substrates. **a**, Rank distributions of kinases for their literature-annotated substrates (AUCDF= area under the cumulative distribution function). **b**, Rank of the literature-annotated kinase–substrate pairs, as a function of number of reports. Higher number of reports correlates with more favourable ranking of reported kinase for its substrate. n = 9,073, n = 3,945, n = 544, n = 224, and n = 201 for kinase–substrate relationships with 1, 2, 3, 4, and 5 or more reports, respectively. Statistical analyses were performed using Double-sided Mann-Whitney U-test. Box minima=25th percentile, centre=50th percentile, maxima=75th percentile. Whiskers extend from the box maxima or minima to the largest or smallest value no further than 1.5 x interquartile range. (ns p > 0.05, * p ≤ 0.05, ** p ≤ 10^−3^, *** p ≤ 10^−4^, **** p ≤ 10^−5^).

Comprehensive knowledge of human Ser/Thr kinase specificity has the potential to ‘deorphanize’ the large number of reported phosphorylation sites with no associated kinase. To do so, we generated a kinome-wide annotation of the human Ser/Thr phosphoproteome, comprising a previously curated set of 82,735 sites that were detected using high-throughput MS^4^ plus an additional 7,017 sites that were identified using only low-throughput methods^3^. Using approaches adapted from previously published research, we computationally ranked these 89,752 sites against each Ser/Thr kinase motif^34,35^ (Fig. 3a and Supplementary Table 3). Notably, approximately 99% of these phosphorylation sites ranked favourably for at least one kinase that we profiled (that is, the site scored in the top 10% of all sites in the human phosphoproteome for that kinase). These annotations were strongly concordant with sites for which protein kinases involved have been previously identified. For phosphorylation sites of which the upstream kinase has been previously verified by at least three independent reports, encompassing 969 sites and over one third of the kinome, our motif-based approach yielded a median percentile of 95% (that is, the reported site received a higher score than 95% of all putative phosphorylation sites in the phosphoproteome for its established kinase) (Extended Data Fig. 8a). Furthermore, when we back-mapped the motifs of all 303 profiled kinases onto the literature-reported phosphorylation sites, our approach yielded a median reported kinase percentile of 92% (that is, the reported kinase scored more favourably than 92% of all profiled kinases in our atlas for its established substrate) (Extended Data Fig. 9a). These rankings further improved when we considered kinase–substrate pairs with higher numbers of previous reports (Extended Data Figs. 8 and 9), suggesting that, in a large majority of cases, the linear sequence context of phosphorylation sites contributes substantially to kinase–substrate relationships.

Notably, motif predictions alone successfully identified numerous prominently studied kinase–substrate relationships. For example, phosphorylase kinases PHKG1 and PHKG2 emerged as the top two hits (out of 303 kinases) for phosphorylating Ser15 of glycogen phosphorylase (Fig. 3b). This phosphoregulatory event, the very first to be discovered^36^, opened up the entire field of phosphorylation-dependent signal transduction. The most highly cited kinase–substrate interaction reported to date is the phosphorylation of the tumour suppressor p53 at Ser15 by the DNA-damage-activated kinase ATM, which scored among the top-ranking kinases associated with that site (Fig. 3c). Notably, other kinases reported to phosphorylate the same site—ATR, SMG1 and DNAPK—scored within the top four predicted kinases^3^.

**Extended Data Fig. 10:**
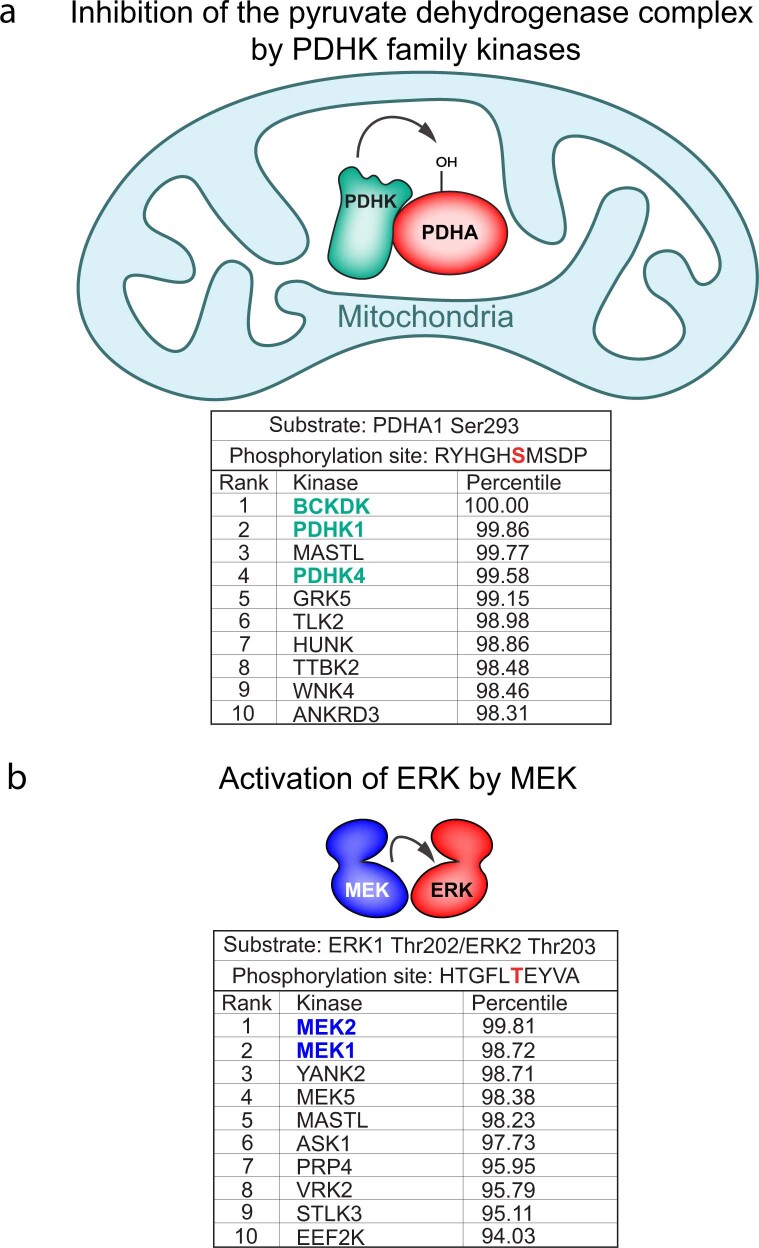
Motif-based scoring results for phosphorylation events facilitated by subcellular localization or docking. **a**, Illustration of the mitochondrial-localized regulation of the pyruvate dehydrogenase complex through phosphorylation by the PDHKs. Scoring results for PDHA1 Ser293, highlighting the PDHK family kinases. **b**, Illustration of docking-driven phosphorylation of ERK1/2 by MEK1/2. Scoring results for ERK1 Thr202 / ERK2 Thr203 (identical sequences), highlighting MEK1 and MEK2.

**Extended Data Fig. 11:**
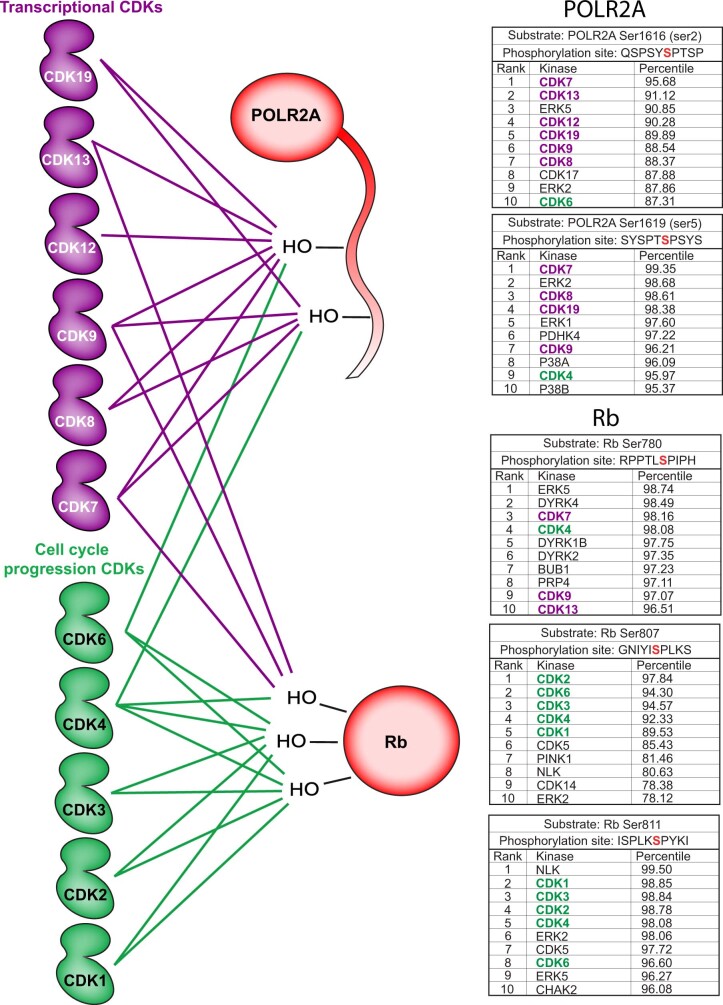
Scoring comparison of CDK subfamilies. Illustration of the phosphoregulation of RNA Polymerase II (POLR2A) CTD and Retinoblastoma protein (Rb) by their respective canonical CDKs, the transcriptional CDKs (purple) and the cell cycle progression CDKs (green) (left). Links between kinases and substrates correspond to favourable scores between motifs and phosphorylation sites (right).

Our approach could also correctly identify kinases for phosphorylation events driven by substrate co-localization or non-catalytic docking interactions, for which we expected less dependence on the phosphorylation-site motifs of their kinases. For example, we correctly identified both the mitochondrial-localized phosphorylation of pyruvate dehydrogenase by the pyruvate dehydrogenase kinases (Extended Data Fig. 10a) and the docking-driven phosphorylation of the MAP kinase ERK by MEK^37^ (Extended Data Fig. 10b). Notably, the phosphorylation site on ERK was selected against by nearly every human protein kinase that we profiled except for MEK, explaining how ERK can be exclusively regulated by MEK while avoiding phosphorylation by the kinome at large. Finally, our approach could tease apart kinase subfamilies with similar motifs and correctly assign them to their established substrates. For example, we could distinguish between the CDK family kinases that assume classical roles in cell cycle progression (that is, CDK1, CDK2, CDK3, CDK4 and CDK6) and the subset of CDKs that govern gene transcription (that is, CDK7, CDK8, CDK9, CDK12, CDK13 and CDK19)^38,39^ (Extended Data Fig. 11).

Functional annotation of the human phosphoproteome enabled us to examine global trends in kinase–substrate interactions. We found that most phosphorylation sites could be assigned to a small number of putative kinases (that is, BRAF–MEK1, ATM–p53 and CDK4–Rb; Fig. 3d and Supplementary Table 3). However, approximately one-third of all sites lacked unique negative sequence-discriminating features and, instead, matched well to the optimal phosphorylation motifs for a greater number of kinases^21,40,41,42^ (that is, Ser119 of CREB, Ser9 of GSK3B and Thr1079 of LATS1; Fig. 3d). This could suggest the importance of other kinase-determining factors (scaffolds, localization and so on) for proper kinase substrate recognition, or may indicate that these specific phosphorylation sites are points of convergence for multiple signalling pathways. For example, cAMP response element binding protein (CREB) is canonically phosphorylated at Ser119 by cAMP-dependent protein kinase (PKA); however, numerous previous reports demonstrate that a broad range of cellular stimuli and drug perturbations impinge on the phosphorylation of this site by no less than ten distinct kinases^3^. Taken together, these findings suggest that the presence of negative-selectivity elements flanking a putative phosphorylation site can be used to insulate a substrate from inappropriate phosphorylation by dozens of related kinases, whereas the absence of such negative selectivity can enable protein kinases in distinct pathways to converge on the same target.

## Motif-enrichment analysis

**Fig. 4:**
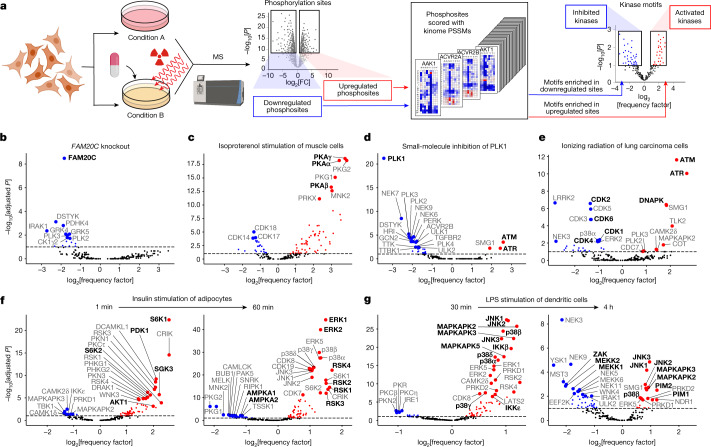
Global motif analysis reveals how kinase perturbations and pathway rewiring reshape the phosphoproteome. **a**, Workflow of the motif enrichment analysis of phosphoproteomics data. The schematic was created using BioRender. **b**–**g**, Results from published datasets. **b**, Conditioned medium of HepG2 cells after genetic deletion of *FAM20C*^44^. **c**, Cultured myotubes after 30 min treatment with 2 μM isoproterenol^45^. **d**, HeLa cells after mitotic arrest by treatment for 45 min with 0.1 μM PLK1 inhibitor BI 2536 (ref. ^46^). **e**, A549 cells 2 h after exposure to 6 Gy of ionizing radiation^49^. **f**, 3T3-L1 adipocytes after serum starvation and then 1 min and 60 min treatment with 100 nM insulin^54^. **g**, C57BL/6J mouse bone-marrow-derived dendritic cells after 30 min and 4 h treatment with 100 ng ml^−1^ lipopolysaccharide (LPS)^55^. The enrichments in **b**–**g** were determined using one-sided exact Fisher’s tests and corrected for multiple hypotheses using the Benjamini–Hochberg method. Fully annotated versions of these plots are presented in Supplementary Fig. 2.

Cell signalling networks are complex and dynamic. Perturbation of kinase signalling pathways by genetic manipulations, treatment with growth factors and ligands, environmental stress or small-molecule inhibitors reshapes the phosphoproteome through both direct and indirect effects as a consequence of secondary signalling responses and/or off-target effects from the experimental treatment^43^. Owing to the interconnected and dynamic nature of phosphorylation networks, distinguishing between initial signalling events and those that result from the subsequent activation of additional signalling pathways is a common and challenging problem. We reasoned that kinases underlying both primary and secondary phosphorylation events could potentially be revealed by a global motif-based analysis of changes in the corresponding phosphoproteome. To test this idea, we used publicly available MS datasets from cells collected in the absence or presence of various perturbations and scored all phosphorylation sites with our atlas of Ser/Thr kinase motifs. Kinase motifs that were significantly enriched or depleted after experimental treatment were then represented as volcano plots of motif frequencies and adjusted *P* values (Fig. 4a).

Using this approach, we found that sequence motifs corresponding to the most direct target of a genetic or chemical perturbation were among the most significantly regulated, as seen, for example, for the genetic deletion of the secreted primordial casein kinase FAM20C (Fig. 4b). When quantitative phosphoproteomics data from HepG2 cells lacking FAM20C^44^ were analysed using our kinome-wide dataset, the most downregulated kinase-recognition motif corresponded to that of FAM20C. Similarly, when skeletal-muscle-like myotube cells were stimulated for 30 min with isoproterenol^45^, the most upregulated phosphorylation motifs corresponded to multiple isoforms of cAMP-dependent protein kinase (PKA)—canonical effector kinases downstream of the β_1_ and β_2_ adrenergic receptors (Fig. 4c). Notably, PKA motifs are highly similar to those of several other basophilic kinases, yet we could identify their enrichment in this scenario. Moreover, our comprehensive Ser/Thr kinome motif collection elucidated secondary signalling events in a dataset from HeLa cells arrested in mitosis using the PLK1 inhibitor BI 2536 (Fig. 4d)^46^; here, in addition to observing a notable downregulation of substrates containing the optimal PLK1 motif, we also noted upregulation of substrates phosphorylated by ATM and ATR. This finding is in good agreement with previous reports that PLK1 can suppress DNA damage signalling in mitotic cells^47,48^.

Our motif-based analysis could also be used to reveal key signalling events resulting from more complex interventions. For example, we examined phosphoproteomic data from A549 cells treated with 6 Gy of ionizing radiation^49^ (Fig. 4e). Our analysis revealed the up- and downregulation of numerous signalling pathways, including upregulation of canonical kinases involved in the DNA-damage response (ATM, ATR, DNA-PK) and downregulation of canonical kinases involved in cell cycle progression (CDK1, CDK2, CDK4 and CDK6), consistent with G1/S and G2/M arrest. Furthermore, we found up- and downregulation of less-appreciated DNA-damage-responsive kinases (MAPKAPK2^50,51^, PLK3^52^ and LRRK2^53^).

The full collection of Ser/Thr kinome motifs also enabled the temporal dynamics of signalling to be resolved from time-resolved phosphoproteomic datasets. For example, motif-based analysis of phosphoproteomic data from insulin-treated 3T3-L1 adipocytes^54^ revealed rapid activation of the phosphoinositide 3-kinase signalling pathway within 1 min after insulin stimulation followed by subsequent activation of the MAPK pathway, together with downregulation of AMP-activated protein kinases within 60 min (Fig. 4f). Similarly, phosphoproteomic data analysis from lipopolysaccharide-stimulated dendritic cells^55^ suggested marked upregulation at 30 min of a set of stress-activated kinases including the IKKs, JNK and p38 MAPKs, along with the MAPKAPK family of p38 effector kinases. This was followed within 4 h by the subsequent upregulation of the PIM kinases and suppression of the MAPKs in parallel with the downregulation of their upstream MAPK3Ks (MEKK1, MEKK2 and ZAK)^56^, suggestive of a negative-feedback loop (Fig. 4g). Thus, comprehensive motif-based approaches, when applied to time-resolved phosphoproteomics experiments, can decipher the distinct temporal dynamics of different groups of kinases.

## Discussion

Here we present the full spectrum of substrate motifs of the human serine/threonine kinome and provide an unbiased comprehensive framework to further explore their cellular functions. Globally, these motifs are substantially more diverse than expected, suggesting a broader substrate repertoire of the kinome. Hierarchical clustering of this dataset reorganized the kinome into at least 38 motif classes and introduced several shared motif features (Fig. 2 and Extended Data Figs. 2–4).

The Ser/Thr kinases that we profiled were, almost without exception, strongly discriminatory against specific motif features. These findings suggest that fidelity in kinase signalling pathways is largely achieved through selective pressure on substrates to avoid phosphorylation by the majority of irrelevant kinases, and that this may occur by tuning the amino acid sequences surrounding the phosphorylation sites to be disfavoured by non-cognate kinases. As this negative selection contributes substantially to proper substrate recognition, accurate identification of kinase–substrate relationships requires a comprehensive knowledge of kinase phosphorylation motifs—not only for an individual kinase of interest, but also for all other kinases in the human kinome that might compete for the same substrate pool.

When this kinome-wide dataset was used to predict the specific kinases that are responsible for substrate phosphorylation solely based on the amino acid sequence surrounding the phosphorylation site, the results were highly accurate at identifying correct kinase–substrate relationships, even without knowledge of tissue specificity, scaffolding effects or subcellular localization. Including such additional information will probably further improve these predictive approaches^57,58^. A limitation of using first-order peptide arrays in these experiments is that they do not directly measure the contributions of interpositional contacts within the substrate peptides, which we have previously shown can affect substrate selection for some tyrosine kinases^32^, albeit less so for Ser/Thr kinases^59^. Moreover, we were unable to differentiate between positional selection of Ser or Thr residues and direct phosphorylation of neighbouring residues (for example, peptides containing more than one phospho-acceptor). Structural modelling approaches guided by kinase substrate motif data will potentially decipher this additional information to further improve predictions^60,61^.

The examination of MS phosphoproteomic datasets using this global collection of motifs yielded potential biological insights and putative kinase substrates (Fig. 4). For example, in cells undergoing exposure to ionizing radiation (Fig. 4e), ATM was predicted to target 37 of the phosphorylation sites that were upregulated, most of which have never been associated as substrates for ATM (Supplementary Table 4). As the application of phosphoproteomics to human clinical samples and disease model systems continues to advance, our comprehensive motif-based approach will be uniquely equipped to unravel the complex signalling that underlies human disease progressions, mechanisms of cancer drug resistance, dietary interventions and other important physiological processes. In summary, we foresee that this will provide a valuable resource for a broad spectrum of researchers who study signalling pathways in human biology and disease.

## Methods

### Cell lines

Expi293 (Thermo Fisher Scientific) and HEK293T (ATCC) cells were obtained directly from vendors that perform short tandem repeat genotyping for authentication of human cells and were verified to be mycoplasma-free. Sf9 insect cells were obtained from Thermo Fisher Scientific.

### Plasmids

For expression and purification from bacteria, the DNA sequences for the human Ser/Thr kinases, binding partners and chaperones listed below were codon-optimized for expression in *Escherichia coli* using GeneSmart prediction software (GenScript). Optimized coding sequences were synthesized as gBlocks (Integrated DNA Technologies) carrying 16 bp overhangs at the 5′ and 3′ ends to facilitate in-fusion cloning (Clontech) into pET expression vectors (EMD Millipore).

pCDFDuet1 constructs were as follows: HSP90AA1-His_6_(full length), hereafter referred to as ‘HSP90’; untagged HSP90(full length); His_6_-MO25a(full length); His_6_-ALPHAK3/ALPK1 N-terminal domain (1–474); and His_8_-CCNC(full length) in tandem with MED12-His_8_(1–100); untagged MEK5/MAP2K5(S311D, T315D; full length); and untagged CK2B(full length). pET28a constructs were as follows: His_6_-PDPK1(full length); His_6_-PRP4/PRPF4B(519–end); GST-CAMK1A(full length); GST-CHAK2/TRPM6(1699–end); His_6_-caMLCK/MYLK3(490–end); His_6_-CAMKK1 (124–411); His_6_-ERK7/MAPK15(full length); His_6_-SUMO-ALPHAK3/ALPK1 CTD(959–end); MYO3A-His_6_(1–308); ERK5/MAPK7-His_6_(1–405); His_6_-NIK/MAP3K14(327–673); and BMPR2-His_6_(172–504). pETDuet1 constructs were as follows: His_6_-CDK8(1–360, fusion with C-tail of CDK19(360–end)), His_6_-CDK19(full length); His_6_-AAK1(27–365); His_6_-BIKE(37–345); CK2A1-His_6_(full length); CK2A2-His_6_(full length); His_10_-MBP-MEKK1/MAP3K1(1174–end); His_6_-CLK1(128–end); His_8_-PLK2(57–360); His_10_-MAP3K15(631–922); His_6_-SUMO-ASK1/MAP3K5(659–951); and His_6_-TAO2(1–350). The pACYDuet1 construct was as follows: untagged CDC37(full length).

For enhanced expression in mammalian lines cells, the DNA sequences of His_6_-GST-SBK1(full length) and Flag-His_6_-WNK3(1–434) were optimized for expression in *Homo sapiens* using GeneSmart (GenScript) and synthesized as gBlocks (Integrated DNA Technologies) carrying 16 bp overhangs to facilitate in-fusion cloning into digested pCDNA3.4 (Thermo Fisher Scientific).

To generate a mammalian expression construct for the TAK1/MAP3K7, the coding sequence for this kinase (GE Healthcare Dharmacon, MHS6278-202756930) and its binding partner TAB1 (GE Healthcare Dharmacon, MHS6278-202760135) were PCR-amplified and ligated as a fusion construct (TAK1(1–303)-TAB1(451–end)) into the mammalian expression vector pLenti-X by in-fusion.

Expression constructs purchased or obtained from other laboratories or Addgene were as follows: bacterial expression constructs for GST-VRK1(full length) and GST-VRK2(full length), in pGEX-4T, were received as gifts from P. Lazo^62^. The bacterial expression construct for mouse CDKL5-His_6_(1–352), in pET23a+, was received as a gift from S. Katayama^63^. Bacterial expression constructs for His_6_-SUMO-PDHK1(full length), His_6_-SUMO-PDHK4(full length), pGroESL (GroEL/GroES) and MBP-BCKDK(full length) were received as gifts from D. Chuang, S.-C. Tso and R. Wynn^64,65^. pProEx HTa-BRAF_16mut V600E(444–721) was a gift from M. Therrien at Université de Montréal^66^. Mammalian expression constructs for Flag-ATR(S1333A) and HA-ATRIP were provided by D. Cortez^67^. Bacterial expression constructs for DMPK1, CAMK1G, CAMK2G, PHKG2, CDKL1, GAK and lambda phosphatase were purchased from Addgene (Addgene, 1000000094)^68^.

### Expression and Purification from bacteria

Transformations were performed using BL21 Star cells (Thermo Fisher Scientific) unless specified otherwise. Antibiotic concentrations used were as follows: carbenicillin (100 mg l^−1^), kanamycin (50 mg l^−1^), spectinomycin (25 mg l^−1^) and chloramphenicol (25 mg l^−1^ in ethanol, prepared fresh). Transformed cells were grown in 1 l Terrific Broth by shaking at 190 rpm at 37 °C until the optical density (*λ *= 600 nm) reached 0.7–0.8, at which point 1 mM IPTG was added to induce expression. The cells were then transferred to a refrigerated shaker and shaken at 220 rpm at 18 °C for 16–20 h. Cells were centrifuged at 6,000*g*, and the pellets were snap-frozen in liquid nitrogen and stored at −80 °C.

All of the steps for protein purification were performed at 4 °C. Cell pellets were solubilized in lysis buffer (described below) and lysed by probe sonication. The lysate was centrifuged at 20,000*g* for 1 h and the supernatant was combined with affinity purification resin, nickel NTA (Qiagen) or glutathione Sepharose (GE Health) that had been rinsed in base buffer. The supernatant-bead slurry was agitated for 30 min. Resin was washed with 1 l base buffer and eluted in 10 bed volumes of elution buffer. Eluted protein was concentrated using the Ultra Centrifugal Filter Units (Amicon), supplemented with 1 mM DTT and 25% glycerol, and snap-frozen in liquid nitrogen and stored at −80 °C.

The buffers were as follows. Standard lysis buffer: 50 mM Tris pH 8.0, 100 mM NaCl, 2 mM MgCl_2_, 2% glycerol, HALT EDTA-free phosphatase and protease inhibitor cocktail (Life technologies), 5 mM β-mercaptoethanol and 1–3 g of lysozyme (Sigma-Aldrich). Standard base buffer: 50 mM Tris pH 8.0, 100 mM NaCl, 2 mM MgCl_2_, 2% glycerol (50 mM imidazole was included for purifications involving polyhistidine tags). Standard wash buffer: 50 mM Tris pH 8.0, 500 mM NaCl, 2 mM MgCl_2_, 2% glycerol (50 mM imidazole was included for purifications involving polyhistidine tags). Polyhistidine-tag elution buffer: 50 mM Tris pH 8.0, 100 mM NaCl, 2 mM MgCl_2_, 2% glycerol, 350 mM imidazole. GST-tag elution buffer: 50 mM Tris pH 8.0, 100 mM NaCl, 2 mM MgCl_2_, 2% glycerol, 10 mM glutathione (pH was adjusted after addition of glutathione).

CDK8 was co-purified with CCNC/MED12. CDK19 was co-purified with CCNC/MED12. CK2A1 and CK2A2 were co-purified with CK2B. ERK5 was co-expressed with MEK5DD. The kinases BRAF and NIK were co-expressed with untagged HSP90–CDC37 complex. ALPHAK3 N- and C-terminal domains were co-purified. DMPK1, CAMK1G, CAMK2G, PHKG2, CDKL1 and GAK were co-expressed with lambda phosphatase in Rosetta 2 cells (Novagen). PDHK1, PDHK4 and BCKDK were co-expressed with GroeL/GroeS and purified with the following buffers: lysis buffer (100 mM potassium phosphate pH 7.5, 10 mM l-arginine, 500 mM KCl, 0.1 mM EDTA, 0.1 mM EGTA, 0.2% Triton X-100, lysozyme), wash buffer (50 mM potassium phosphate pH 7.5, 10 mM arginine, 500 mM NaCl, 0.1% Triton X-100, 2 mM MgCl_2_), and elution buffer (25 mM Tris pH 7.5, 120 mM KCl, 0.02% Tween-20, 50 mM arginine, 350 mM imidazole for PDHK1 and PDHK4, 20 mM maltose for BCKDK). BCKDK was purified by its MBP tag on amylose resin (NEB). CDKL5 was expressed in BL21-codonplus(DE3)-RIL cells. KIS (full length) was purified as described previously^69^.

### Expression and purification from mammalian cells

Expi293F cells (Thermo Fisher Scientific) were cultured in 500 ml Expi293 Expression Medium (Thermo Fisher Scientific) in 2 l spinner flasks on a magnetic stirring platform at 100 r.c.f. at 36.8 °C under 8% CO_2_. For transfection, 500 μg of expression constructs was diluted in Opti-MEM I Reduced Serum Medium (Thermo Fisher Scientific). ExpiFectamine 293 Reagent (Thermo Fisher Scientific) was diluted with Opti-MEM separately then combined with diluted plasmid DNA for 10 min at room temperature. The mixture was then transferred to the cells (3 × 10^6^ cells per ml) and stirred. Then, 20 h after transfection, ExpiFectamine 293 Transfection Enhancer 1 and Enhancer 2 (Thermo Fisher Scientific) were added to the cells. Two days later, the cells were centrifuged at 300*g* for 5 min, snap-frozen in liquid nitrogen and stored at −80 °C (3 days after transfection).

All of the steps for protein purification were performed at 4 °C. Cell pellets were solubilized in lysis buffer and lysed by dounce homogenization (20 strokes). The lysate was centrifuged at 100,000*g* for 1 h and the supernatant was combined with affinity purification resin, nickel NTA (Qiagen), glutathione Sepharose (GE Health) or anti-Flag M2 affinity gel (Sigma-Aldrich), and agitated for 30 min (nickel and glutathione beads) or 1 h (anti-Flag beads). Resin was washed with 1 l base buffer and eluted in 10 bed volumes of elution buffer. For elution of Flag tagged-proteins, beads were immersed in elution buffer (0.15 μg ml^−1^ 3× Flag peptide (Sigma-Aldrich)) and agitated for 1 h before elution. Eluted protein was concentrated using Ultra Centrifugal Filter Units (Amicon), supplemented with 1 mM DTT and 25% glycerol, and snap-frozen in liquid nitrogen and stored at −80 °C.

Buffers were as follows. Standard lysis buffer: 50 mM Tris pH 8.0, 150 mM NaCl, 2 mM MgCl_2_, 5% glycerol, 1% Triton X-100, 5 mM β-mercaptoethanol, HALT protease inhibitors. Standard base buffer: 50 mM Tris pH 8.0, 100 mM NaCl, 2 mM MgCl_2_, 2% glycerol. Standard wash buffer: 50 mM Tris pH 8.0, 500 mM NaCl, 2 mM MgCl_2_, 2% glycerol.

His_6_–GST-tagged SBK was purified sequentially on nickel and then glutathione resins. The buffers were as follows: the first wash buffer: 25 mM imidazole. SBK1 elution buffer for polyhistidine tag: 50 mM Tris pH 8.0, 100 mM NaCl, 2 mM MgCl_2_, 2% glycerol, 250 mM imidazole. SBK1 elution buffer for GST tag: 50 mM Tris pH 8.0, 100 mM NaCl, 2 mM MgCl_2_, 2% glycerol, 10 mM glutathione. Flag–TAK1–TAB1 elution buffer: 50 mM Tris pH 8.0, 100 mM NaCl, 2 mM MgCl_2_, 2% glycerol, 0.15 μg ml^−1^ 3× Flag peptide.

Flag–His_6_–WNK3 was purified sequentially on nickel and then anti-Flag resins. The buffers were as follows: the first wash buffer: 25 mM imidazole. Flag-tag elution buffer (chloride-free): 50 mM Tris pH 7.5, 2 mM magnesium acetate, 2% glycerol, 0.15 μg ml^−1^ 3× Flag peptide.

Flag–ATR(S1333A) (350 μl) and HA–ATRIP (150 μg) were co-transfected into Expi293 cells and incubated for one additional day after addition of enhancers (4 days after transfection). The buffers were as follows. ATR lysis buffer: 50 mM HEPES pH 7.4, 150 mM NaCl, 10% glycerol, 0.25% Tween-20, 2 mM MgCl_2_, DTT. ATR wash buffer: 50 mM HEPES pH 7.4, 150 mM NaCl, 0.01% Brij-35, 2 mM MgCl_2_, 5 mM ATP, DTT. ATR elution buffer: 20 mM HEPES pH 7.4, 150 mM NaCl, 0.01% Brij-35, DTT, 0.15 μg ml^−1^ 3× Flag peptide.

Eluates were concentrated to 1 ml in 100 kDa MWCO Amicon tubes and resolved using the MonoS column in a 0–1 M NaCl gradient (buffer: 25 mM Bis-Tris pH 6.9, 0.01% Brij-35 and 5 mM TCEP). A total of 1 ml of each fraction was collected. Fractions 1–4 were combined and concentrated to 1 ml using a 100 kDa MWCO filter and resolved using size-exclusion (Superose 6) in 20 mM HEPES pH 7.4, 200 mM NaCl, 0.01% Brij-35 and 5 mM TCEP. A total of 1 ml of each fraction was collected. Fractions 11–14 were verified to be pure ATR–ATRIP complex on SDS–PAGE.

SMG1–SMG9 complexes were purified from HEK293T cells as described previously^70^. RIPK1, RIPK2 and RIPK3 were purified from insect cells (Sf9) as described previously^71^. The following recombinant active kinases obtained from other laboratories. Recombinant active CDK12–CycK, CDK13–CycK and CDK9–CycT complexes were provided as gifts from M. Geyer^72,73^. Recombinant active DCAMKL1/DCLK1 and MELK were provided as gifts from N. Gray, H.-T. Huang and K. Westover, Y. Liu and W. Harshburger^74–76^. Recombinant active PRPK(full length)–CGI121/TPRKB(full length) complex was provided as a gift from L. Wan and F. Sicheri^77^. Recombinant active HASPIN(452–798) was provided as a gift from A. Musacchio^78^. Recombinant active YSK1 was provided as a gift from X. Luo^79^. Recombinant CK1G2 was provided as a gift from S. Knapp. A list of catalogue and lot numbers of purchased recombinant kinases is provided in Supplementary Table 1.

### PSPA analysis

Recombinant kinase was added to a 384-well plate containing peptide substrate library mixtures in solution phase at 50 μM (Anaspec, AS-62017-1 and AS-62335). The reaction was initiated with the addition of 50 μM ATP (50 μCi ml^−1^ γ-^32^P-ATP, Perkin-Elmer) and incubated for 90 min. The assay conditions for each kinase are described in Supplementary Table 1 (refs. ^80–84^). After completion of the reaction, the solutions were spotted onto streptavidin-conjugated membranes (Promega, V2861), where the peptides tightly associated through their C-terminal biotinylation. The membranes were rinsed and then imaged using the Typhoon FLA 7000 phosphorimager (GE) to measure the extent of peptide phosphorylation. Raw data (GEL file) were quantified using ImageQuant (GE) to generate densitometry matrices (Supplementary Table 2). For the kinase ALPHAK3, spots were normalized to the surrounding background, owing to spatial variation in background signal. PDHK1 and PDHK4 showed dual specificity for serine and tyrosine. For these kinases, we used a customized peptide substrate library devoid of tyrosine residues at randomized positions.

In total, 283 human kinase motifs, one motif from a mouse kinase orthologue (CDKL5), one motif from a rat kinase orthologue (KIS) and one motif from an arthropod *Pediculus humanus corporis* kinase orthologue (PINK1), were combined with 17 human kinase motifs that we previously published, including AKT1^85^, SRPK1^35^, SRPK2^35^, SRPK3^35^, CK1D^35^, DYRK1A^86^, DYRK2^86^, GSK3A^86^, GSK3B^86^, CK1A^86^, CK1E^86^, CK1G1^86^, CDK10^87^, CDK2^88^, CDK3^88^, CDK18^88^ and CDK7^89^.

For the zero-control experiments in Extended Data Fig. 6, biotinylated peptides were synthesized containing only serine or threonine as the phospho-acceptor, where all nine surrounding positions contained degenerate mixtures of the 20 natural amino acids excluding serine, threonine, tyrosine and cysteine.

### Matrix processing

The densitometry matrices were column-normalized at all positions by the sum of the 17 randomized amino acids (excluding serine, threonine and cysteine), to yield PSSMs (Supplementary Table 2). PDHK1 and PDHK4 were normalized to the 16 randomized amino acids (excluding serine, threonine, cysteine and additionally tyrosine), corresponding to the uniquely customized peptide library that profiled these kinases. The cysteine row was scaled by its median to be 1/17 (1/16 for PDHK1 and PDHK4). The serine and threonine values in each position were set to be the median of that position. The ratio of serine versus threonine phospho-acceptor favourability (*S*_0_ and *T*_0_, respectively) was determined by summing the values of the serine and threonine rows in the densitometry matrix (*S*_S_ and *S*_T_, respectively), accounting for the different serine versus threonine composition of the central (1:1) and peripheral (only serine or only threonine) positions (S_ctrl_ and T_ctrl_, respectively), and then normalizing to the higher value among the two (S_0_ and T_0_, respectively, Supplementary Note 1).

### Matrix clustering

The dendrogram in Fig. 2 was generated using the normalized matrices with the 20 unmodified amino acids, as well as phosphothreonine and phosphotyrosine. The linkage matrix was computed using the SciPy package in Python (v.3.7.6), using the Ward method. Results were converted to the Newick tree format and plotted using FigTree (v.1.4.4).

### Substrate scoring

For scoring substrates, the values of the corresponding amino acids in the corresponding positions were multiplied and scaled by the probability of a random peptide (Supplementary Note 2).

For the percentile score of a substrate by a given kinase, we first computed the a priori score distribution of that kinase PSSM by scoring a reference Ser/Thr phosphoproteome comprising 82,735 identified sites^4^ using the method discussed above (Fig. 3a). The percentile score of a kinase–substrate pair is defined as the percentile ranking of the substrate within the score distribution of each kinase^34^. This value was used when analysing all of the detected phosphorylation sites for kinase enrichment.

### Kinase enrichment analysis

The single phosphorylation sites (not including multi-phosphorylated peptides) in the analysed phosphoproteomics studies were scored by all of the characterized kinases (303 Ser/Thr kinases), and their ranks in the known phosphoproteome score distribution were determined as described above. For every non-duplicate, singly phosphorylated site, kinases that ranked within the top 15 kinases for the Ser/Thr kinases were considered to be biochemically favoured kinases for that phosphorylation site. For assessing kinase motif enrichment in phosphoproteomics datasets, we compared the percentage of phosphorylation sites for which each kinase was predicted among the upregulated/downregulated (increased/decreased, respectively) phosphorylation sites (sites with |log_2_[fold change]| equal or greater than the log[fold change] threshold), versus the percentage of biochemically favoured phosphorylation sites for that kinase within the set of unregulated (unchanged) sites in this study (sites with |log_2_[fold change]| less than the log[fold change] threshold). The log-transformed fold change threshold was determined to be 1.5 for all panels in Fig. 4, except for Fig. 4e, in which the threshold was set to 0.5 owing to the low range of the log[fold change] in the data. Contingency tables were corrected using Haldane correction (adding 0.5 to the cases with zero in one of the counts). Statistical significance was determined using one-sided Fisher’s exact tests, and the corresponding *P* values were adjusted using the Benjamini–Hochberg procedure. Kinases that were significantly enriched (adjusted *P* ≤ 0.1), or depleted (log_2_[frequency factor] < 0) for both upregulated and downregulated analysis were excluded from downstream analysis. Then, for every kinase, the most significant enrichment side (upregulated or downregulated) was selected on the basis of the adjusted *P* value and presented in the volcano plots.

### Sequence logos

Sequence logos were made using logomaker package in Python^90^. For individual kinases, the normalized matrix was used, where the height of every letter is the ratio of its value to the median value of that position. The serine and threonine heights in the central position (position zero) were set to the ratio between their favourability. For clustered groups of kinases, the average matrix was calculated and presented as sequence logo as described above.

### Comparative analyses between amino acids in the kinase domains and their substrate specificities

For Extended Data Fig. 7, kinases were sorted by their log_2_[*S*_0_/*T*_0_] values. For the sequence logo, kinase domains of 290 available kinases were obtained from previously aligned kinase sequences^91^. The alignments to residues Met1–Leu296 in CDK2 (Protein Data Bank (PDB): 1QMZ) were obtained for each kinase, and the frequencies of amino acids in increments of 15-kinases were calculated and plotted as a sequence logo.

### Known kinase–substrate pairs

Experimentally validated kinase–substrate relationships were obtained from PhosphoSitePlus (July 2021). The number of reports for each pair was determined by the sum of the in vivo and in vitro reports.

### Illustrations

Experimental schema and illustrative models were generated using BioRender (https://biorender.com/). Kinome tree images were generated and modified using Coral (http://phanstiel-lab.med.unc.edu/CORAL/). Structural illustrations were generated using PyMOL. Generic kinase domains in Figs. 1 and 3 were as follows: PKAα (PDB: 1ATP). The kinase and substrate structures in Fig. 3 were as follows: ATM (PDB: 7SIC)^92^ and p53 (chimera of AlphaFold AF-P04637-F1-model_v2_1 (1–95)^61^ and 2ATA(96–292)^92^) (Fig. 3c), and PHKG2 (PDB: 2Y7J)^92^ and PYGM (PDB: 1ABB)^92^ (Fig. 3b).

### Reporting summary

Further information on research design is available in the Nature Portfolio Reporting Summary linked to this article.

## Online content

Any methods, additional references, Nature Portfolio reporting summaries, source data, extended data, supplementary information, acknowledgements, peer review information; details of author contributions and competing interests; and statements of data and code availability are available at 10.1038/s41586-022-05575-3.

## Supplementary information

Supplementary Information Supplementary Figs. 1 and 2 and Supplementary Notes 1 and 2.

Reporting Summary

Supplementary Table 1 Profiling Ser/Thr kinase substrate specificity. Experimental details for obtaining and profiling the recombinant Ser/Thr kinases in this study.

Supplementary Table 2 PSPA data and PSSMs. Raw densitometries obtained from PSPA experiments and their normalized forms.

Supplementary Table 3 Annotation of the human Ser/Thr phosphoproteome. A total of 89,752 experimentally identified Ser and Thr phosphorylation sites were scored by 303 Ser/Thr kinase PSSMs. The table enables one to sort substrates by percentile scores or ranks for given kinases or by promiscuity indices (the number of kinases scoring above the 90th percentile) or median percentile scores (Fig. 3d).

Supplementary Table 4 Motif-enrichment analysis with ATM. Serine and threonine phosphorylation sites upregulated after treatment with ionizing radiation (Fig. 4e), where ATM was ranked within the top 15 out of 303 predicted kinases.

## Data Availability

The data generated (RAW files) and analysed in this study are provided in this paper. All plasmids generated in this study have either been deposited at Addgene or available on request.

## Code availability

The analytical tools used in this study and underlying code are available to the public online (https://kinase-library.phosphosite.org).
