# Supplementary material for: An atlas of substrate specificities for the human serine/threonine kinome
Source: Nature. 2023 Jan 11;613(7945):759–66. doi: 10.1038/s41586-022-05575-3 (PMC9876800; doi:10.1038/s41586-022-05575-3)
Supplement: Supplementary file 1 — Supplementary Figs. 1 and 2 and Supplementary Notes 1 and 2. [file 41586_2022_5575_MOESM1_ESM.pdf]

nature portfolio

https://doi.org/10.1038/s41586-022-05575-3

Supplementary information

An atlas of substrate specificities for the human serine/threonine kinome

In the format provided by the authors and unedited

Supplementary Figure 1:
Compendium of experimental peptide phosphorylation motifs for the human serine/threonine kinome (compressed file)
See high resolution images at https://kinase-library.phosphosite.org/site

Click name of kinase to go to results page

GENE	PROTEIN	UNIPROT_ID
AAK1	AAK1	Q2M2I8
ACVR1	ALK2	Q04771
ACVR1B	ALK4	P36896
ACVR2A	ACVR2A	P27037
ACVR2B	ACVR2B	Q13705
AKT1	AKT1	P31749
AKT2	AKT2	P31751
AKT3	AKT3	Q9Y243
ALPK1	ALPHAK3	Q96QP1
ATM	ATM	Q13315
ATR	ATR	Q13535
AURKA	AURA	O14965
AURKB	AURB	Q96GD4
AURKC	AURC	Q9UQB9
BCKDK	BCKDK	O14874
BMP2K	BIKE	Q9NSY1
BMPR1A	BMPR1A	P36894
BMPR1B	BMPR1B	O00238
BMPR2	BMPR2	Q13873
BRAF	BRAF	P15056
BRSK1	BRSK1	Q8TDC3
BRSK2	BRSK2	Q8IWQ3
BUB1	BUB1	O43683
CAMK1	CAMK1A	Q14012
CAMK1D	CAMK1D	Q8IU85
CAMK1G	CAMK1G	Q96NX5
CAMK2A	CAMK2A	Q9UQM7
CAMK2B	CAMK2B	Q13554
CAMK2D	CAMK2D	Q13557
CAMK2G	CAMK2G	Q13555
CAMK4	CAMK4	Q16566
CAMKK1	CAMKK1	Q8N5S9
CAMKK2	CAMKK2	Q96RR4
CDC42BPA	MRCKA	Q5VT25
CDC42BPB	MRCKB	Q9Y5S2
CDC7	CDC7	O00311
CDK1	CDK1	P06493
CDK10	CDK10	Q15131
CDK12	CDK12	Q9NYV4
CDK13	CDK13	Q14004
CDK14	CDK14	O94921
CDK16	CDK16	Q00536
CDK17	CDK17	Q00537
CDK18	CDK18	Q07002
CDK19	CDK19	Q9BWU1
CDK2	CDK2	P24941
CDK3	CDK3	Q00526

GENE	PROTEIN	UNIPROT_ID
CDK4	CDK4	P11802
CDK5	CDK5	Q00535
CDK6	CDK6	Q00534
CDK7	CDK7	P50613
CDK8	CDK8	P49336
CDK9	CDK9	P50750
CDKL1	CDKL1	Q00532
CDKL5	CDKL5	Q3UTQ8
CHEK1	CHK1	O14757
CHEK2	CHK2	O96017
CHUK	IKKA	O15111
CILK1	ICK	Q9UPZ9
CIT	CRIK	O14578
CLK1	CLK1	P49759
CLK2	CLK2	P49760
CLK3	CLK3	P49761
CLK4	CLK4	Q9HAZ1
CSNK1A1	CK1A	P48729
CSNK1A1L	CK1A2	Q8N752
CSNK1D	CK1D	P48730
CSNK1E	CK1E	P49674
CSNK1G1	CK1G1	Q9HCP0
CSNK1G2	CK1G2	P78368
CSNK1G3	CK1G3	Q9Y6M4
CSNK2A1	CK2A1	P68400
CSNK2A2	CK2A2	P19784
DAPK1	DAPK1	P53355
DAPK2	DAPK2	Q9UIK4
DAPK3	DAPK3	O43293
DCLK1	DCAMKL1	O15075
DCLK2	DCAMKL2	Q8N568
DMPK	DMPK1	Q09013
DSTYK	DSTYK	Q6XUX3
DYRK1A	DYRK1A	Q13627
DYRK1B	DYRK1B	Q9Y463
DYRK2	DYRK2	Q92630
DYRK3	DYRK3	O43781
DYRK4	DYRK4	Q9NR20
EEF2K	EEF2K	O00418
EIF2AK1	HRI	Q9BQI3
EIF2AK2	PKR	P19525
EIF2AK3	PERK	Q9NZJ5
EIF2AK4	GCN2	Q9P2K8
ERN1	IRE1	O75460
ERN2	IRE2	Q76MJ5
FAM20C	FAM20C	Q8IXL6
GAK	GAK	O14976

GENE	PROTEIN	UNIPROT_ID
GRK1	GRK1	Q15835
GRK2	GRK2	P25098
GRK3	GRK3	P35626
GRK4	GRK4	P32298
GRK5	GRK5	P34947
GRK6	GRK6	P43250
GRK7	GRK7	Q8WTQ7
GSK3A	GSK3A	P49840
GSK3B	GSK3B	P49841
HASPIN	HASPIN	Q8TF76
HIPK1	HIPK1	Q86Z02
HIPK2	HIPK2	Q9H2X6
HIPK3	HIPK3	Q9H422
HIPK4	HIPK4	Q8NE63
HUNK	HUNK	P57058
IKBKB	IKKB	O14920
IKBKE	IKKE	Q14164
IRAK1	IRAK1	P51617
IRAK4	IRAK4	Q9NWZ3
LATS1	LATS1	O95835
LATS2	LATS2	Q9NRM7
LRRK2	LRRK2	Q5S007
MAK	MAK	P20794
MAP2K1	MEK1	Q02750
MAP2K2	MEK2	P36507
MAP2K5	MEK5	Q13163
MAP3K1	MEKK1	Q13233
MAP3K10	MLK2	Q02779
MAP3K11	MLK3	Q16584
MAP3K12	DLK	Q12852
MAP3K14	NIK	Q99558
MAP3K15	MAP3K15	Q6ZN16
MAP3K19	YSK4	Q56UN5
MAP3K2	MEKK2	Q9Y2U5
MAP3K20	ZAK	Q9NYL2
MAP3K21	MLK4	Q5TCX8
MAP3K3	MEKK3	Q99759
MAP3K5	ASK1	Q99683
MAP3K6	MEKK6	O95382
MAP3K7	TAK1	O43318
MAP3K8	COT	P41279
MAP3K9	MLK1	P80192
MAP4K1	HPK1	Q92918
MAP4K2	GCK	Q12851
MAP4K3	KHS2	Q8IVH8
MAP4K4	HGK	O95819
MAP4K5	KHS1	Q9Y4K4
MAPK1	ERK2	P28482
MAPK10	JNK3	P53779
MAPK11	P38B	Q15759

GENE	PROTEIN	UNIPROT_ID
MAPK12	P38G	P53778
MAPK13	P38D	O15264
MAPK14	P38A	Q16539
MAPK15	ERK7	Q8TD08
MAPK3	ERK1	P27361
MAPK7	ERK5	Q13164
MAPK8	JNK1	P45983
MAPK9	JNK2	P45984
MAPKAPK2	MAPKAPK2	P49137
MAPKAPK3	MAPKAPK3	Q16644
MAPKAPK5	MAPKAPK5	Q8IW41
MARK1	MARK1	Q9P0L2
MARK2	MARK2	Q7KZI7
MARK3	MARK3	P27448
MARK4	MARK4	Q96L34
MASTL	MASTL	Q96GX5
MELK	MELK	Q14680
MINK1	MINK	Q8N4C8
MKNK1	MNK1	Q9BUB5
MKNK2	MNK2	Q9HBH9
MOK	MOK	Q9UQ07
MOS	MOS	P00540
MTOR	MTOR	P42345
MYLK	SMMLCK	Q15746
MYLK2	SKMLCK	Q9H1R3
MYLK3	CAMLCK	Q32MK0
MYLK4	MYLK4	Q86YV6
MYO3A	MYO3A	Q8NEV4
MYO3B	MYO3B	Q8WXR4
NEK1	NEK1	Q96PY6
NEK11	NEK11	Q8NG66
NEK2	NEK2	P51955
NEK3	NEK3	P51956
NEK4	NEK4	P51957
NEK5	NEK5	Q6P3R8
NEK6	NEK6	Q9HC98
NEK7	NEK7	Q8TDX7
NEK8	NEK8	Q86SG6
NEK9	NEK9	Q8TD19
NIM1K	NIM1	Q8IY84
NLK	NLK	Q9UBE8
NUAK1	NUAK1	O60285
NUAK2	NUAK2	Q9H093
OXSR1	OSR1	O95747
PAK1	PAK1	Q13153
PAK2	PAK2	Q13177
PAK3	PAK3	O75914
PAK4	PAK4	O96013
PAK5	PAK5	Q9P286
PAK6	PAK6	Q9NQU5

GENE	PROTEIN	UNIPROT_ID
PASK	PASK	Q96RG2
PBK	PBK	Q96KB5
PDK1	PDHK1	Q15118
PDK4	PDHK4	Q16654
PDPK1	PDK1	O15530
PHKG1	PHKG1	Q16816
PHKG2	PHKG2	P15735
PIM1	PIM1	P11309
PIM2	PIM2	Q9P1W9
PIM3	PIM3	Q86V86
PINK1	PINK1	E0W1I1
PKN1	PKN1	Q16512
PKN2	PKN2	Q16513
PKN3	PKN3	Q6P5Z2
PLK1	PLK1	P53350
PLK2	PLK2	Q9NYY3
PLK3	PLK3	Q9H4B4
PLK4	PLK4	O00444
PNCK	CAMK1B	Q6P2M8
PRKAA1	AMPKA1	Q13131
PRKAA2	AMPKA2	P54646
PRKACA	PKACA	P17612
PRKACB	PKACB	P22694
PRKACG	PKACG	P22612
PRKCA	PKCA	P17252
PRKCB	PKCB	P05771
PRKCD	PKCD	Q05655
PRKCE	PKCE	Q02156
PRKCG	PKCG	P05129
PRKCH	PKCH	P24723
PRKCI	PKCI	P41743
PRKCQ	PKCT	Q04759
PRKCZ	PKCZ	Q05513
PRKD1	PRKD1	Q15139
PRKD2	PRKD2	Q9BZL6
PRKD3	PRKD3	O94806
PRKDC	DNAPK	P78527
PRKG1	PKG1	Q13976
PRKG2	PKG2	Q13237
PRKX	PRKX	P51817
PRPF4B	PRP4	Q13523
RAF1	RAF1	P04049
RIPK1	RIPK1	Q13546
RIPK2	RIPK2	O43353
RIPK3	RIPK3	Q9Y572
RIPK4	ANKRD3	P57078
ROCK1	ROCK1	Q13464
ROCK2	ROCK2	O75116
RPS6KA1	P90RSK	Q15418
RPS6KA2	RSK3	Q15349

GENE	PROTEIN	UNIPROT_ID
RPS6KA3	RSK2	P51812
RPS6KA4	MSK2	O75676
RPS6KA5	MSK1	O75582
RPS6KA6	RSK4	Q9UK32
RPS6KB1	P70S6K	P23443
RPS6KB2	P70S6KB	Q9UBS0
SBK1	SBK	Q52WX2
SGK1	SGK1	O00141
SGK3	SGK3	Q96BR1
SIK1	SIK	P57059
SIK2	QIK	Q9H0K1
SIK3	QSK	Q9Y2K2
SLK	SLK	Q9H2G2
SMG1	SMG1	Q96Q15
SNRK	SNRK	Q9NRH2
SRPK1	SRPK1	Q96SB4
SRPK2	SRPK2	P78362
SRPK3	SRPK3	Q9UPE1
STK10	LOK	O94804
STK11	LKB1	Q15831
STK16	MPSK1	O75716
STK17A	DRAK1	Q9UEE5
STK24	MST3	Q9Y6E0
STK25	YSK1	O00506
STK26	MST4	Q9P289
STK3	MST2	Q13188
STK32B	YANK2	Q9NY57
STK32C	YANK3	Q86UX6
STK33	STK33	Q9BYT3
STK38	NDR1	Q15208
STK38L	NDR2	Q9Y2H1
STK39	STLK3	Q9UEW8
STK4	MST1	Q13043
TAOK1	TAO1	Q7L7X3
TAOK2	TAO2	Q9UL54
TAOK3	TAO3	Q9H2K8
TBK1	TBK1	Q9UHD2
TGFBR1	TGFBR1	P36897
TGFBR2	TGFBR2	P37173
TLK1	TLK1	Q9UKI8
TLK2	TLK2	Q86UE8
TNIK	TNIK	Q9UKE5
TP53RK	PRPK	Q96S44
TRPM6	CHAK2	Q9BX84
TRPM7	CHAK1	Q96QT4
TSSK1B	TSSK1	Q9BXA7
TSSK2	TSSK2	Q96PF2
TSSK6	SSTK	Q9BXA6
TTBK1	TTBK1	Q5TCY1
TTBK2	TTBK2	Q6IQ55

GENE	PROTEIN	UNIPROT_ID
TTK	TTK	P33981
UHMK1	KIS	Q8TAS1
ULK1	ULK1	O75385
ULK2	ULK2	Q8IYT8
VRK1	VRK1	Q99986
VRK2	VRK2	Q86Y07
WNK1	WNK1	Q9H4A3
WNK3	WNK3	Q9BYP7
WNK4	WNK4	Q96J92

AGC

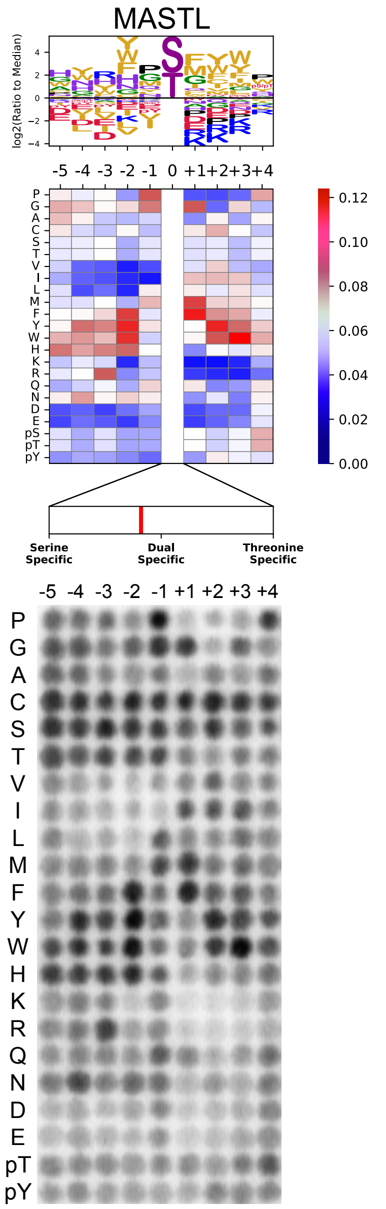
MASTL
log2(Ratio to Median)
-5 -4 -3 -2 -1 0 +1 +2 +3 +4
P G A C S T V I L M F Y W H K R Q N D E pS pT pY
0.12
0.10
0.08
0.06
0.04
0.02
0.00
Serine Specific
Dual Specific
Threonine Specific
-5 -4 -3 -2 -1 +1 +2 +3 +4
P G A C S T V I L M F Y W H K R Q N D E pT pY

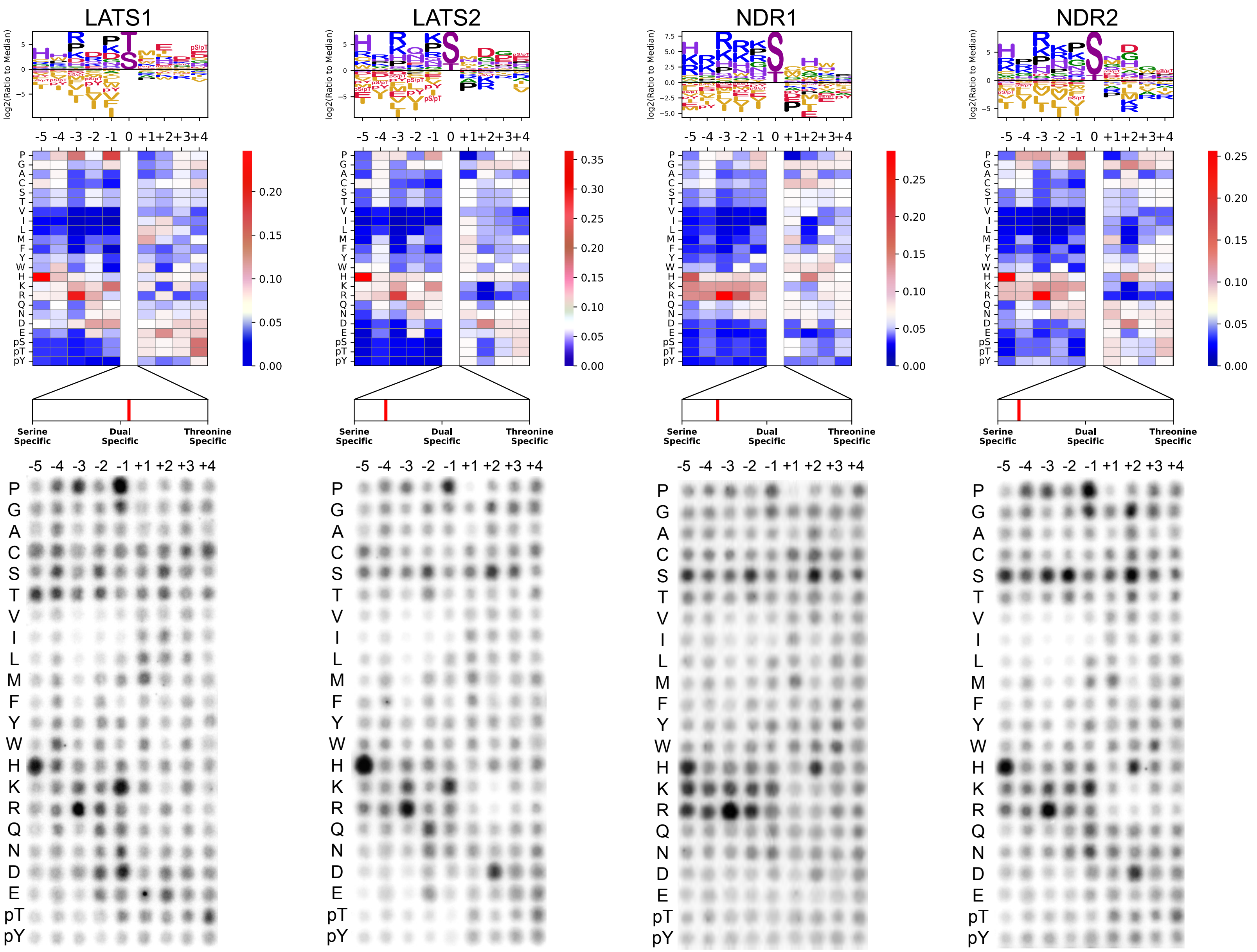
LATS1
LATS2
NDR1
NDR2
log2(Ratio to Median)
-5 -4 -3 -2 -1 0 +1 +2 +3 +4
P G A C S T V I L M F Y W H K R Q N D E pS pT pY
Serine Specific
Dual Specific
Threonine Specific
-5 -4 -3 -2 -1 +1 +2 +3 +4
P G A C S T V I L M F Y W H K R Q N D E pT pY

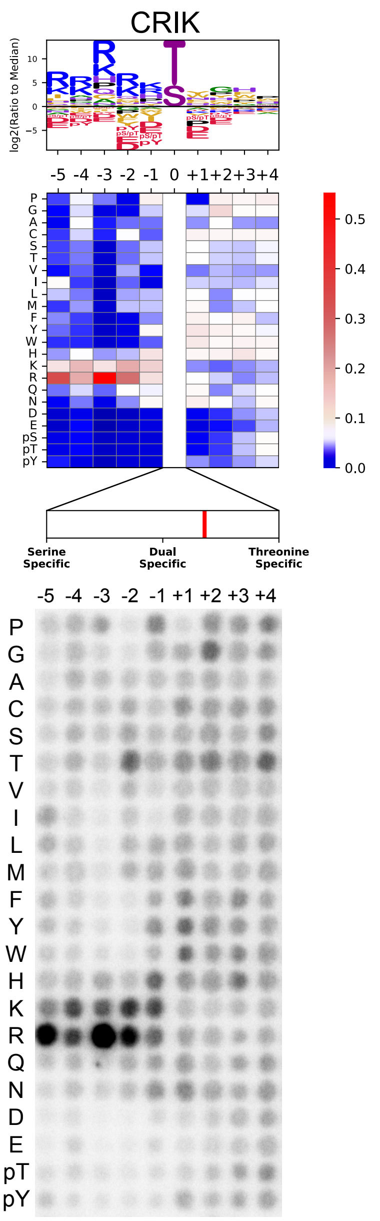

CRIK
log2(Ratio to Median)
-5 -4 -3 -2 -1 0 +1 +2 +3 +4
P G A C S T V I L M F Y W H K R Q N D E pS pT pY
Serine Specific
Dual Specific
Threonine Specific
-5 -4 -3 -2 -1 +1 +2 +3 +4
P G A C S T V I L M F Y W H K R Q N D E pT pY

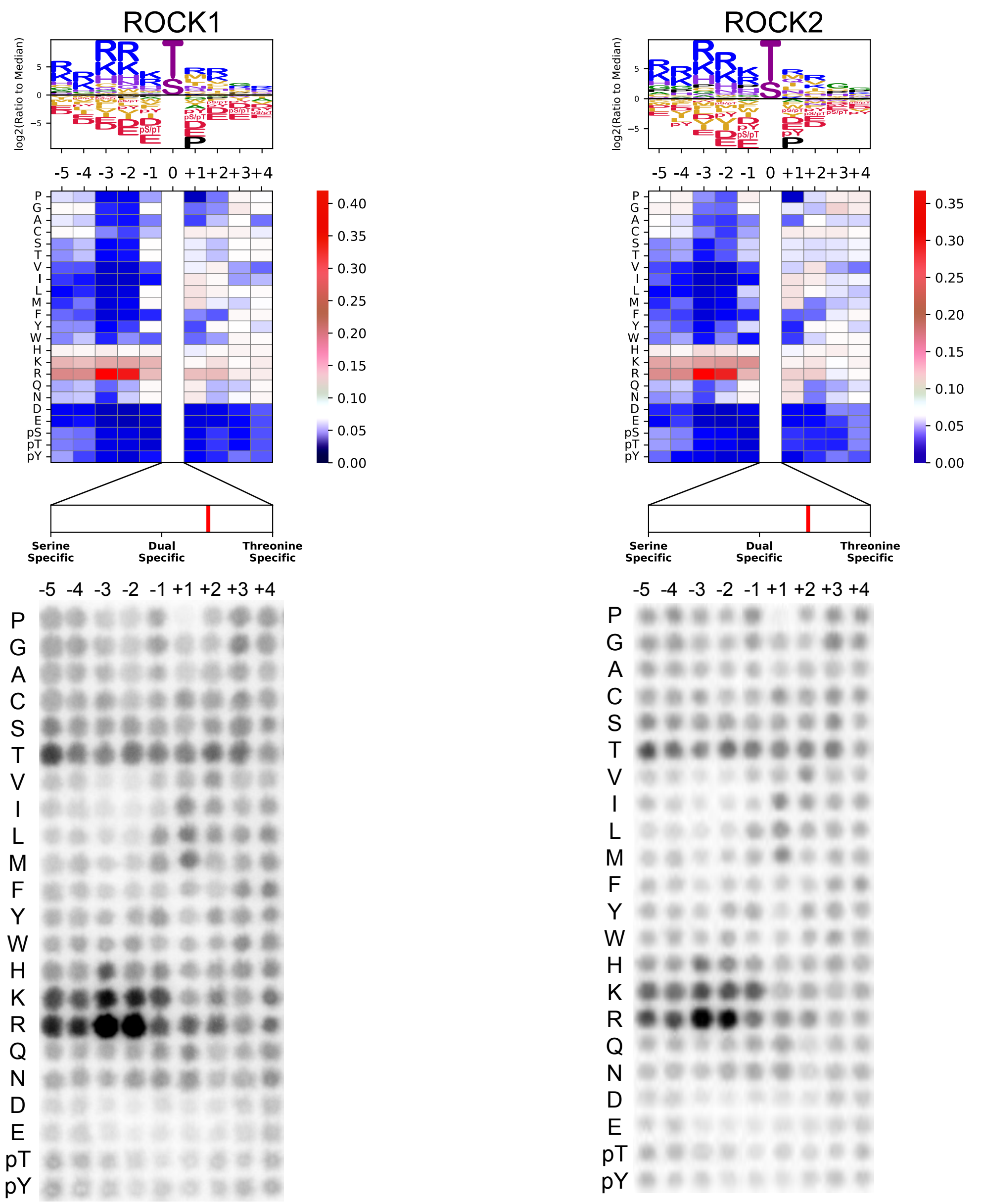
ROCK1
ROCK2
log2(Ratio to Median)
-5 -4 -3 -2 -1 0 +1 +2 +3 +4
P G A C S T V I L M F Y W H K R Q N D E pS pT pY
Serine Specific
Dual Specific
Threonine Specific
P G A C S T V I L M F Y W H K R Q N D E pT pY

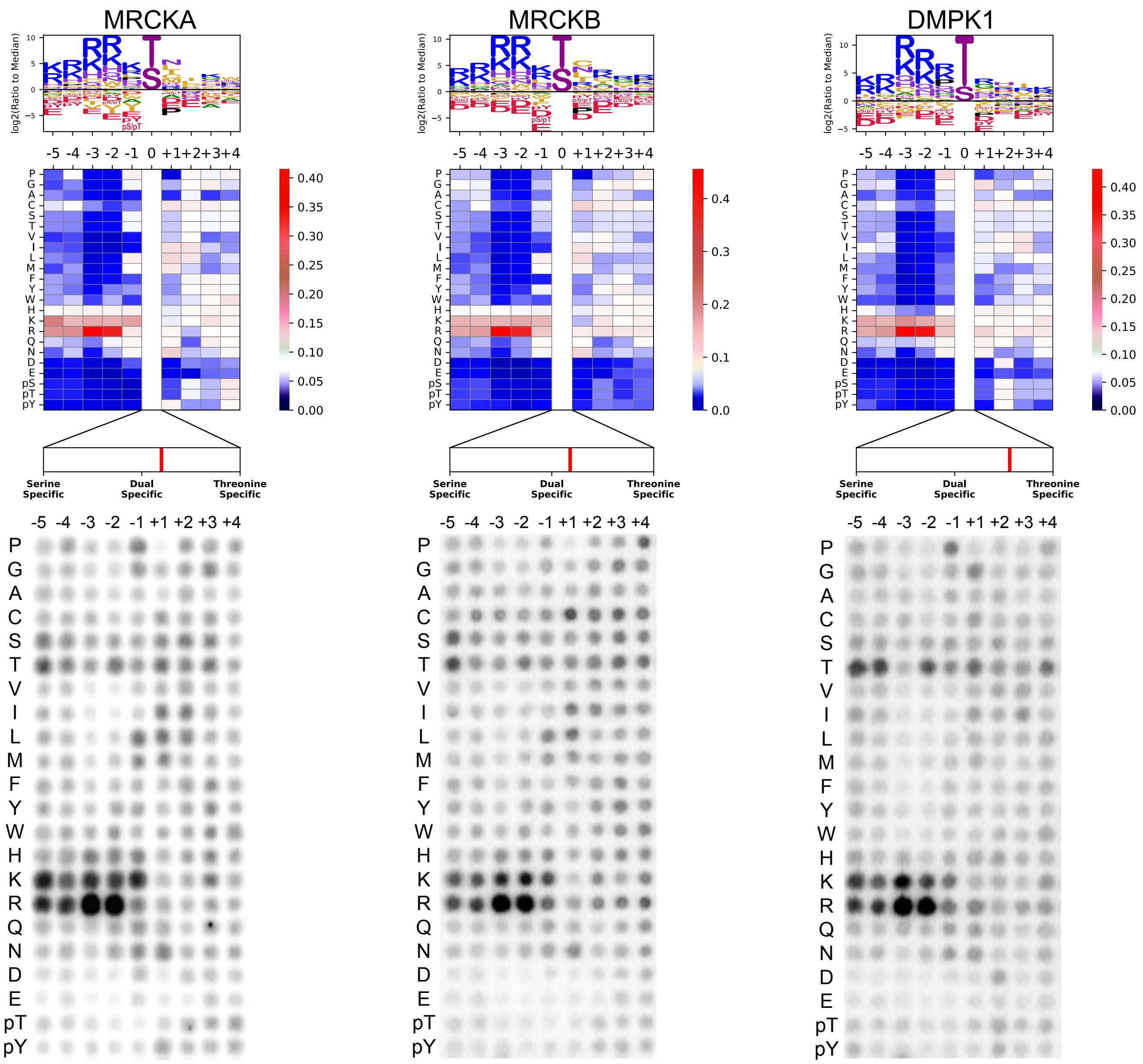
MRCKA
MRCKB
DMPK1
log2(Ratio to Median)
-5 -4 -3 -2 -1 0 +1 +2 +3 +4
P G A C S T V I L M F Y W H K R Q N D E pS pT pY
0.00 0.05 0.10 0.15 0.20 0.25 0.30 0.35 0.40
0.0 0.1 0.2 0.3 0.4
Serine Specific
Dual Specific
Threonine Specific
-5 -4 -3 -2 -1 +1 +2 +3 +4
P G A C S T V I L M F Y W H K R Q N D E pT pY

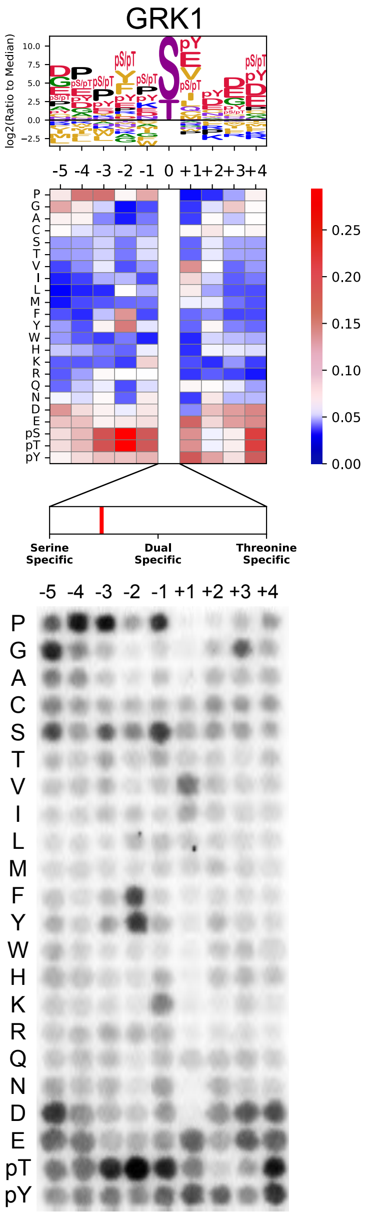
GRK1
log2(Ratio to Median)
-5 -4 -3 -2 -1 0 +1 +2 +3 +4
P G A C S T V I L M F Y W H K R Q N D E pS pT pY
0.00 0.05 0.10 0.15 0.20 0.25
Serine Specific
Dual Specific
Threonine Specific
-5 -4 -3 -2 -1 +1 +2 +3 +4
P G A C S T V I L M F Y W H K R Q N D E pT pY

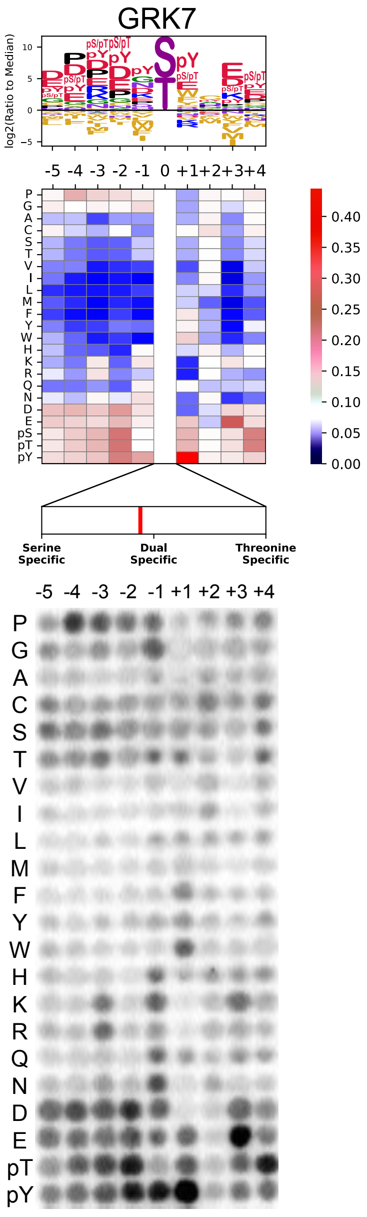
GRK7
log2(Ratio to Median)
-5 -4 -3 -2 -1 0 +1 +2 +3 +4
P G A C S T V I L M F Y W H K R Q N D E pS pT pY
0.00 0.05 0.10 0.15 0.20 0.25 0.30 0.35 0.40
Serine Specific
Dual Specific
Threonine Specific
-5 -4 -3 -2 -1 +1 +2 +3 +4
P G A C S T V I L M F Y W H K R Q N D E pT pY

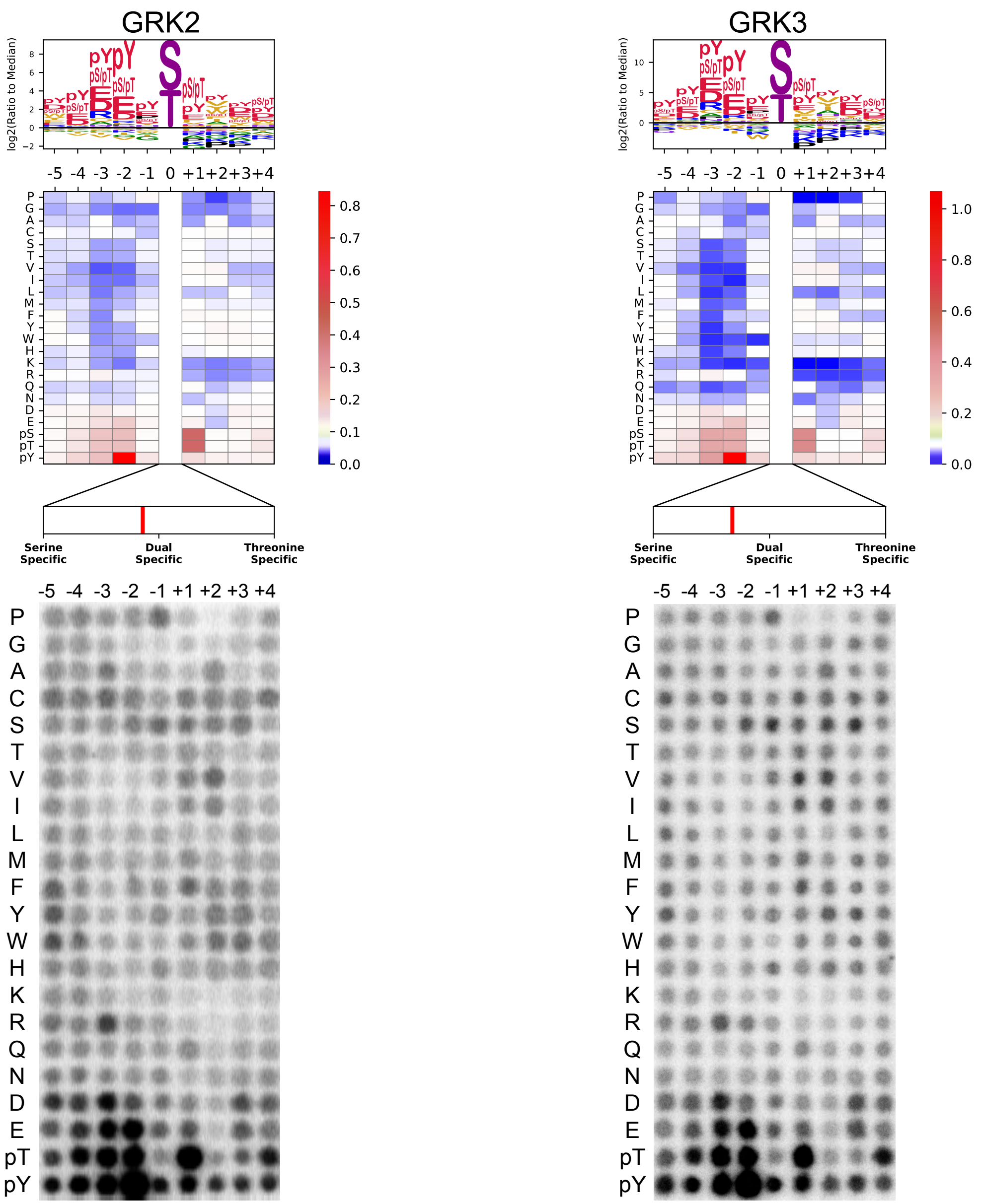
GRK2
GRK3
log2(Ratio to Median)
-5 -4 -3 -2 -1 0 +1 +2 +3 +4
P G A C S T V I L M F Y W H K R Q N D E pS pT pY
Serine Specific
Dual Specific
Threonine Specific
-5 -4 -3 -2 -1 +1 +2 +3 +4
P G A C S T V I L M F Y W H K R Q N D E pT pY

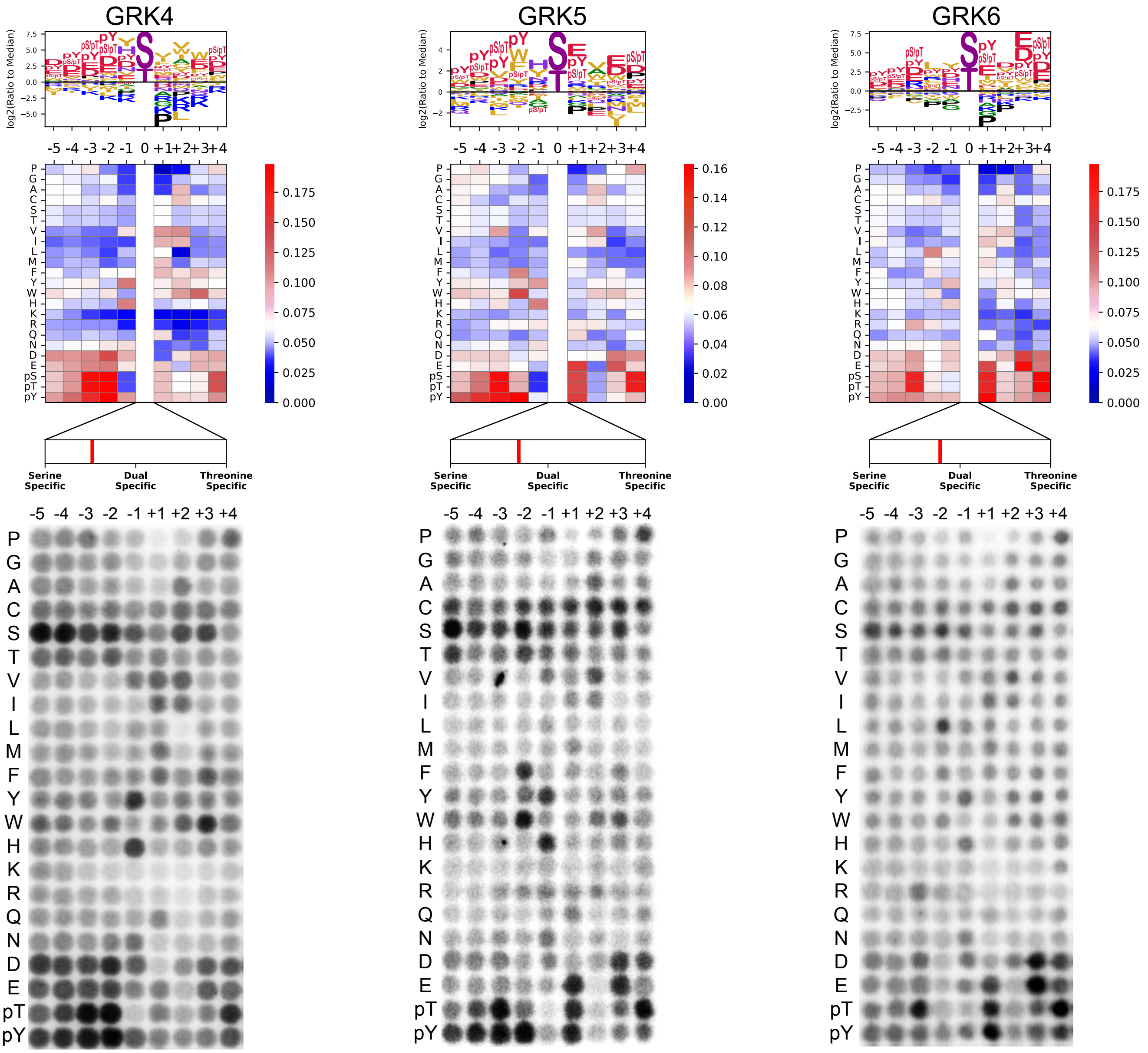
GRK4
GRK5
GRK6
log2(Ratio to Median)
-5 -4 -3 -2 -1 0 +1 +2 +3 +4
P G A C S T V I L M F Y W H K R Q N D E pS pT pY
Serine Specific
Dual Specific
Threonine Specific
-5 -4 -3 -2 -1 +1 +2 +3 +4
P G A C S T V I L M F Y W H K R Q N D E pT pY

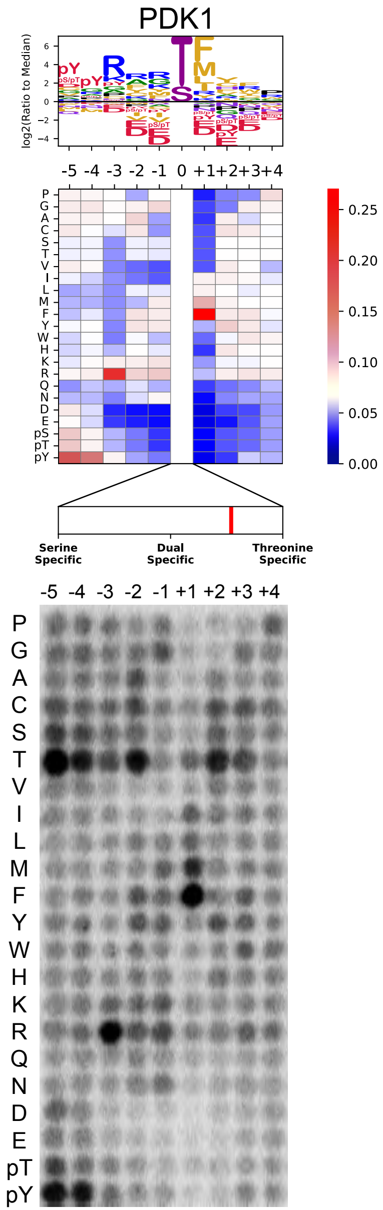

PDK1
log2(Ratio to Median)
-5 -4 -3 -2 -1 0 +1 +2 +3 +4
P G A C S T V I L M F Y W H K R Q N D E pS pT pY
0.25 0.20 0.15 0.10 0.05 0.00
Serine Specific
Dual Specific
Threonine Specific
-5 -4 -3 -2 -1 +1 +2 +3 +4
P G A C S T V I L M F Y W H K R Q N D E pT pY

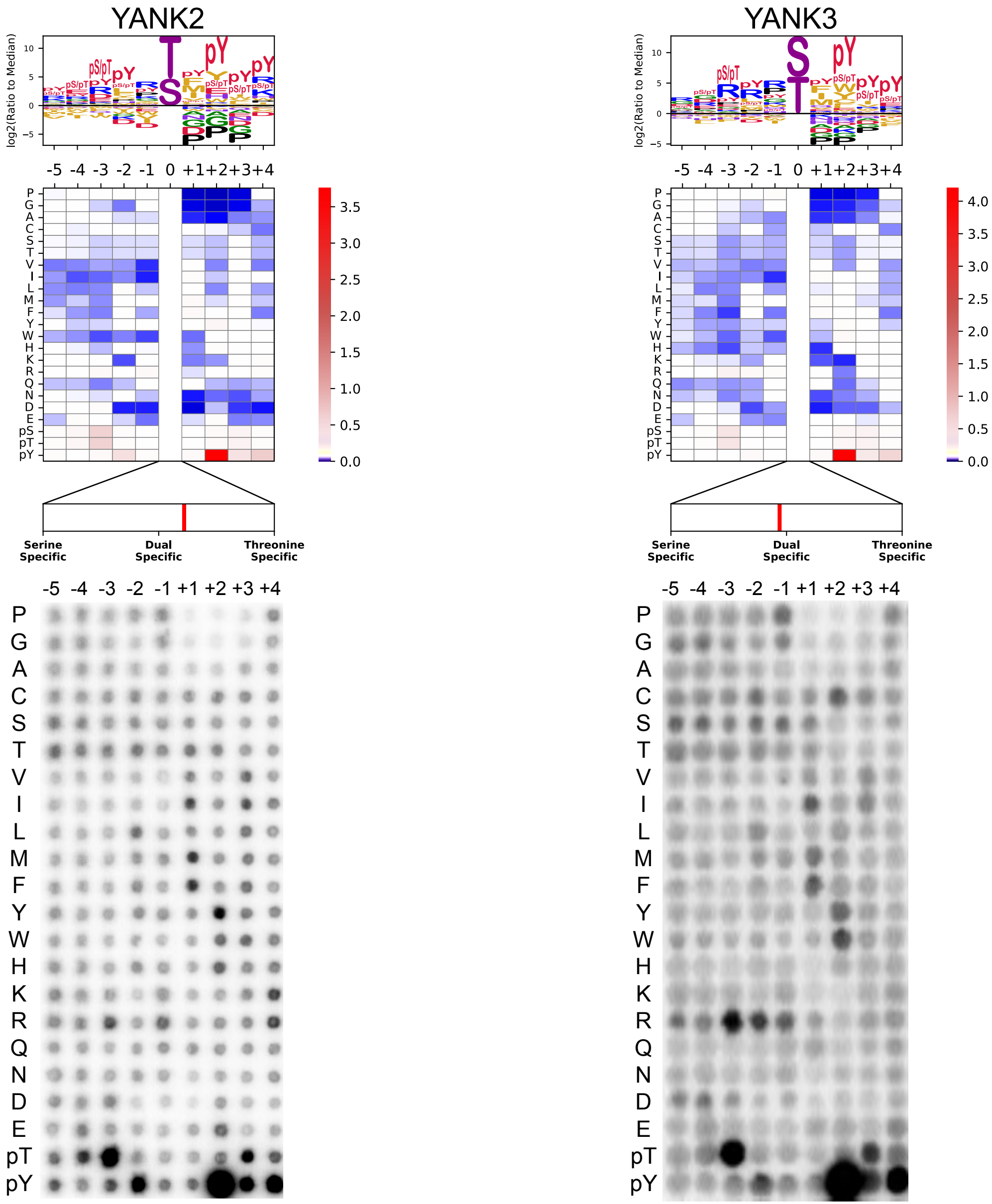

YANK2
YANK3
log2(Ratio to Median)
-5 -4 -3 -2 -1 0 +1 +2 +3 +4
P G A C S T V I L M F Y W H K R Q N D E pS pT pY
Serine Specific
Dual Specific
Threonine Specific
-5 -4 -3 -2 -1 +1 +2 +3 +4
P G A C S T V I L M F Y W H K R Q N D E pT pY

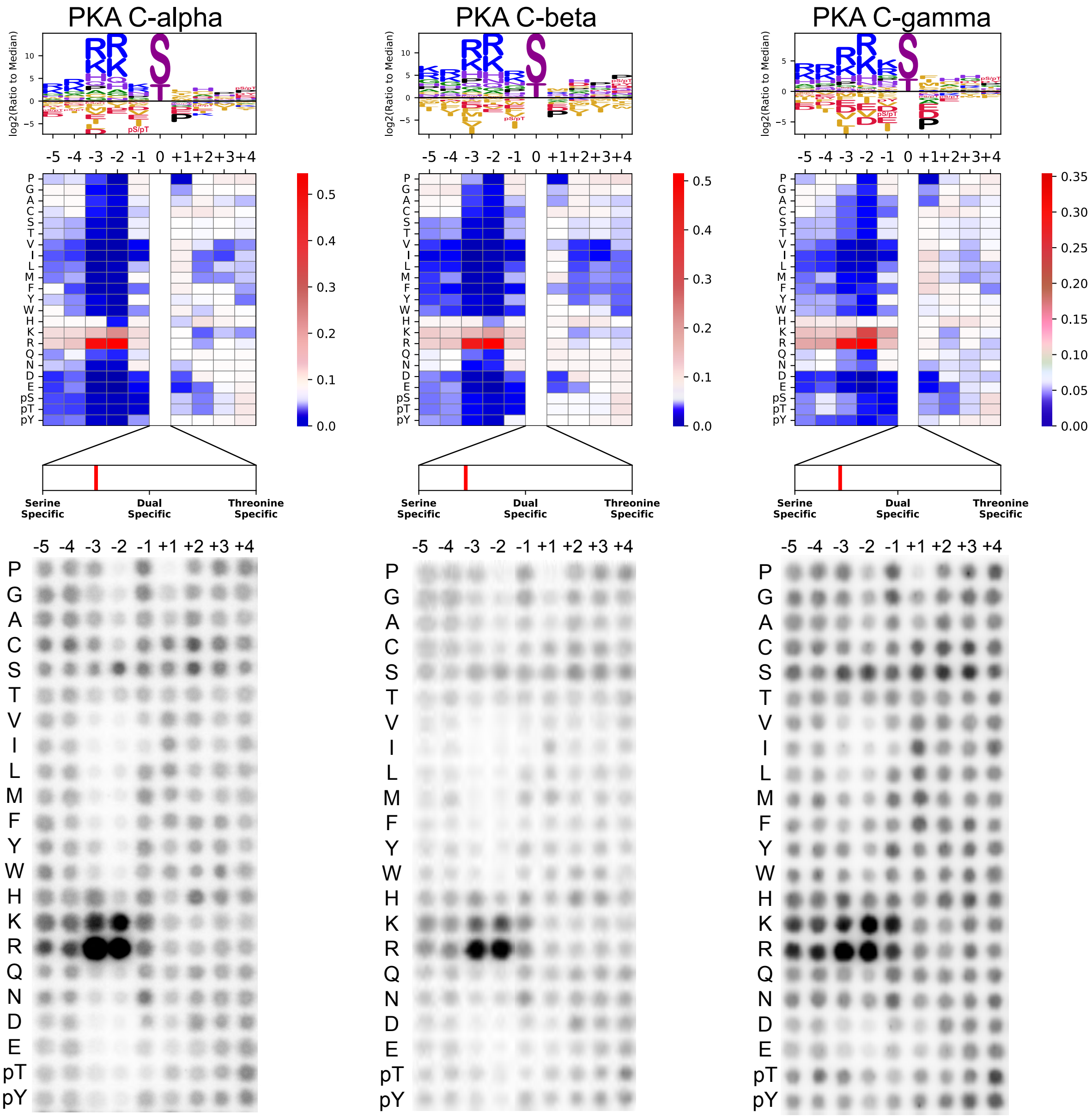
PKA C-alpha
PKA C-beta
PKA C-gamma
log2(Ratio to Median)
-5 -4 -3 -2 -1 0 +1 +2 +3 +4
P G A C S T V I L M F Y W H K R Q N D E pS pT pY
Serine Specific
Dual Specific
Threonine Specific
-5 -4 -3 -2 -1 +1 +2 +3 +4
P G A C S T V I L M F Y W H K R Q N D E pT pY

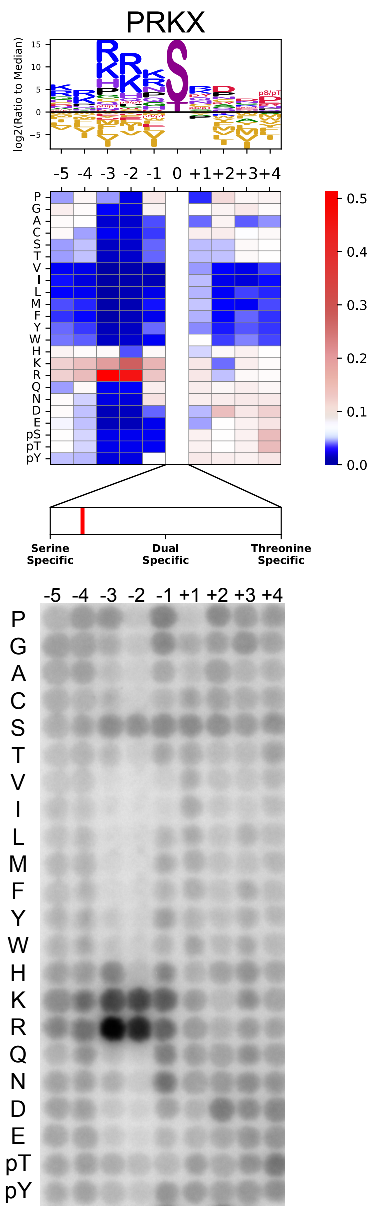
PRKX
log2(Ratio to Median)
-5 -4 -3 -2 -1 0 +1 +2 +3 +4
P G A C S T V I L M F Y W H K R Q N D E pS pT pY
0.5 0.4 0.3 0.2 0.1 0.0
Serine Specific
Dual Specific
Threonine Specific
-5 -4 -3 -2 -1 +1 +2 +3 +4
P G A C S T V I L M F Y W H K R Q N D E pT pY

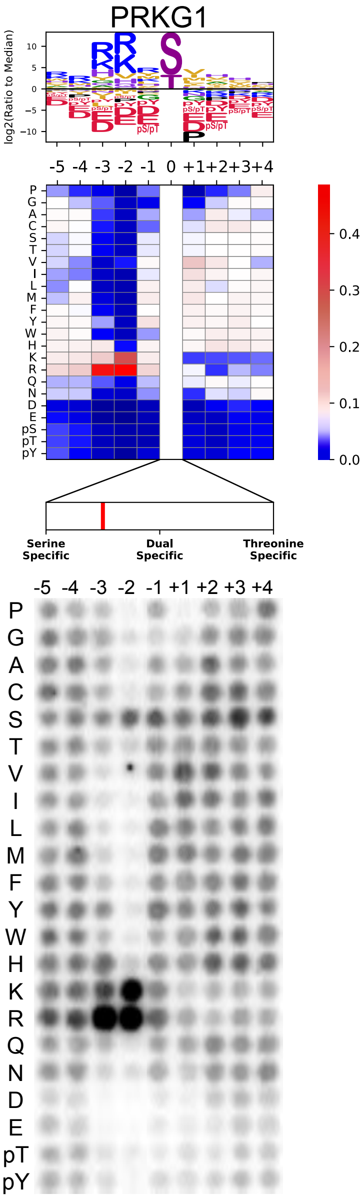
PRKG1
log2(Ratio to Median)
-5 -4 -3 -2 -1 0 +1 +2 +3 +4
P G A C S T V I L M F Y W H K R Q N D E pS pT pY
0.0 0.1 0.2 0.3 0.4
Serine Specific
Dual Specific
Threonine Specific
-5 -4 -3 -2 -1 +1 +2 +3 +4
P G A C S T V I L M F Y W H K R Q N D E pT pY

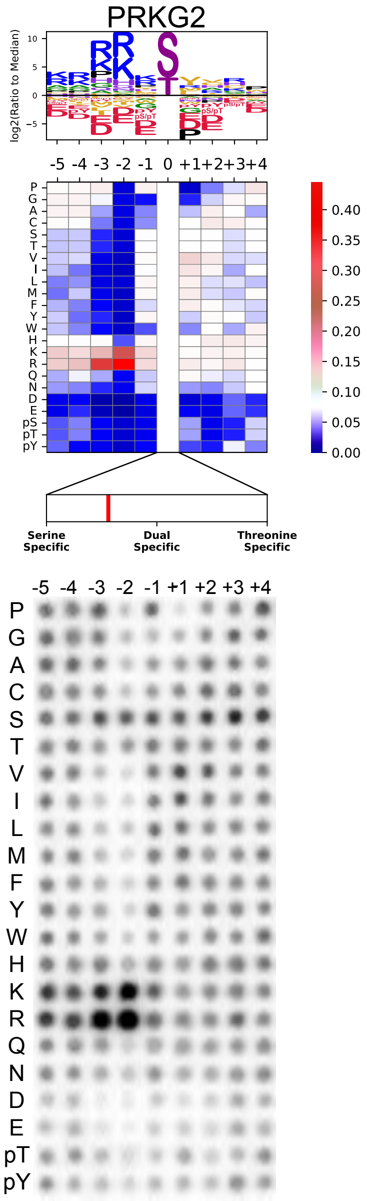
PRKG2
log2(Ratio to Median)
-5 -4 -3 -2 -1 0 +1 +2 +3 +4
P G A C S T V I L M F Y W H K R Q N D E pS pT pY
0.00 0.05 0.10 0.15 0.20 0.25 0.30 0.35 0.40
Serine Specific
Dual Specific
Threonine Specific
-5 -4 -3 -2 -1 +1 +2 +3 +4
P G A C S T V I L M F Y W H K R Q N D E pT pY

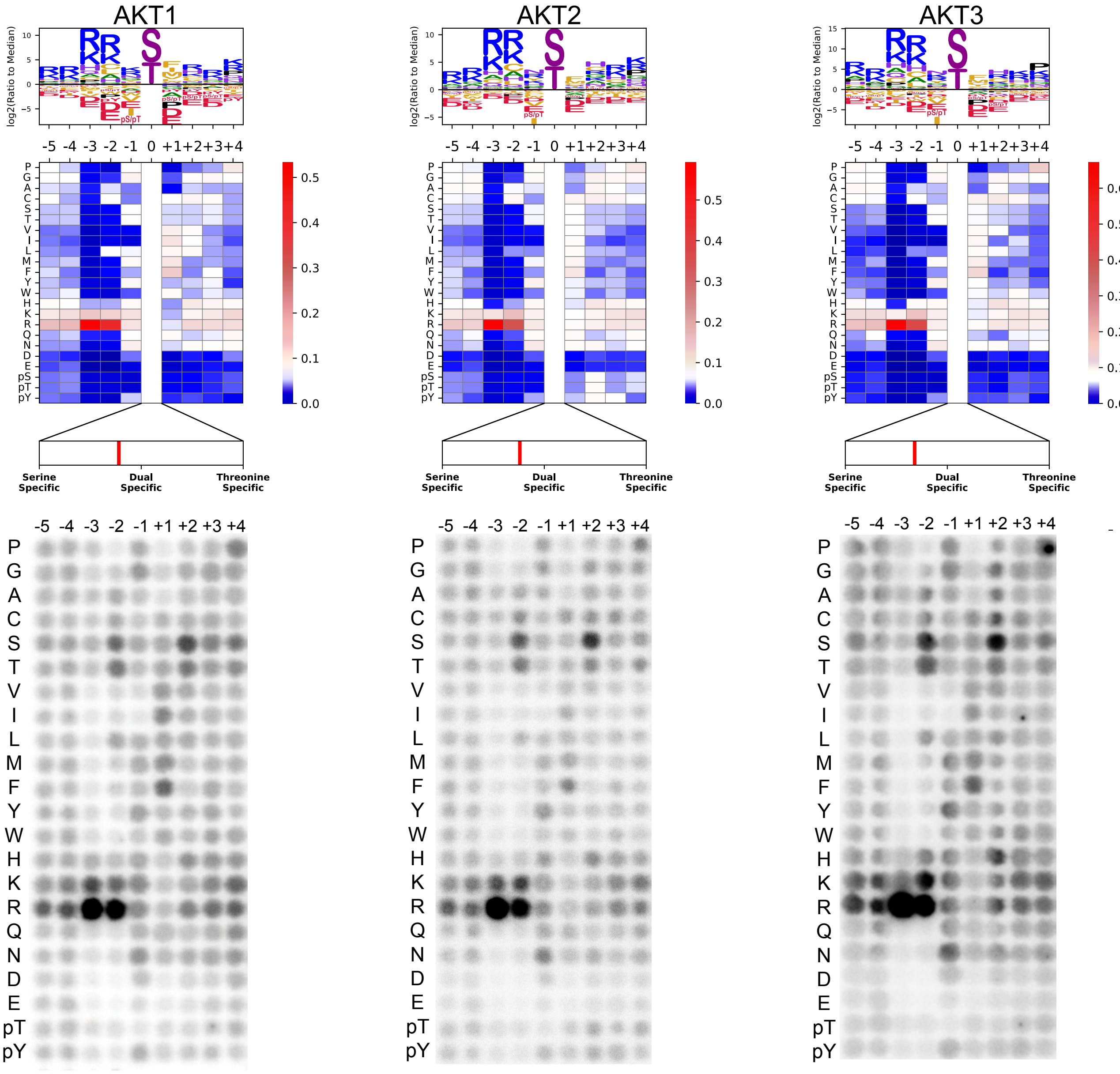
AKT1
AKT2
AKT3
log2(Ratio to Median)
-5 -4 -3 -2 -1 0 +1 +2 +3 +4
P G A C S T V I L M F Y W H K R Q N D E pS pT pY
Serine Specific
Dual Specific
Threonine Specific
-5 -4 -3 -2 -1 +1 +2 +3 +4
P G A C S T V I L M F Y W H K R Q N D E pT pY

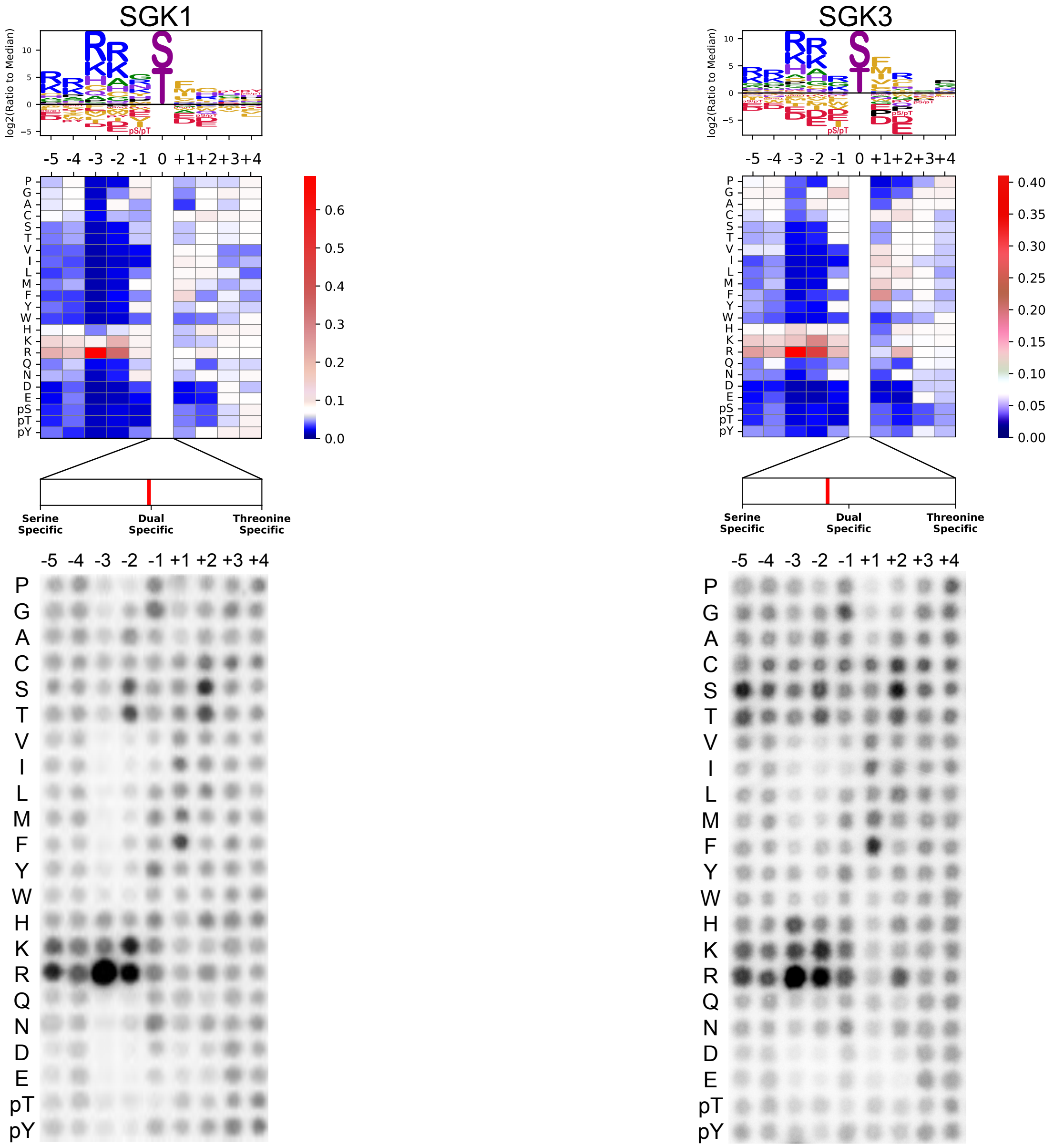
SGK1
log2(Ratio to Median)
-5 -4 -3 -2 -1 0 +1 +2 +3 +4
P G A C S T V I L M F Y W H K R Q N D E pS pT pY
0.0 0.1 0.2 0.3 0.4 0.5 0.6
Serine Specific
Dual Specific
Threonine Specific
-5 -4 -3 -2 -1 +1 +2 +3 +4
P G A C S T V I L M F Y W H K R Q N D E pT pY
SGK3
log2(Ratio to Median)
-5 -4 -3 -2 -1 0 +1 +2 +3 +4
P G A C S T V I L M F Y W H K R Q N D E pS pT pY
0.00 0.05 0.10 0.15 0.20 0.25 0.30 0.35 0.40
Serine Specific
Dual Specific
Threonine Specific
-5 -4 -3 -2 -1 +1 +2 +3 +4
P G A C S T V I L M F Y W H K R Q N D E pT pY

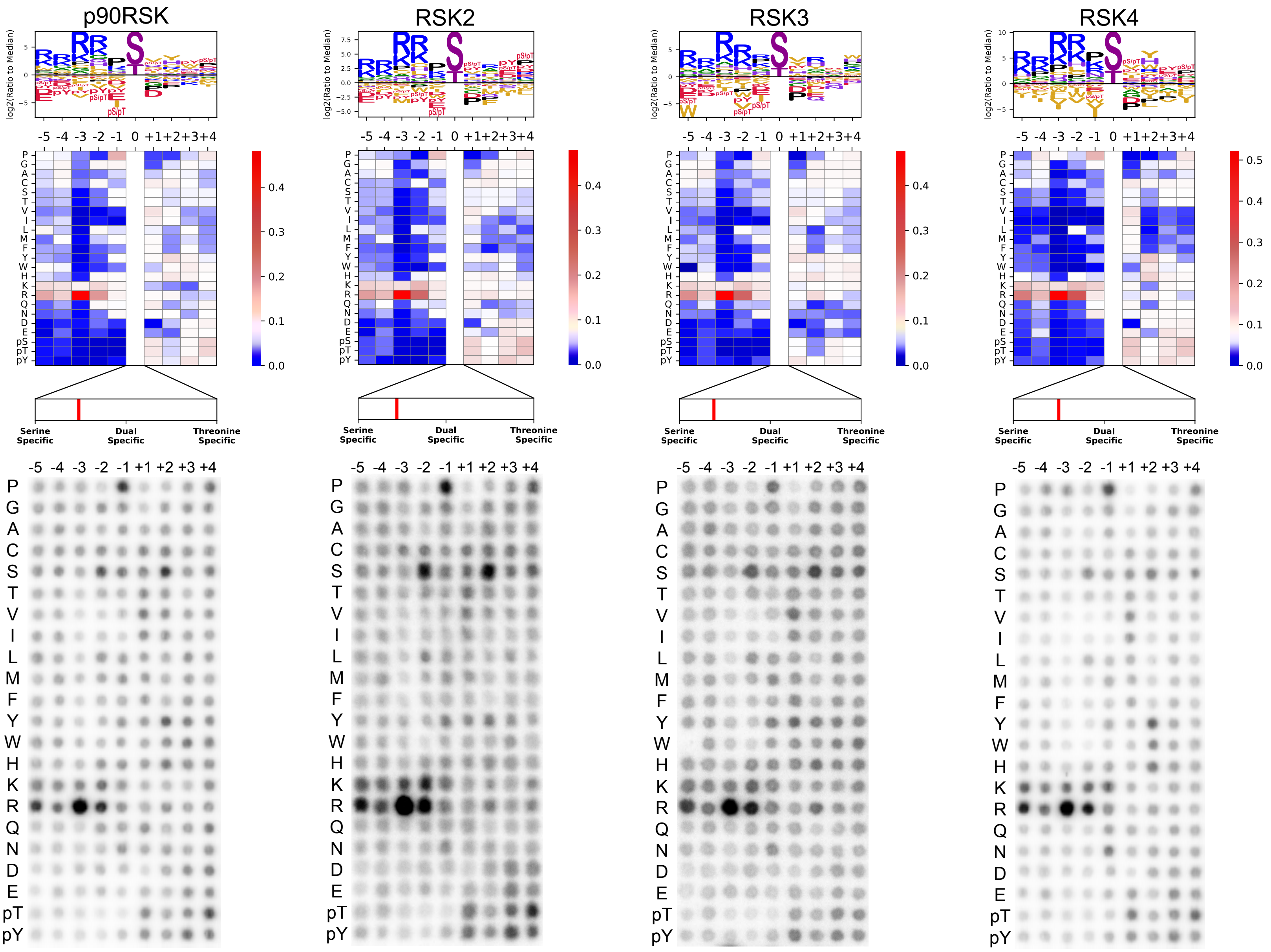

p90RSK
RSK2
RSK3
RSK4
log2(Ratio to Median)
-5 -4 -3 -2 -1 0 +1 +2 +3 +4
P G A C S T V I L M F Y W H K R Q N D E pS pT pY
Serine Specific
Dual Specific
Threonine Specific
-5 -4 -3 -2 -1 +1 +2 +3 +4
P G A C S T V I L M F Y W H K R Q N D E pT pY

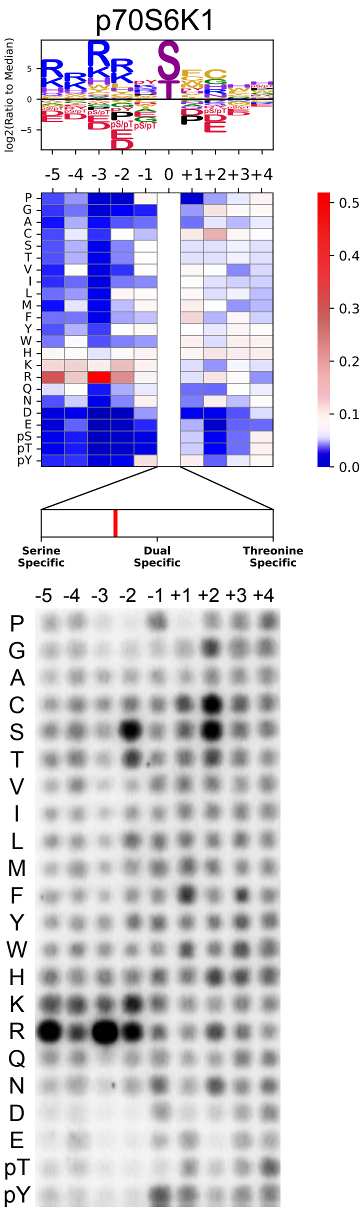
p70S6K1
log2(Ratio to Median)
-5 -4 -3 -2 -1 0 +1 +2 +3 +4
P G A C S T V I L M F Y W H K R Q N D E pS pT pY
0.5 0.4 0.3 0.2 0.1 0.0
Serine Specific
Dual Specific
Threonine Specific
-5 -4 -3 -2 -1 +1 +2 +3 +4
P G A C S T V I L M F Y W H K R Q N D E pT pY

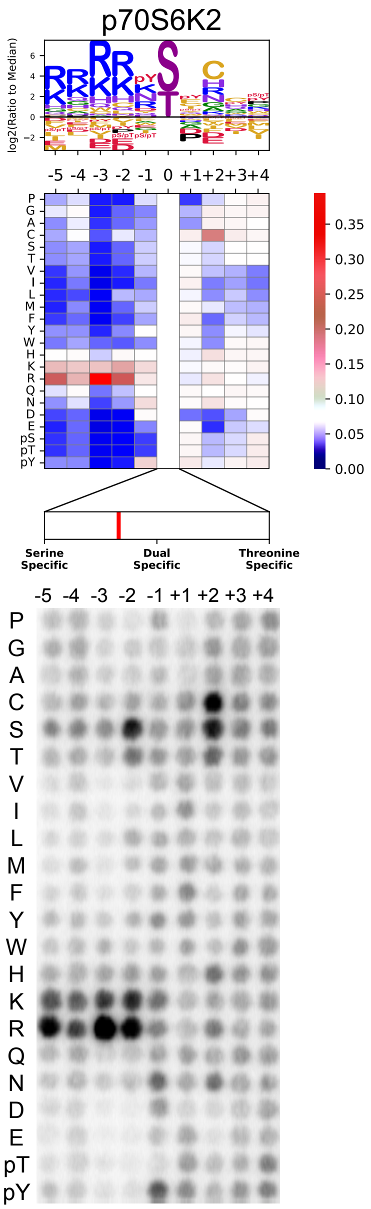
p70S6K2
log2(Ratio to Median)
-5 -4 -3 -2 -1 0 +1 +2 +3 +4
P G A C S T V I L M F Y W H K R Q N D E pS pT pY
0.35 0.30 0.25 0.20 0.15 0.10 0.05 0.00
Serine Specific
Dual Specific
Threonine Specific
-5 -4 -3 -2 -1 +1 +2 +3 +4
P G A C S T V I L M F Y W H K R Q N D E pT pY

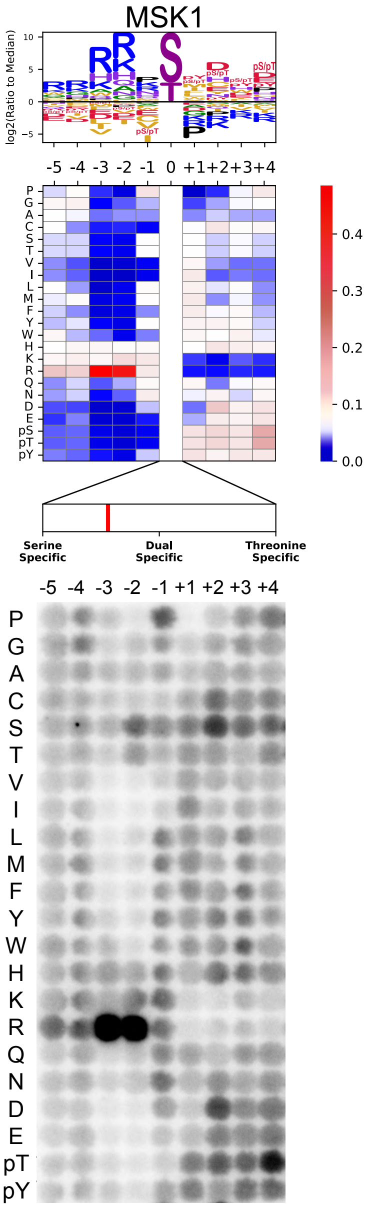
MSK1
log2(Ratio to Median)
-5 -4 -3 -2 -1 0 +1 +2 +3 +4
P G A C S T V I L M F Y W H K R Q N D E pS pT pY
0.0 0.1 0.2 0.3 0.4
Serine Specific
Dual Specific
Threonine Specific
-5 -4 -3 -2 -1 +1 +2 +3 +4
P G A C S T V I L M F Y W H K R Q N D E pT pY

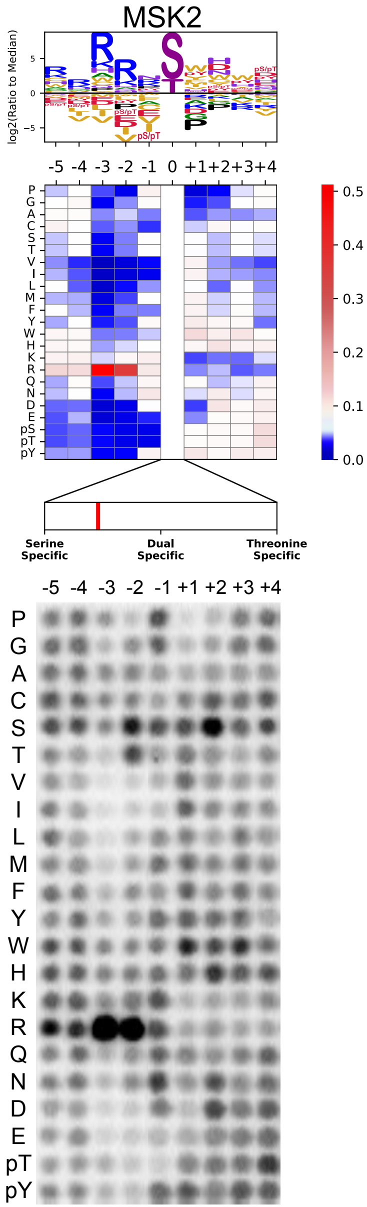
MSK2
log2(Ratio to Median)
-5 -4 -3 -2 -1 0 +1 +2 +3 +4
P G A C S T V I L M F Y W H K R Q N D E pS pT pY
0.0 0.1 0.2 0.3 0.4 0.5
Serine Specific
Dual Specific
Threonine Specific
-5 -4 -3 -2 -1 +1 +2 +3 +4
P G A C S T V I L M F Y W H K R Q N D E pT pY

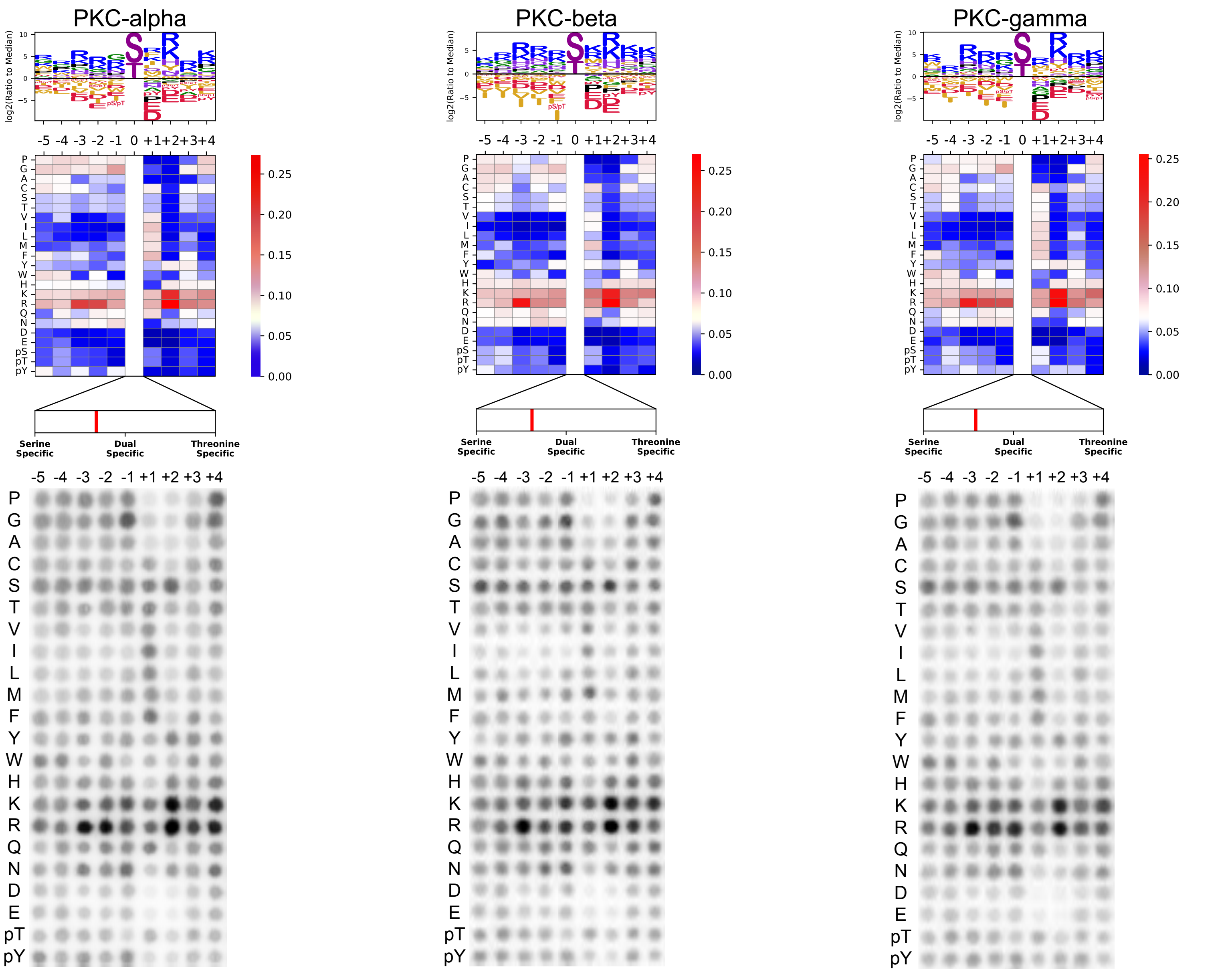

PKC-alpha
PKC-beta
PKC-gamma
log2(Ratio to Median)
-5 -4 -3 -2 -1 0 +1 +2 +3 +4
P G A C S T V I L M F Y W H K R Q N D E pS pT pY
0.00
0.05
0.10
0.15
0.20
0.25
Serine Specific
Dual Specific
Threonine Specific
-5 -4 -3 -2 -1 +1 +2 +3 +4
P G A C S T V I L M F Y W H K R Q N D E pT pY

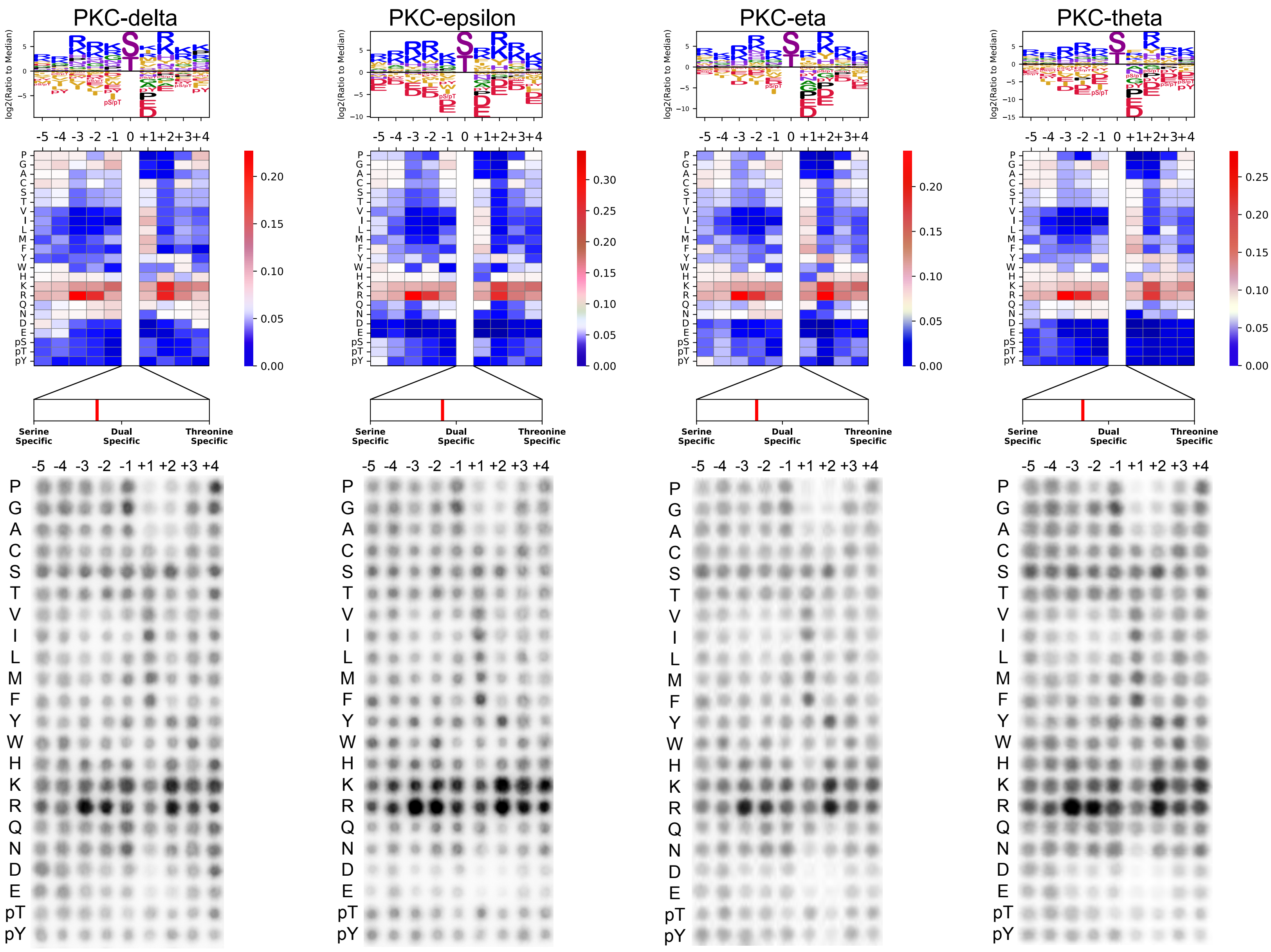
PKC-delta
PKC-epsilon
PKC-eta
PKC-theta
log2(Ratio to Median)
-5 -4 -3 -2 -1 0 +1 +2 +3 +4
P G A C S T V I L M F Y W H K R Q N D E pS pT pY
Serine Specific
Dual Specific
Threonine Specific
-5 -4 -3 -2 -1 +1 +2 +3 +4
P G A C S T V I L M F Y W H K R Q N D E pT pY

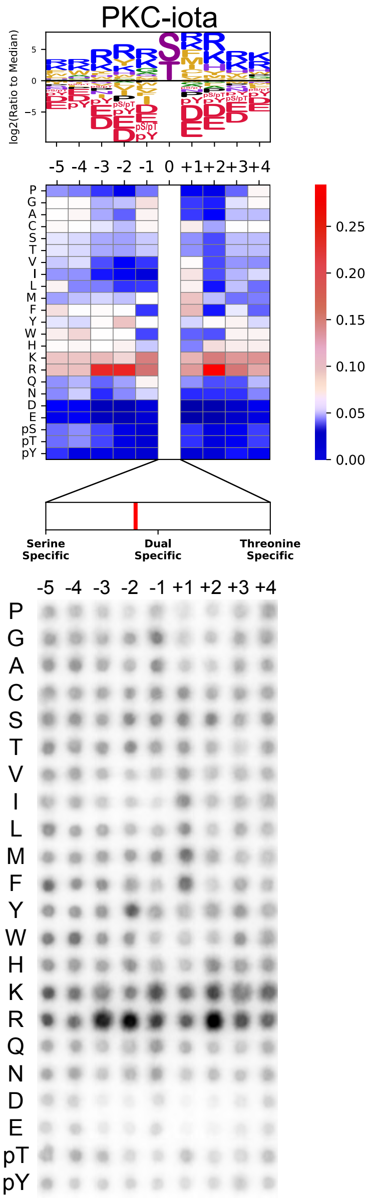
PKC-iota
log2(Ratio to Median)
5
0
−5
-5 -4 -3 -2 -1 0 +1 +2 +3 +4
P G A C S T V I L M F Y W H K R Q N D E pS pT pY
0.25
0.20
0.15
0.10
0.05
0.00
Serine Specific
Dual Specific
Threonine Specific
-5 -4 -3 -2 -1 +1 +2 +3 +4
P G A C S T V I L M F Y W H K R Q N D E pT pY

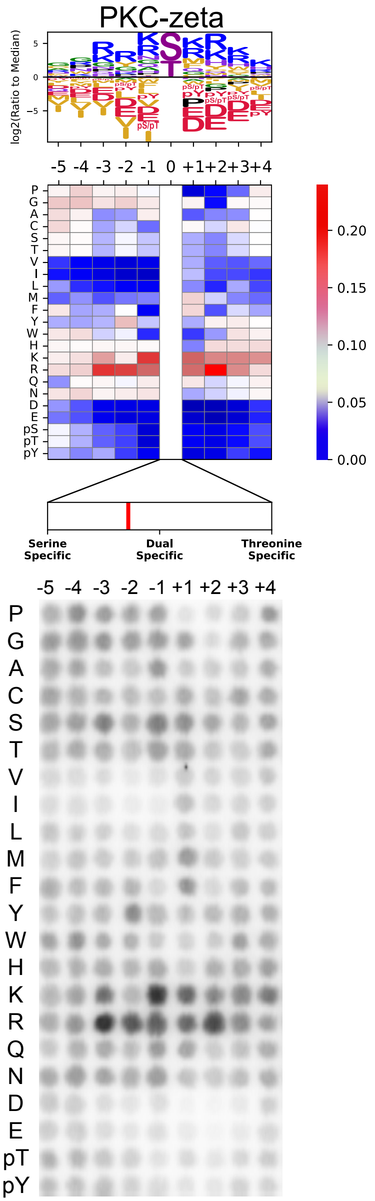
PKC-zeta
log2(Ratio to Median)
5
0
−5
-5 -4 -3 -2 -1 0 +1 +2 +3 +4
P G A C S T V I L M F Y W H K R Q N D E pS pT pY
0.20
0.15
0.10
0.05
0.00
Serine Specific
Dual Specific
Threonine Specific
-5 -4 -3 -2 -1 +1 +2 +3 +4
P G A C S T V I L M F Y W H K R Q N D E pT pY

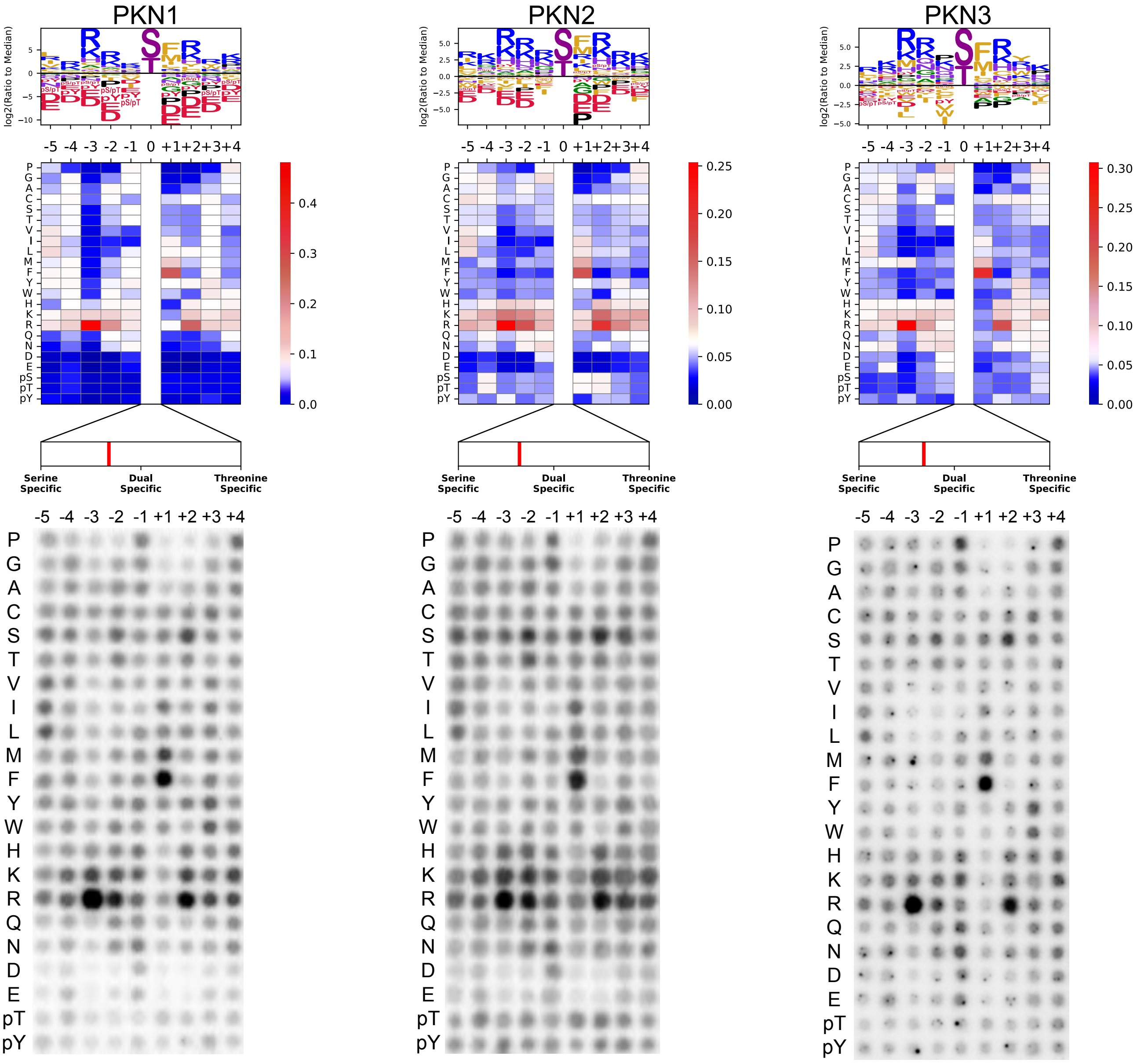

PKN1
PKN2
PKN3
log2(Ratio to Median)
-5 -4 -3 -2 -1 0 +1 +2 +3 +4
P G A C S T V I L M F Y W H K R Q N D E pS pT pY
Serine Specific
Dual Specific
Threonine Specific
-5 -4 -3 -2 -1 +1 +2 +3 +4
P G A C S T V I L M F Y W H K R Q N D E pT pY

CAMK

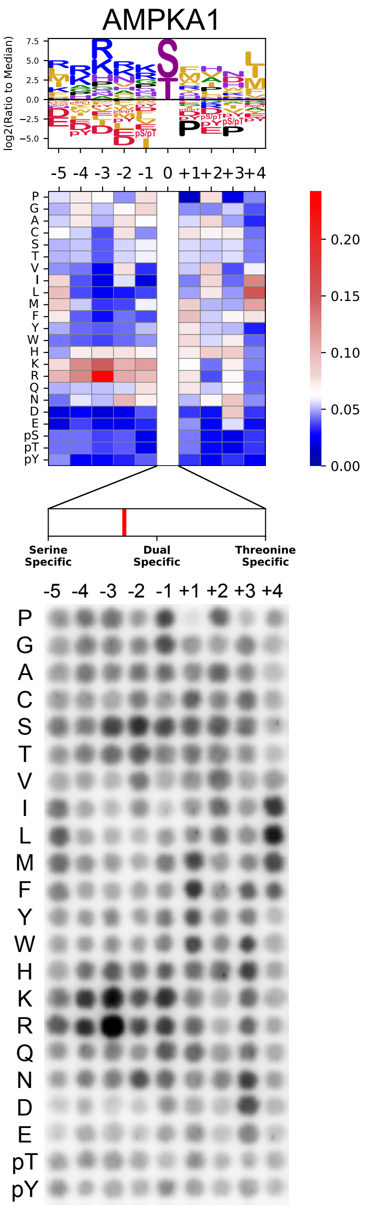
AMPKA1
log2(Ratio to Median)
-5 -4 -3 -2 -1 0 +1 +2 +3 +4
P G A C S T V I L M F Y W H K R Q N D E pS pT pY
Serine Specific
Dual Specific
Threonine Specific
-5 -4 -3 -2 -1 +1 +2 +3 +4
P G A C S T V I L M F Y W H K R Q N D E pT pY

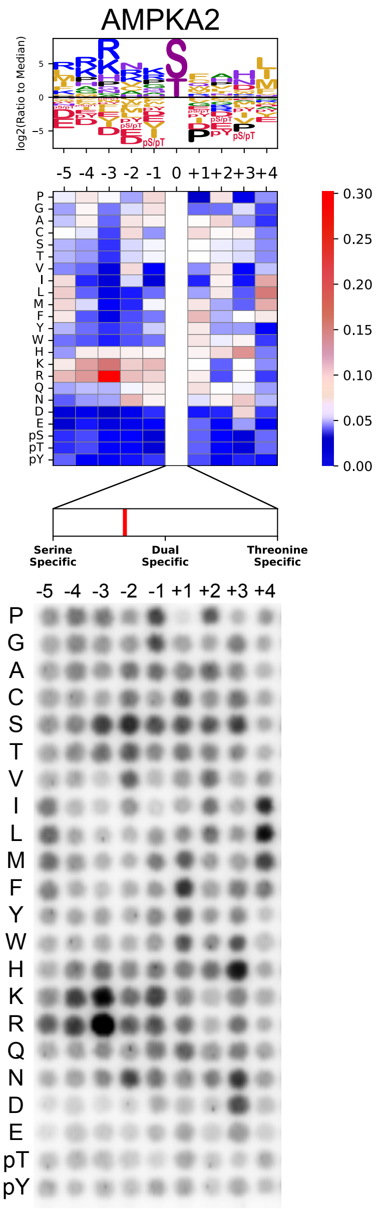
AMPKA2
log2(Ratio to Median)
-5 -4 -3 -2 -1 0 +1 +2 +3 +4
P G A C S T V I L M F Y W H K R Q N D E pS pT pY
Serine Specific
Dual Specific
Threonine Specific
-5 -4 -3 -2 -1 +1 +2 +3 +4
P G A C S T V I L M F Y W H K R Q N D E pT pY

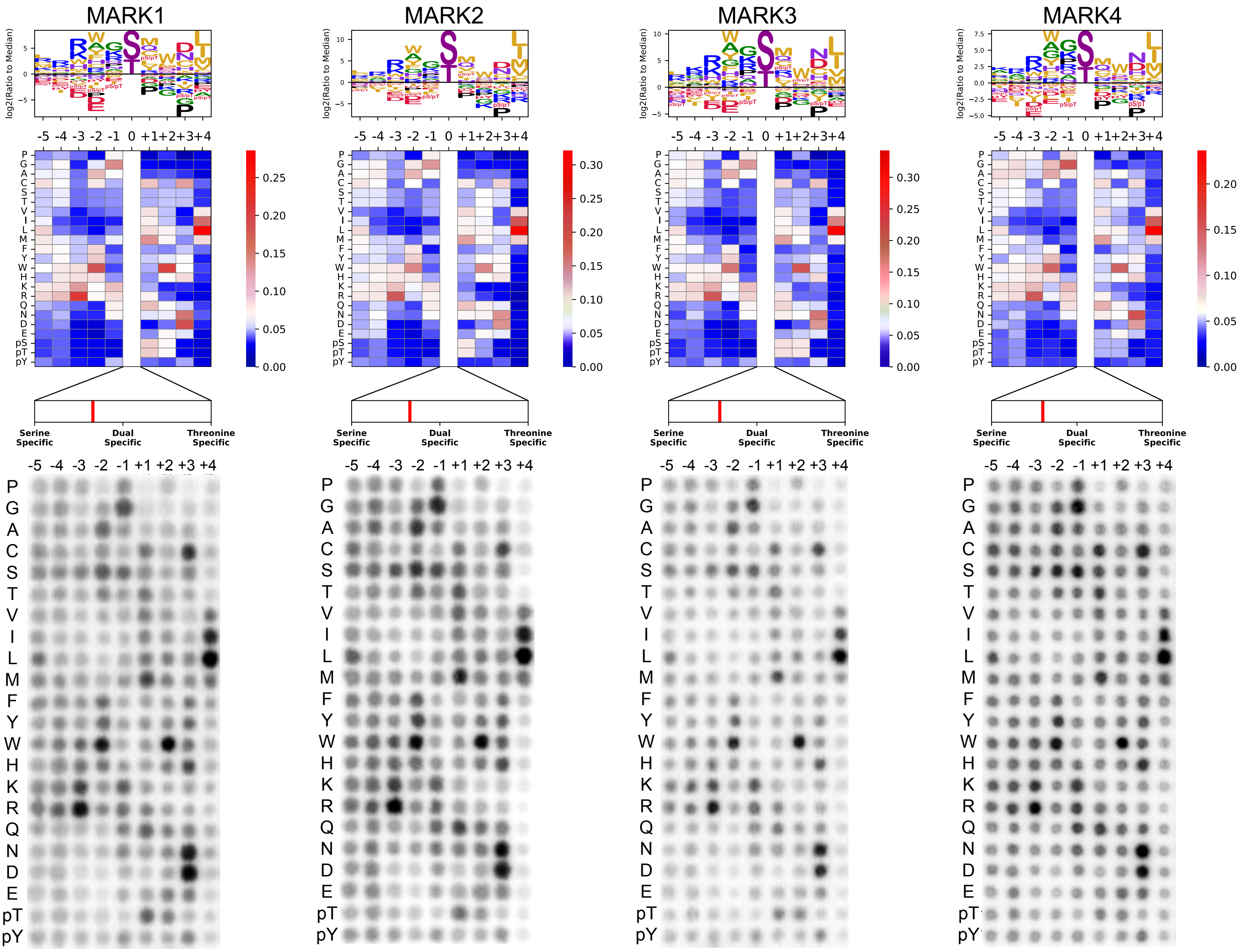
MARK1
MARK2
MARK3
MARK4
log2(Ratio to Median)
-5 -4 -3 -2 -1 0 +1 +2 +3 +4
P G A C S T V I L M F Y W H K R Q N D E pS pT pY
Serine Specific
Dual Specific
Threonine Specific
-5 -4 -3 -2 -1 +1 +2 +3 +4
P G A C S T V I L M F Y W H K R Q N D E pT pY

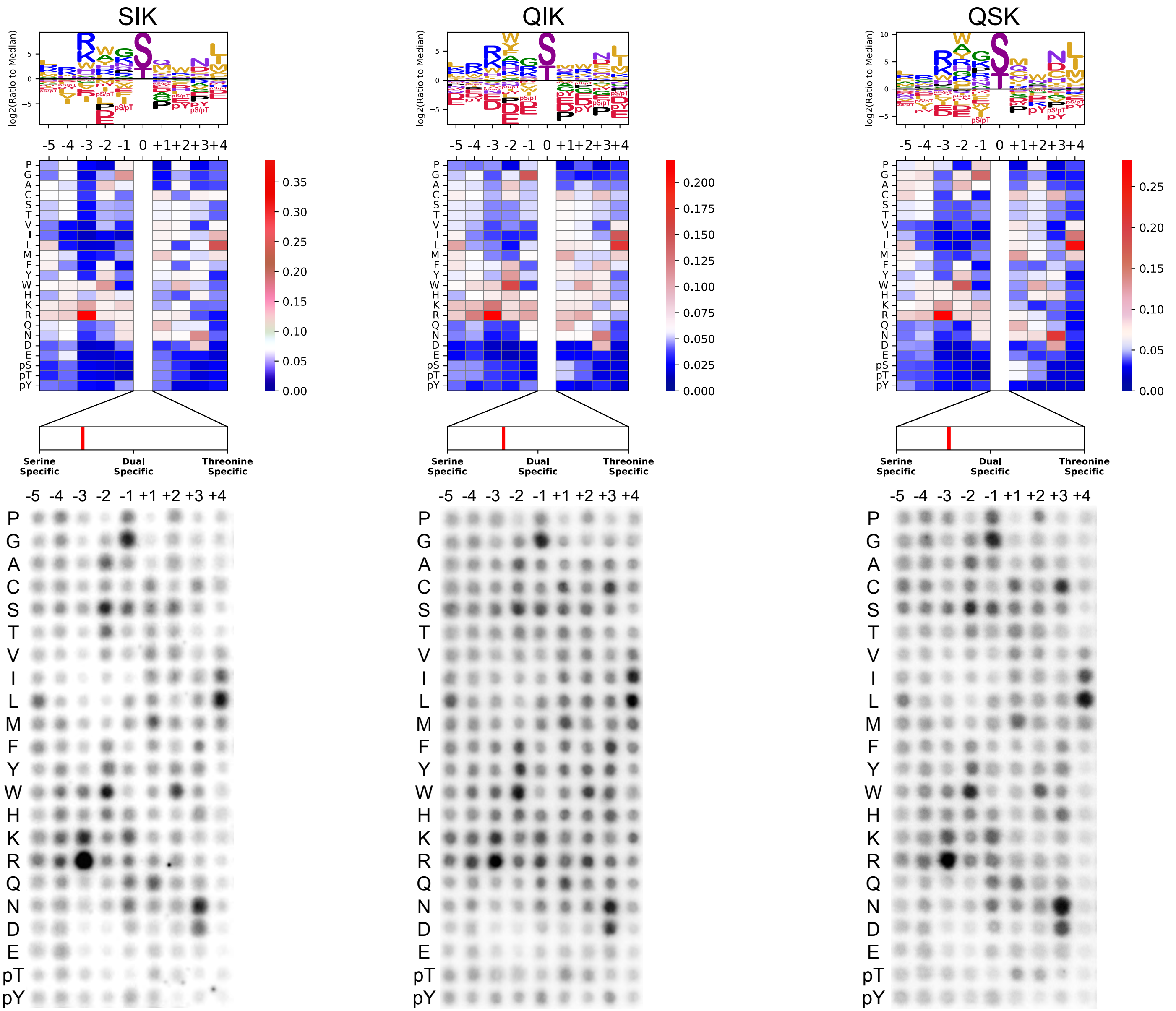
SIK
QIK
QSK
log2(Ratio to Median)
-5 -4 -3 -2 -1 0 +1 +2 +3 +4
P G A C S T V I L M F Y W H K R Q N D E pS pT pY
0.35 0.30 0.25 0.20 0.15 0.10 0.05 0.00
0.200 0.175 0.150 0.125 0.100 0.075 0.050 0.025 0.000
0.25 0.20 0.15 0.10 0.05 0.00
Serine Specific
Dual Specific
Threonine Specific
-5 -4 -3 -2 -1 +1 +2 +3 +4
P G A C S T V I L M F Y W H K R Q N D E pT pY

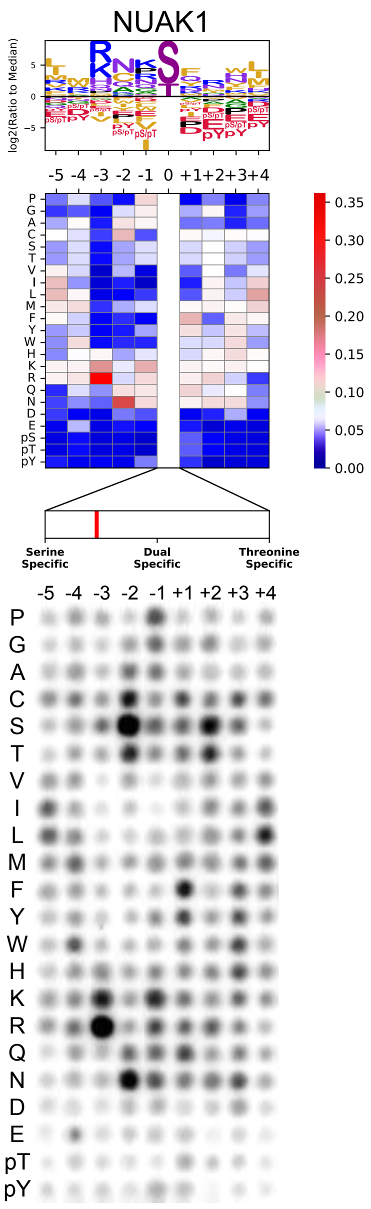
NUAK1
log2(Ratio to Median)
5
0
-5
-5 -4 -3 -2 -1 0 +1 +2 +3 +4
P G A C S T V I L M F Y W H K R Q N D E pS pT pY
0.35
0.30
0.25
0.20
0.15
0.10
0.05
0.00
Serine Specific
Dual Specific
Threonine Specific
-5 -4 -3 -2 -1 +1 +2 +3 +4
P G A C S T V I L M F Y W H K R Q N D E pT pY

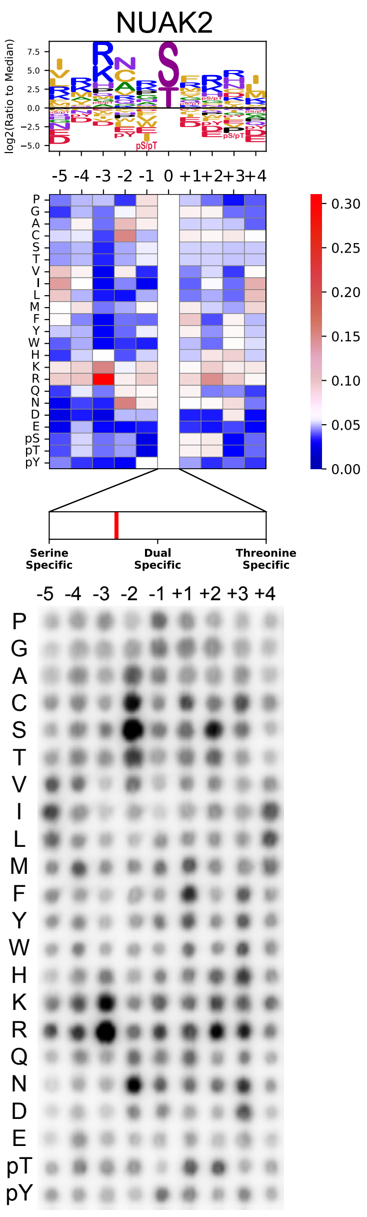
NUAK2
log2(Ratio to Median)
7.5
5.0
2.5
0.0
-2.5
-5.0
-5 -4 -3 -2 -1 0 +1 +2 +3 +4
P G A C S T V I L M F Y W H K R Q N D E pS pT pY
0.30
0.25
0.20
0.15
0.10
0.05
0.00
Serine Specific
Dual Specific
Threonine Specific
-5 -4 -3 -2 -1 +1 +2 +3 +4
P G A C S T V I L M F Y W H K R Q N D E pT pY

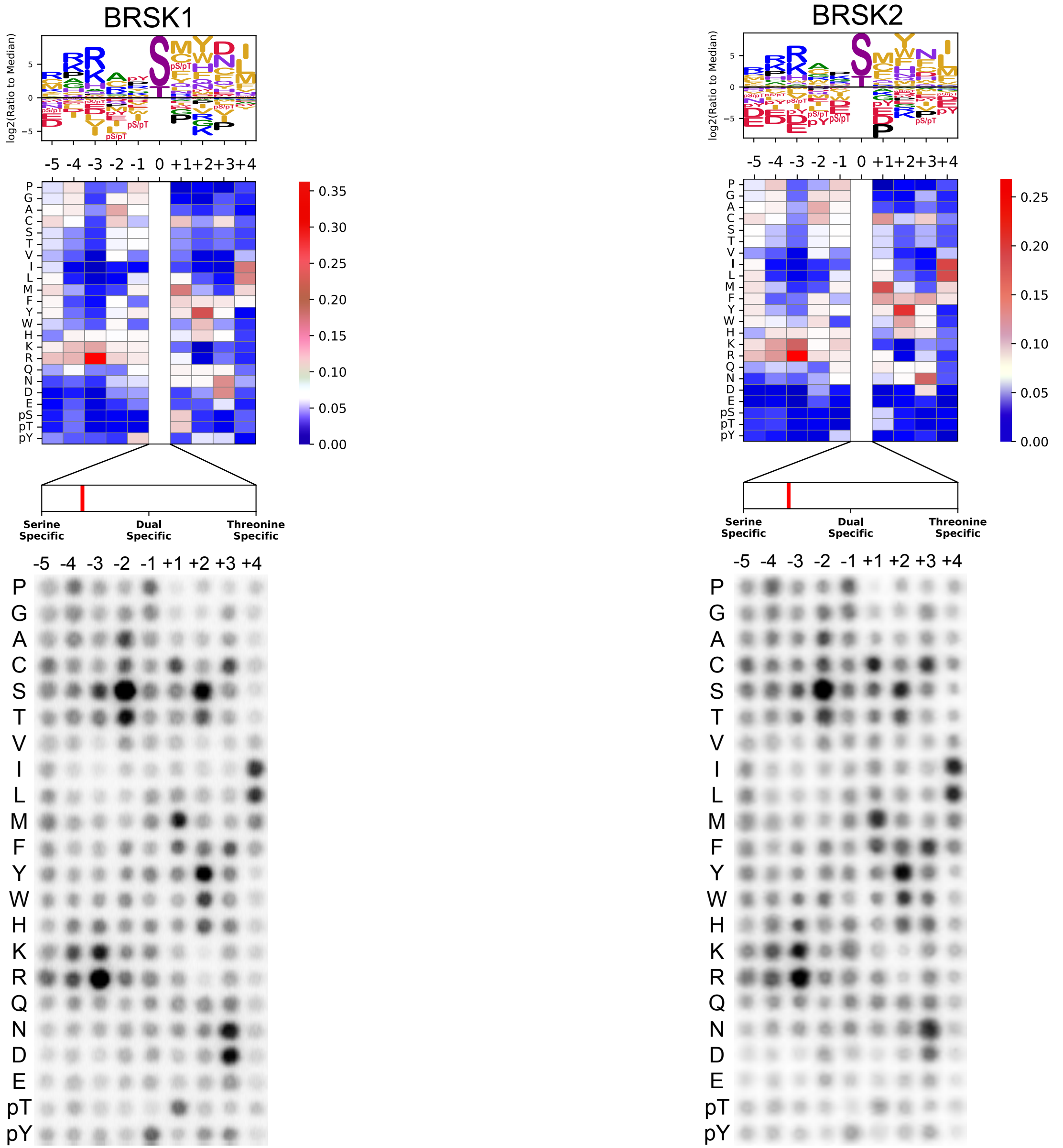

BRSK1
BRSK2
log2(Ratio to Median)
-5 -4 -3 -2 -1 0 +1 +2 +3 +4
P G A C S T V I L M F Y W H K R Q N D E pS pT pY
0.35 0.30 0.25 0.20 0.15 0.10 0.05 0.00
0.25 0.20 0.15 0.10 0.05 0.00
Serine Specific
Dual Specific
Threonine Specific
P G A C S T V I L M F Y W H K R Q N D E pT pY

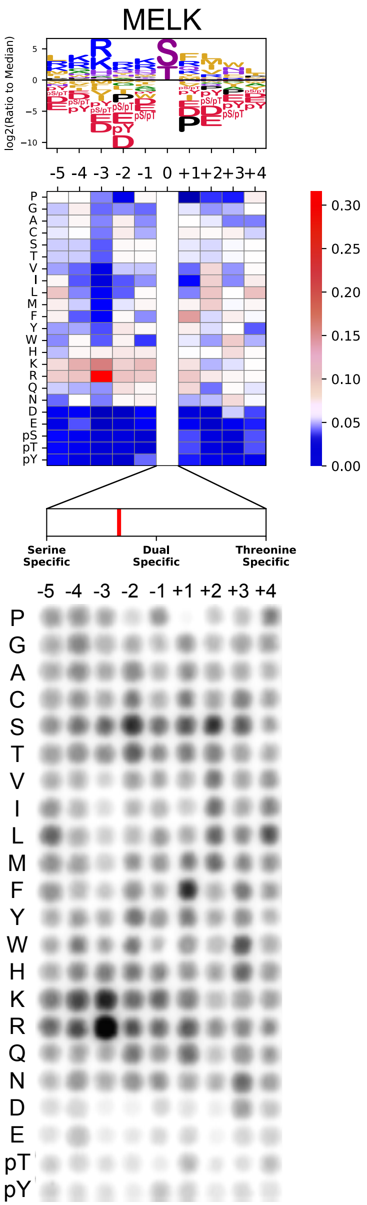
MELK
log2(Ratio to Median)
-5 -4 -3 -2 -1 0 +1 +2 +3 +4
P G A C S T V I L M F Y W H K R Q N D E pS pT pY
0.30 0.25 0.20 0.15 0.10 0.05 0.00
Serine Specific
Dual Specific
Threonine Specific
-5 -4 -3 -2 -1 +1 +2 +3 +4
P G A C S T V I L M F Y W H K R Q N D E pT pY

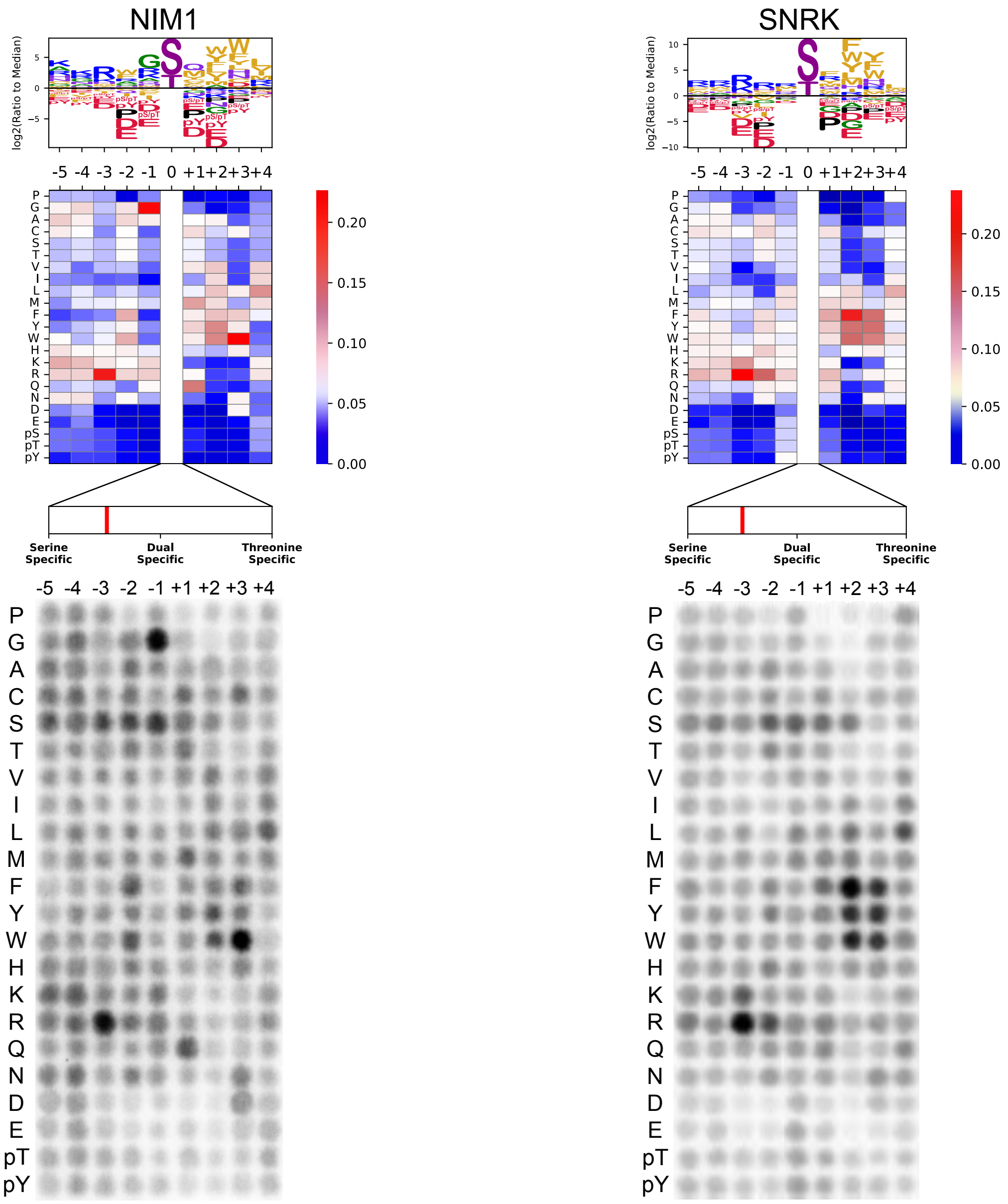

NIM1
SNRK
log2(Ratio to Median)
-5 -4 -3 -2 -1 0 +1 +2 +3 +4
P G A C S T V I L M F Y W H K R Q N D E pS pT pY
0.00 0.05 0.10 0.15 0.20
Serine Specific
Dual Specific
Threonine Specific
-5 -4 -3 -2 -1 +1 +2 +3 +4
P G A C S T V I L M F Y W H K R Q N D E pT pY

HUNK

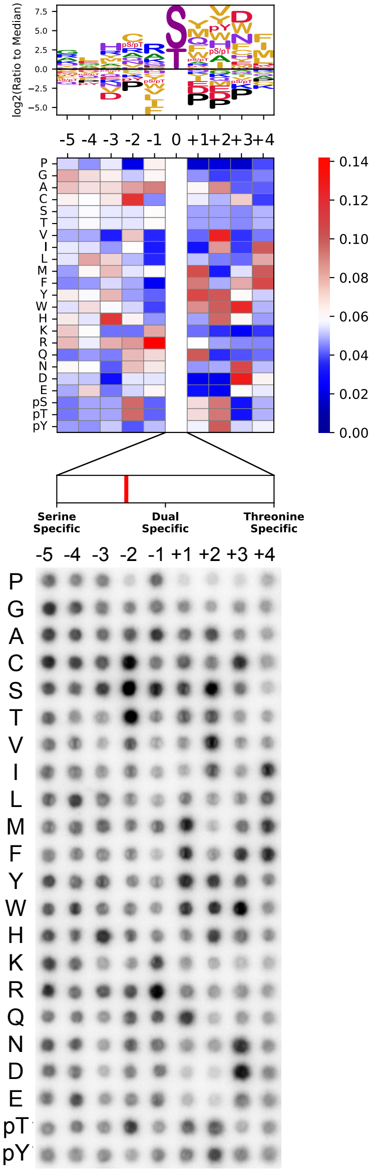
log2(Ratio to Median)
7.5
5.0
2.5
0.0
−2.5
−5.0
-5 -4 -3 -2 -1 0 +1 +2 +3 +4
P
G
A
C
S
T
V
I
L
M
F
Y
W
H
K
R
Q
N
D
E
pS
pT
pY
0.14
0.12
0.10
0.08
0.06
0.04
0.02
0.00
Serine Specific
Dual Specific
Threonine Specific
-5 -4 -3 -2 -1 +1 +2 +3 +4
P
G
A
C
S
T
V
I
L
M
F
Y
W
H
K
R
Q
N
D
E
pT
pY

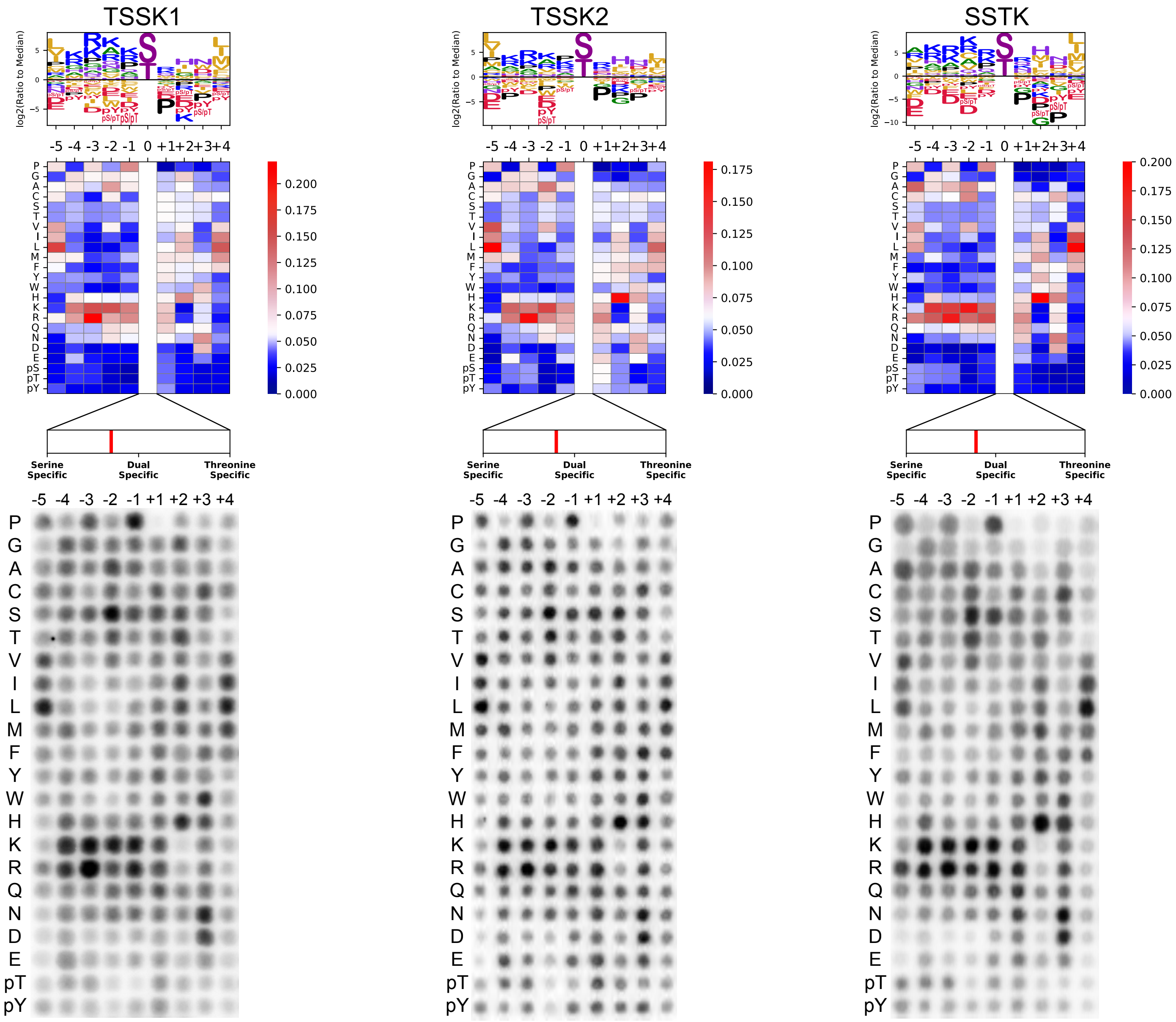
TSSK1
TSSK2
SSTK
log2(Ratio to Median)
-5 -4 -3 -2 -1 0 +1 +2 +3 +4
P G A C S T V I L M F Y W H K R Q N D E pS pT pY
0.000 0.025 0.050 0.075 0.100 0.125 0.150 0.175 0.200
Serine Specific
Dual Specific
Threonine Specific
-5 -4 -3 -2 -1 +1 +2 +3 +4
P G A C S T V I L M F Y W H K R Q N D E pT pY

STK33

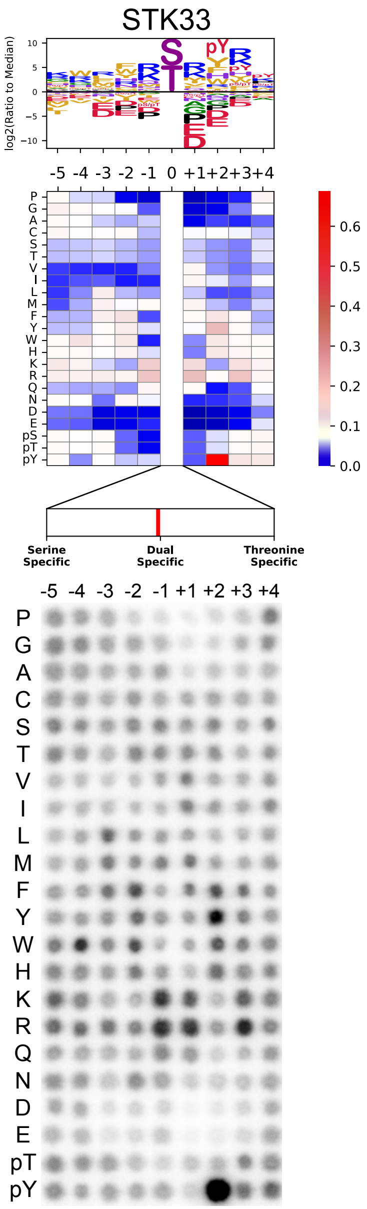
STK33
log2(Ratio to Median)
-5 -4 -3 -2 -1 0 +1 +2 +3 +4
P G A C S T V I L M F Y W H K R Q N D E pS pT pY
0.6 0.5 0.4 0.3 0.2 0.1 0.0
Serine Specific
Dual Specific
Threonine Specific
-5 -4 -3 -2 -1 +1 +2 +3 +4
P G A C S T V I L M F Y W H K R Q N D E pT pY

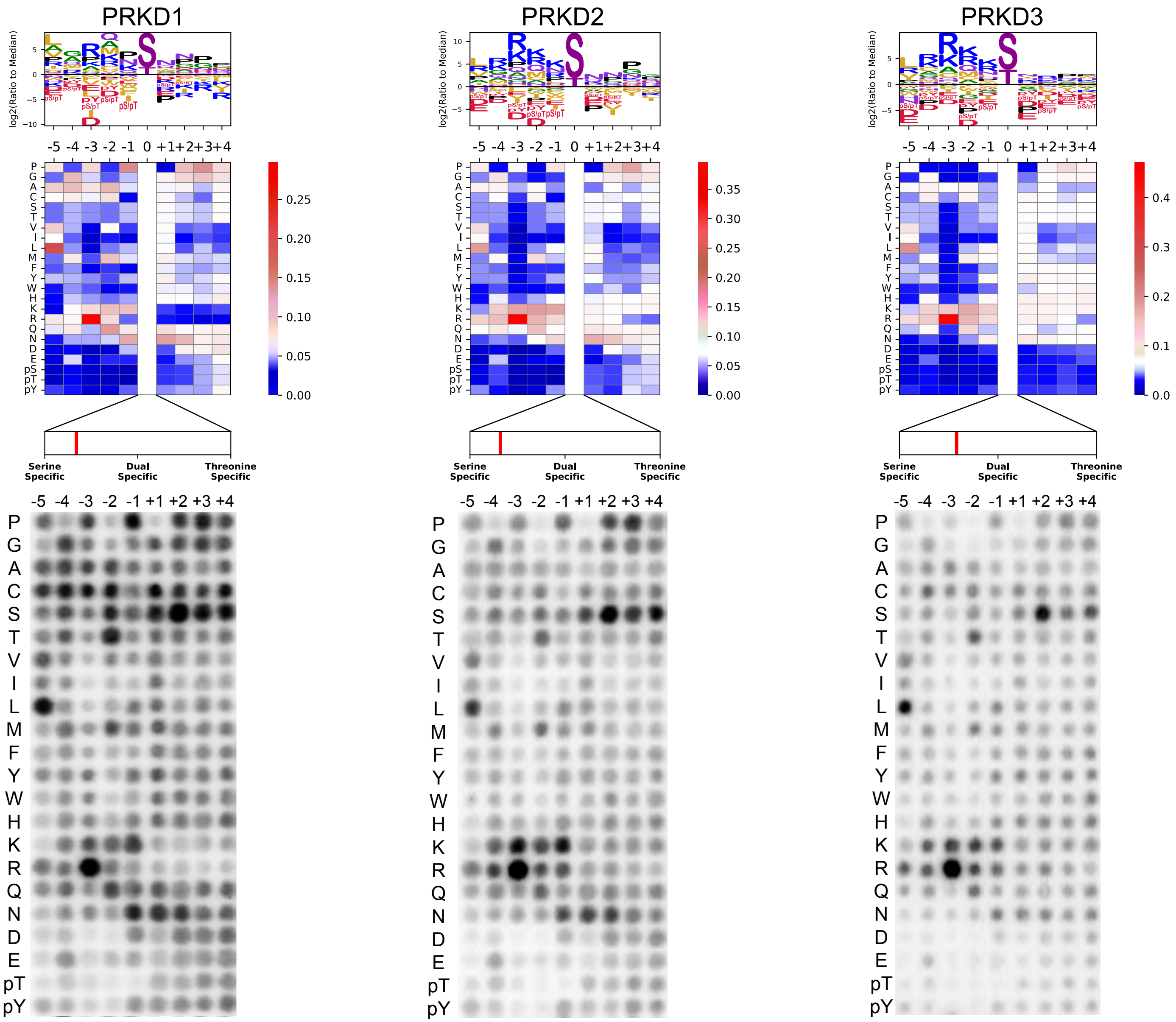
PRKD1
PRKD2
PRKD3
log2(Ratio to Median)
-5 -4 -3 -2 -1 0 +1 +2 +3 +4
P G A C S T V I L M F Y W H K R Q N D E pS pT pY
Serine Specific
Dual Specific
Threonine Specific
-5 -4 -3 -2 -1 +1 +2 +3 +4
P G A C S T V I L M F Y W H K R Q N D E pT pY

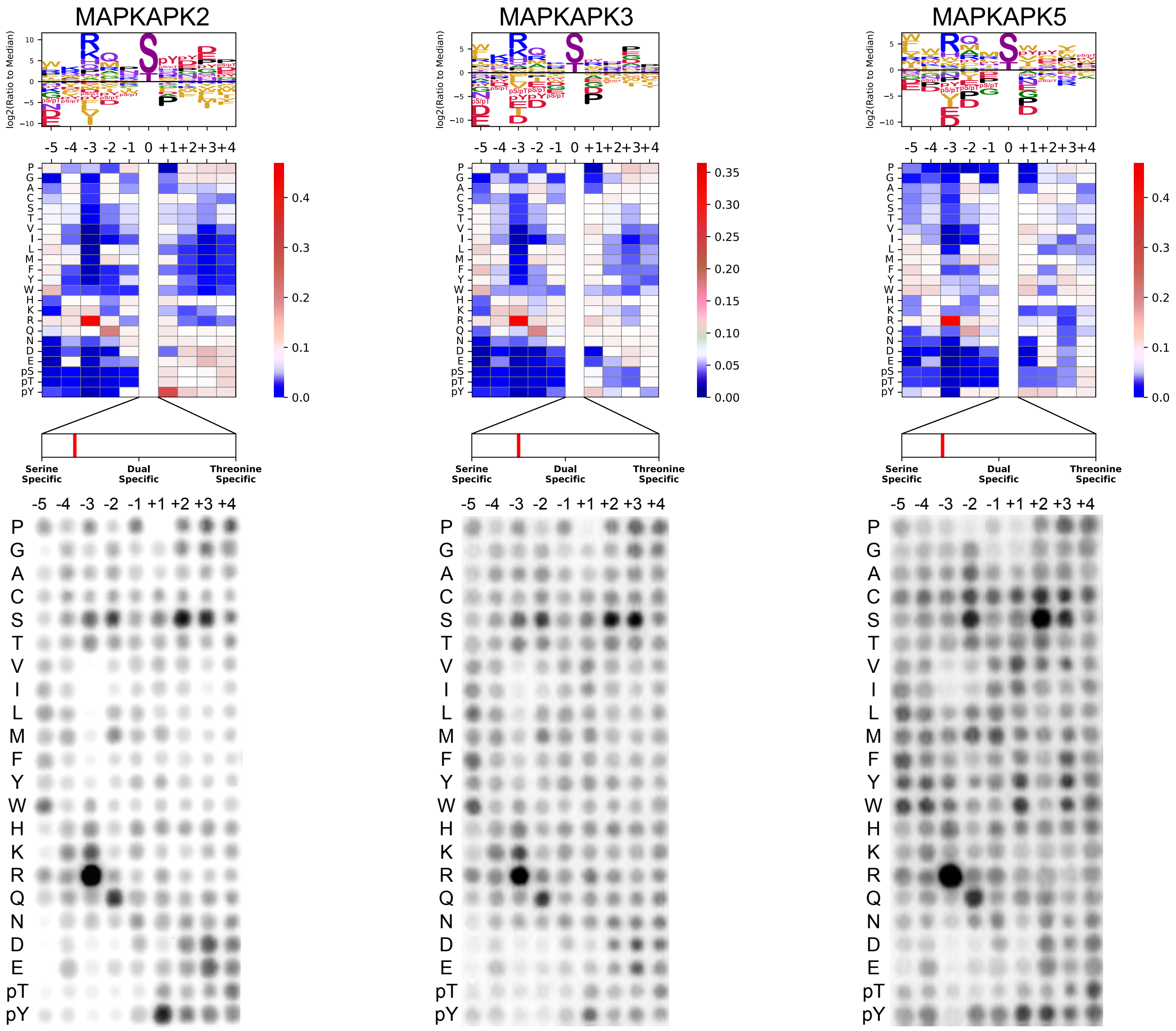
MAPKAPK2
MAPKAPK3
MAPKAPK5
log2(Ratio to Median)
-5 -4 -3 -2 -1 0 +1 +2 +3 +4
P G A C S T V I L M F Y W H K R Q N D E pS pT pY
Serine Specific
Dual Specific
Threonine Specific
-5 -4 -3 -2 -1 +1 +2 +3 +4
P G A C S T V I L M F Y W H K R Q N D E pT pY

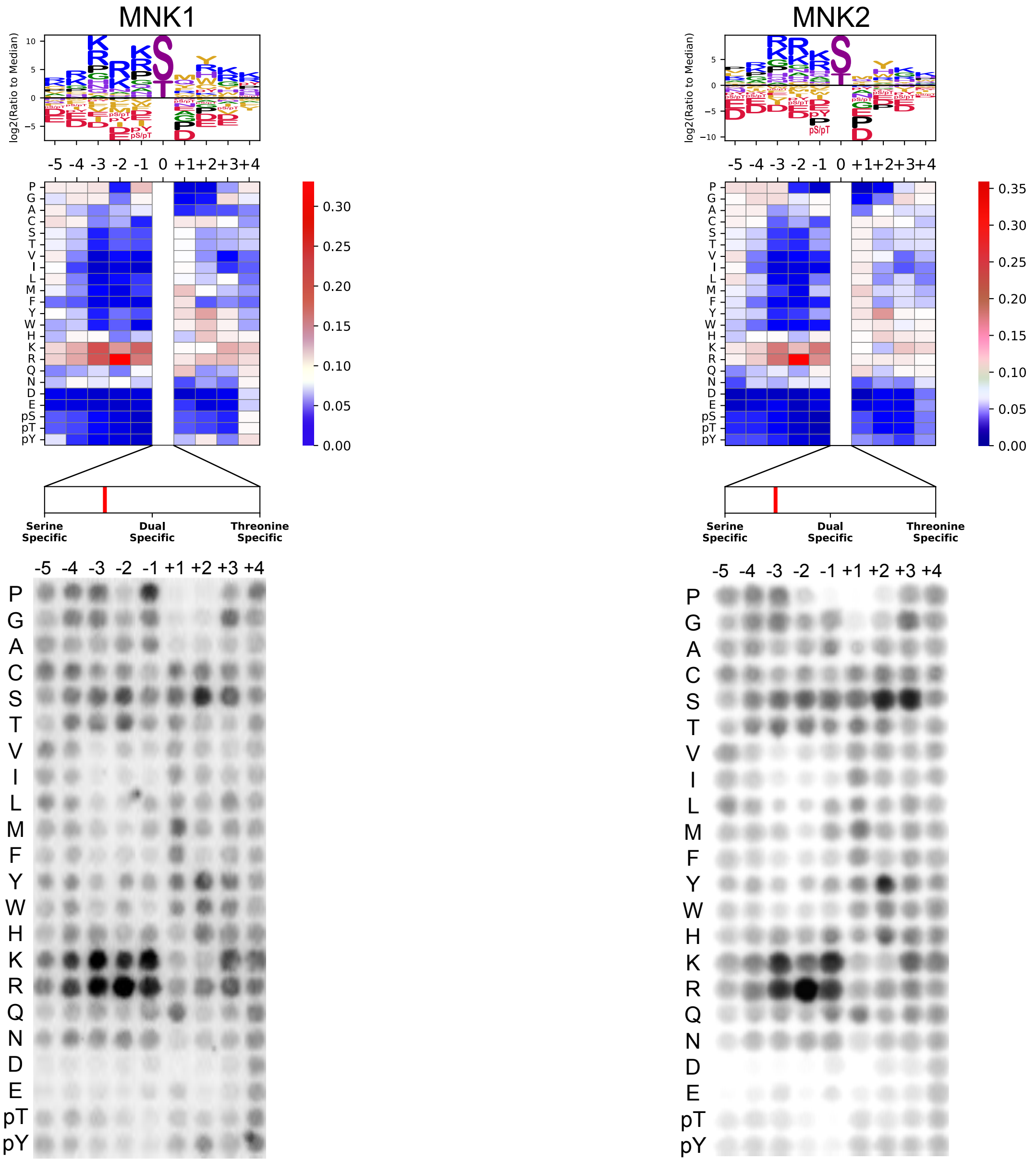
MNK1
MNK2
log2(Ratio to Median)
-5 -4 -3 -2 -1 0 +1 +2 +3 +4
P G A C S T V I L M F Y W H K R Q N D E pS pT pY
Serine Specific
Dual Specific
Threonine Specific
-5 -4 -3 -2 -1 +1 +2 +3 +4
P G A C S T V I L M F Y W H K R Q N D E pT pY

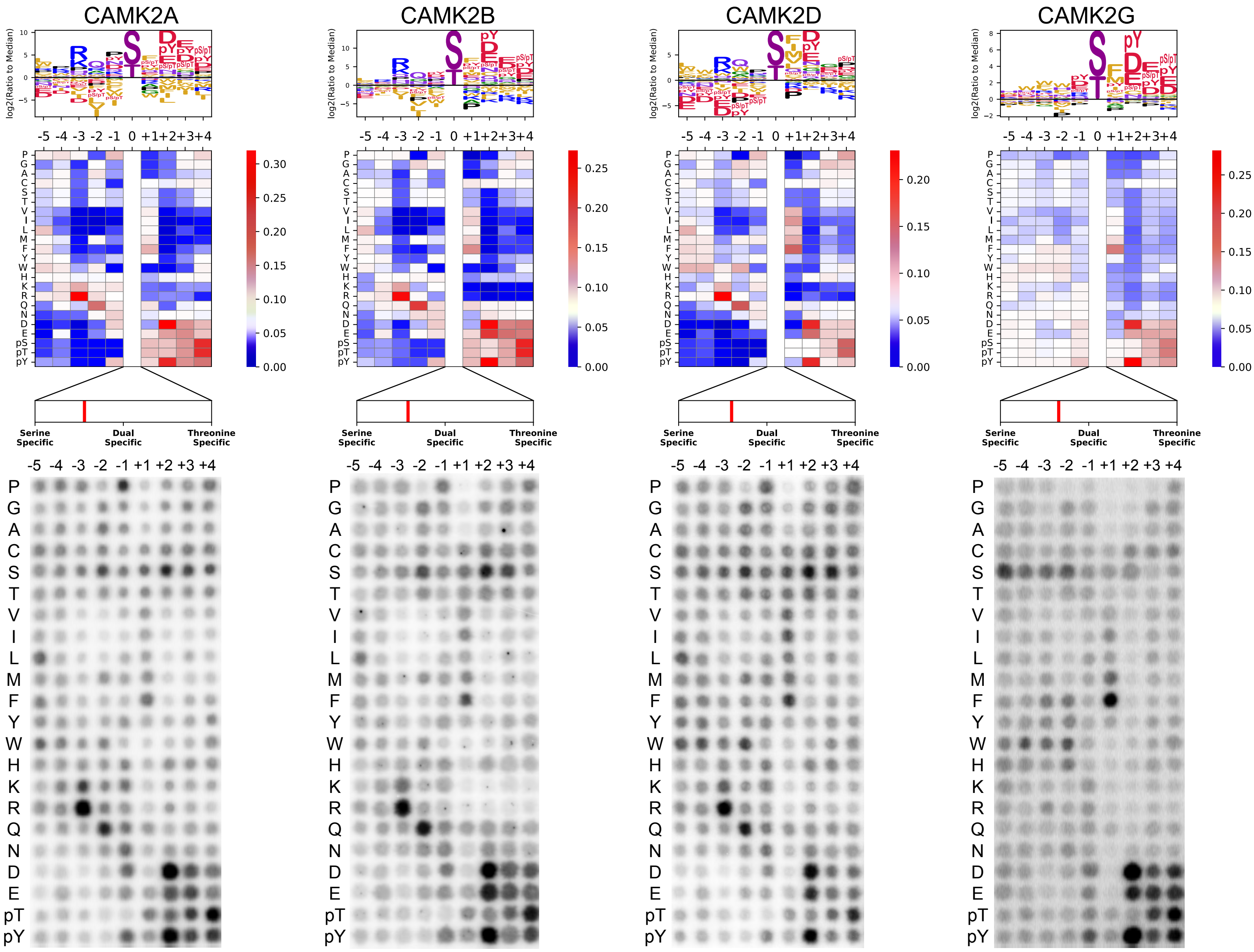

CAMK2A
CAMK2B
CAMK2D
CAMK2G
log2(Ratio to Median)
-5 -4 -3 -2 -1 0 +1 +2 +3 +4
P G A C S T V I L M F Y W H K R Q N D E pS pT pY
Serine Specific
Dual Specific
Threonine Specific
-5 -4 -3 -2 -1 +1 +2 +3 +4
P G A C S T V I L M F Y W H K R Q N D E pT pY

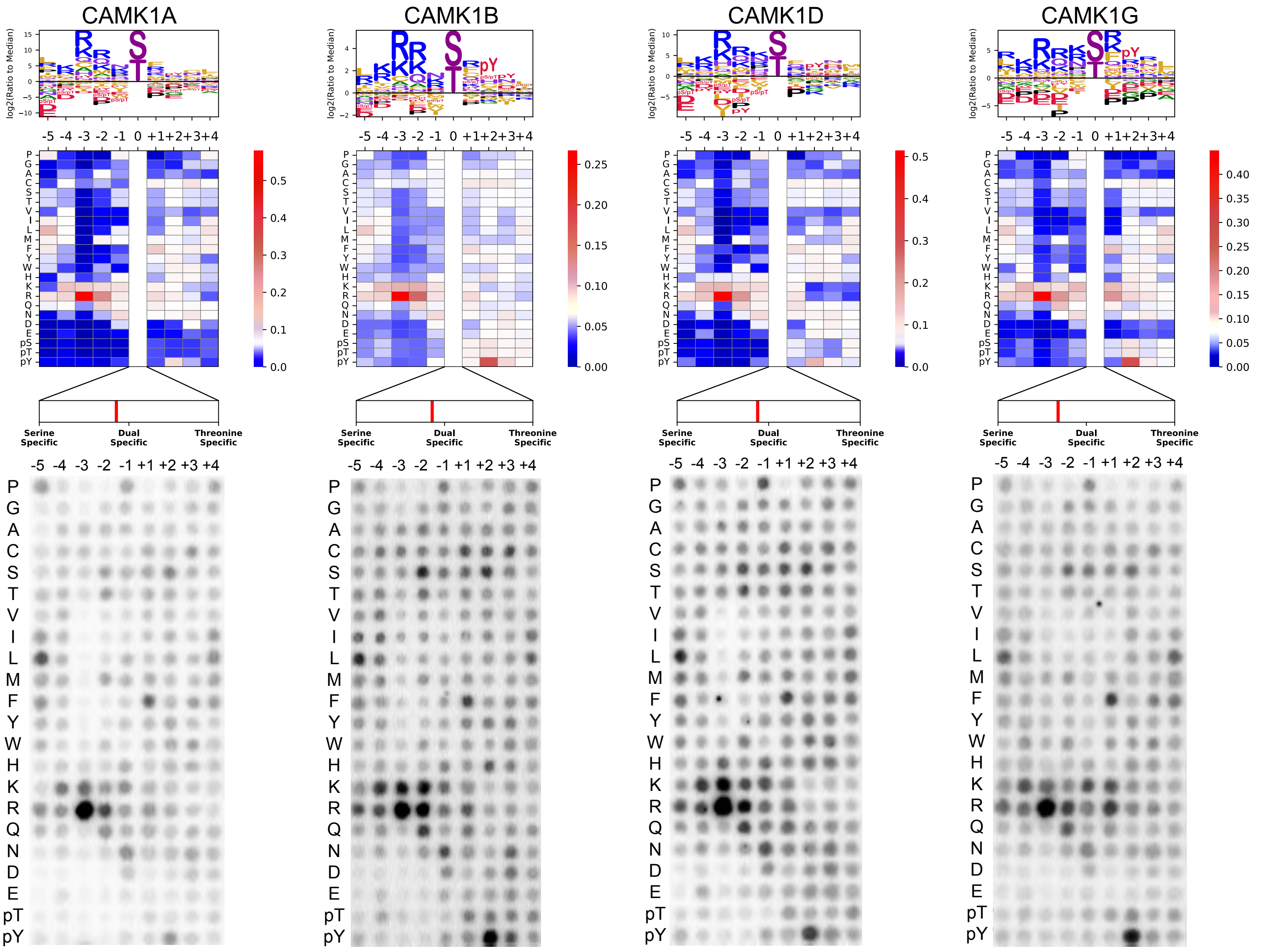
CAMK1A
CAMK1B
CAMK1D
CAMK1G
log2(Ratio to Median)
-5 -4 -3 -2 -1 0 +1 +2 +3 +4
P G A C S T V I L M F Y W H K R Q N D E pS pT pY
Serine Specific
Dual Specific
Threonine Specific
-5 -4 -3 -2 -1 +1 +2 +3 +4
P G A C S T V I L M F Y W H K R Q N D E pT pY

CAMK4

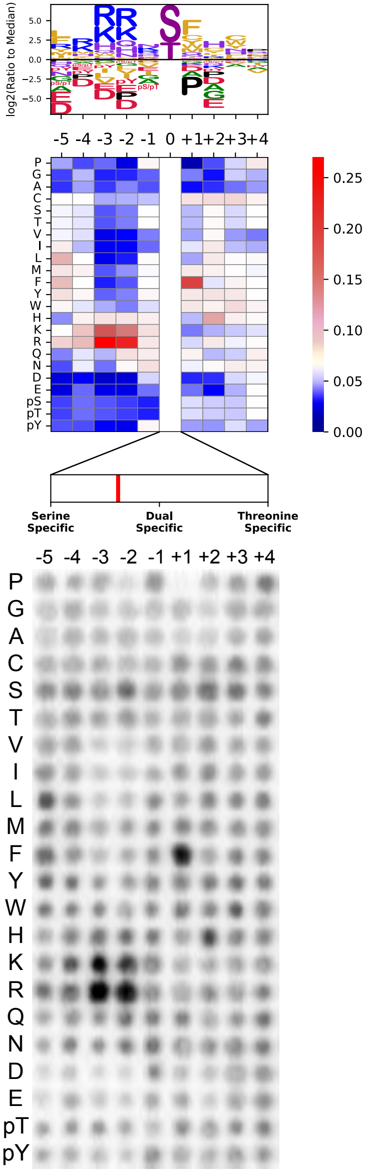
log2(Ratio to Median)
5.0
2.5
0.0
−2.5
−5.0
-5 -4 -3 -2 -1 0 +1+2+3+4
P G A C S T V I L M F Y W H K R Q N D E pS pT pY
0.25
0.20
0.15
0.10
0.05
0.00
Serine Specific
Dual Specific
Threonine Specific
-5 -4 -3 -2 -1 +1 +2 +3 +4
P G A C S T V I L M F Y W H K R Q N D E pT pY

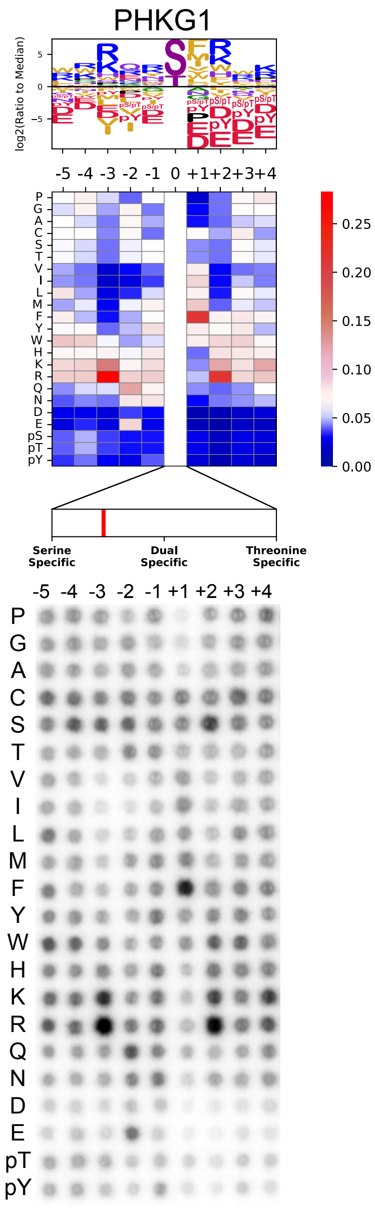
PHKG1
log2(Ratio to Median)
-5 -4 -3 -2 -1 0 +1 +2 +3 +4
P G A C S T V I L M F Y W H K R Q N D E pS pT pY
0.25
0.20
0.15
0.10
0.05
0.00
Serine Specific
Dual Specific
Threonine Specific
P G A C S T V I L M F Y W H K R Q N D E pT pY

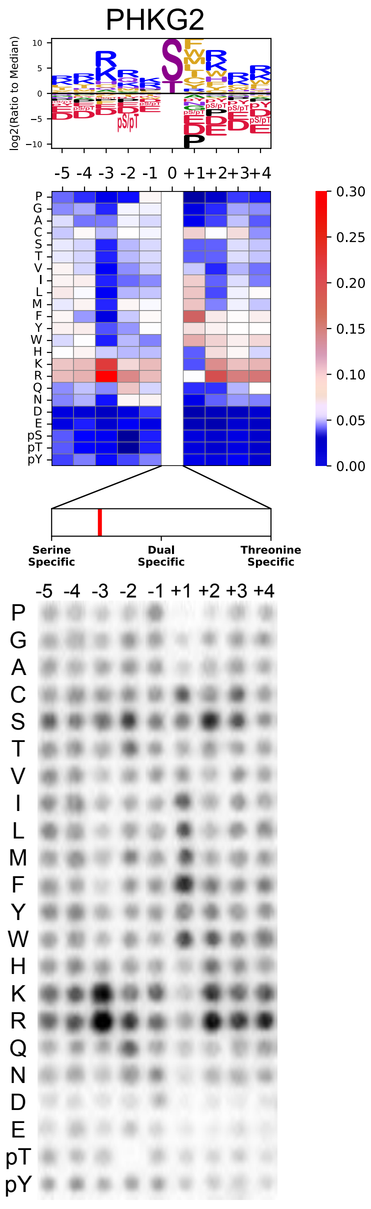
PHKG2
log2(Ratio to Median)
-5 -4 -3 -2 -1 0 +1 +2 +3 +4
P G A C S T V I L M F Y W H K R Q N D E pS pT pY
0.30
0.25
0.20
0.15
0.10
0.05
0.00
Serine Specific
Dual Specific
Threonine Specific
P G A C S T V I L M F Y W H K R Q N D E pT pY

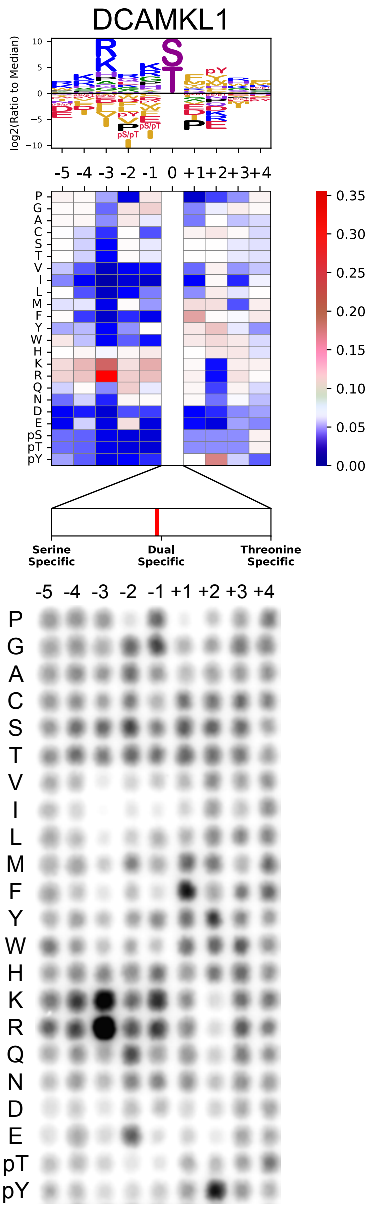
DCAMKL1
log2(Ratio to Median)
10
5
0
−5
−10
-5 -4 -3 -2 -1 0 +1 +2 +3 +4
P G A C S T V I L M F Y W H K R Q N D E pS pT pY
0.35
0.30
0.25
0.20
0.15
0.10
0.05
0.00
Serine Specific
Dual Specific
Threonine Specific
-5 -4 -3 -2 -1 +1 +2 +3 +4
P G A C S T V I L M F Y W H K R Q N D E pT pY

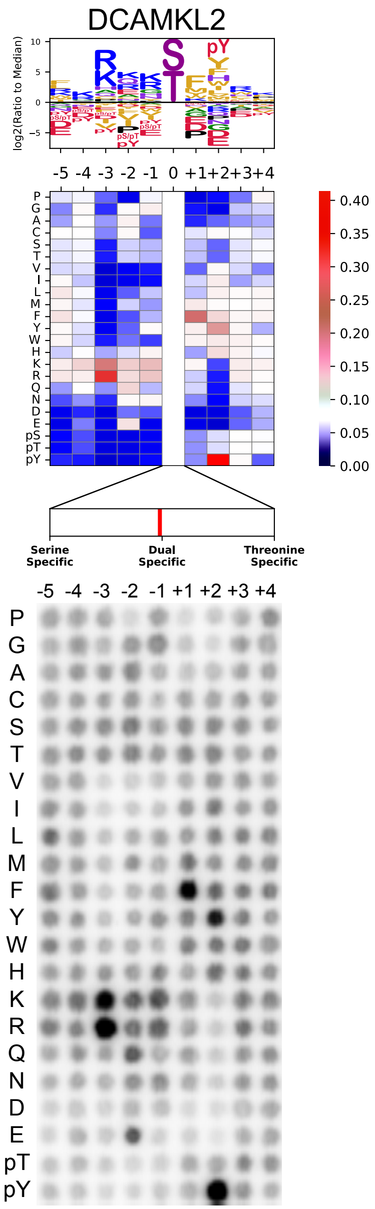
DCAMKL2
log2(Ratio to Median)
10
5
0
−5
-5 -4 -3 -2 -1 0 +1 +2 +3 +4
P G A C S T V I L M F Y W H K R Q N D E pS pT pY
0.40
0.35
0.30
0.25
0.20
0.15
0.10
0.05
0.00
Serine Specific
Dual Specific
Threonine Specific
-5 -4 -3 -2 -1 +1 +2 +3 +4
P G A C S T V I L M F Y W H K R Q N D E pT pY

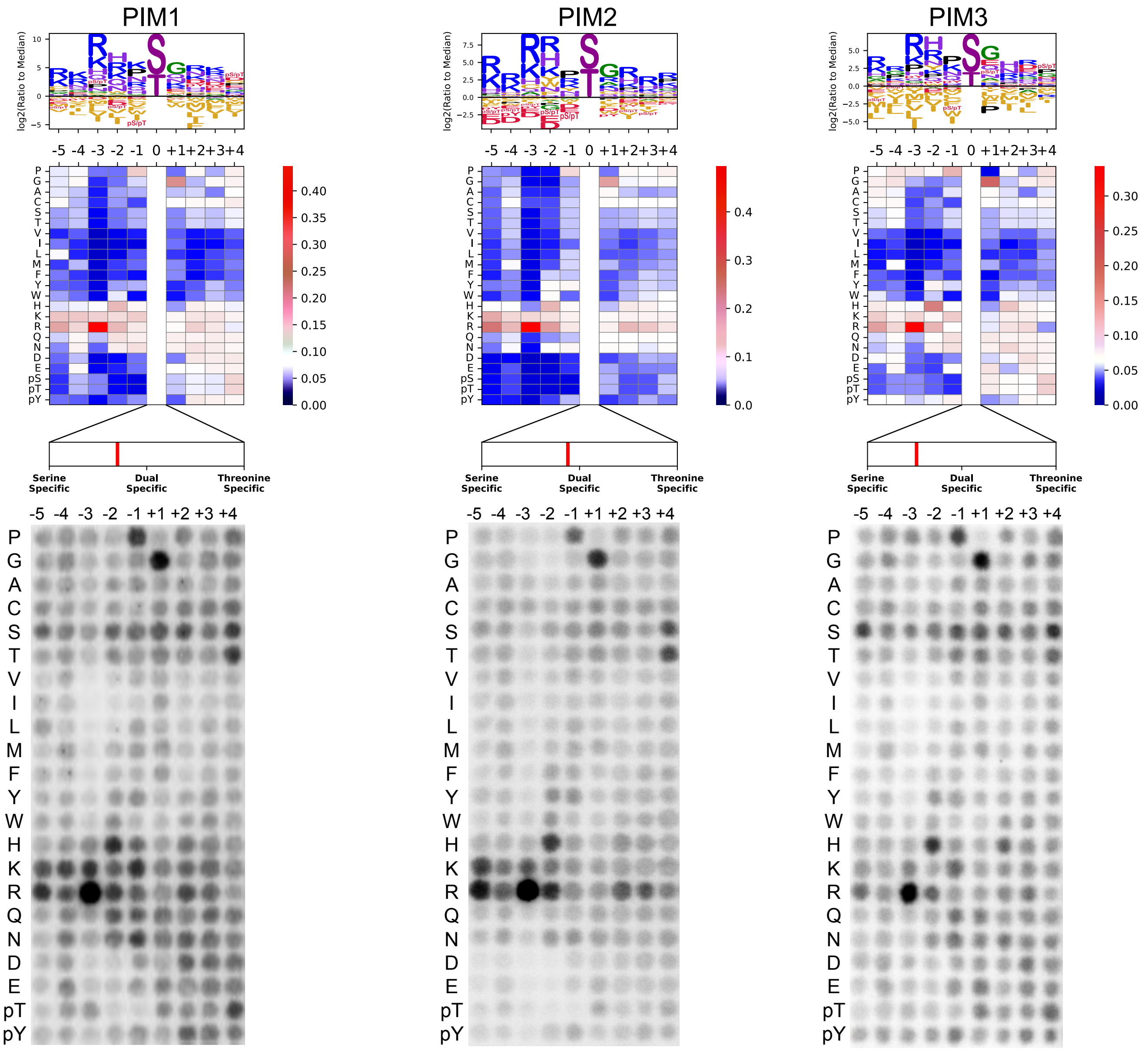
PIM1
PIM2
PIM3
log2(Ratio to Median)
-5 -4 -3 -2 -1 0 +1 +2 +3 +4
P G A C S T V I L M F Y W H K R Q N D E pS pT pY
Serine Specific
Dual Specific
Threonine Specific
-5 -4 -3 -2 -1 +1 +2 +3 +4
P G A C S T V I L M F Y W H K R Q N D E pT pY

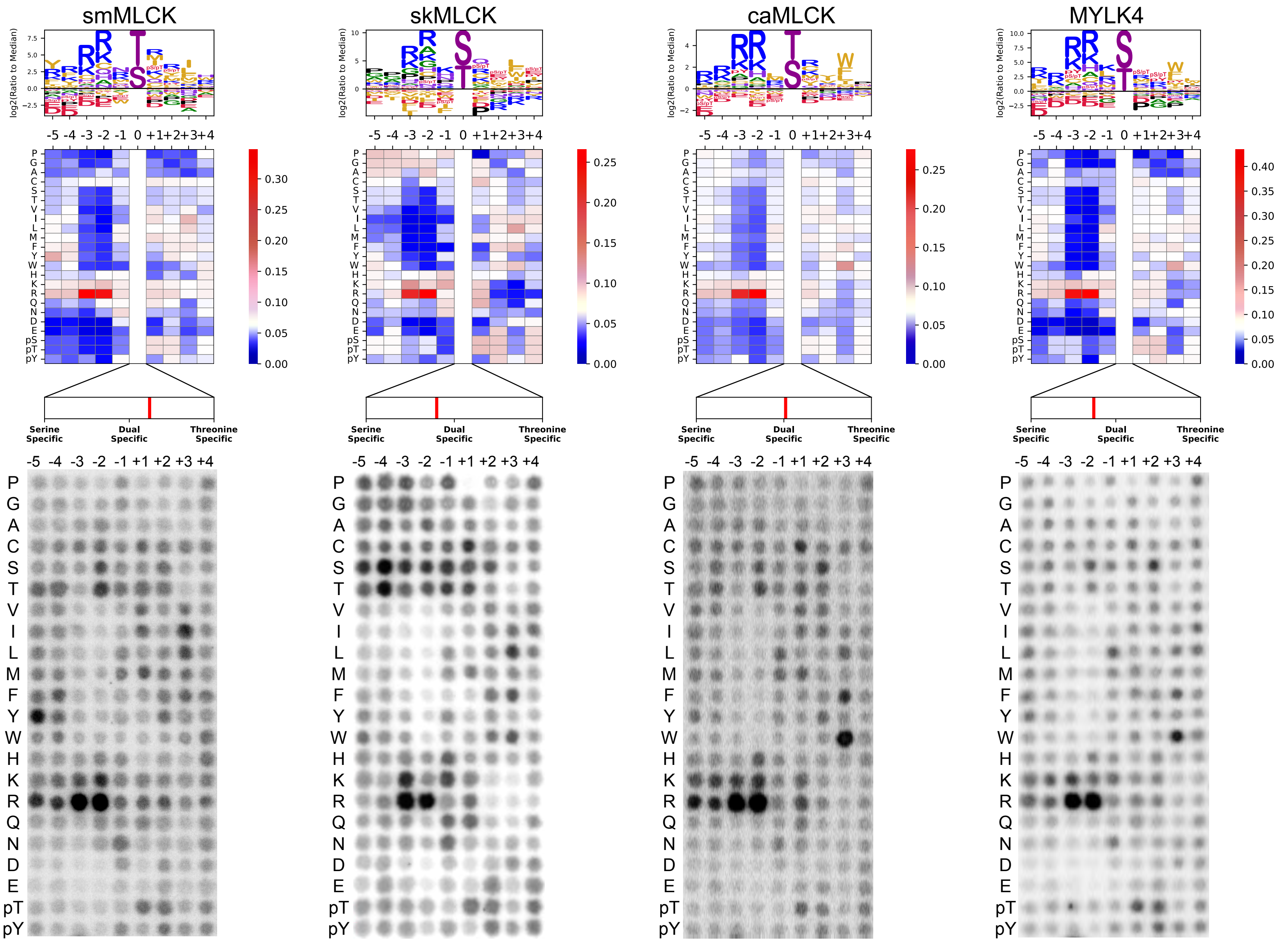

smMLCK
skMLCK
caMLCK
MYLK4
log2(Ratio to Median)
-5 -4 -3 -2 -1 0 +1 +2 +3 +4
P G A C S T V I L M F Y W H K R Q N D E pS pT pY
Serine Specific
Dual Specific
Threonine Specific
-5 -4 -3 -2 -1 +1 +2 +3 +4
P G A C S T V I L M F Y W H K R Q N D E pT pY

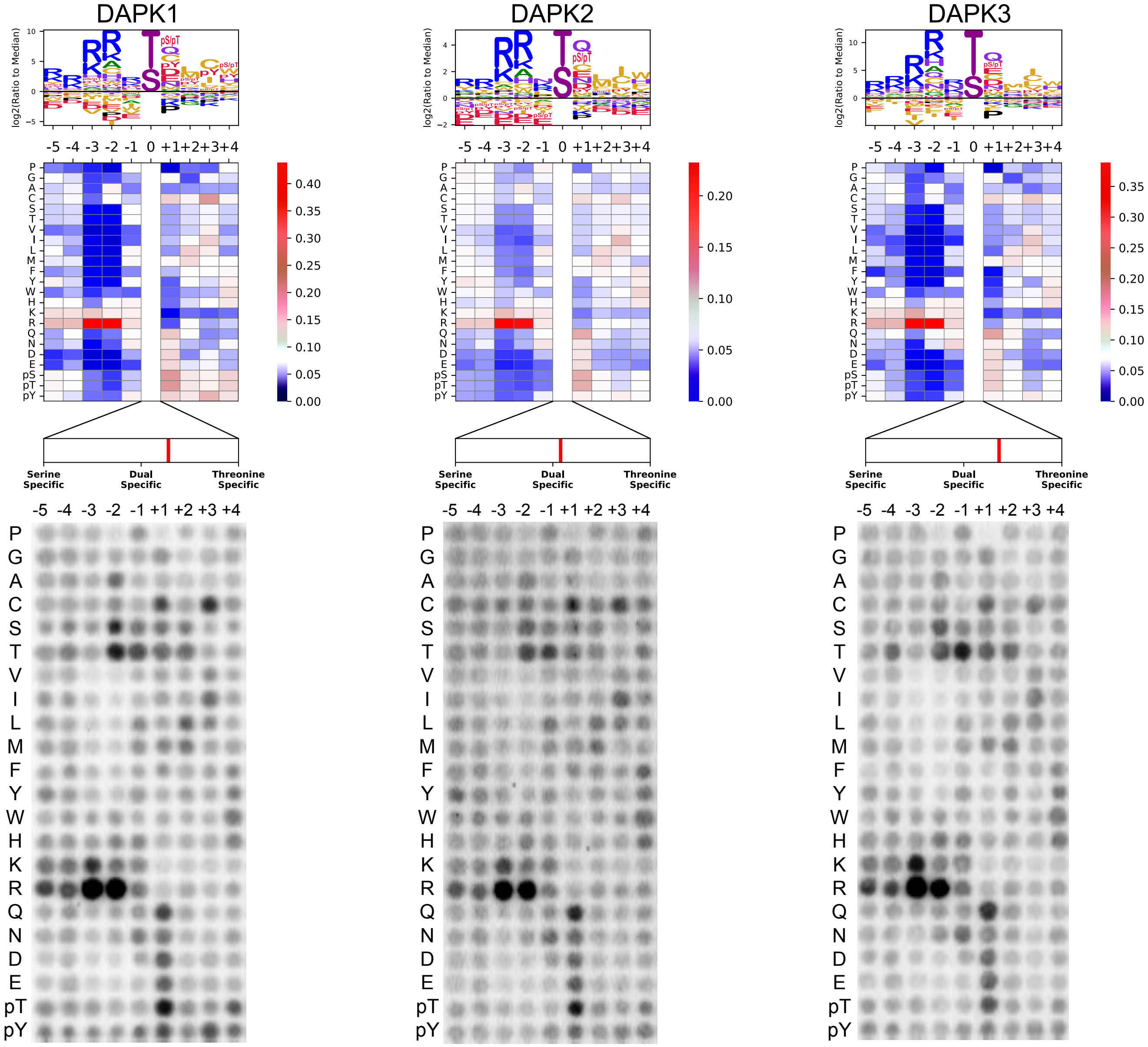

DAPK1
DAPK2
DAPK3
log2(Ratio to Median)
-5 -4 -3 -2 -1 0 +1 +2 +3 +4
P G A C S T V I L M F Y W H K R Q N D E pS pT pY
Serine Specific
Dual Specific
Threonine Specific
P G A C S T V I L M F Y W H K R Q N D E pT pY

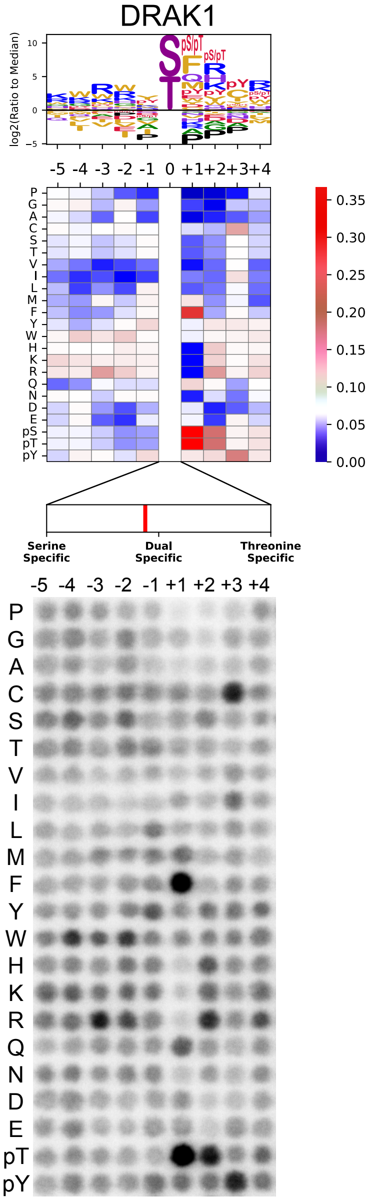
DRAK1
log2(Ratio to Median)
-5 -4 -3 -2 -1 0 +1 +2 +3 +4
P G A C S T V I L M F Y W H K R Q N D E pS pT pY
0.35 0.30 0.25 0.20 0.15 0.10 0.05 0.00
Serine Specific
Dual Specific
Threonine Specific
-5 -4 -3 -2 -1 +1 +2 +3 +4
P G A C S T V I L M F Y W H K R Q N D E pT pY

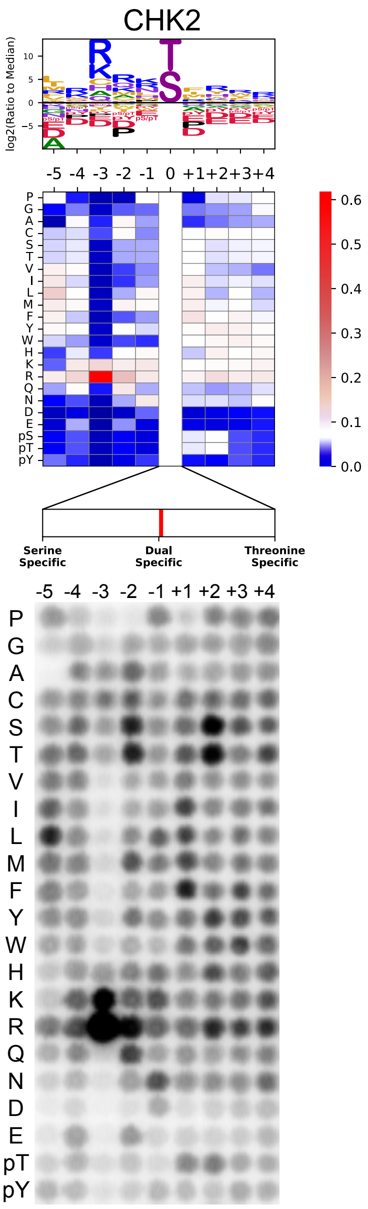
CHK2
log2(Ratio to Median)
-5 -4 -3 -2 -1 0 +1 +2 +3 +4
P G A C S T V I L M F Y W H K R Q N D E pS pT pY
Serine Specific
Dual Specific
Threonine Specific
-5 -4 -3 -2 -1 +1 +2 +3 +4
P G A C S T V I L M F Y W H K R Q N D E pT pY

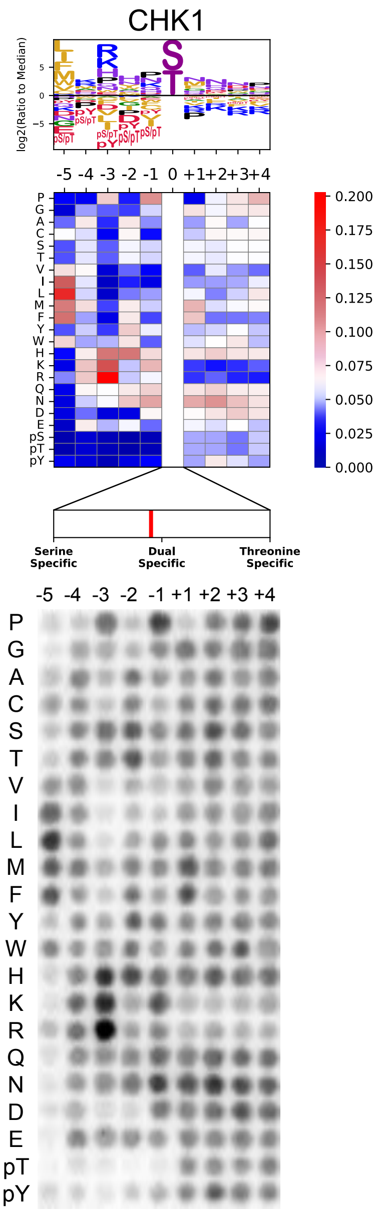
CHK1
log2(Ratio to Median)
-5 -4 -3 -2 -1 0 +1 +2 +3 +4
P G A C S T V I L M F Y W H K R Q N D E pS pT pY
0.200
0.175
0.150
0.125
0.100
0.075
0.050
0.025
0.000
Serine Specific
Dual Specific
Threonine Specific
-5 -4 -3 -2 -1 +1 +2 +3 +4
P G A C S T V I L M F Y W H K R Q N D E pT pY

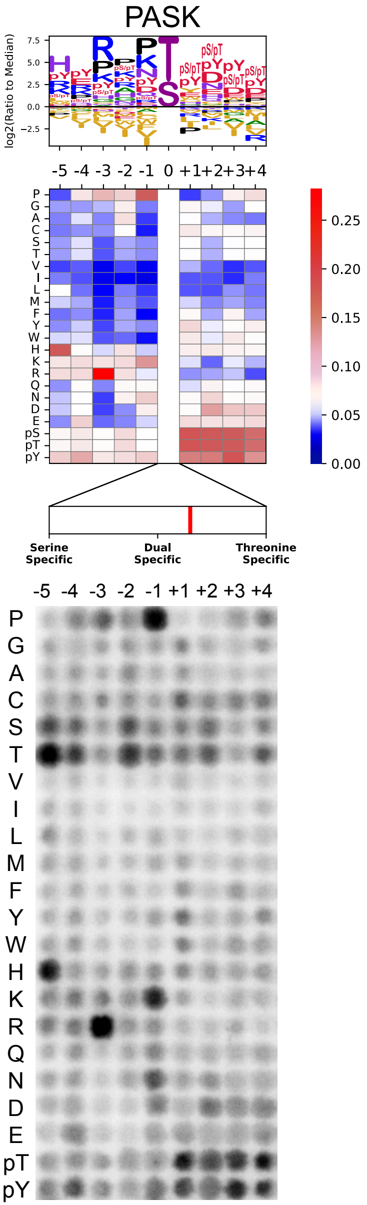
PASK
log2(Ratio to Median)
-5 -4 -3 -2 -1 0 +1 +2 +3 +4
P G A C S T V I L M F Y W H K R Q N D E pS pT pY
0.25 0.20 0.15 0.10 0.05 0.00
Serine Specific
Dual Specific
Threonine Specific
-5 -4 -3 -2 -1 +1 +2 +3 +4
P G A C S T V I L M F Y W H K R Q N D E pT pY

LKB1

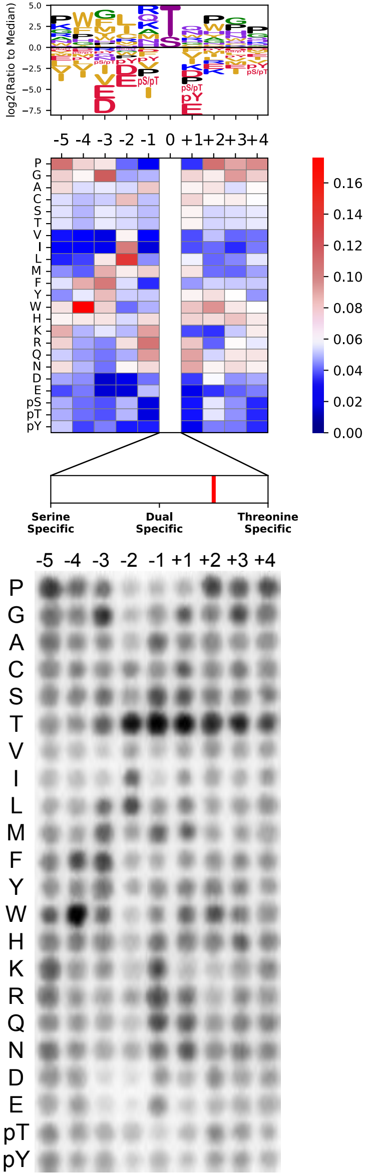
log2(Ratio to Median)
-5 -4 -3 -2 -1 0 +1 +2 +3 +4
P G A C S T V I L M F Y W H K R Q N D E pS pT pY
0.16 0.14 0.12 0.10 0.08 0.06 0.04 0.02 0.00
Serine Specific
Dual Specific
Threonine Specific
-5 -4 -3 -2 -1 +1 +2 +3 +4
P G A C S T V I L M F Y W H K R Q N D E pT pY

CMGC

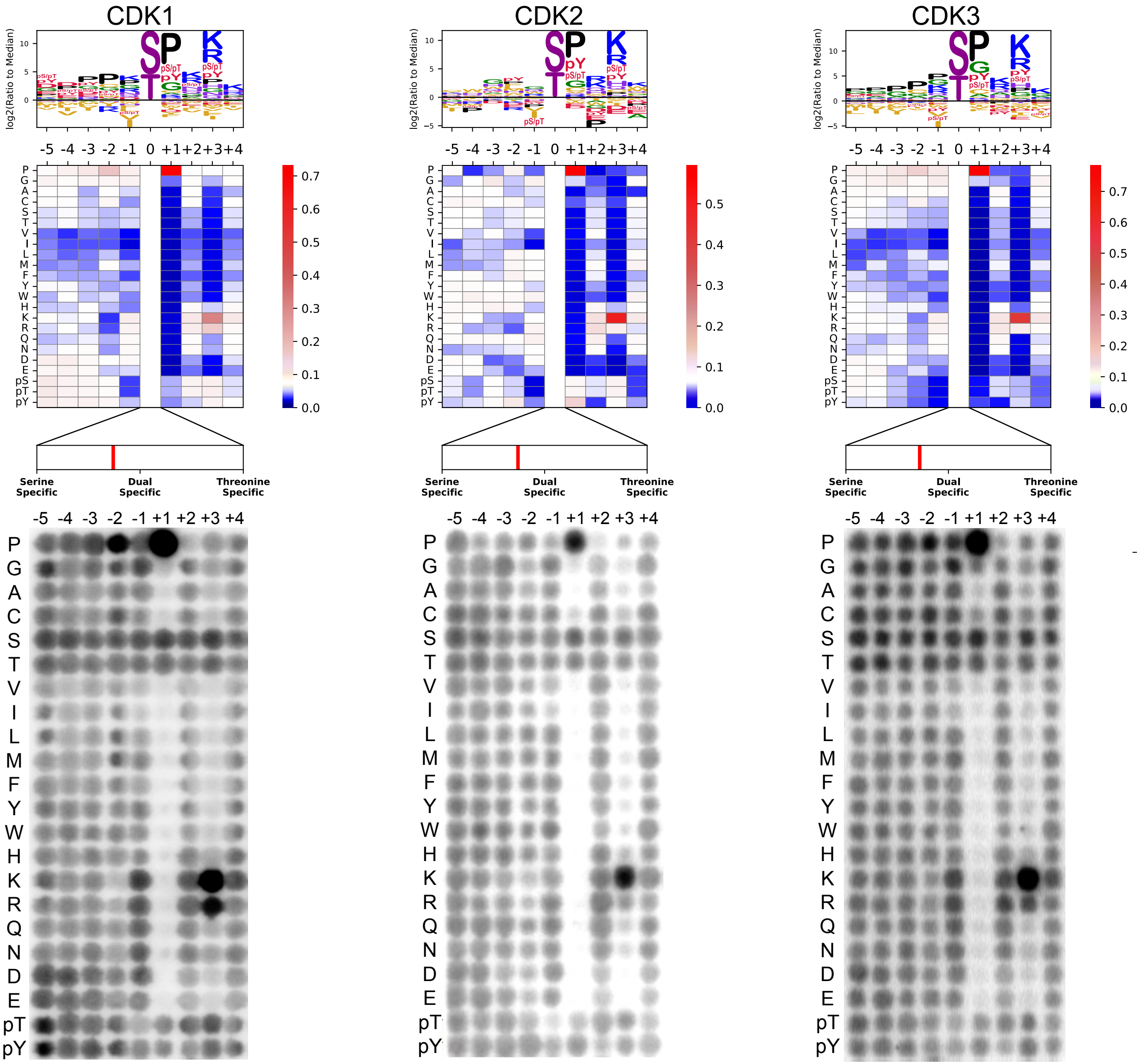
CDK1
CDK2
CDK3
log2(Ratio to Median)
10
5
0
-5
-5 -4 -3 -2 -1 0 +1 +2 +3 +4
P G A C S T V I L M F Y W H K R Q N D E pS pT pY
0.7 0.6 0.5 0.4 0.3 0.2 0.1 0.0
0.5 0.4 0.3 0.2 0.1 0.0
Serine Specific
Dual Specific
Threonine Specific
-5 -4 -3 -2 -1 +1 +2 +3 +4
P G A C S T V I L M F Y W H K R Q N D E pT pY

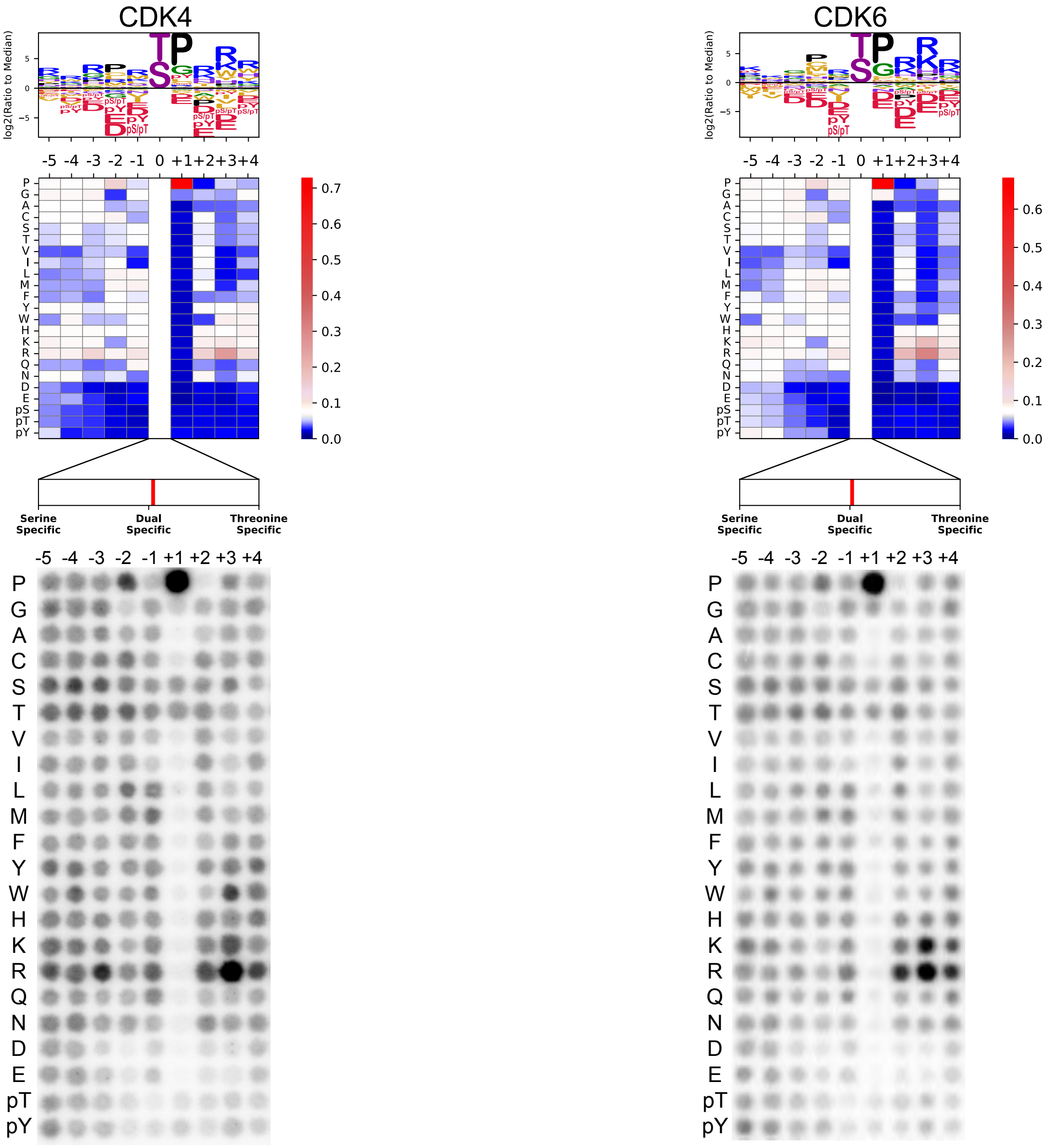

CDK4
CDK6
log2(Ratio to Median)
5
0
-5
-5 -4 -3 -2 -1 0 +1 +2 +3 +4
P G A C S T V I L M F Y W H K R Q N D E pS pT pY
0.7
0.6
0.5
0.4
0.3
0.2
0.1
0.0
Serine Specific
Dual Specific
Threonine Specific
-5 -4 -3 -2 -1 +1 +2 +3 +4
P G A C S T V I L M F Y W H K R Q N D E pT pY

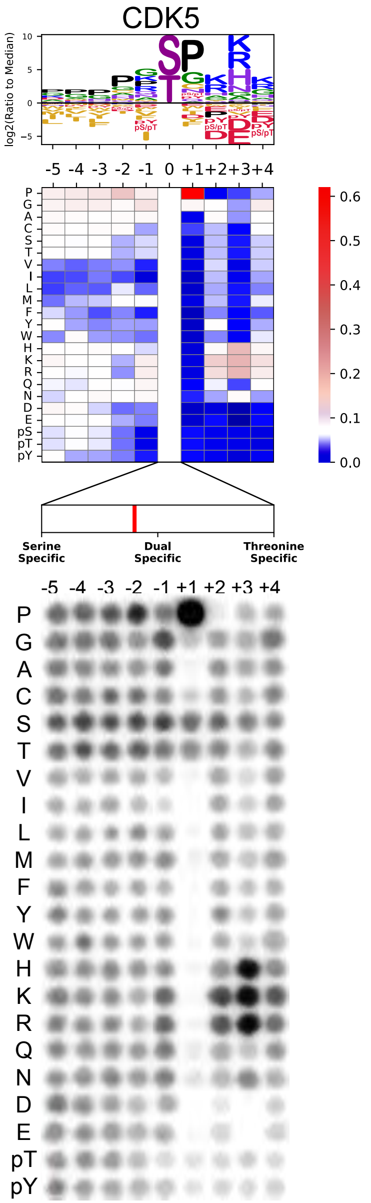
CDK5
log2(Ratio to Median)
-5 -4 -3 -2 -1 0 +1 +2 +3 +4
Serine Specific
Dual Specific
Threonine Specific
-5 -4 -3 -2 -1 +1 +2 +3 +4
P
G
A
C
S
T
V
I
L
M
F
Y
W
H
K
R
Q
N
D
E
pT
pY

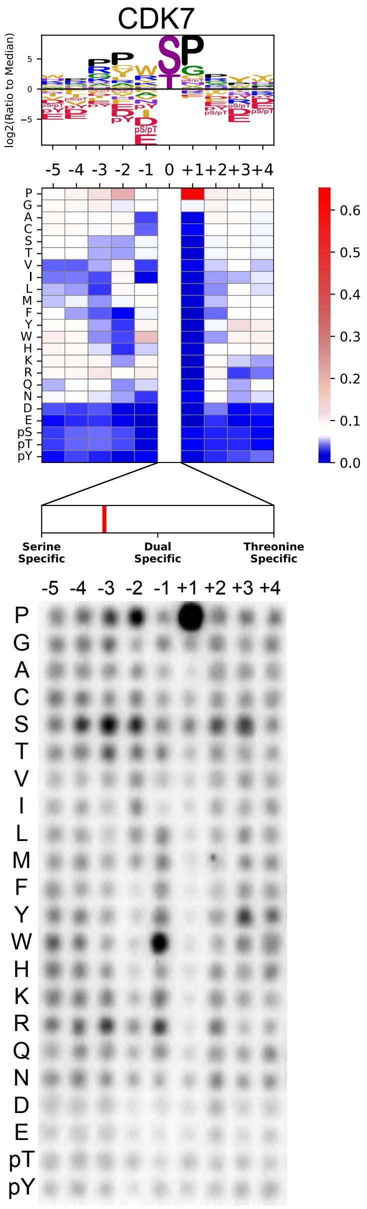
CDK7
log2(Ratio to Median)
-5 -4 -3 -2 -1 0 +1 +2 +3 +4
P G A C S T V I L M F Y W H K R Q N D E pS pT pY
0.6 0.5 0.4 0.3 0.2 0.1 0.0
Serine Specific
Dual Specific
Threonine Specific
-5 -4 -3 -2 -1 +1 +2 +3 +4
P G A C S T V I L M F Y W H K R Q N D E pT pY

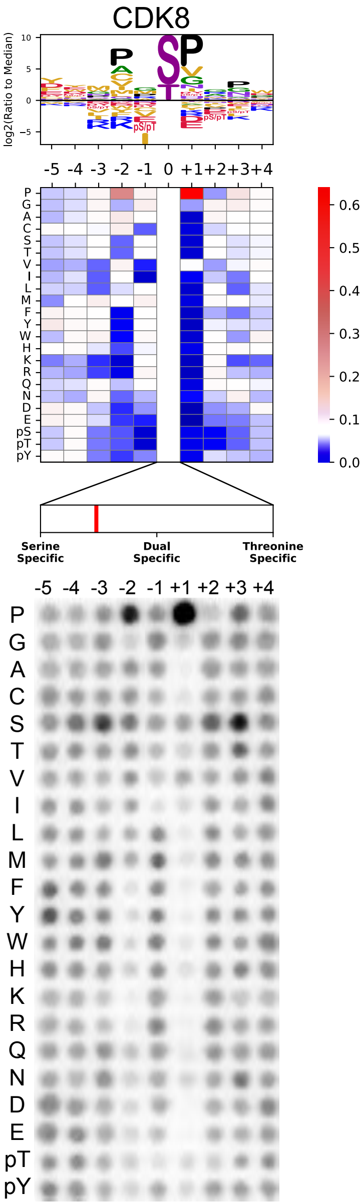
CDK8
log2(Ratio to Median)
10
5
0
-5
-5 -4 -3 -2 -1 0 +1 +2 +3 +4
P G A C S T V I L M F Y W H K R Q N D E pS pT pY
0.6
0.5
0.4
0.3
0.2
0.1
0.0
Serine Specific
Dual Specific
Threonine Specific
-5 -4 -3 -2 -1 +1 +2 +3 +4
P G A C S T V I L M F Y W H K R Q N D E pT pY

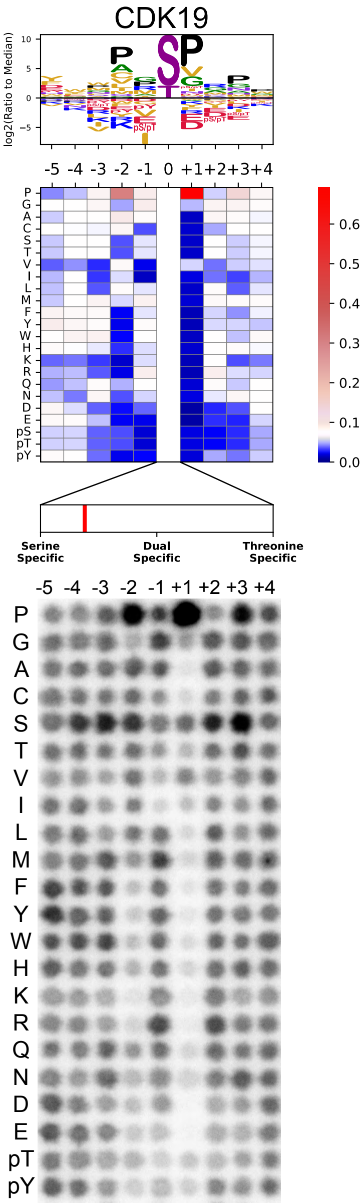
CDK19
log2(Ratio to Median)
10
5
0
-5
-5 -4 -3 -2 -1 0 +1 +2 +3 +4
P G A C S T V I L M F Y W H K R Q N D E pS pT pY
0.6
0.5
0.4
0.3
0.2
0.1
0.0
Serine Specific
Dual Specific
Threonine Specific
-5 -4 -3 -2 -1 +1 +2 +3 +4
P G A C S T V I L M F Y W H K R Q N D E pT pY

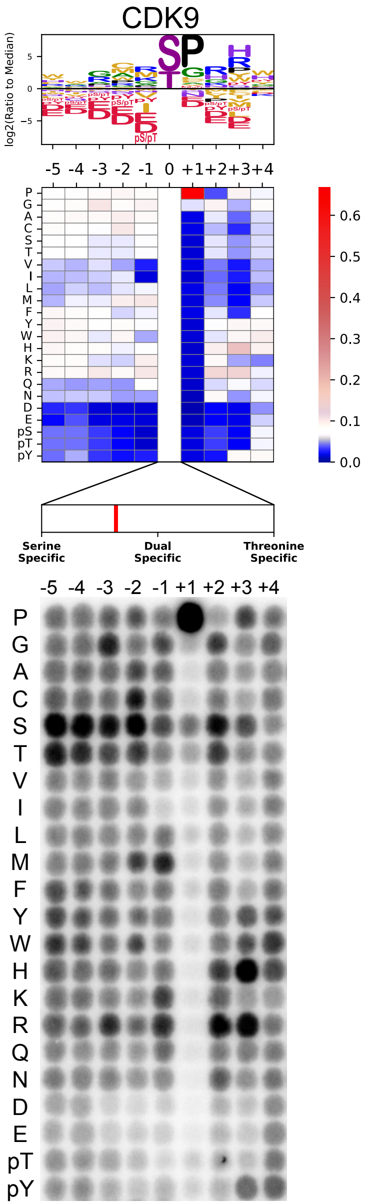
CDK9
log2(Ratio to Median)
-5 -4 -3 -2 -1 0 +1 +2 +3 +4
P G A C S T V I L M F Y W H K R Q N D E pS pT pY
0.6 0.5 0.4 0.3 0.2 0.1 0.0
Serine Specific
Dual Specific
Threonine Specific
-5 -4 -3 -2 -1 +1 +2 +3 +4
P G A C S T V I L M F Y W H K R Q N D E pT pY

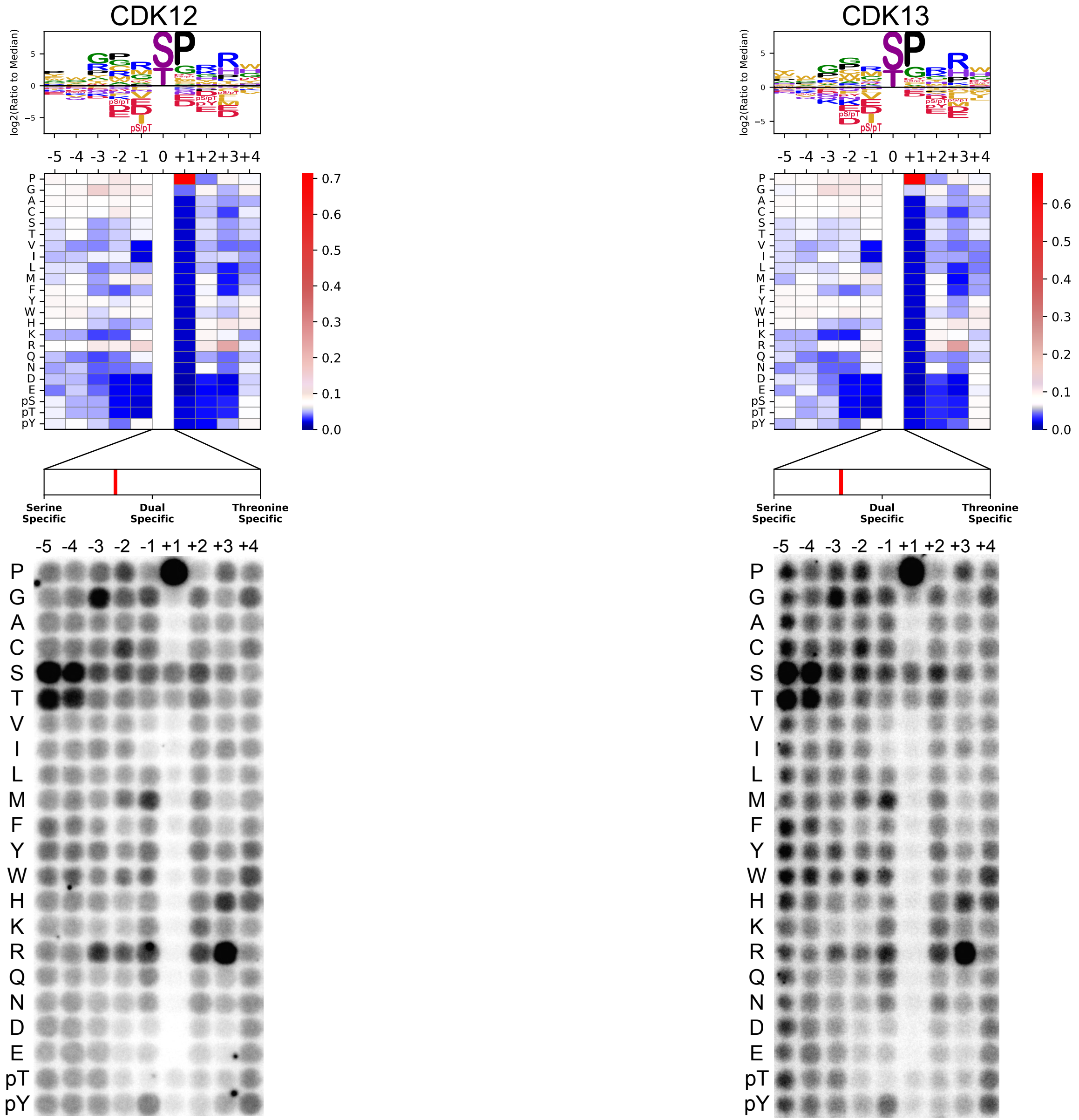

CDK12
CDK13
log2(Ratio to Median)
-5 -4 -3 -2 -1 0 +1 +2 +3 +4
P G A C S T V I L M F Y W H K R Q N D E pS pT pY
Serine Specific
Dual Specific
Threonine Specific
-5 -4 -3 -2 -1 +1 +2 +3 +4
P G A C S T V I L M F Y W H K R Q N D E pT pY

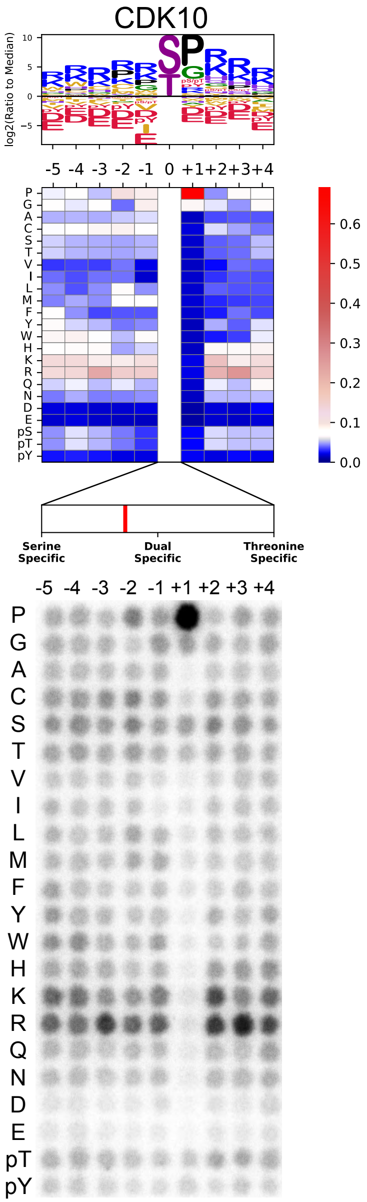
CDK10
log2(Ratio to Median)
10
5
0
−5
-5 -4 -3 -2 -1 0 +1 +2 +3 +4
P
G
A
C
S
T
V
I
L
M
F
Y
W
H
K
R
Q
N
D
E
pS
pT
pY
0.6
0.5
0.4
0.3
0.2
0.1
0.0
Serine Specific
Dual Specific
Threonine Specific
-5 -4 -3 -2 -1 +1 +2 +3 +4
P
G
A
C
S
T
V
I
L
M
F
Y
W
H
K
R
Q
N
D
E
pT
pY

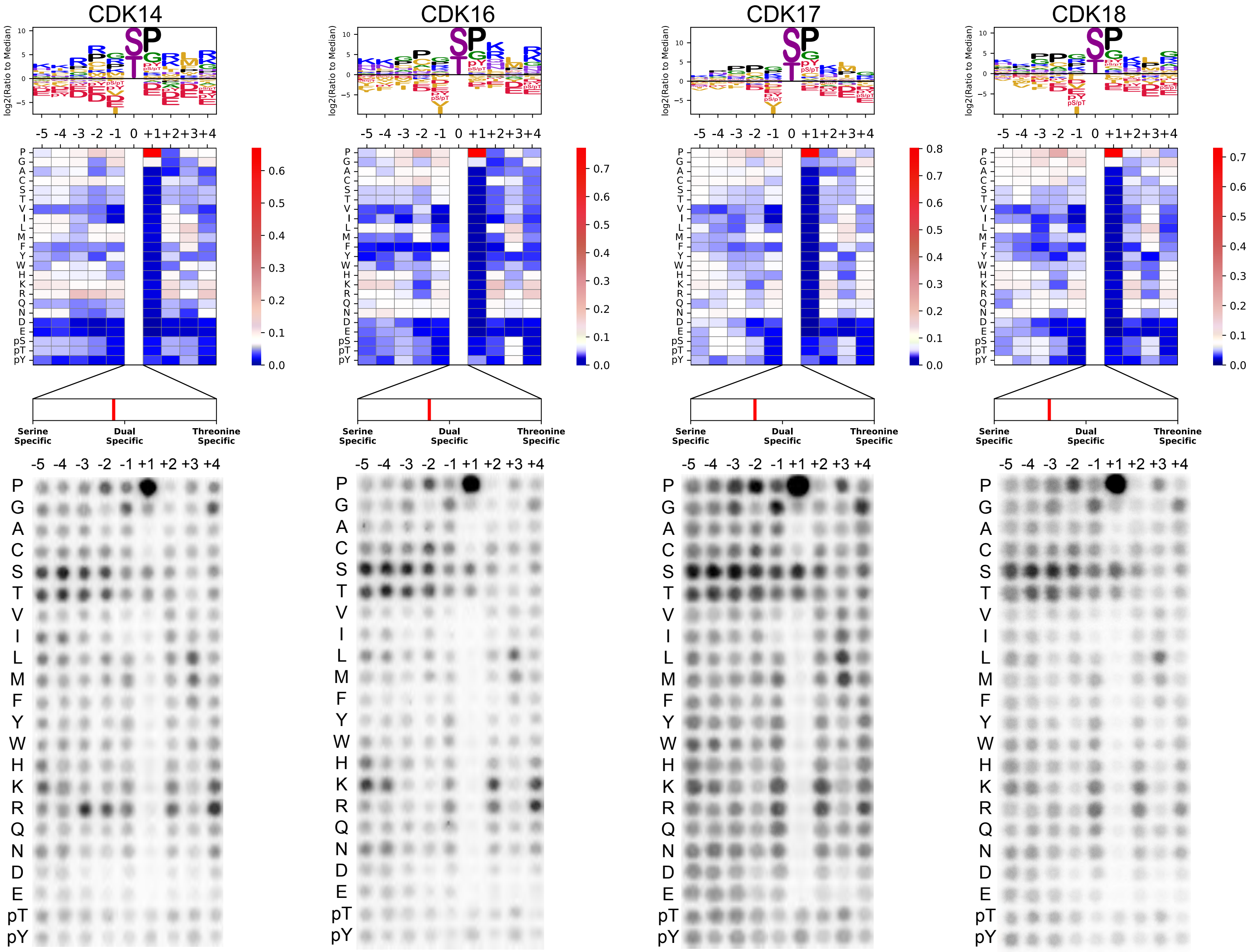
CDK14
CDK16
CDK17
CDK18
log2(Ratio to Median)
-5 -4 -3 -2 -1 0 +1 +2 +3 +4
P G A C S T V I L M F Y W H K R Q N D E pS pT pY
Serine Specific
Dual Specific
Threonine Specific
-5 -4 -3 -2 -1 +1 +2 +3 +4
P G A C S T V I L M F Y W H K R Q N D E pT pY

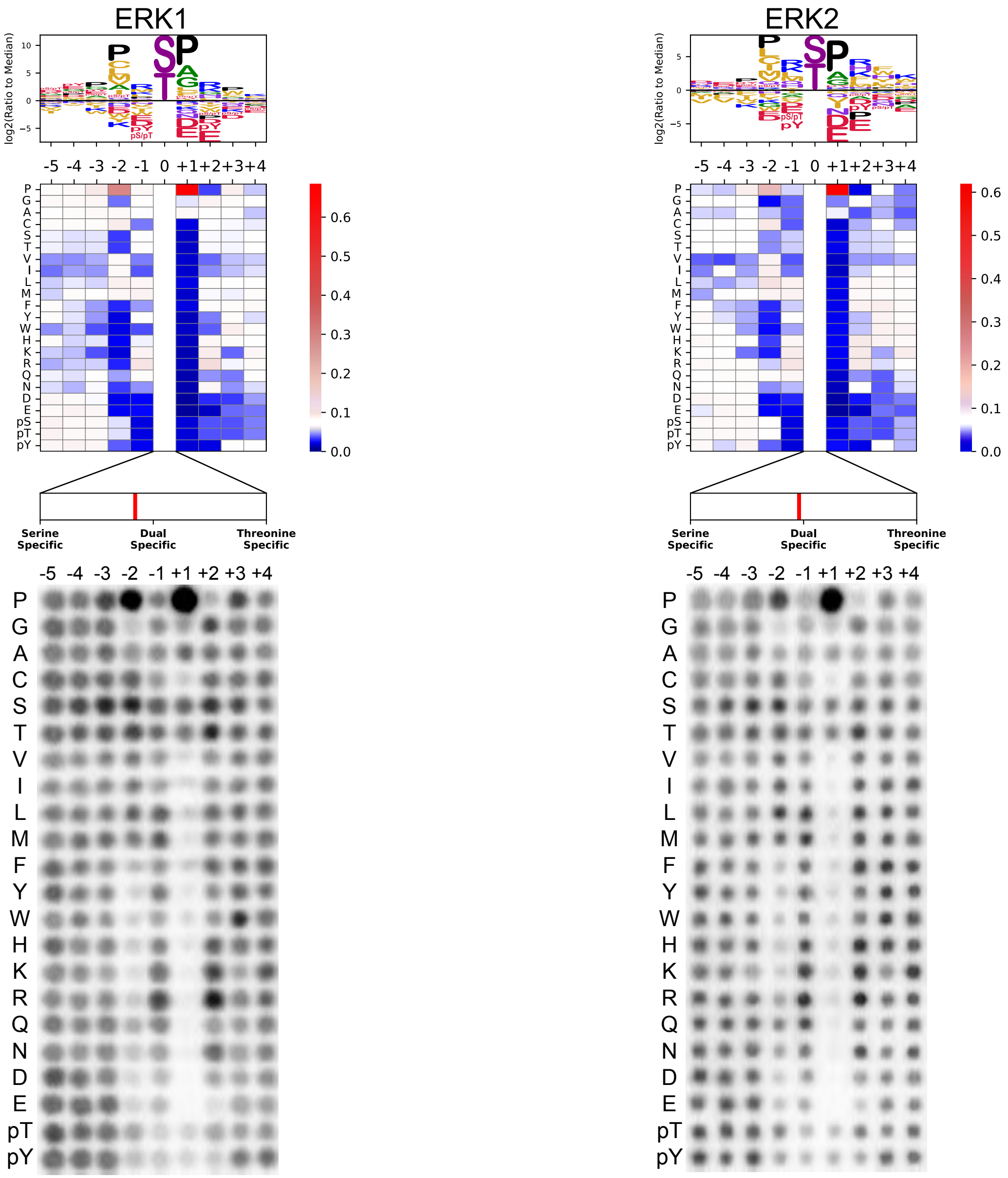
ERK1
ERK2
log2(Ratio to Median)
-5 -4 -3 -2 -1 0 +1 +2 +3 +4
P G A C S T V I L M F Y W H K R Q N D E pS pT pY
0.0 0.1 0.2 0.3 0.4 0.5 0.6
Serine Specific
Dual Specific
Threonine Specific
-5 -4 -3 -2 -1 +1 +2 +3 +4
P G A C S T V I L M F Y W H K R Q N D E pT pY

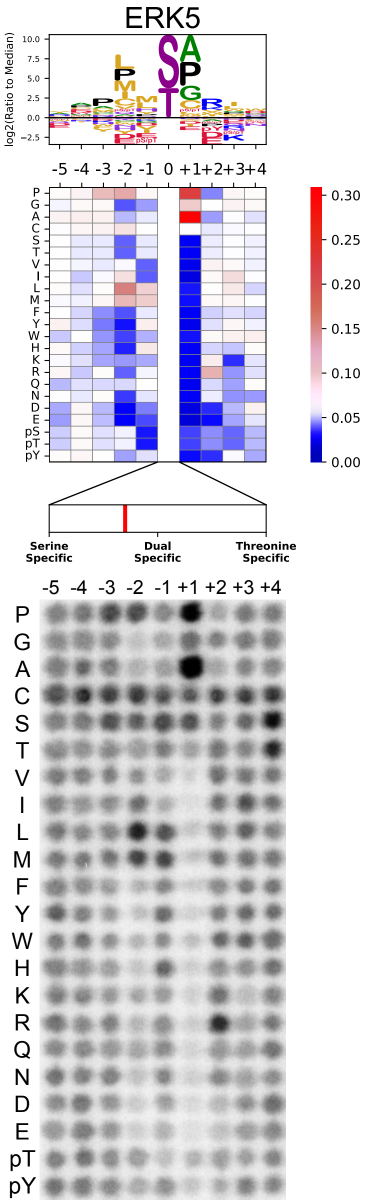
ERK5
log2(Ratio to Median)
-5 -4 -3 -2 -1 0 +1 +2 +3 +4
P G A C S T V I L M F Y W H K R Q N D E pS pT pY
Serine Specific
Dual Specific
Threonine Specific
-5 -4 -3 -2 -1 +1 +2 +3 +4
P G A C S T V I L M F Y W H K R Q N D E pT pY

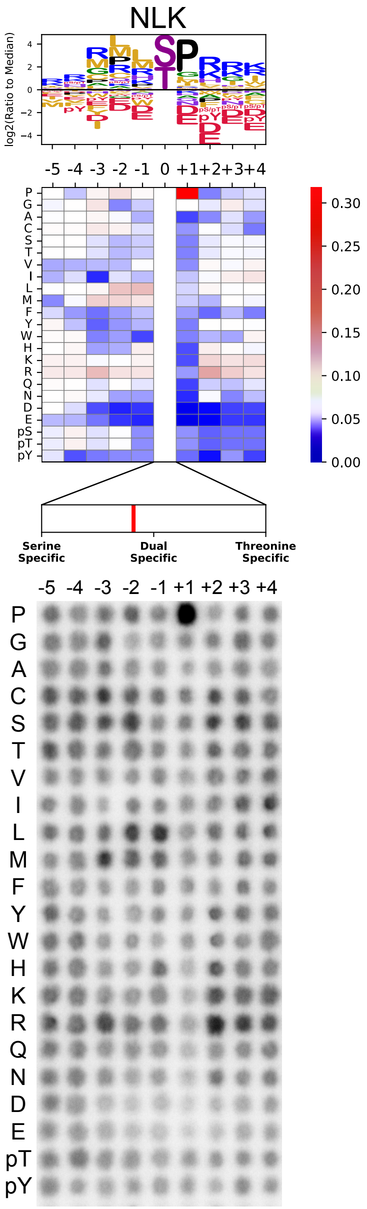
NLK
log2(Ratio to Median)
-5 -4 -3 -2 -1 0 +1 +2 +3 +4
P G A C S T V I L M F Y W H K R Q N D E pS pT pY
0.30 0.25 0.20 0.15 0.10 0.05 0.00
Serine Specific
Dual Specific
Threonine Specific
-5 -4 -3 -2 -1 +1 +2 +3 +4
P G A C S T V I L M F Y W H K R Q N D E pT pY

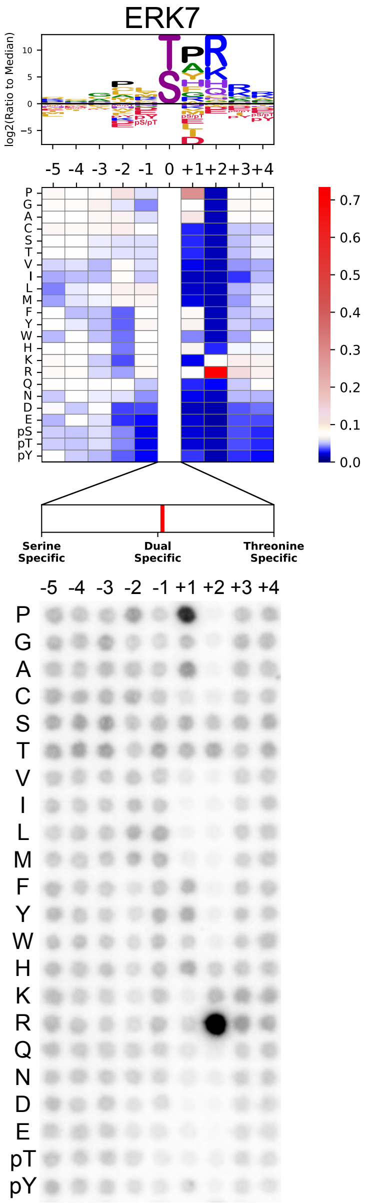
ERK7
log2(Ratio to Median)
-5 -4 -3 -2 -1 0 +1 +2 +3 +4
P G A C S T V I L M F Y W H K R Q N D E pS pT pY
Serine Specific
Dual Specific
Threonine Specific
-5 -4 -3 -2 -1 +1 +2 +3 +4
P G A C S T V I L M F Y W H K R Q N D E pT pY

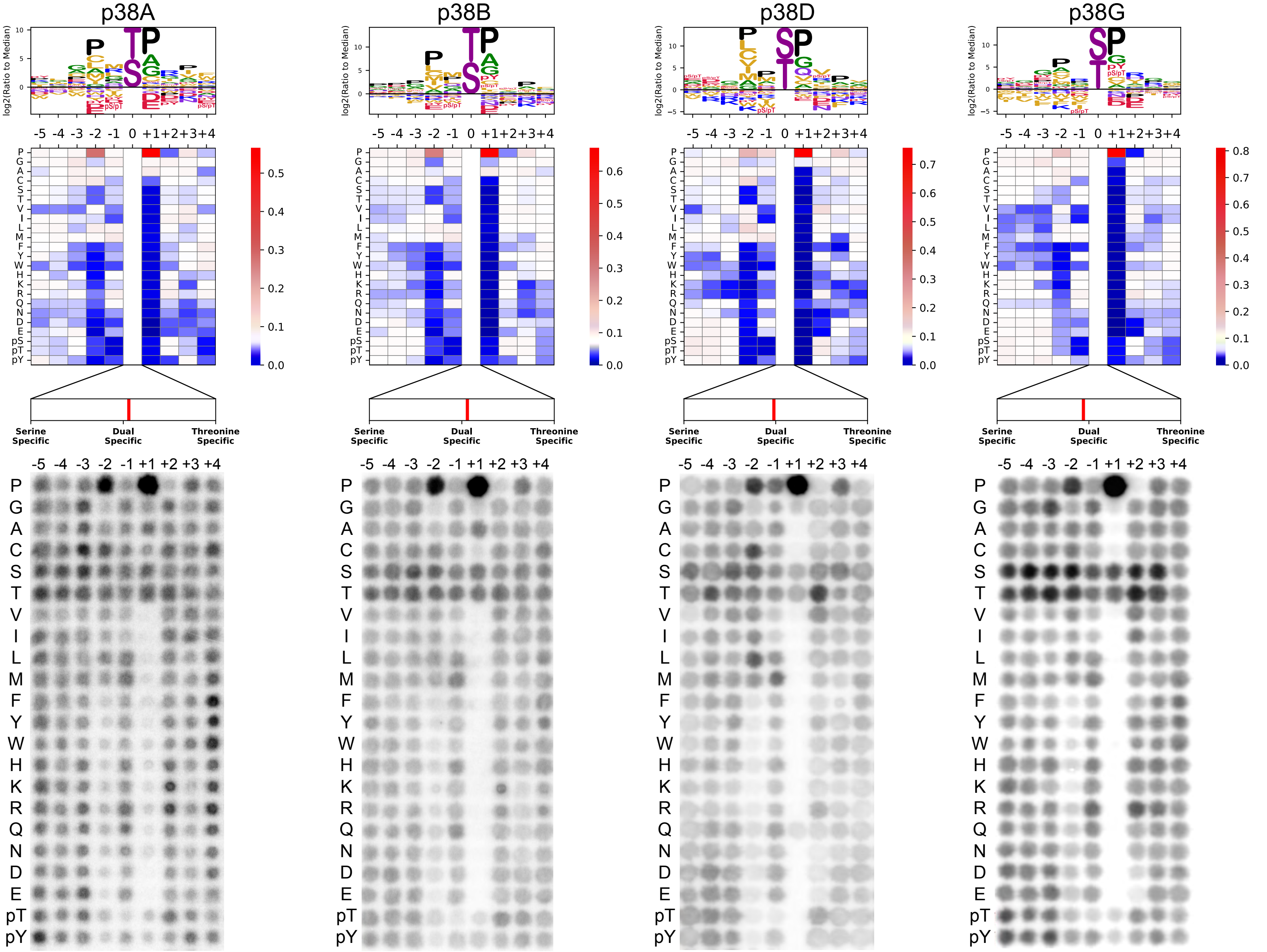

p38A
p38B
p38D
p38G
log2(Ratio to Median)
-5 -4 -3 -2 -1 0 +1 +2 +3 +4
P G A C S T V I L M F Y W H K R Q N D E pS pT pY
Serine Specific
Dual Specific
Threonine Specific
-5 -4 -3 -2 -1 +1 +2 +3 +4
P G A C S T V I L M F Y W H K R Q N D E pT pY

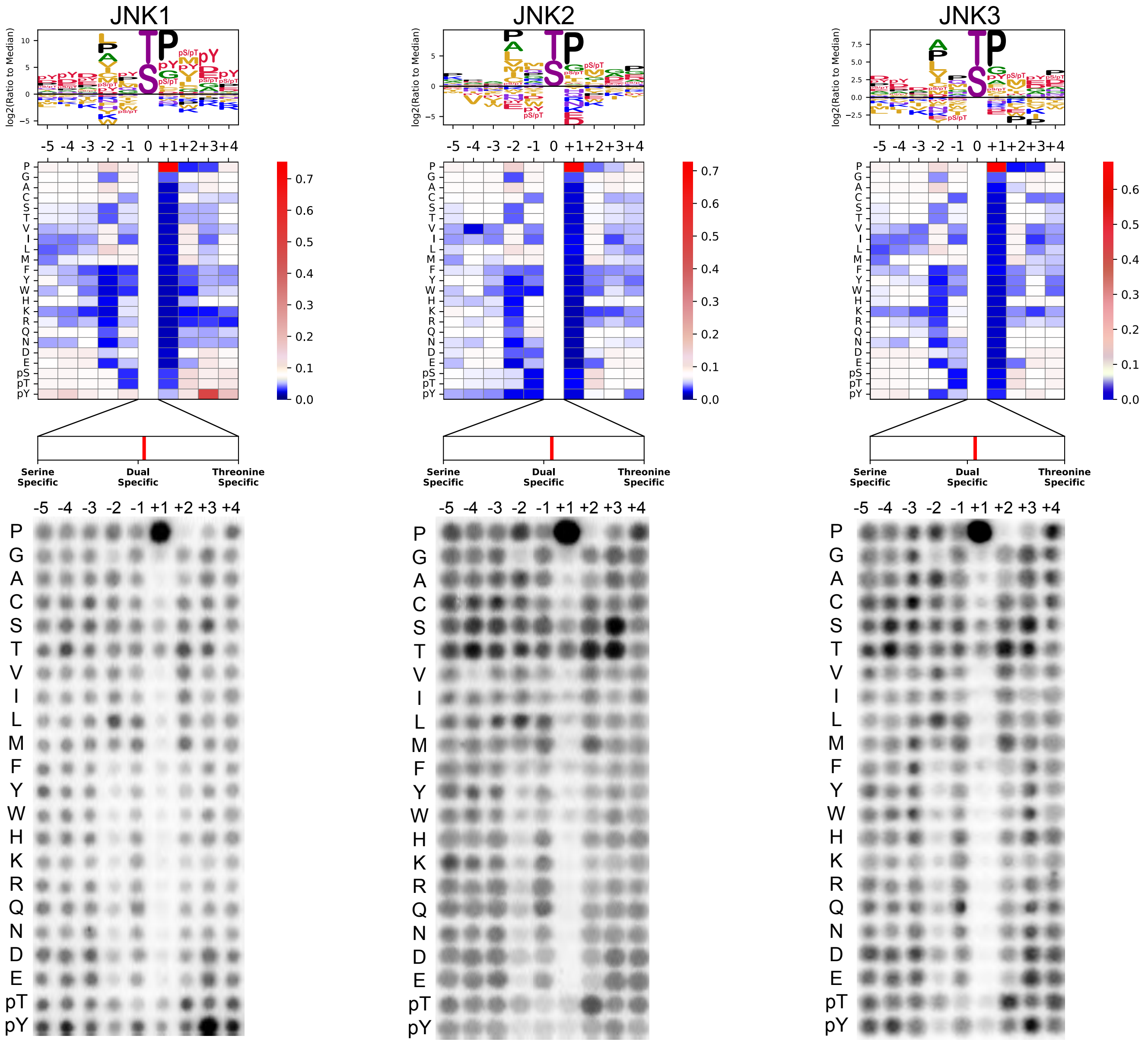

JNK1
JNK2
JNK3
log2(Ratio to Median)
-5 -4 -3 -2 -1 0 +1 +2 +3 +4
P G A C S T V I L M F Y W H K R Q N D E pS pT pY
Serine Specific
Dual Specific
Threonine Specific
-5 -4 -3 -2 -1 +1 +2 +3 +4
P G A C S T V I L M F Y W H K R Q N D E pT pY

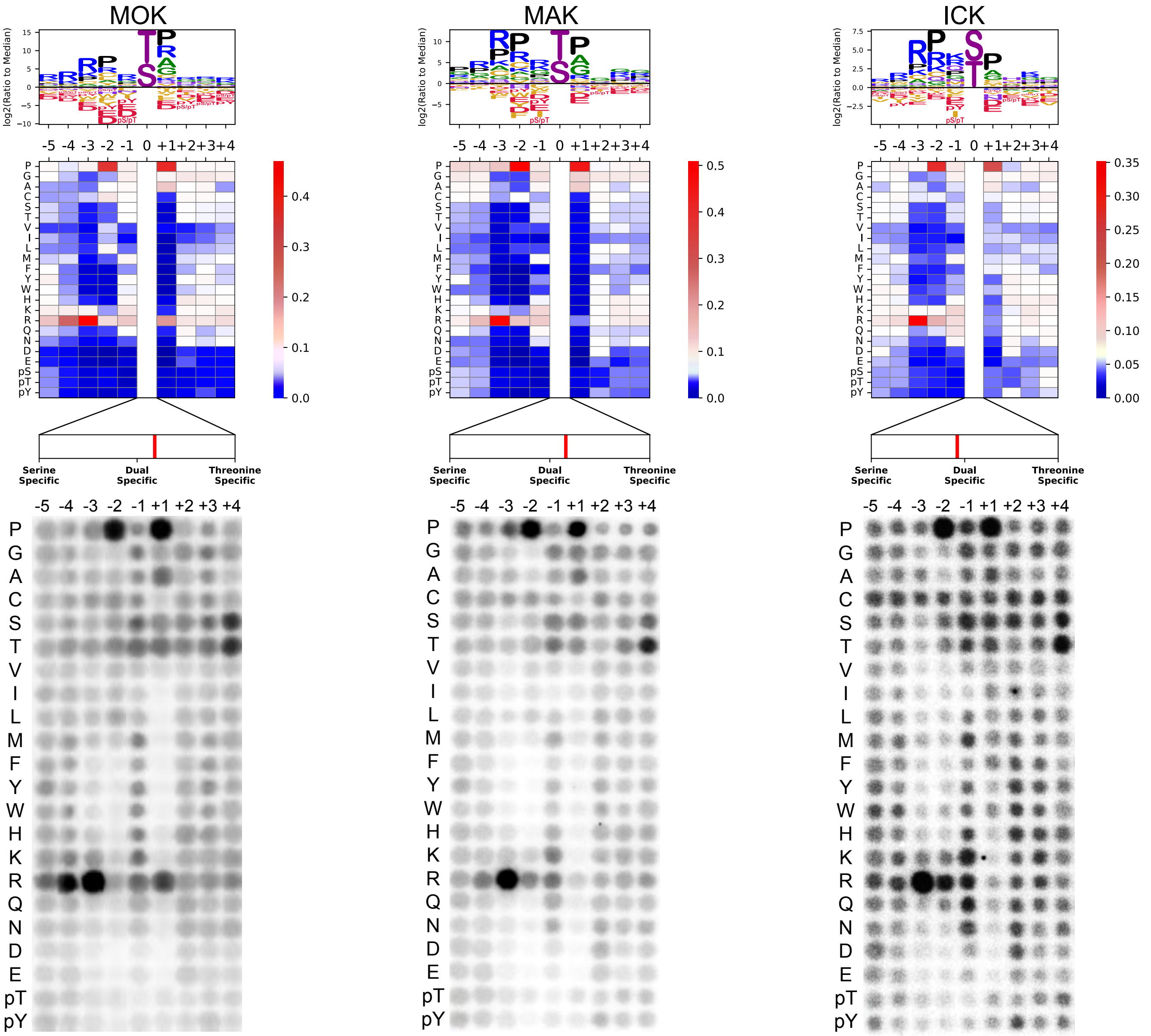
MOK
MAK
ICK
log2(Ratio to Median)
-5 -4 -3 -2 -1 0 +1 +2 +3 +4
P G A C S T V I L M F Y W H K R Q N D E pS pT pY
pS/pT
0.0 0.1 0.2 0.3 0.4
0.0 0.1 0.2 0.3 0.4 0.5
0.00 0.05 0.10 0.15 0.20 0.25 0.30 0.35
Serine Specific
Dual Specific
Threonine Specific
-5 -4 -3 -2 -1 +1 +2 +3 +4
P G A C S T V I L M F Y W H K R Q N D E pT pY

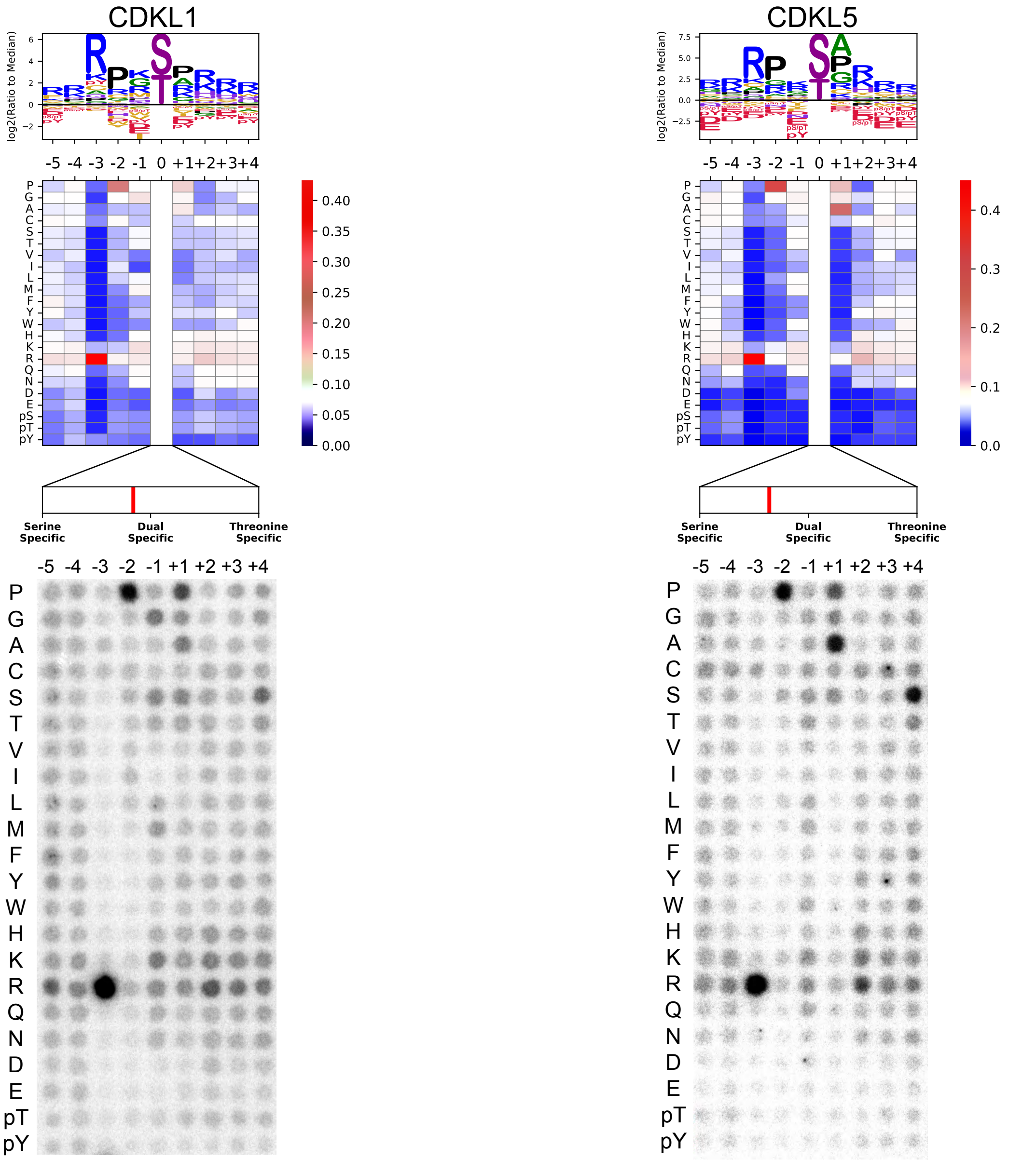

CDKL1
CDKL5
log2(Ratio to Median)
-5 -4 -3 -2 -1 0 +1 +2 +3 +4
P G A C S T V I L M F Y W H K R Q N D E pS pT pY
Serine Specific
Dual Specific
Threonine Specific
P G A C S T V I L M F Y W H K R Q N D E pT pY

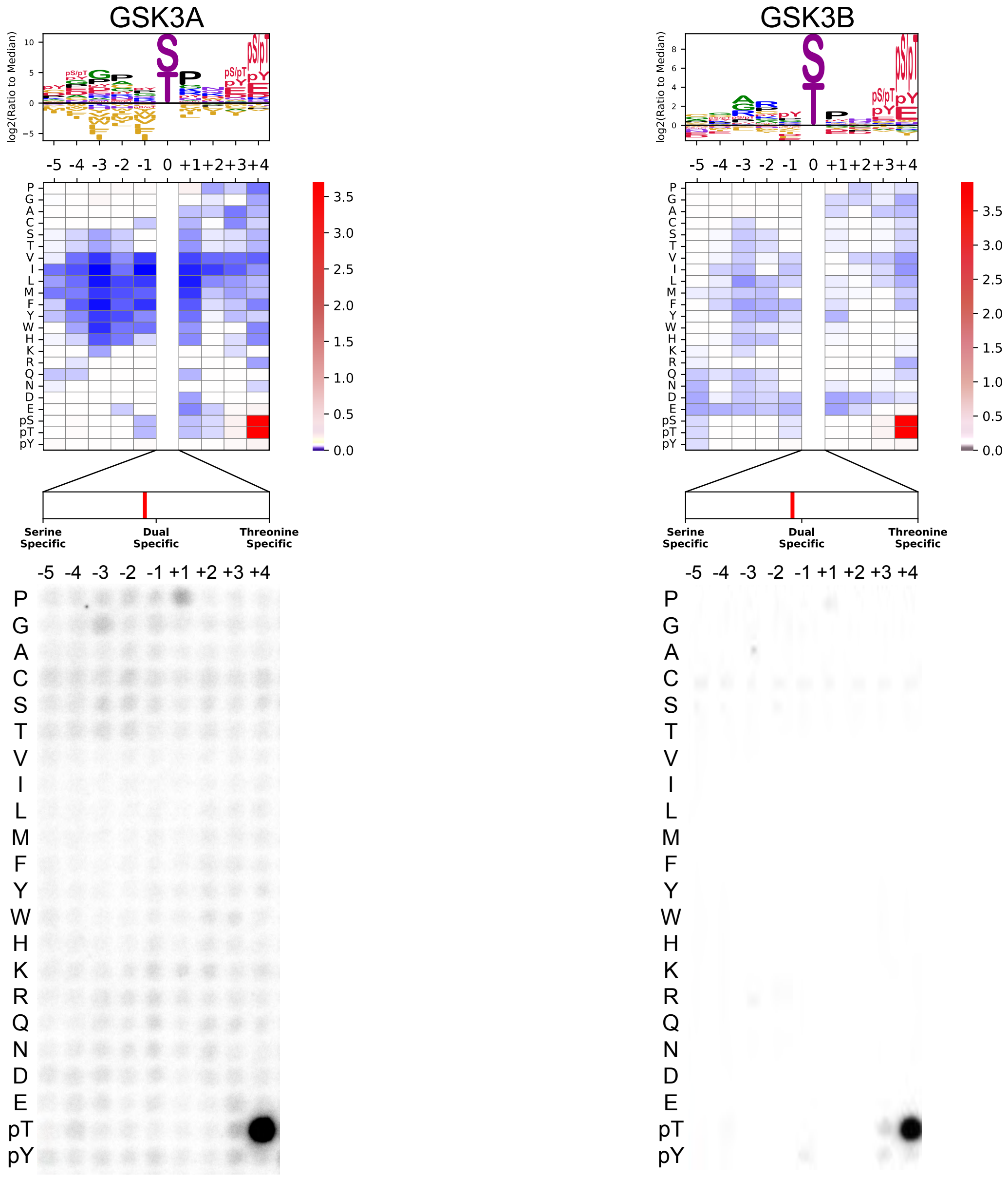

GSK3A
GSK3B
log2(Ratio to Median)
-5 -4 -3 -2 -1 0 +1 +2 +3 +4
P G A C S T V I L M F Y W H K R Q N D E pS pT pY
Serine Specific
Dual Specific
Threonine Specific

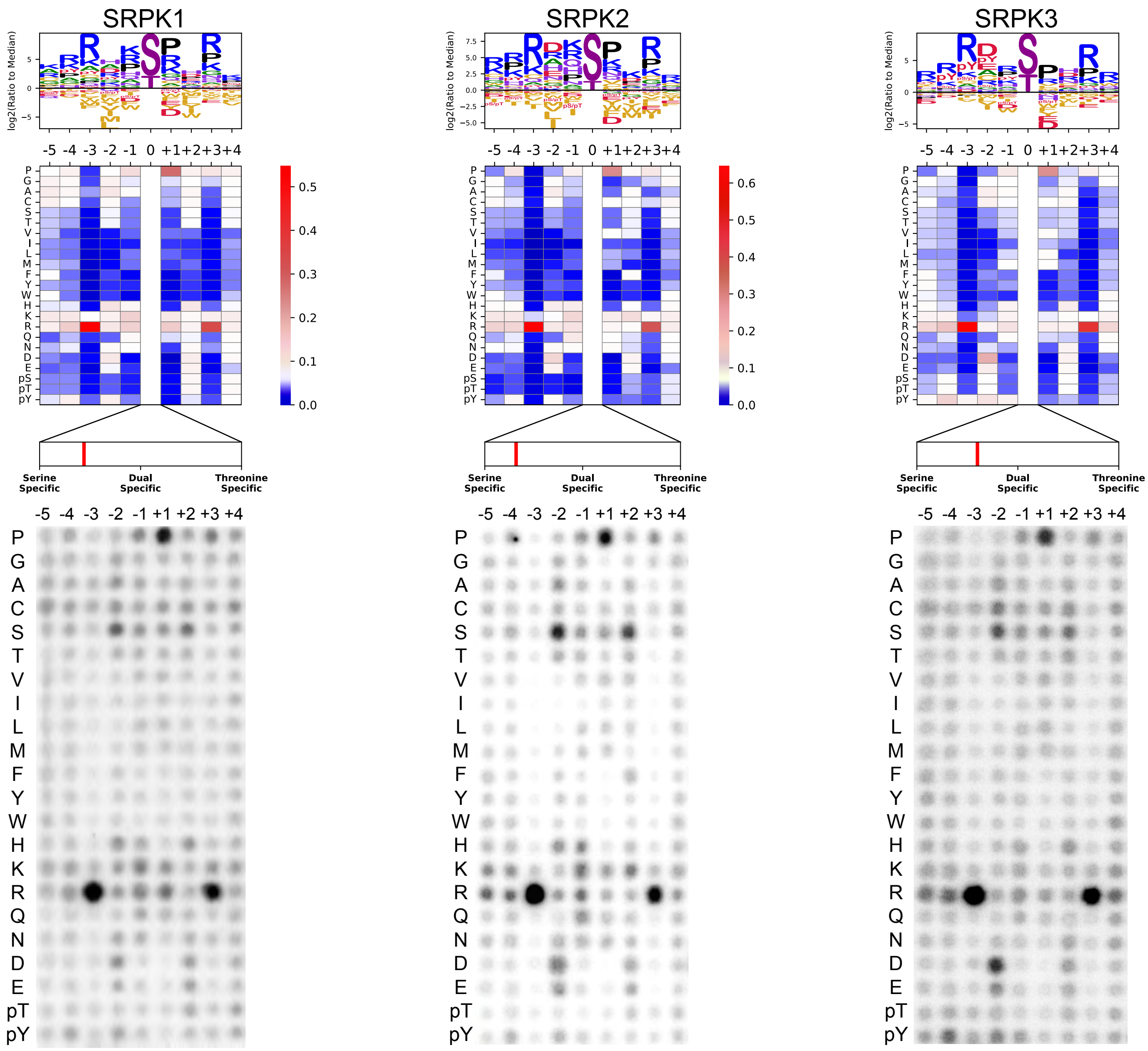
SRPK1
SRPK2
SRPK3
log2(Ratio to Median)
-5 -4 -3 -2 -1 0 +1 +2 +3 +4
P G A C S T V I L M F Y W H K R Q N D E pS pT pY
Serine Specific
Dual Specific
Threonine Specific
-5 -4 -3 -2 -1 +1 +2 +3 +4
P G A C S T V I L M F Y W H K R Q N D E pT pY

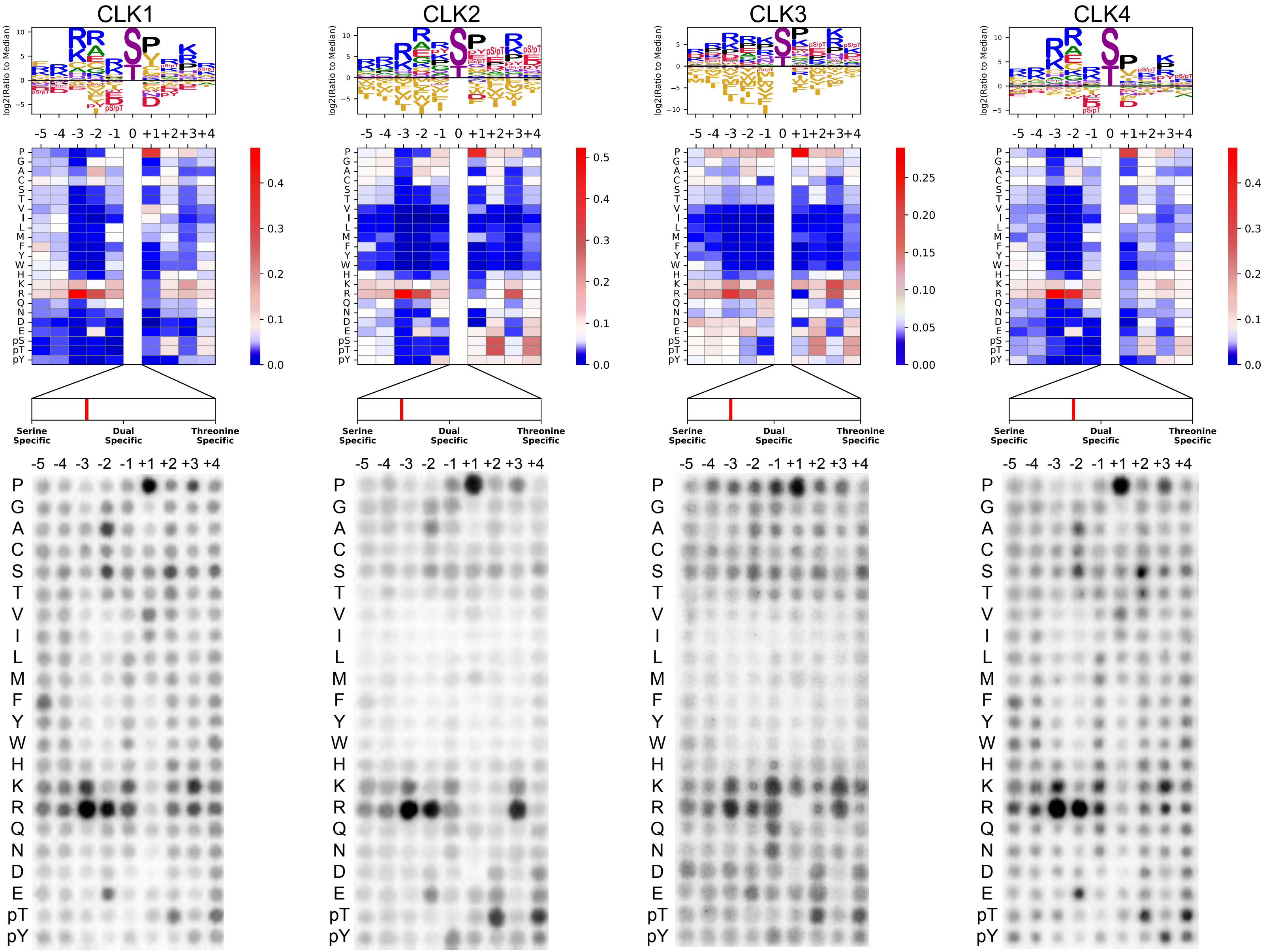
CLK1
CLK2
CLK3
CLK4
log2(Ratio to Median)
-5 -4 -3 -2 -1 0 +1 +2 +3 +4
P G A C S T V I L M F Y W H K R Q N D E pS pT pY
Serine Specific
Dual Specific
Threonine Specific
-5 -4 -3 -2 -1 +1 +2 +3 +4
P G A C S T V I L M F Y W H K R Q N D E pT pY

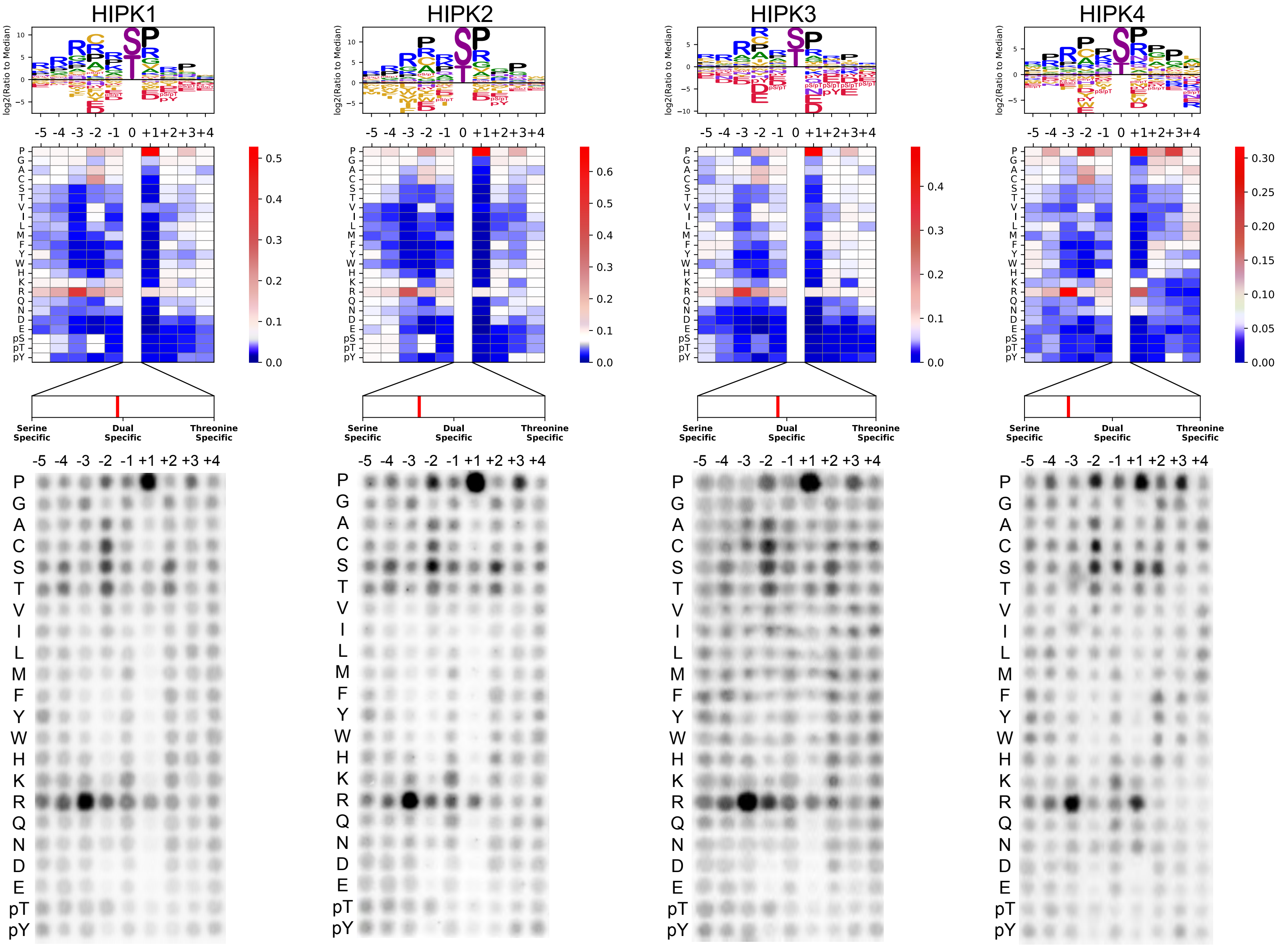

HIPK1
HIPK2
HIPK3
HIPK4
log2(Ratio to Median)
-5 -4 -3 -2 -1 0 +1 +2 +3 +4
P G A C S T V I L M F Y W H K R Q N D E pS pT pY
Serine Specific
Dual Specific
Threonine Specific
-5 -4 -3 -2 -1 +1 +2 +3 +4
P G A C S T V I L M F Y W H K R Q N D E pT pY

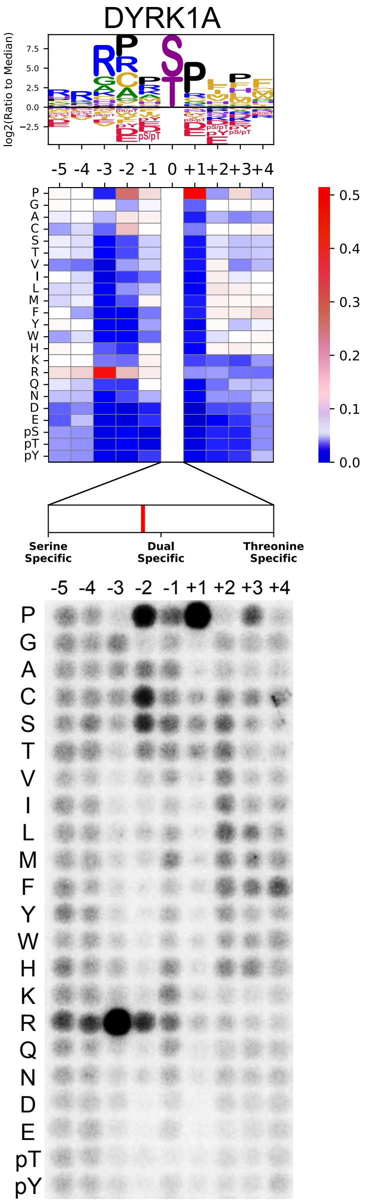
DYRK1A
log2(Ratio to Median)
7.5
5.0
2.5
0.0
-2.5
-5 -4 -3 -2 -1 0 +1 +2 +3 +4
P G A C S T V I L M F Y W H K R Q N D E pS pT pY
0.5
0.4
0.3
0.2
0.1
0.0
Serine Specific
Dual Specific
Threonine Specific
-5 -4 -3 -2 -1 +1 +2 +3 +4
P G A C S T V I L M F Y W H K R Q N D E pT pY

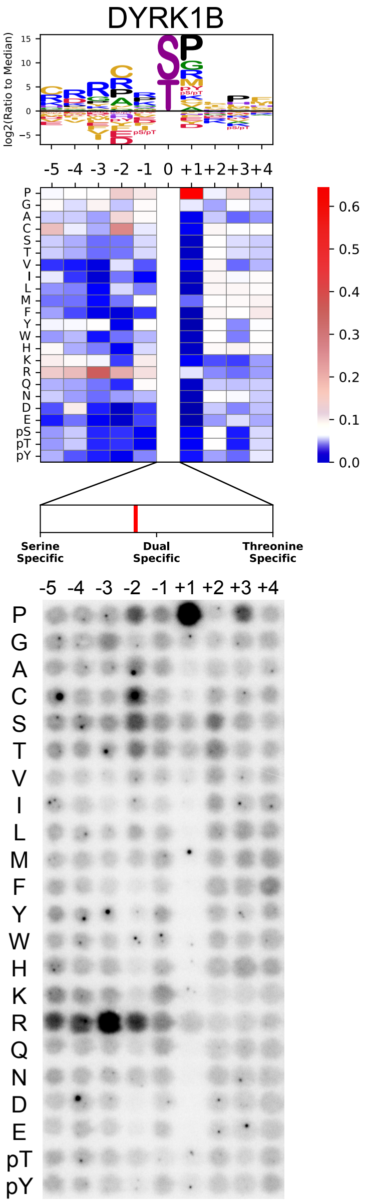
DYRK1B
log2(Ratio to Median)
15
10
5
0
-5
-5 -4 -3 -2 -1 0 +1 +2 +3 +4
P G A C S T V I L M F Y W H K R Q N D E pS pT pY
0.6
0.5
0.4
0.3
0.2
0.1
0.0
Serine Specific
Dual Specific
Threonine Specific
-5 -4 -3 -2 -1 +1 +2 +3 +4
P G A C S T V I L M F Y W H K R Q N D E pT pY

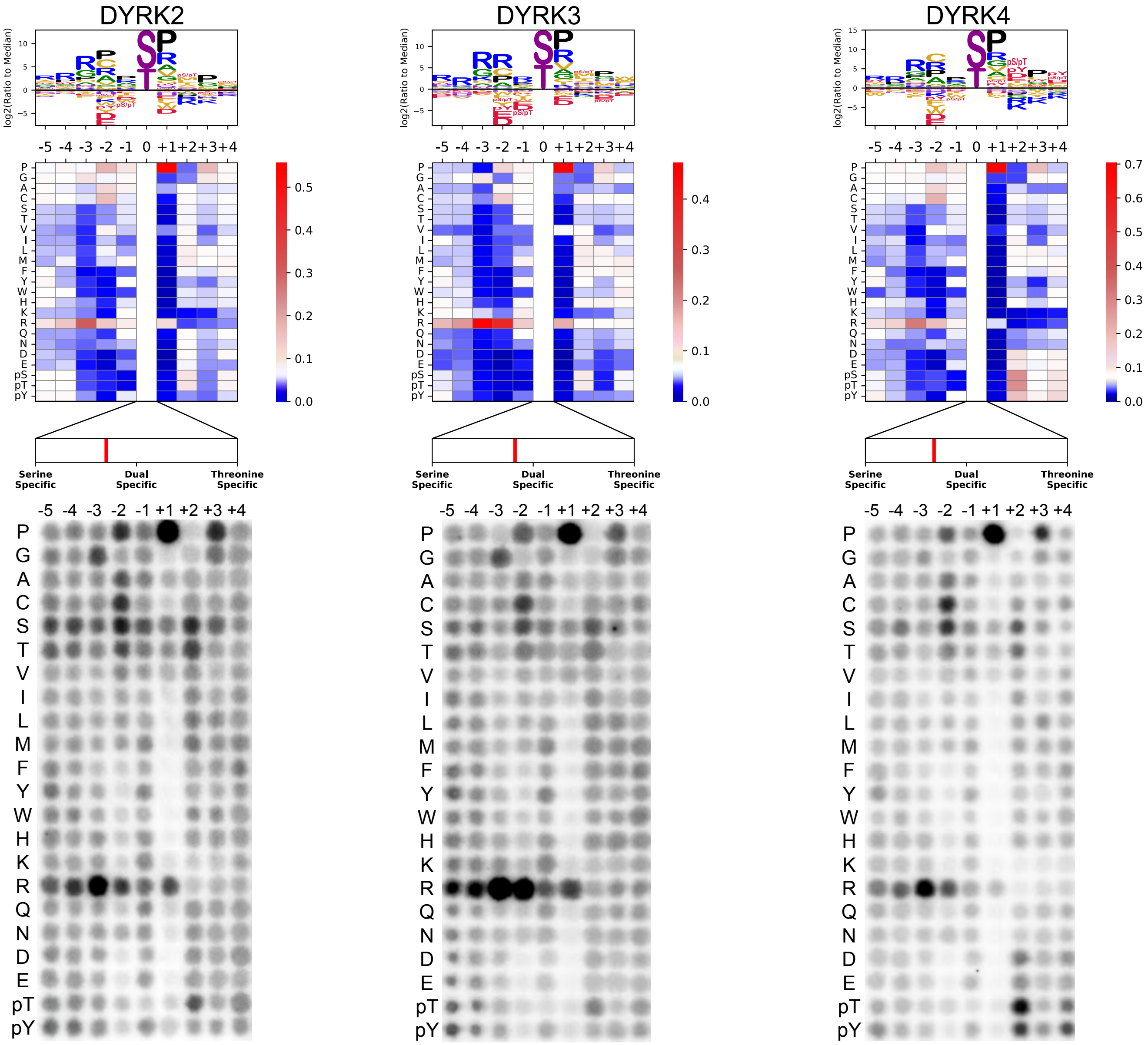
DYRK2
DYRK3
DYRK4
log2(Ratio to Median)
-5 -4 -3 -2 -1 0 +1 +2 +3 +4
P G A C S T V I L M F Y W H K R Q N D E pS pT pY
Serine Specific
Dual Specific
Threonine Specific
-5 -4 -3 -2 -1 +1 +2 +3 +4
P G A C S T V I L M F Y W H K R Q N D E pT pY

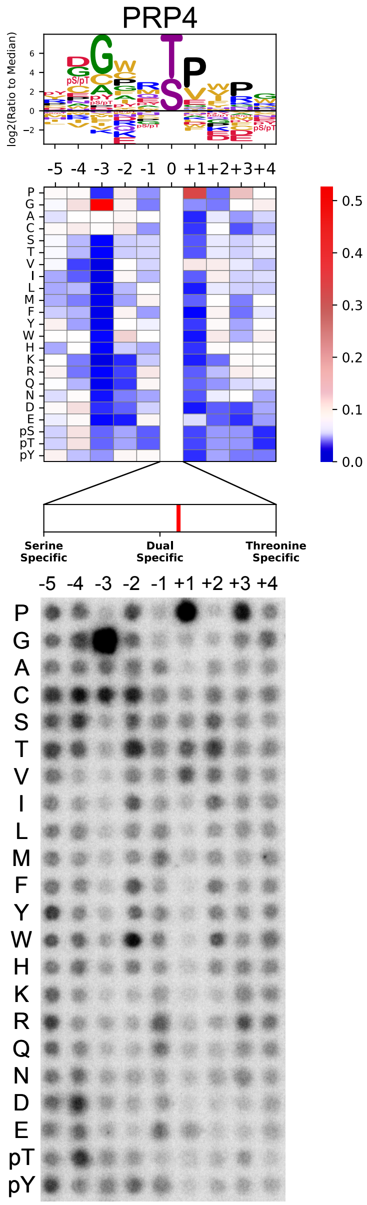

PRP4
log2(Ratio to Median)
-5 -4 -3 -2 -1 0 +1 +2 +3 +4
P G A C S T V I L M F Y W H K R Q N D E pS pT pY
0.5 0.4 0.3 0.2 0.1 0.0
Serine Specific
Dual Specific
Threonine Specific
-5 -4 -3 -2 -1 +1 +2 +3 +4
P G A C S T V I L M F Y W H K R Q N D E pT pY

CK1

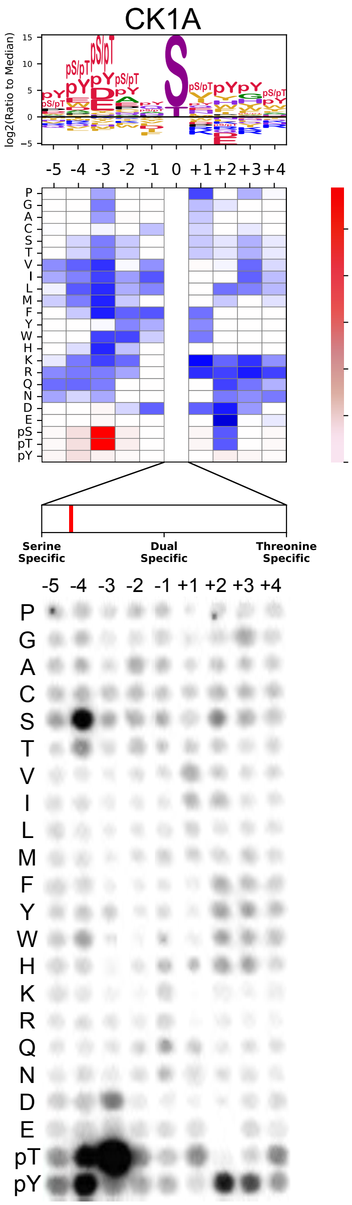
CK1A
log2(Ratio to Median)
-5 -4 -3 -2 -1 0 +1 +2 +3 +4
P G A C S T V I L M F Y W H K R Q N D E pS pT pY
Serine Specific
Dual Specific
Threonine Specific
-5 -4 -3 -2 -1 +1 +2 +3 +4
P G A C S T V I L M F Y W H K R Q N D E pT pY

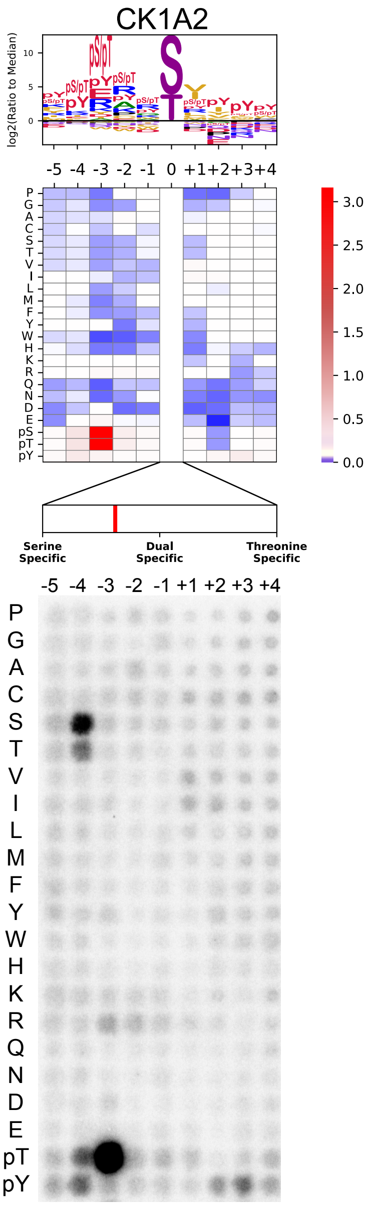
CK1A2
log2(Ratio to Median)
-5 -4 -3 -2 -1 0 +1 +2 +3 +4
P G A C S T V I L M F Y W H K R Q N D E pS pT pY
Serine Specific
Dual Specific
Threonine Specific
-5 -4 -3 -2 -1 +1 +2 +3 +4
P G A C S T V I L M F Y W H K R Q N D E pT pY

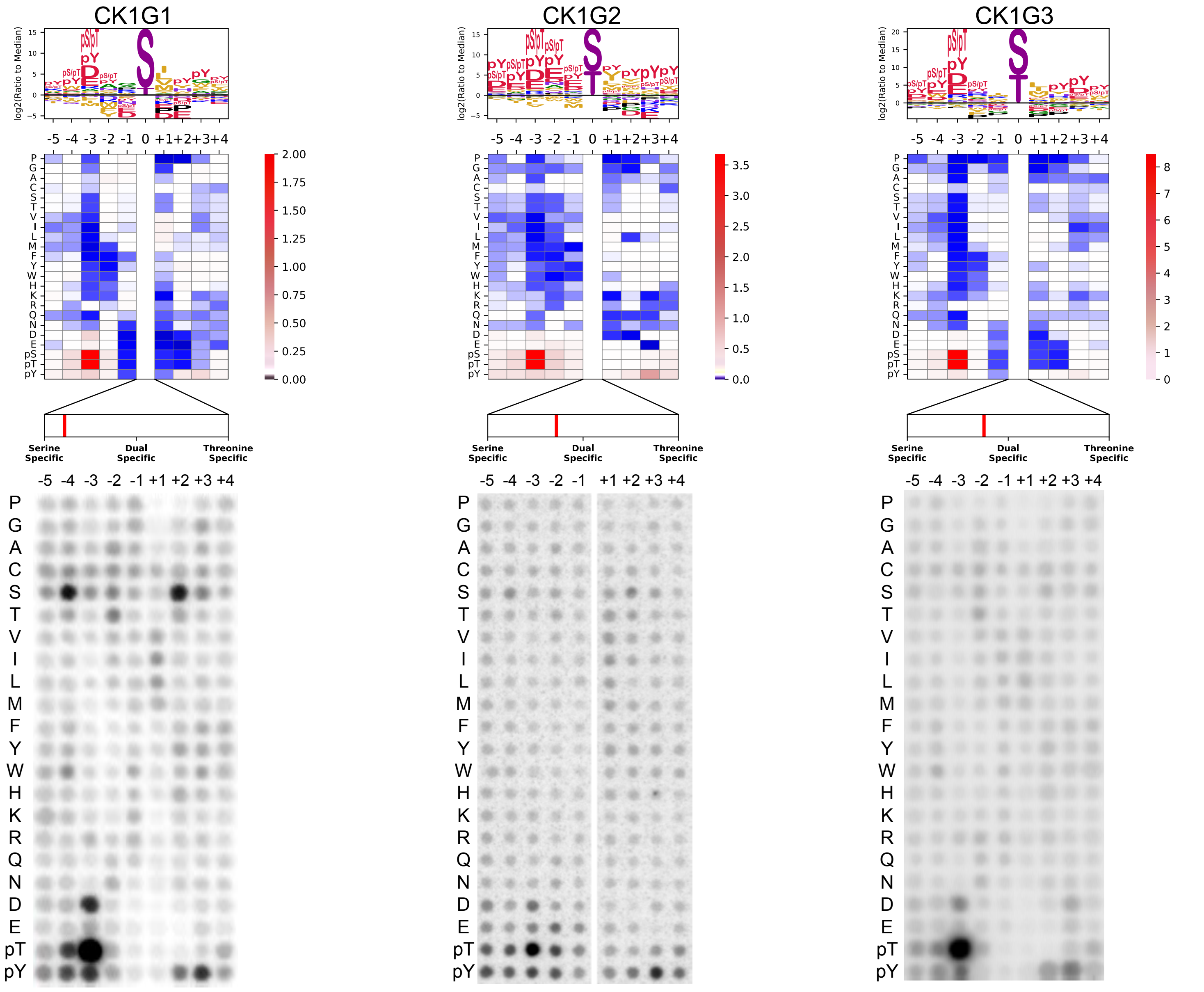

CK1G1
CK1G2
CK1G3
log2(Ratio to Median)
-5 -4 -3 -2 -1 0 +1 +2 +3 +4
P G A C S T V I L M F Y W H K R Q N D E pS pT pY
Serine Specific
Dual Specific
Threonine Specific
P G A C S T V I L M F Y W H K R Q N D E pT pY

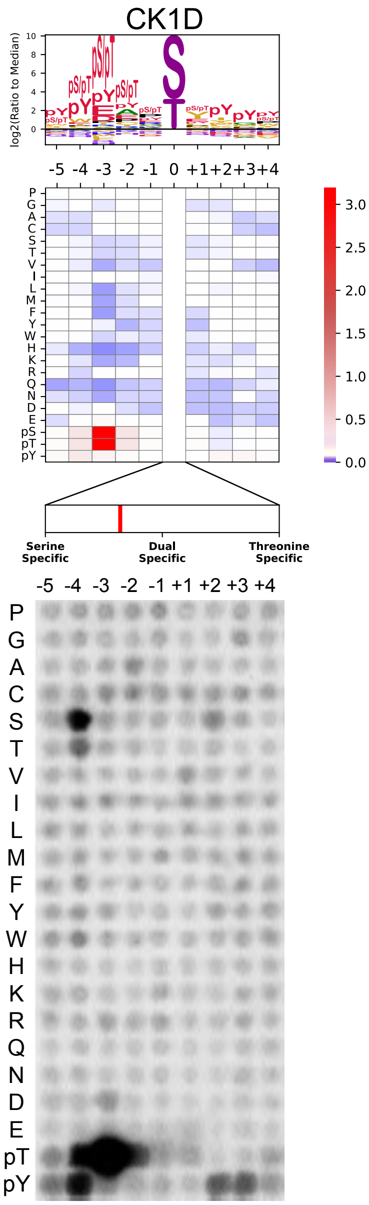
CK1D
log2(Ratio to Median)
-5 -4 -3 -2 -1 0 +1 +2 +3 +4
P G A C S T V I L M F Y W H K R Q N D E pS pT pY
Serine Specific
Dual Specific
Threonine Specific
-5 -4 -3 -2 -1 +1 +2 +3 +4
P G A C S T V I L M F Y W H K R Q N D E pT pY

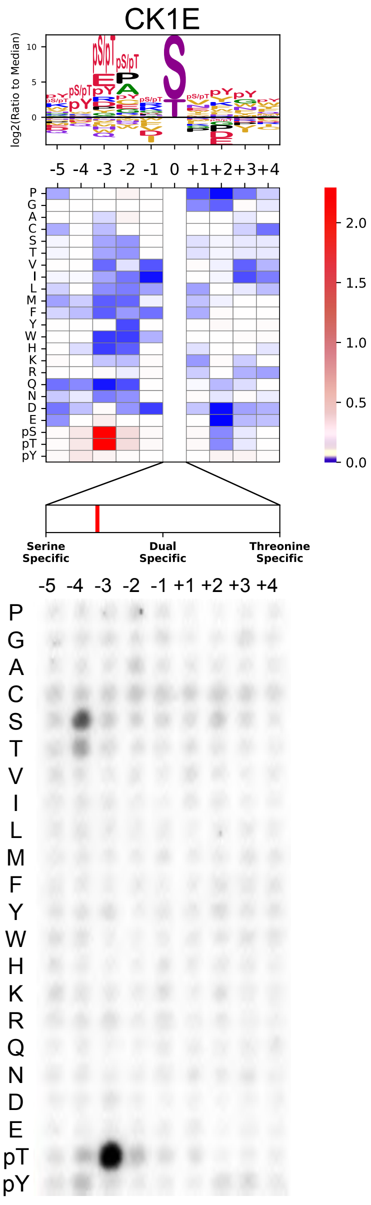
CK1E
log2(Ratio to Median)
-5 -4 -3 -2 -1 0 +1 +2 +3 +4
P G A C S T V I L M F Y W H K R Q N D E pS pT pY
Serine Specific
Dual Specific
Threonine Specific
-5 -4 -3 -2 -1 +1 +2 +3 +4
P G A C S T V I L M F Y W H K R Q N D E pT pY

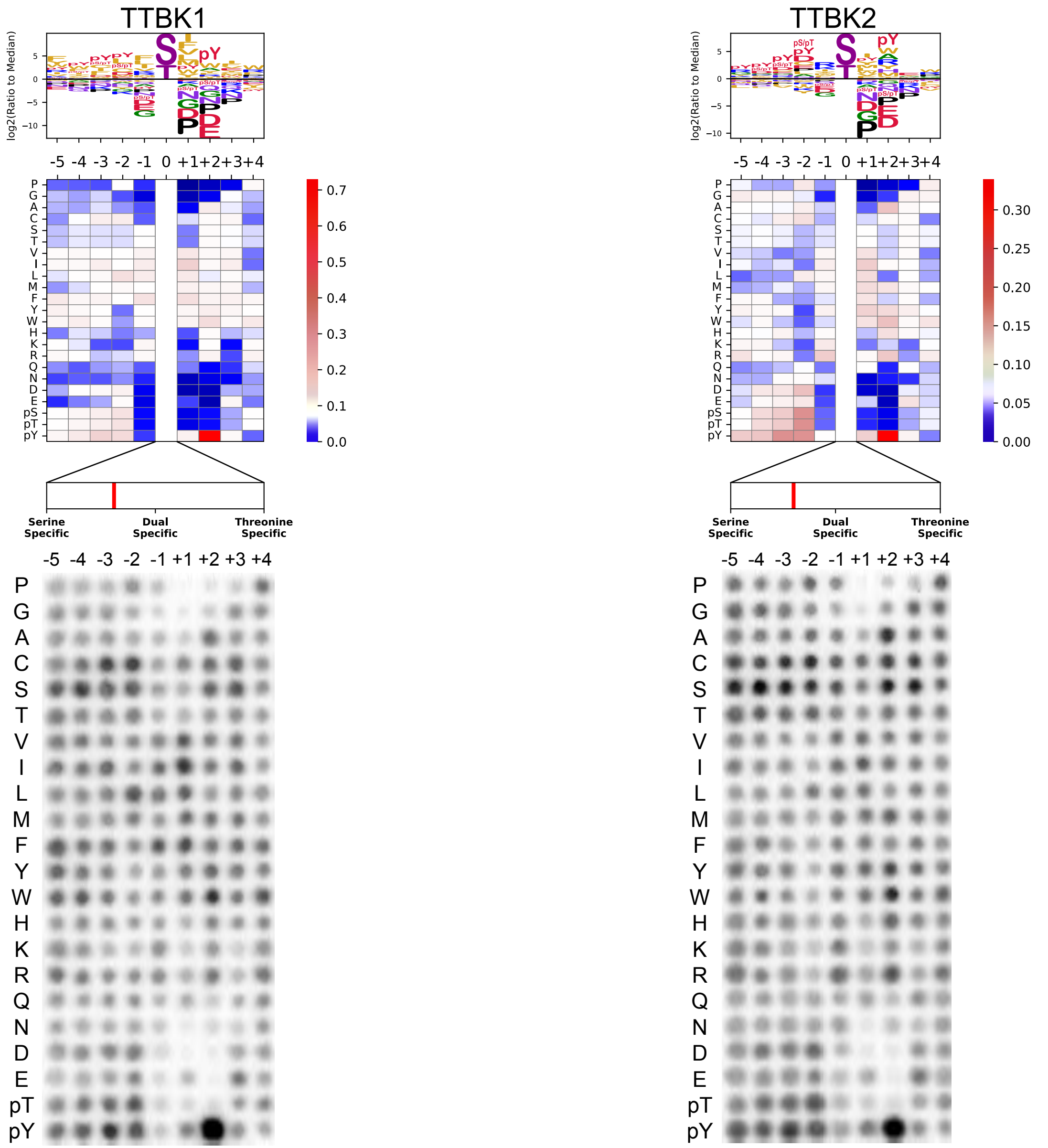
TTBK1
TTBK2
log2(Ratio to Median)
-5 -4 -3 -2 -1 0 +1 +2 +3 +4
P G A C S T V I L M F Y W H K R Q N D E pS pT pY
0.0 0.1 0.2 0.3 0.4 0.5 0.6 0.7
0.00 0.05 0.10 0.15 0.20 0.25 0.30
Serine Specific
Dual Specific
Threonine Specific
-5 -4 -3 -2 -1 +1 +2 +3 +4
P G A C S T V I L M F Y W H K R Q N D E pT pY

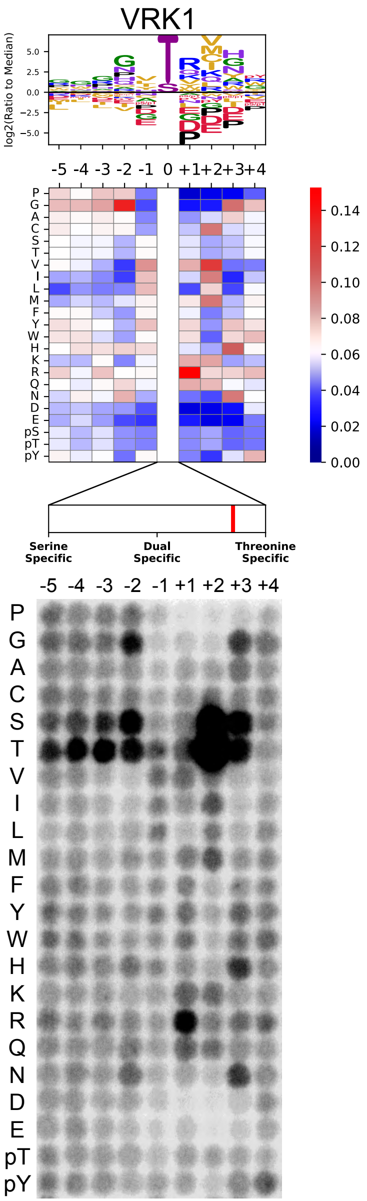
VRK1
log2(Ratio to Median)
5.0
2.5
0.0
−2.5
−5.0
-5 -4 -3 -2 -1 0 +1 +2 +3 +4
P G A C S T V I L M F Y W H K R Q N D E pS pT pY
0.14
0.12
0.10
0.08
0.06
0.04
0.02
0.00
Serine Specific
Dual Specific
Threonine Specific
-5 -4 -3 -2 -1 +1 +2 +3 +4
P G A C S T V I L M F Y W H K R Q N D E pT pY

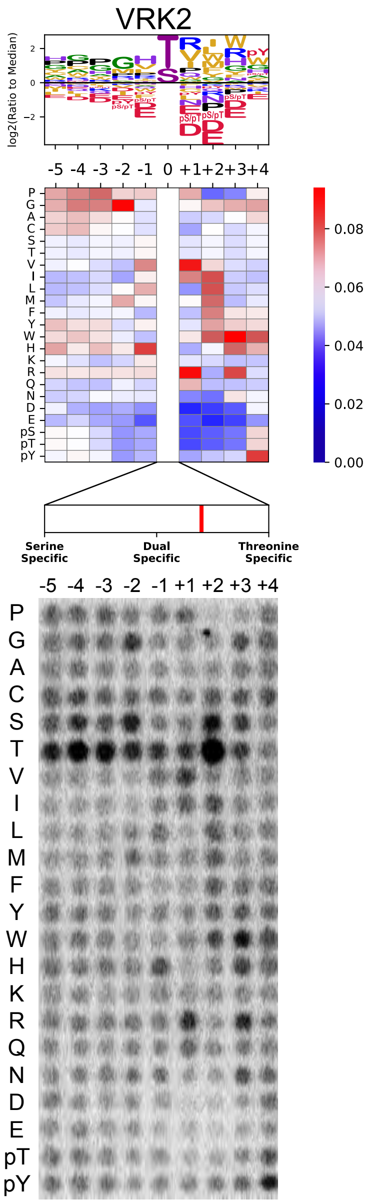
VRK2
log2(Ratio to Median)
2
0
−2
-5 -4 -3 -2 -1 0 +1 +2 +3 +4
P G A C S T V I L M F Y W H K R Q N D E pS pT pY
0.08
0.06
0.04
0.02
0.00
Serine Specific
Dual Specific
Threonine Specific
-5 -4 -3 -2 -1 +1 +2 +3 +4
P G A C S T V I L M F Y W H K R Q N D E pT pY

STE

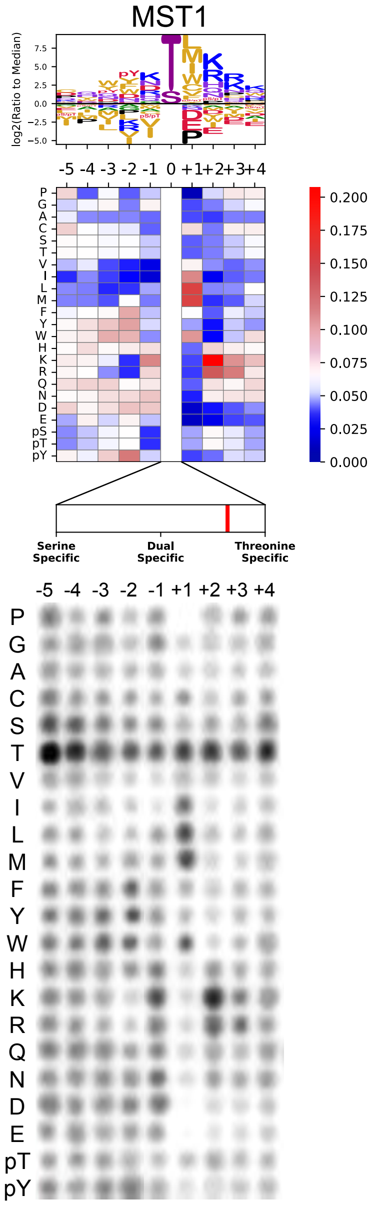
MST1
log2(Ratio to Median)
7.5
5.0
2.5
0.0
−2.5
−5.0
-5 -4 -3 -2 -1 0 +1+2+3+4
P G A C S T V I L M F Y W H K R Q N D E pS pT pY
0.200
0.175
0.150
0.125
0.100
0.075
0.050
0.025
0.000
Serine Specific
Dual Specific
Threonine Specific
-5 -4 -3 -2 -1 +1 +2 +3 +4
P G A C S T V I L M F Y W H K R Q N D E pT pY

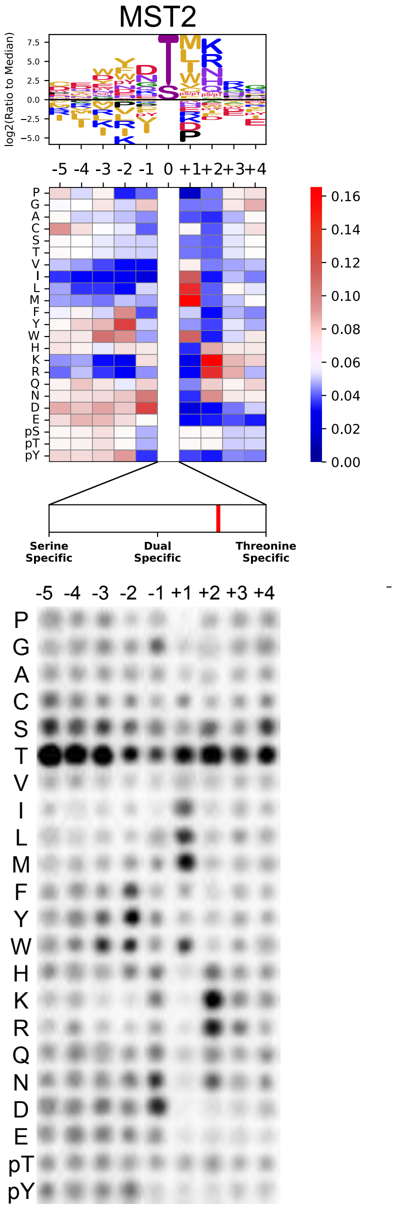
MST2
log2(Ratio to Median)
7.5
5.0
2.5
0.0
−2.5
−5.0
-5 -4 -3 -2 -1 0 +1+2+3+4
P G A C S T V I L M F Y W H K R Q N D E pS pT pY
0.16
0.14
0.12
0.10
0.08
0.06
0.04
0.02
0.00
Serine Specific
Dual Specific
Threonine Specific
-5 -4 -3 -2 -1 +1 +2 +3 +4
P G A C S T V I L M F Y W H K R Q N D E pT pY

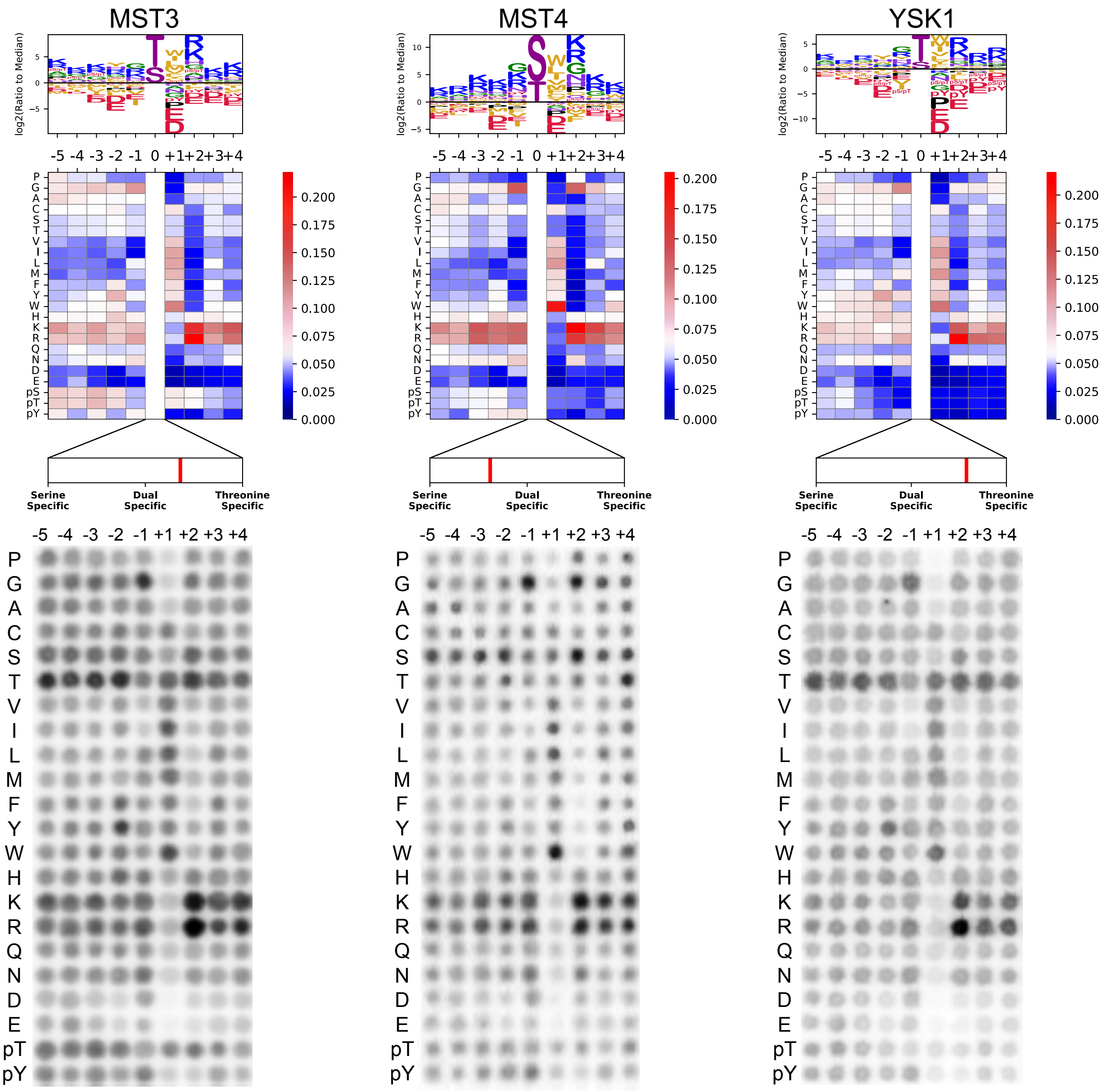

MST3
MST4
YSK1
log2(Ratio to Median)
-5 -4 -3 -2 -1 0 +1 +2 +3 +4
P G A C S T V I L M F Y W H K R Q N D E pS pT pY
0.000 0.025 0.050 0.075 0.100 0.125 0.150 0.175 0.200
Serine Specific
Dual Specific
Threonine Specific
-5 -4 -3 -2 -1 +1 +2 +3 +4
P G A C S T V I L M F Y W H K R Q N D E pT pY

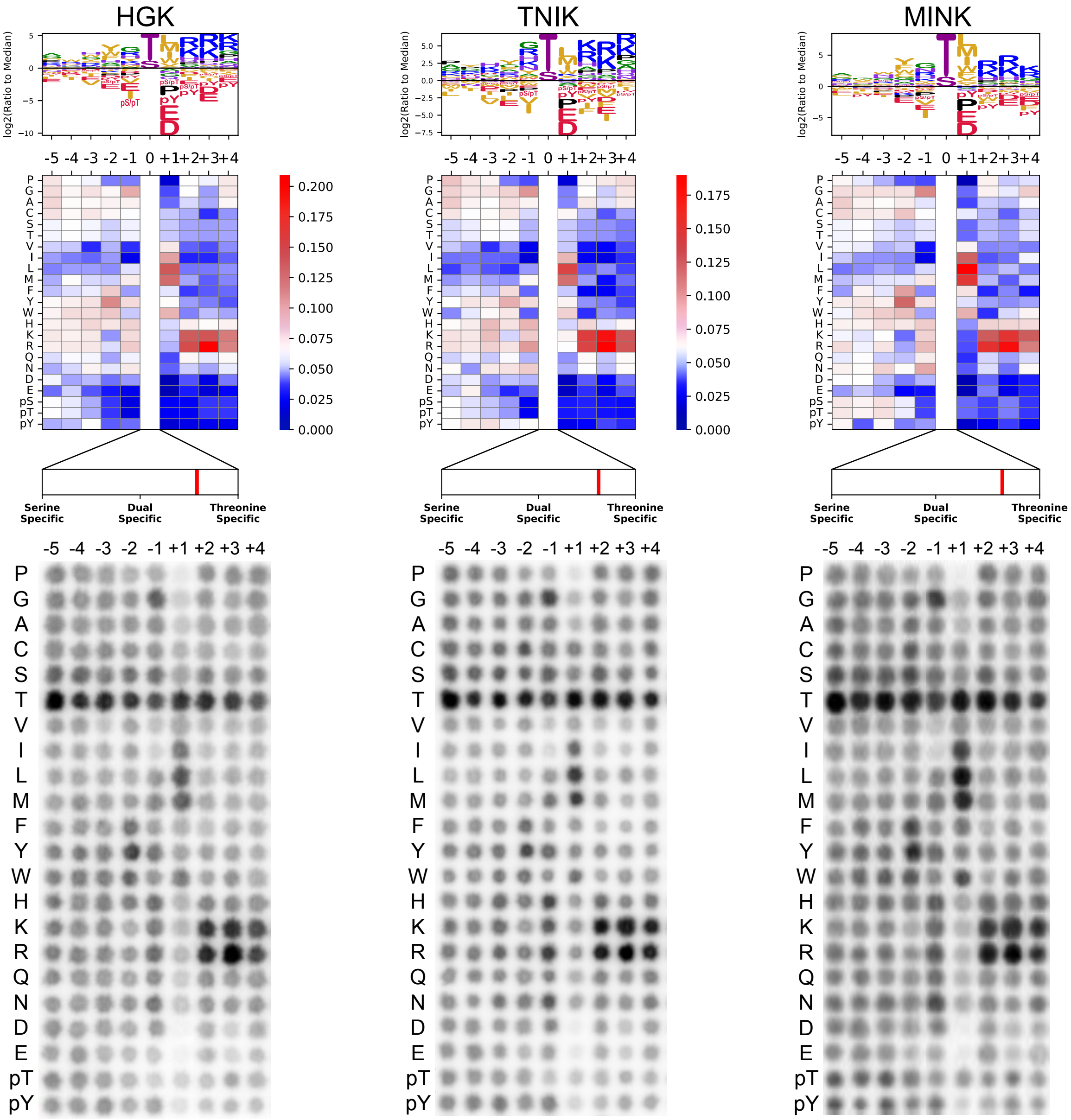

HGK
TNIK
MINK
log2(Ratio to Median)
-5 -4 -3 -2 -1 0 +1 +2 +3 +4
P G A C S T V I L M F Y W H K R Q N D E pS pT pY
0.200 0.175 0.150 0.125 0.100 0.075 0.050 0.025 0.000
0.175 0.150 0.125 0.100 0.075 0.050 0.025 0.000
0.16 0.14 0.12 0.10 0.08 0.06 0.04 0.02 0.00
Serine Specific
Dual Specific
Threonine Specific
-5 -4 -3 -2 -1 +1 +2 +3 +4
P G A C S T V I L M F Y W H K R Q N D E pT pY

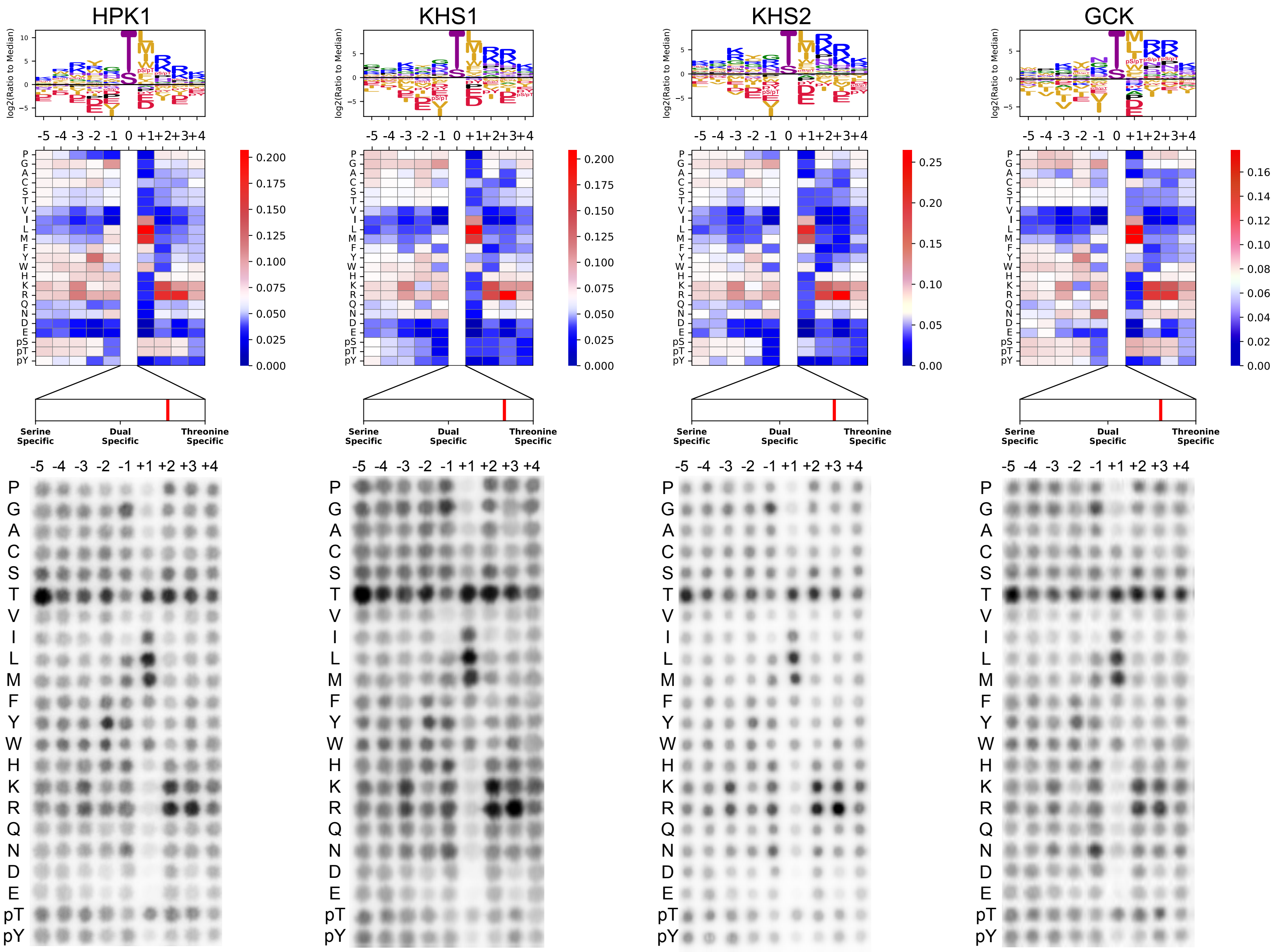

HPK1
KHS1
KHS2
GCK
log2(Ratio to Median)
-5 -4 -3 -2 -1 0 +1 +2 +3 +4
P G A C S T V I L M F Y W H K R Q N D E pS pT pY
Serine Specific
Dual Specific
Threonine Specific
-5 -4 -3 -2 -1 +1 +2 +3 +4
P G A C S T V I L M F Y W H K R Q N D E pT pY

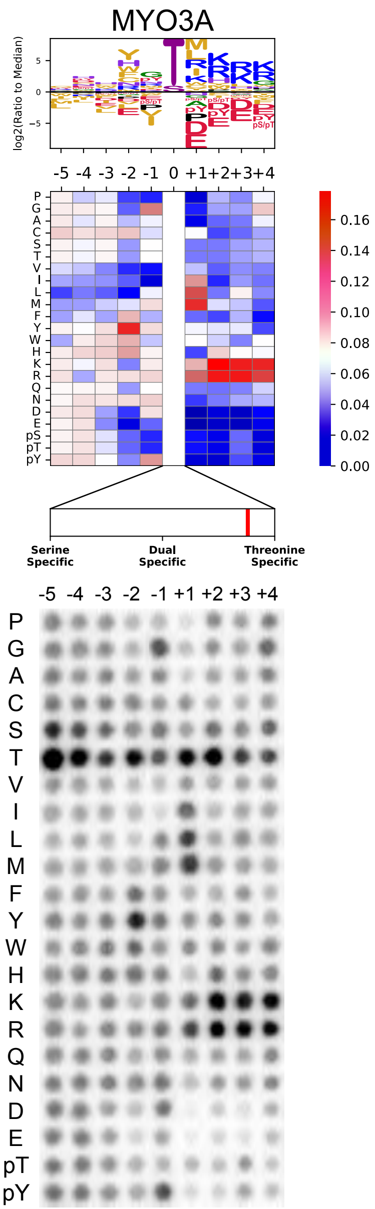
MYO3A
log2(Ratio to Median)
-5 -4 -3 -2 -1 0 +1+2+3+4
P G A C S T V I L M F Y W H K R Q N D E pS pT pY
0.16 0.14 0.12 0.10 0.08 0.06 0.04 0.02 0.00
Serine Specific
Dual Specific
Threonine Specific
-5 -4 -3 -2 -1 +1 +2 +3 +4
P G A C S T V I L M F Y W H K R Q N D E pT pY

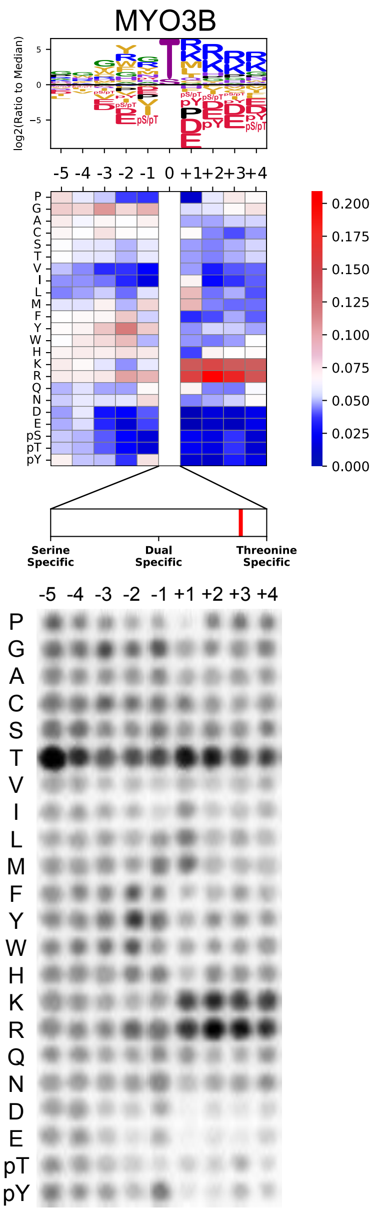
MYO3B
log2(Ratio to Median)
-5 -4 -3 -2 -1 0 +1+2+3+4
P G A C S T V I L M F Y W H K R Q N D E pS pT pY
0.200 0.175 0.150 0.125 0.100 0.075 0.050 0.025 0.000
Serine Specific
Dual Specific
Threonine Specific
-5 -4 -3 -2 -1 +1 +2 +3 +4
P G A C S T V I L M F Y W H K R Q N D E pT pY

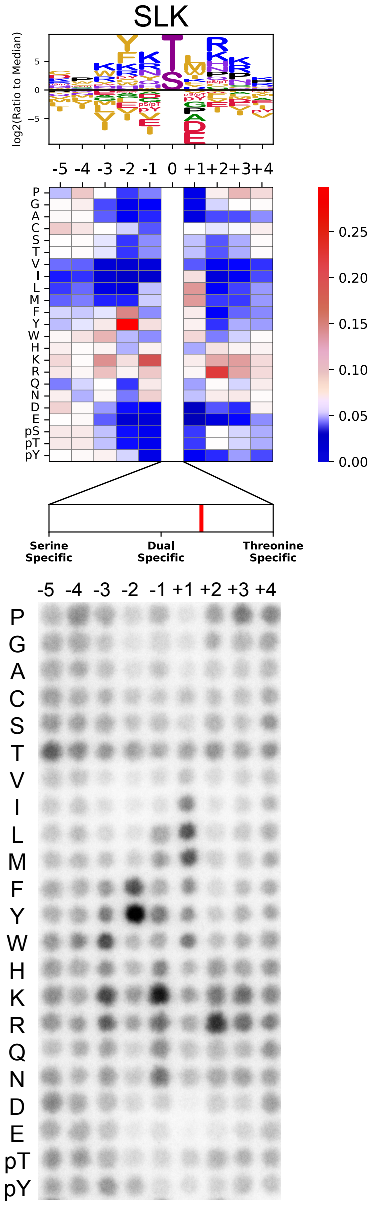
SLK
log2(Ratio to Median)
-5 -4 -3 -2 -1 0 +1 +2 +3 +4
P G A C S T V I L M F Y W H K R Q N D E pS pT pY
0.00 0.05 0.10 0.15 0.20 0.25
Serine Specific
Dual Specific
Threonine Specific
-5 -4 -3 -2 -1 +1 +2 +3 +4
P G A C S T V I L M F Y W H K R Q N D E pT pY

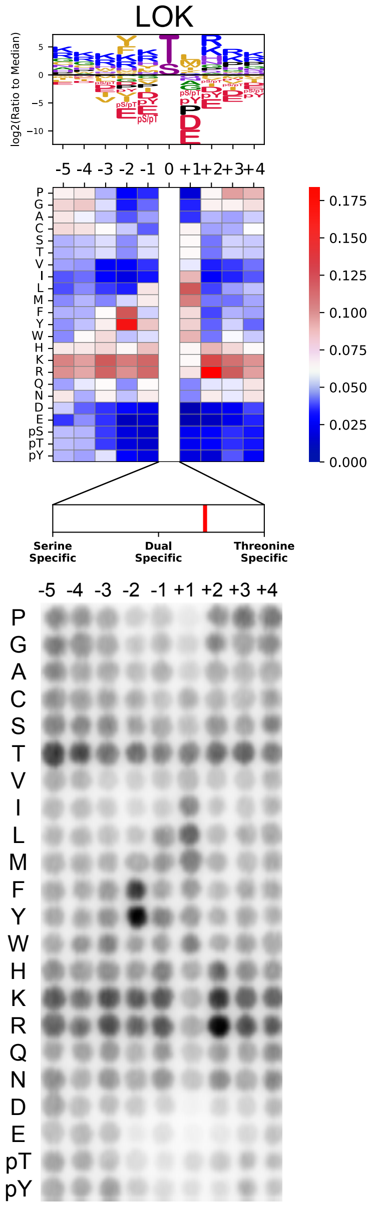
LOK
log2(Ratio to Median)
-5 -4 -3 -2 -1 0 +1 +2 +3 +4
P G A C S T V I L M F Y W H K R Q N D E pS pT pY
0.000 0.025 0.050 0.075 0.100 0.125 0.150 0.175
Serine Specific
Dual Specific
Threonine Specific
-5 -4 -3 -2 -1 +1 +2 +3 +4
P G A C S T V I L M F Y W H K R Q N D E pT pY

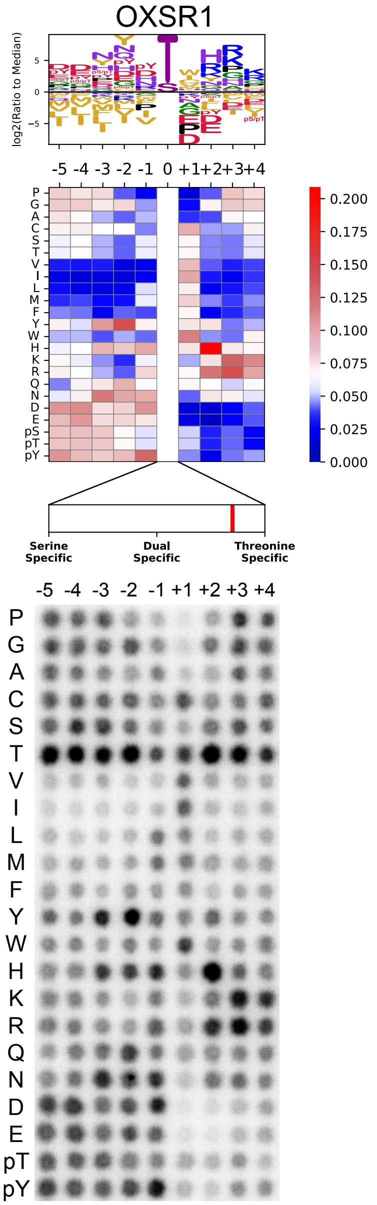
OXSR1
log2(Ratio to Median)
-5 -4 -3 -2 -1 0 +1+2+3+4
P G A C S T V I L M F Y W H K R Q N D E pS pT pY
0.200
0.175
0.150
0.125
0.100
0.075
0.050
0.025
0.000
Serine Specific
Dual Specific
Threonine Specific
-5 -4 -3 -2 -1 +1 +2 +3 +4
P G A C S T V I L M F Y W H K R Q N D E pT pY

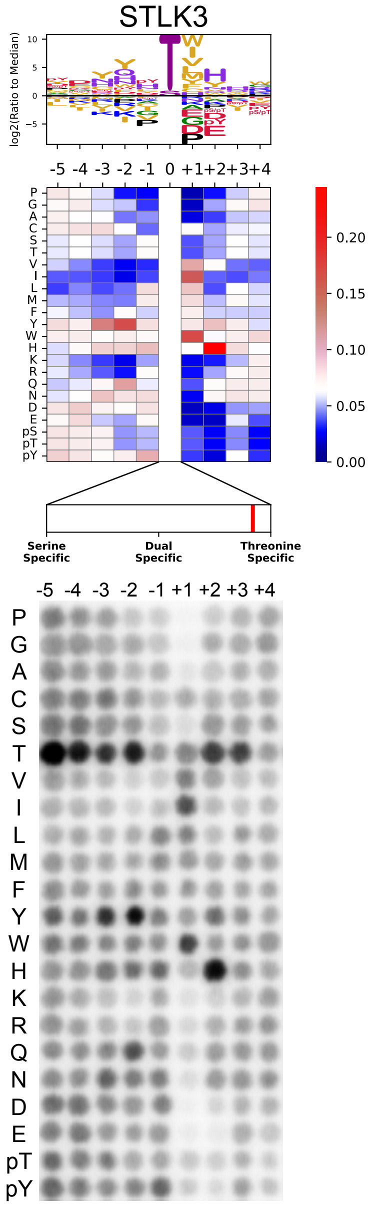
STLK3
log2(Ratio to Median)
-5 -4 -3 -2 -1 0 +1+2+3+4
P G A C S T V I L M F Y W H K R Q N D E pS pT pY
0.20
0.15
0.10
0.05
0.00
Serine Specific
Dual Specific
Threonine Specific
-5 -4 -3 -2 -1 +1 +2 +3 +4
P G A C S T V I L M F Y W H K R Q N D E pT pY

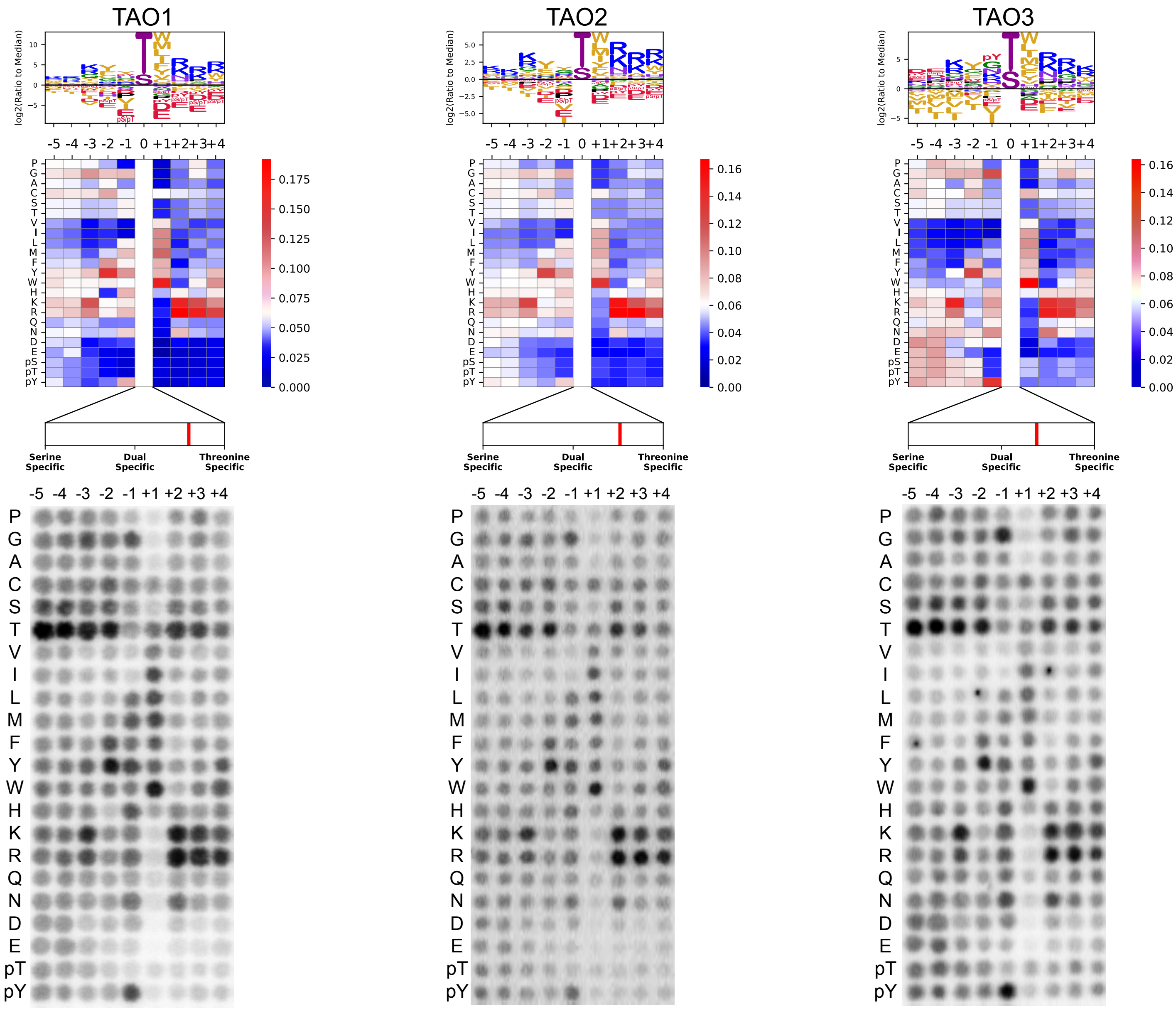
TAO1
TAO2
TAO3
log2(Ratio to Median)
-5 -4 -3 -2 -1 0 +1 +2 +3 +4
P G A C S T V I L M F Y W H K R Q N D E pS pT pY
Serine Specific
Dual Specific
Threonine Specific
-5 -4 -3 -2 -1 +1 +2 +3 +4
P G A C S T V I L M F Y W H K R Q N D E pT pY

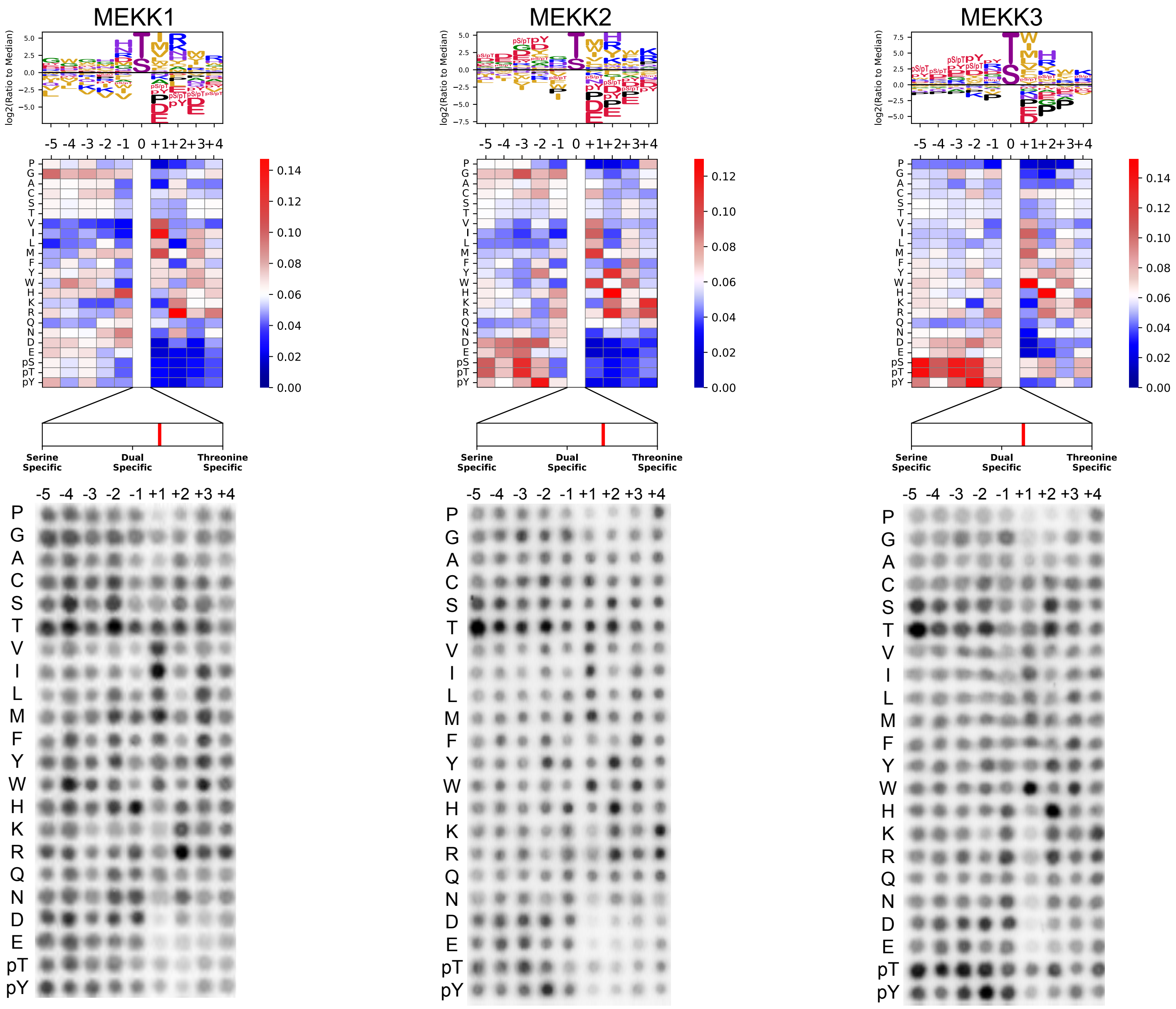
MEKK1
MEKK2
MEKK3
log2(Ratio to Median)
-5 -4 -3 -2 -1 0 +1+2+3+4
P G A C S T V I L M F Y W H K R Q N D E pS pT pY
0.14 0.12 0.10 0.08 0.06 0.04 0.02 0.00
0.12 0.10 0.08 0.06 0.04 0.02 0.00
0.14 0.12 0.10 0.08 0.06 0.04 0.02 0.00
Serine Specific
Dual Specific
Threonine Specific
-5 -4 -3 -2 -1 +1 +2 +3 +4
P G A C S T V I L M F Y W H K R Q N D E pT pY

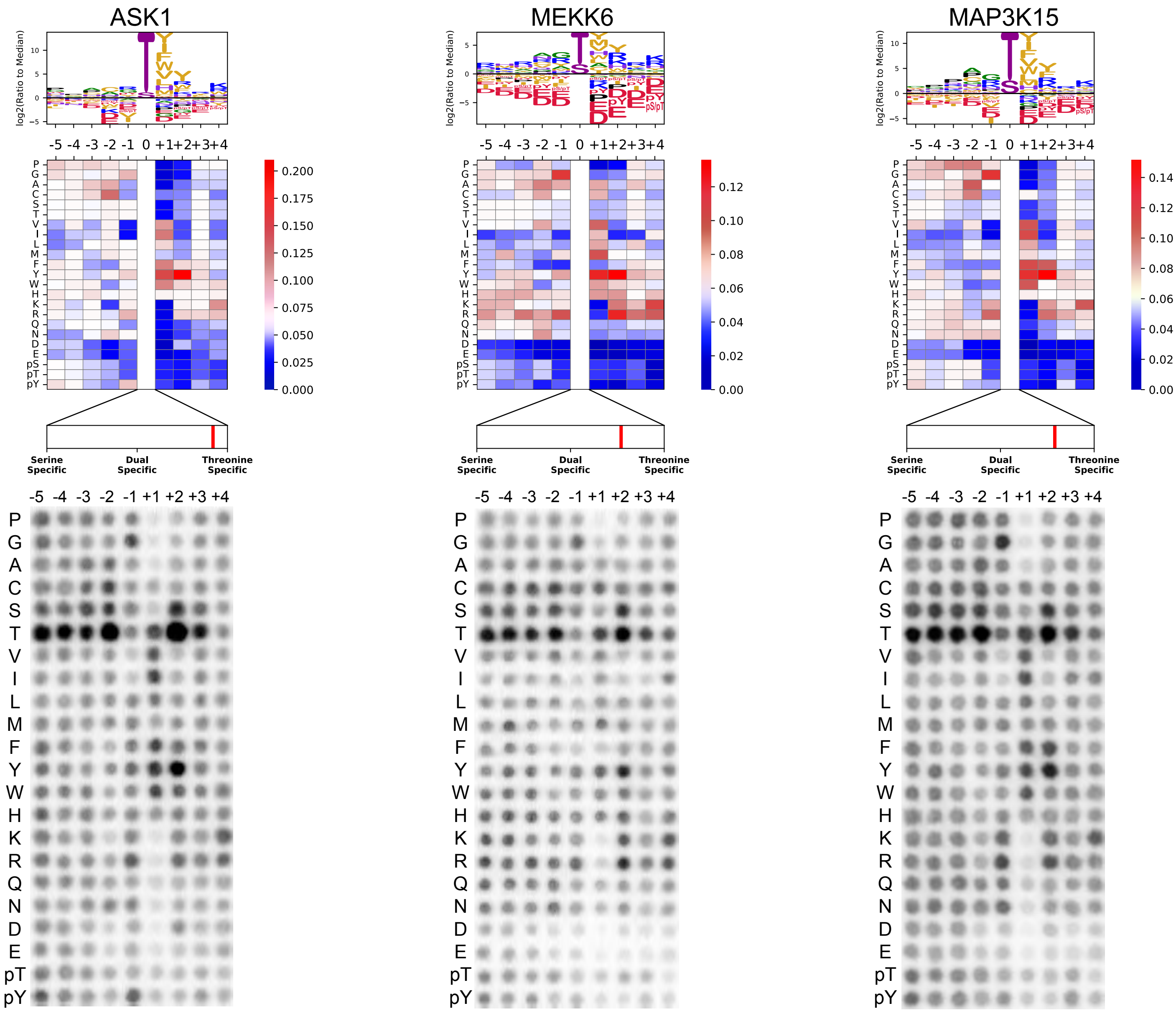
ASK1
MEKK6
MAP3K15
log2(Ratio to Median)
-5 -4 -3 -2 -1 0 +1 +2 +3 +4
P G A C S T V I L M F Y W H K R Q N D E pS pT pY
0.200
0.175
0.150
0.125
0.100
0.075
0.050
0.025
0.000
0.12
0.10
0.08
0.06
0.04
0.02
0.00
0.14
Serine Specific
Dual Specific
Threonine Specific
-5 -4 -3 -2 -1 +1 +2 +3 +4
P G A C S T V I L M F Y W H K R Q N D E pT pY

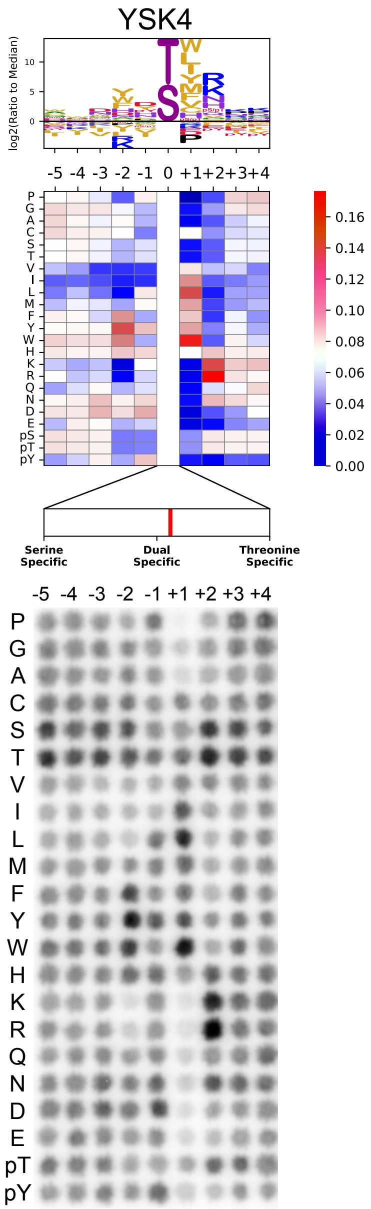
YSK4
log2(Ratio to Median)
-5 -4 -3 -2 -1 0 +1 +2 +3 +4
P G A C S T V I L M F Y W H K R Q N D E pS pT pY
Serine Specific
Dual Specific
Threonine Specific
P G A C S T V I L M F Y W H K R Q N D E pT pY

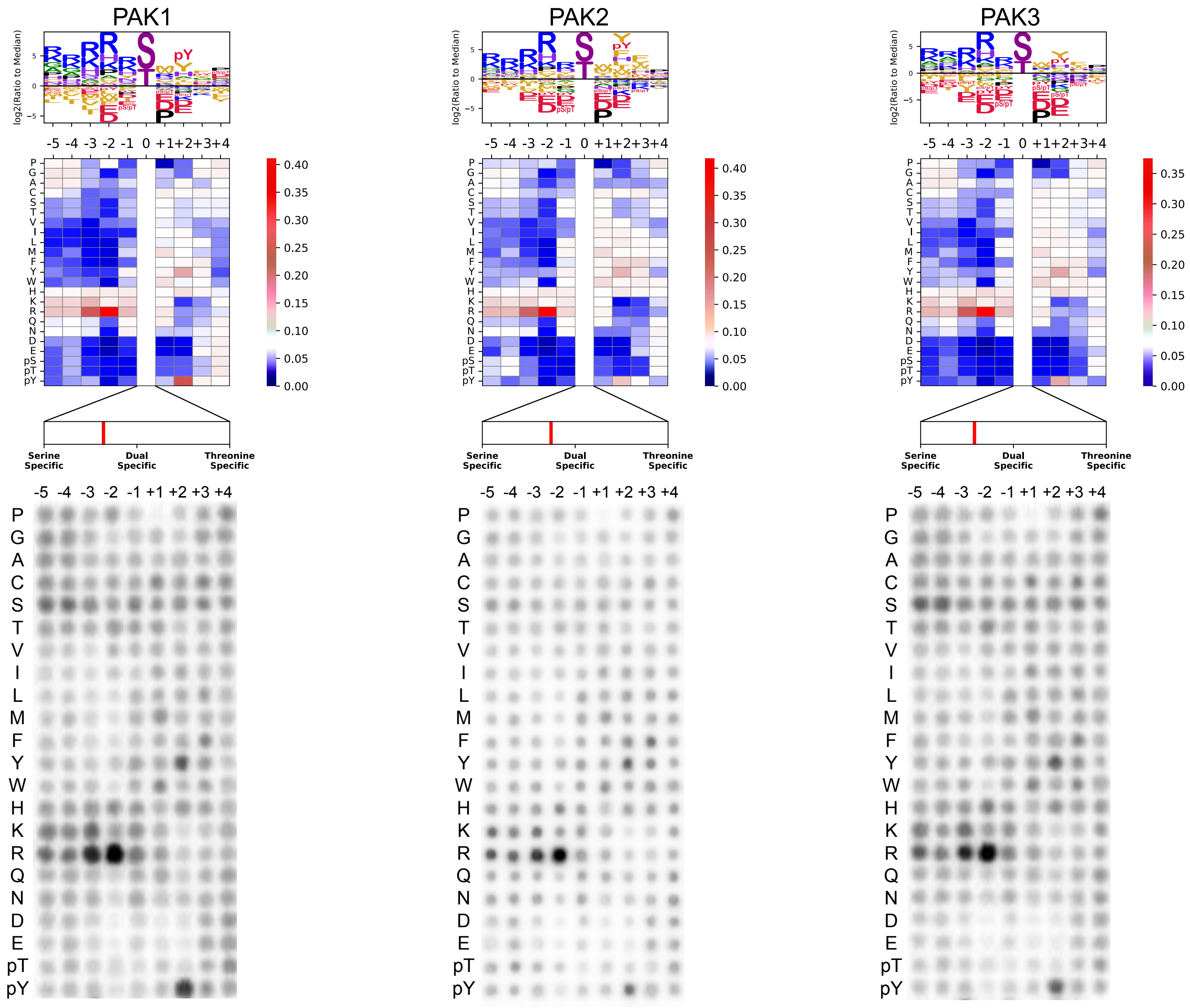

PAK1
PAK2
PAK3
log2(Ratio to Median)
-5 -4 -3 -2 -1 0 +1 +2 +3 +4
P G A C S T V I L M F Y W H K R Q N D E pS pT pY
Serine Specific
Dual Specific
Threonine Specific
-5 -4 -3 -2 -1 +1 +2 +3 +4
P G A C S T V I L M F Y W H K R Q N D E pT pY

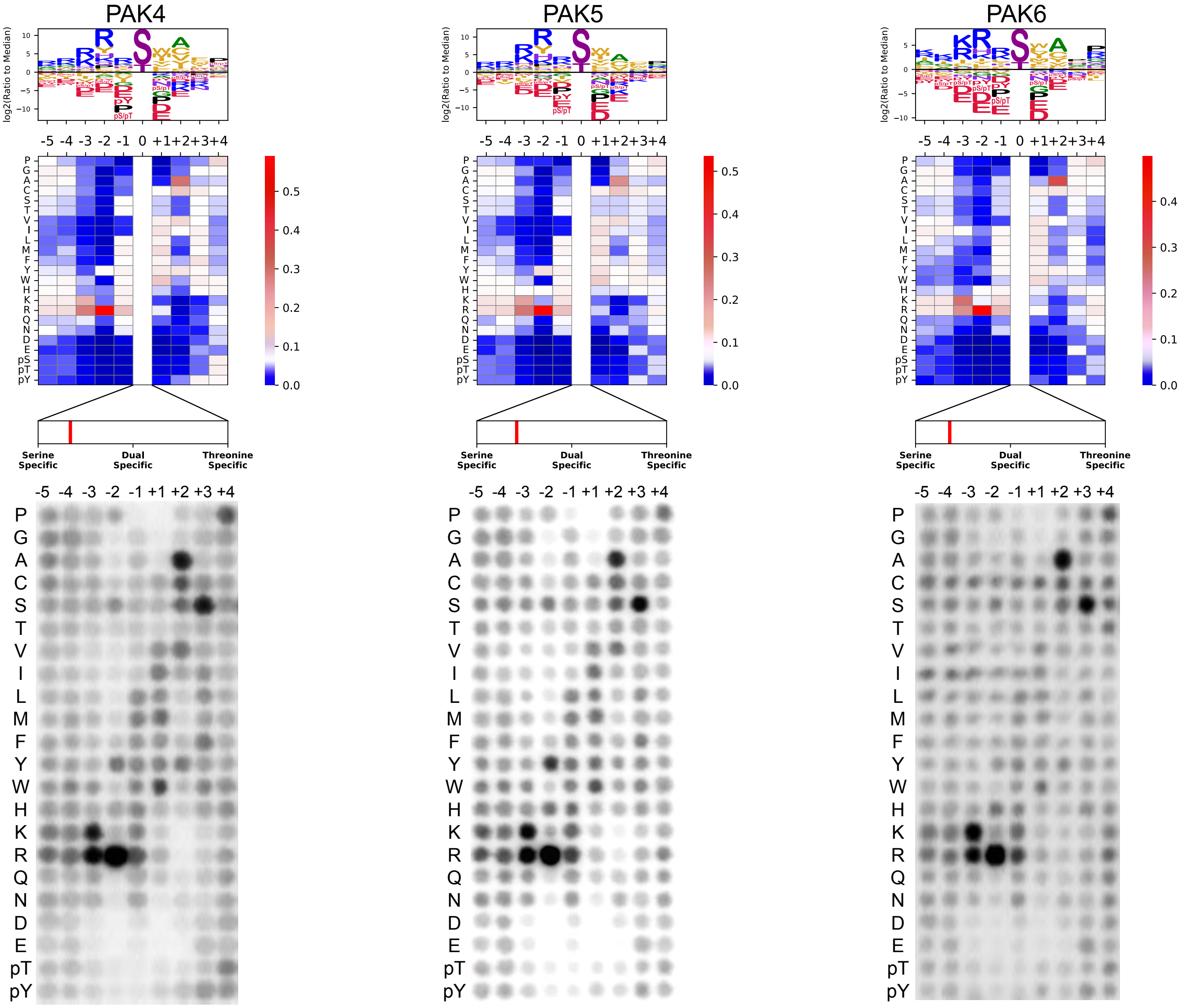
PAK4
PAK5
PAK6
log2(Ratio to Median)
-5 -4 -3 -2 -1 0 +1 +2 +3 +4
P G A C S T V I L M F Y W H K R Q N D E pS pT pY
Serine Specific
Dual Specific
Threonine Specific
-5 -4 -3 -2 -1 +1 +2 +3 +4
P G A C S T V I L M F Y W H K R Q N D E pT pY

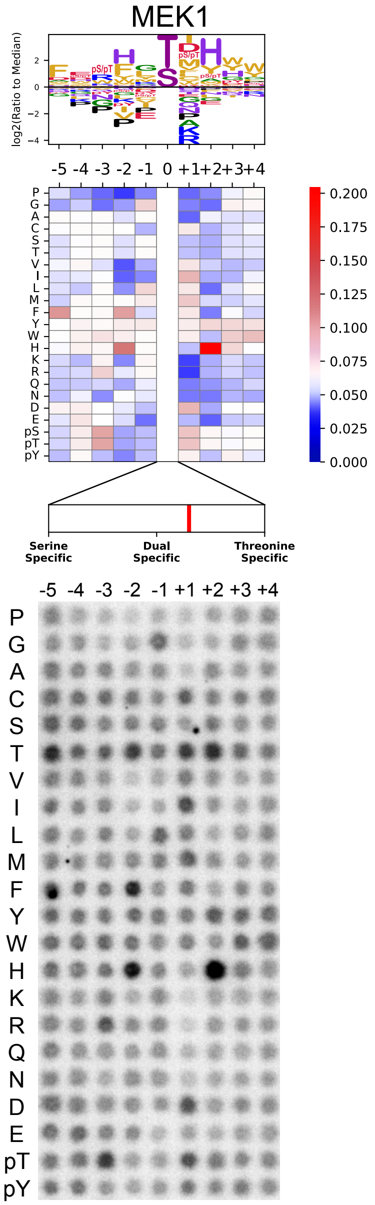
MEK1
log2(Ratio to Median)
-5 -4 -3 -2 -1 0 +1 +2 +3 +4
P G A C S T V I L M F Y W H K R Q N D E pS pT pY
0.200 0.175 0.150 0.125 0.100 0.075 0.050 0.025 0.000
Serine Specific
Dual Specific
Threonine Specific
-5 -4 -3 -2 -1 +1 +2 +3 +4
P G A C S T V I L M F Y W H K R Q N D E pT pY

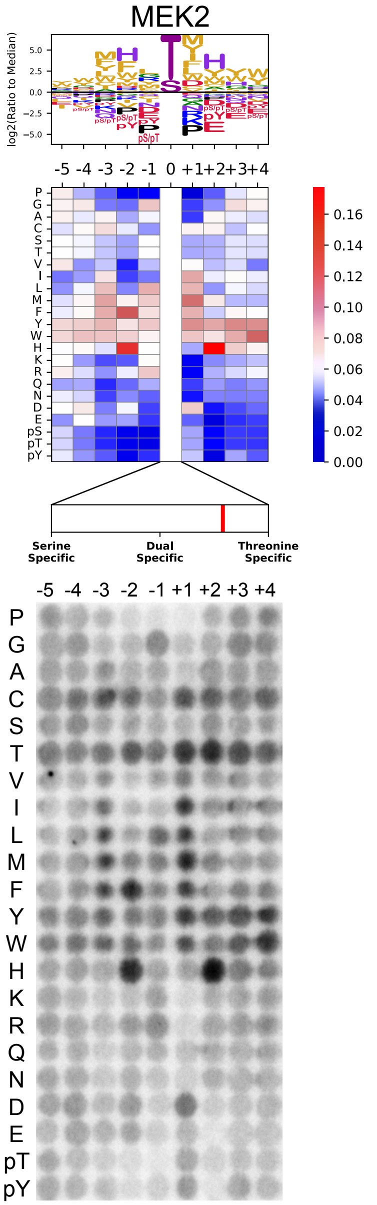
MEK2
log2(Ratio to Median)
-5 -4 -3 -2 -1 0 +1 +2 +3 +4
P G A C S T V I L M F Y W H K R Q N D E pS pT pY
0.16 0.14 0.12 0.10 0.08 0.06 0.04 0.02 0.00
Serine Specific
Dual Specific
Threonine Specific
-5 -4 -3 -2 -1 +1 +2 +3 +4
P G A C S T V I L M F Y W H K R Q N D E pT pY

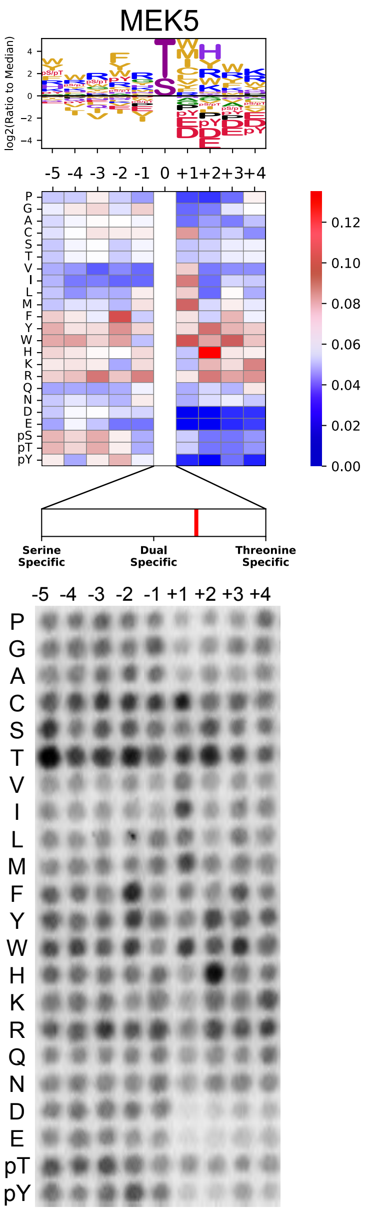
MEK5
log2(Ratio to Median)
-5 -4 -3 -2 -1 0 +1 +2 +3 +4
P G A C S T V I L M F Y W H K R Q N D E pS pT pY
0.12 0.10 0.08 0.06 0.04 0.02 0.00
Serine Specific
Dual Specific
Threonine Specific
-5 -4 -3 -2 -1 +1 +2 +3 +4
P G A C S T V I L M F Y W H K R Q N D E pT pY

COT

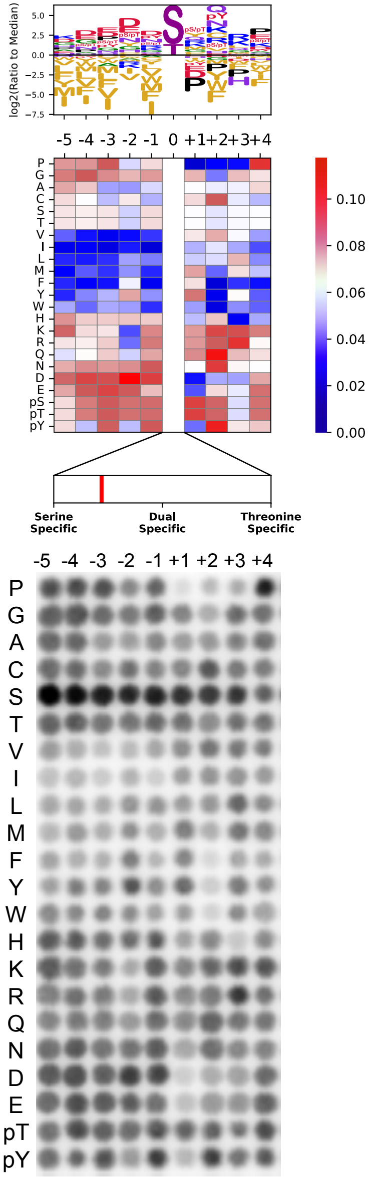
log2(Ratio to Median)
5.0
2.5
0.0
−2.5
−5.0
−7.5
-5 -4 -3 -2 -1 0 +1 +2 +3 +4
P
G
A
C
S
T
V
I
L
M
F
Y
W
H
K
R
Q
N
D
E
pS
pT
pY
0.10
0.08
0.06
0.04
0.02
0.00
Serine Specific
Dual Specific
Threonine Specific
-5 -4 -3 -2 -1 +1 +2 +3 +4
P
G
A
C
S
T
V
I
L
M
F
Y
W
H
K
R
Q
N
D
E
pT
pY

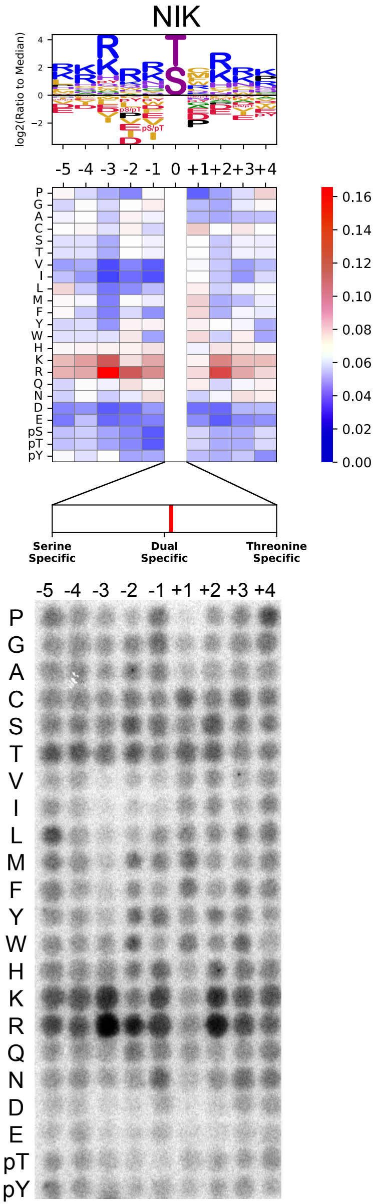
NIK
log2(Ratio to Median)
-5 -4 -3 -2 -1 0 +1 +2 +3 +4
P G A C S T V I L M F Y W H K R Q N D E pS pT pY
0.16 0.14 0.12 0.10 0.08 0.06 0.04 0.02 0.00
Serine Specific
Dual Specific
Threonine Specific
-5 -4 -3 -2 -1 +1 +2 +3 +4
P G A C S T V I L M F Y W H K R Q N D E pT pY

TKL

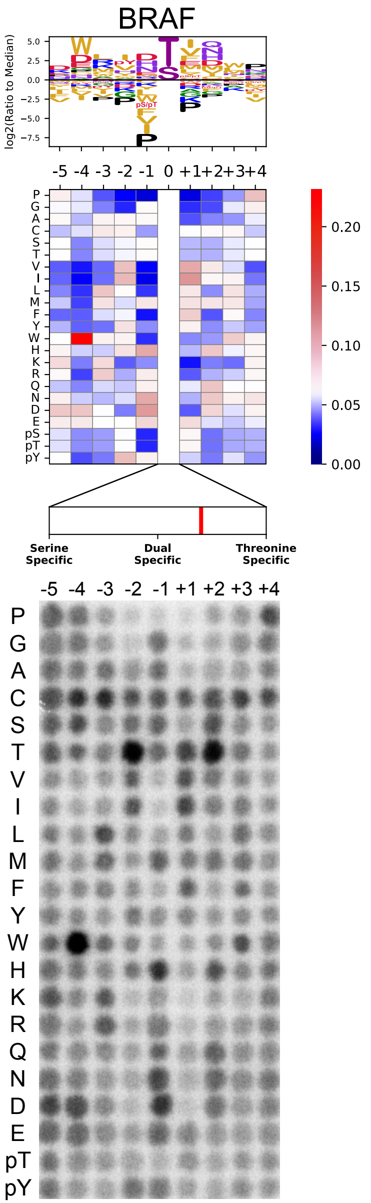
BRAF
log2(Ratio to Median)
5.0
2.5
0.0
−2.5
−5.0
−7.5
-5 -4 -3 -2 -1 0 +1 +2 +3 +4
P G A C S T V I L M F Y W H K R Q N D E pS pT pY
0.20
0.15
0.10
0.05
0.00
Serine Specific
Dual Specific
Threonine Specific
-5 -4 -3 -2 -1 +1 +2 +3 +4
P G A C S T V I L M F Y W H K R Q N D E pT pY

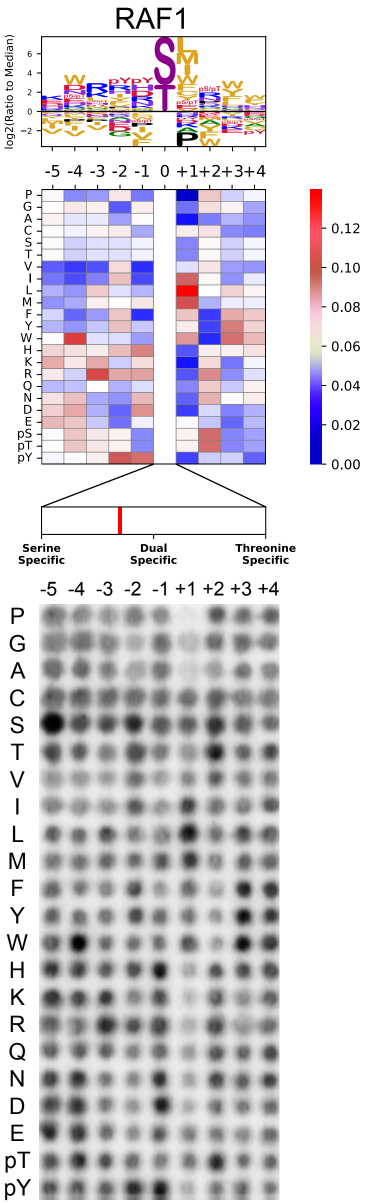
RAF1
log2(Ratio to Median)
6
4
2
0
−2
-5 -4 -3 -2 -1 0 +1 +2 +3 +4
P G A C S T V I L M F Y W H K R Q N D E pS pT pY
0.12
0.10
0.08
0.06
0.04
0.02
0.00
Serine Specific
Dual Specific
Threonine Specific
-5 -4 -3 -2 -1 +1 +2 +3 +4
P G A C S T V I L M F Y W H K R Q N D E pT pY

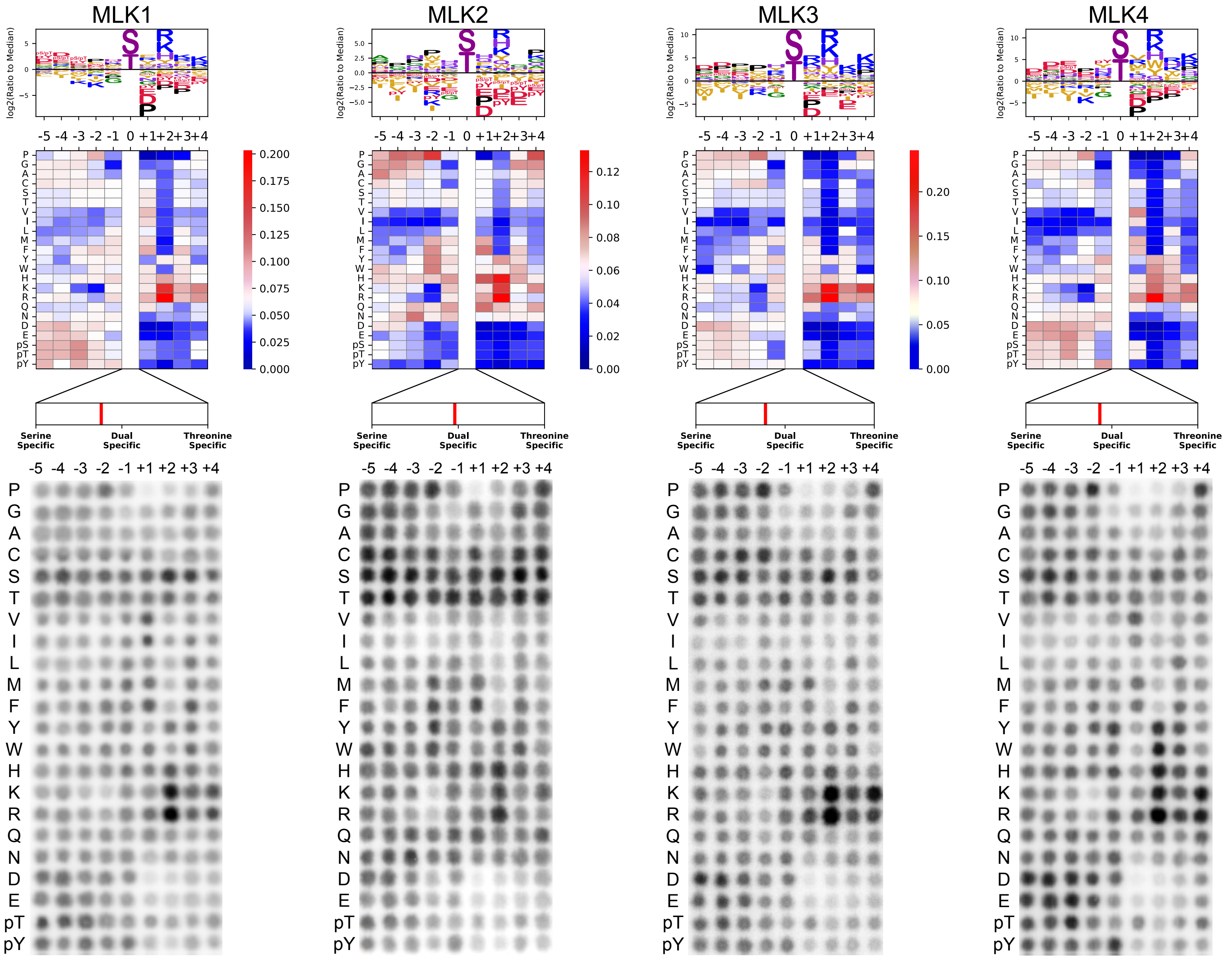
MLK1
MLK2
MLK3
MLK4
log2(Ratio to Median)
-5 -4 -3 -2 -1 0 +1 +2 +3 +4
P G A C S T V I L M F Y W H K R Q N D E pS pT pY
Serine Specific
Dual Specific
Threonine Specific
-5 -4 -3 -2 -1 +1 +2 +3 +4
P G A C S T V I L M F Y W H K R Q N D E pT pY

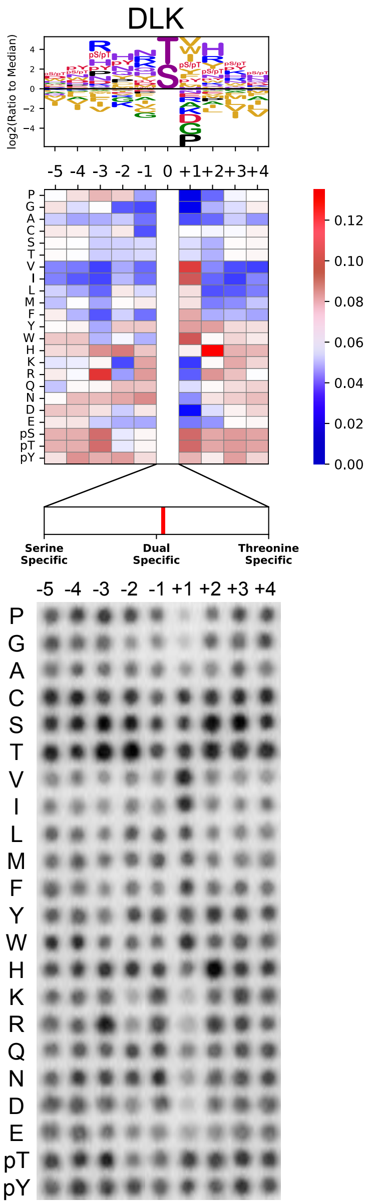
DLK
log2(Ratio to Median)
-5 -4 -3 -2 -1 0 +1 +2 +3 +4
P G A C S T V I L M F Y W H K R Q N D E pS pT pY
0.12 0.10 0.08 0.06 0.04 0.02 0.00
Serine Specific
Dual Specific
Threonine Specific
-5 -4 -3 -2 -1 +1 +2 +3 +4
P G A C S T V I L M F Y W H K R Q N D E pT pY

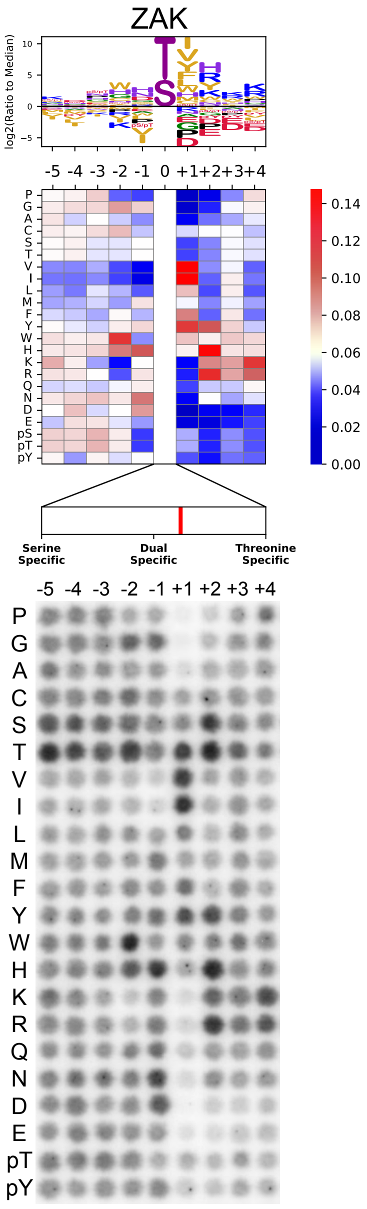
ZAK
log2(Ratio to Median)
-5 -4 -3 -2 -1 0 +1 +2 +3 +4
P G A C S T V I L M F Y W H K R Q N D E pS pT pY
0.14 0.12 0.10 0.08 0.06 0.04 0.02 0.00
Serine Specific
Dual Specific
Threonine Specific
-5 -4 -3 -2 -1 +1 +2 +3 +4
P G A C S T V I L M F Y W H K R Q N D E pT pY

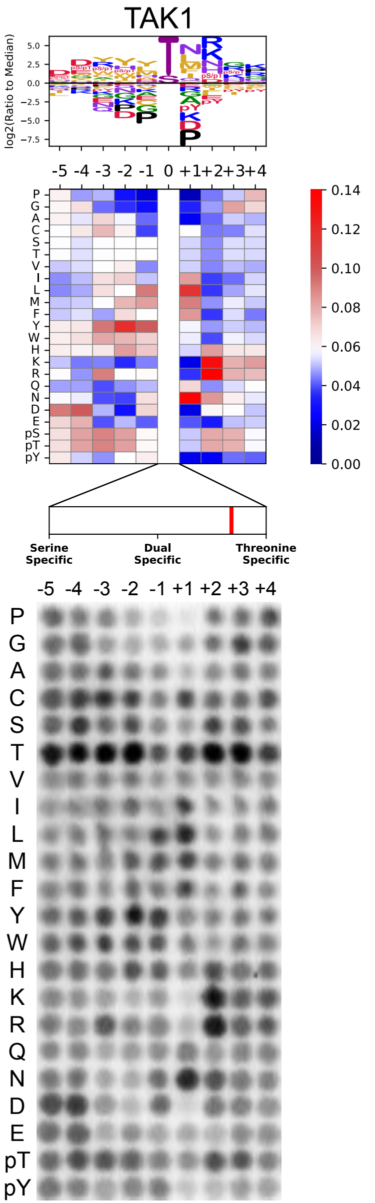

TAK1
log2(Ratio to Median)
-5 -4 -3 -2 -1 0 +1 +2 +3 +4
Serine Specific
Dual Specific
Threonine Specific
-5 -4 -3 -2 -1 +1 +2 +3 +4
P
G
A
C
S
T
V
I
L
M
F
Y
W
H
K
R
Q
N
D
E
pT
pY

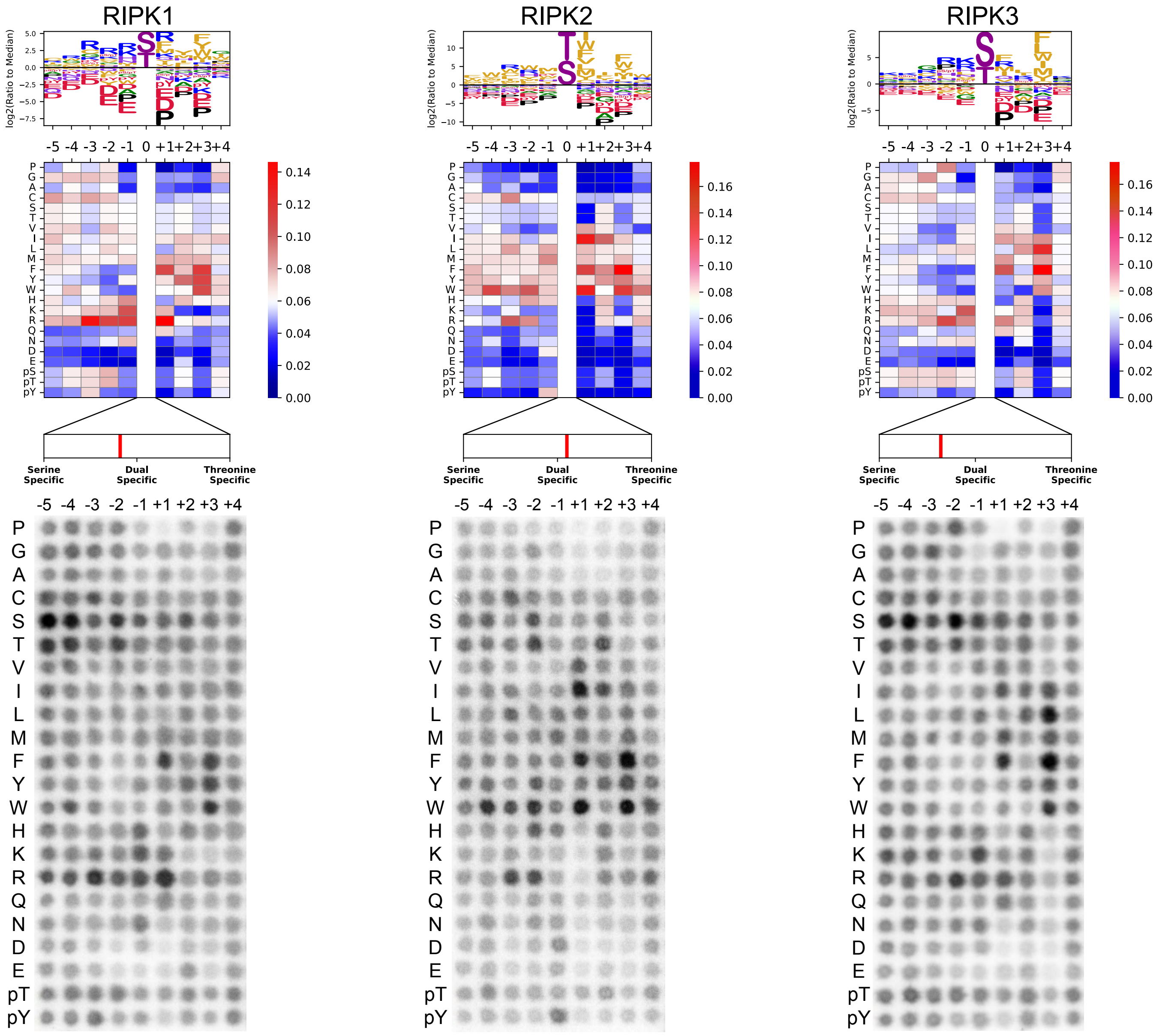
RIPK1
RIPK2
RIPK3
log2(Ratio to Median)
-5 -4 -3 -2 -1 0 +1 +2 +3 +4
P G A C S T V I L M F Y W H K R Q N D E pS pT pY
Serine Specific
Dual Specific
Threonine Specific
-5 -4 -3 -2 -1 +1 +2 +3 +4
P G A C S T V I L M F Y W H K R Q N D E pT pY

ANKRD3

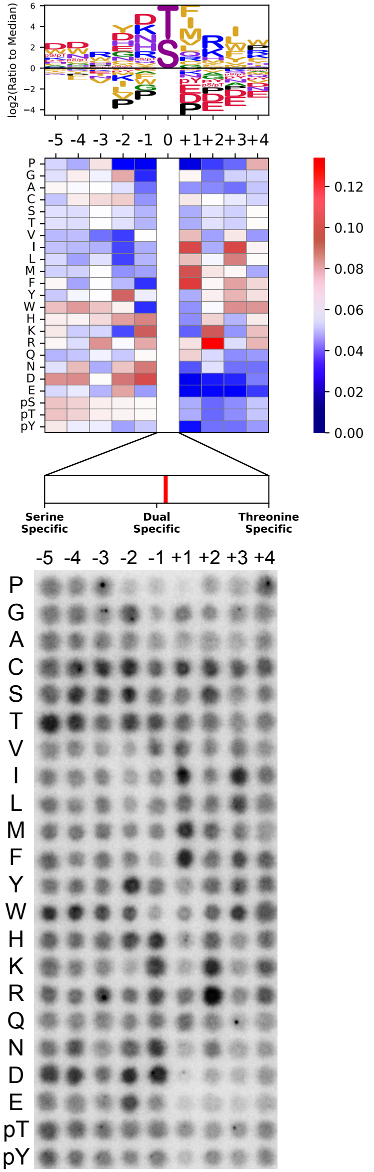
log2(Ratio to Median)
-5 -4 -3 -2 -1 0 +1 +2 +3 +4
P G A C S T V I L M F Y W H K R Q N D E pS pT pY
0.12
0.10
0.08
0.06
0.04
0.02
0.00
Serine Specific
Dual Specific
Threonine Specific
-5 -4 -3 -2 -1 +1 +2 +3 +4
P G A C S T V I L M F Y W H K R Q N D E pT pY

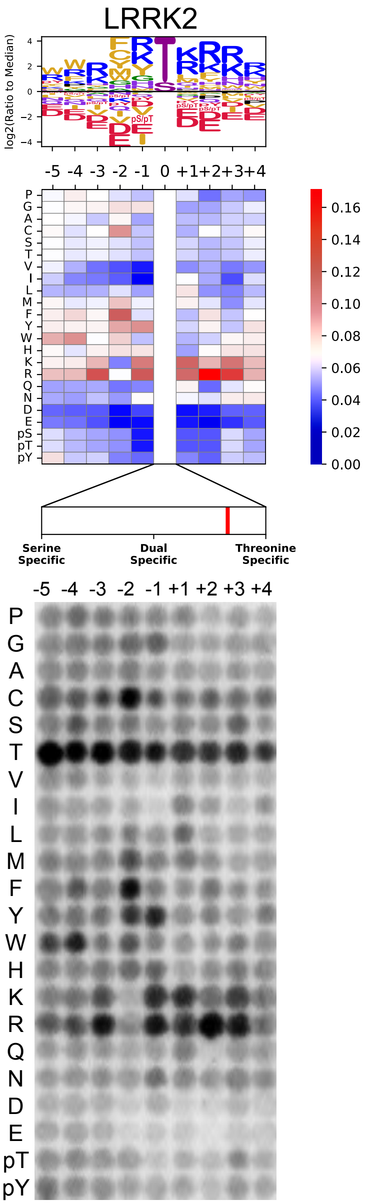
LRRK2
log2(Ratio to Median)
-5 -4 -3 -2 -1 0 +1 +2 +3 +4
P G A C S T V I L M F Y W H K R Q N D E pS pT pY
0.16 0.14 0.12 0.10 0.08 0.06 0.04 0.02 0.00
Serine Specific
Dual Specific
Threonine Specific
-5 -4 -3 -2 -1 +1 +2 +3 +4
P G A C S T V I L M F Y W H K R Q N D E pT pY

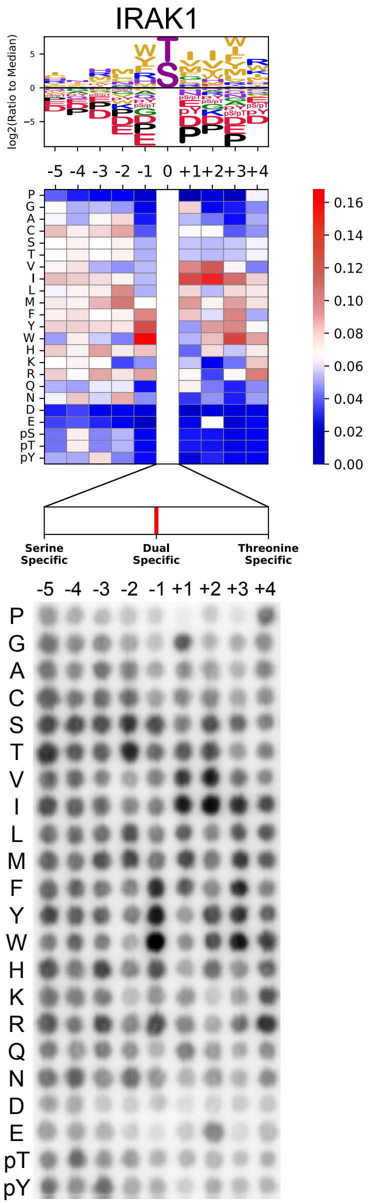
IRAK1
log2(Ratio to Median)
-5 -4 -3 -2 -1 0 +1 +2 +3 +4
P G A C S T V I L M F Y W H K R Q N D E pS pT pY
0.16 0.14 0.12 0.10 0.08 0.06 0.04 0.02 0.00
Serine Specific
Dual Specific
Threonine Specific
-5 -4 -3 -2 -1 +1 +2 +3 +4
P G A C S T V I L M F Y W H K R Q N D E pT pY

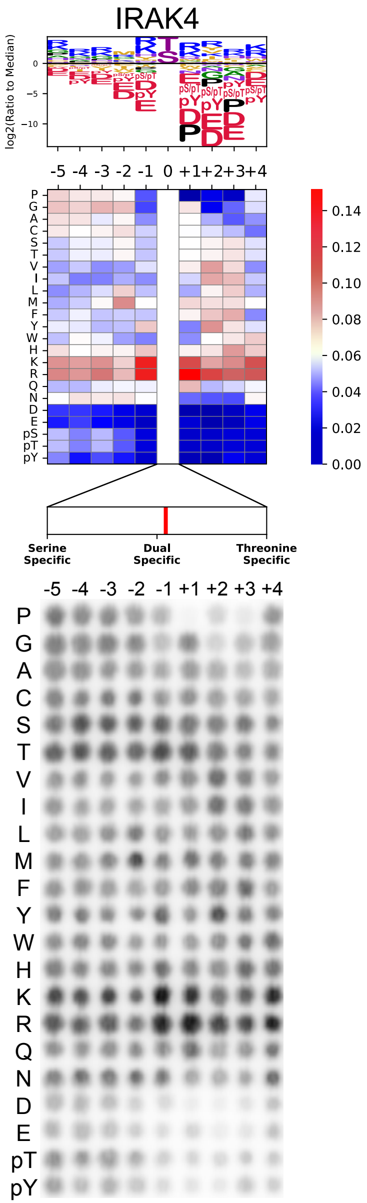
IRAK4
log2(Ratio to Median)
-5 -4 -3 -2 -1 0 +1 +2 +3 +4
P G A C S T V I L M F Y W H K R Q N D E pS pT pY
0.14 0.12 0.10 0.08 0.06 0.04 0.02 0.00
Serine Specific
Dual Specific
Threonine Specific
-5 -4 -3 -2 -1 +1 +2 +3 +4
P G A C S T V I L M F Y W H K R Q N D E pT pY

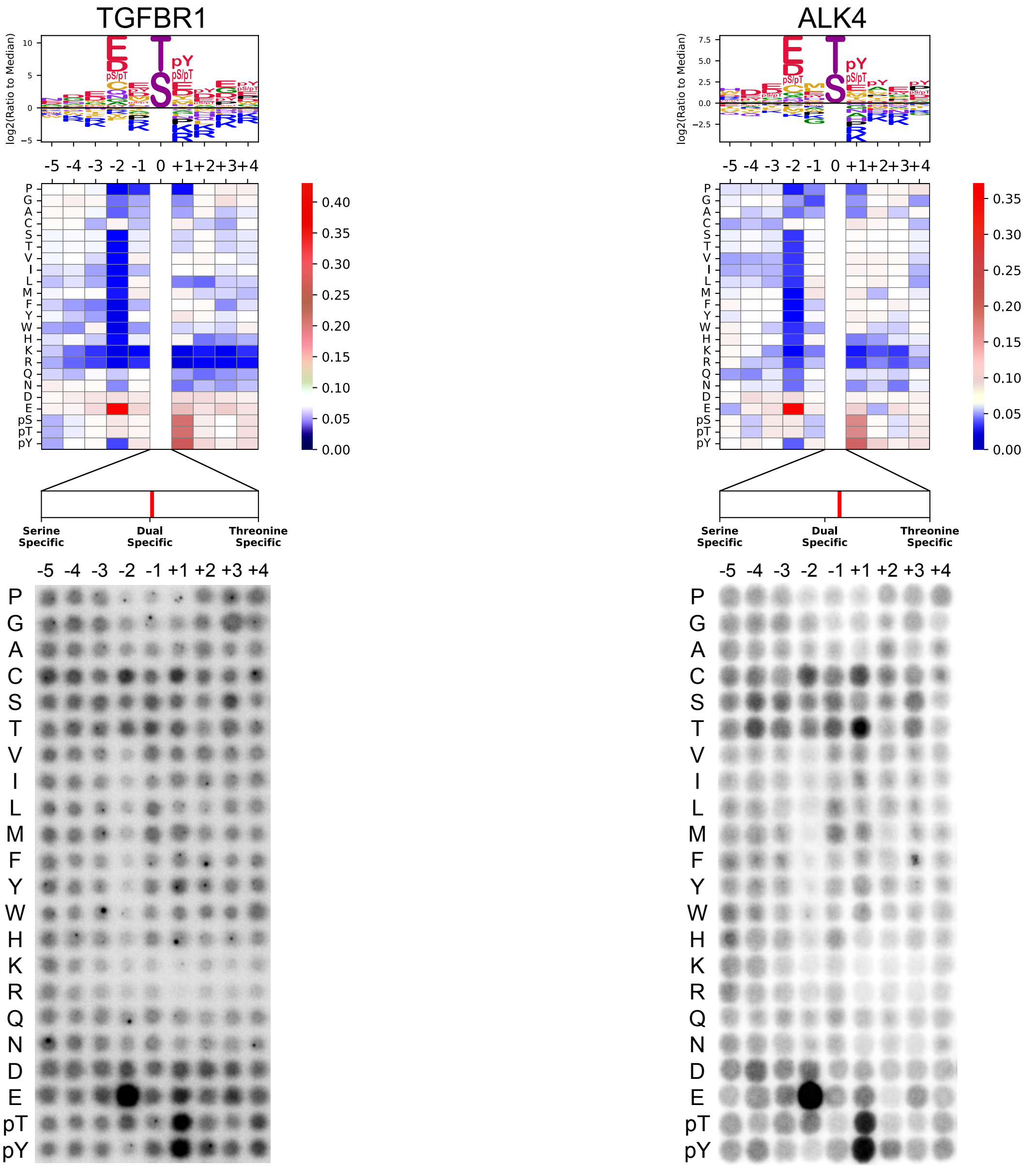
TGFBR1
ALK4
log2(Ratio to Median)
-5 -4 -3 -2 -1 0 +1 +2 +3 +4
P G A C S T V I L M F Y W H K R Q N D E pS pT pY
Serine Specific
Dual Specific
Threonine Specific
-5 -4 -3 -2 -1 +1 +2 +3 +4
P G A C S T V I L M F Y W H K R Q N D E pT pY

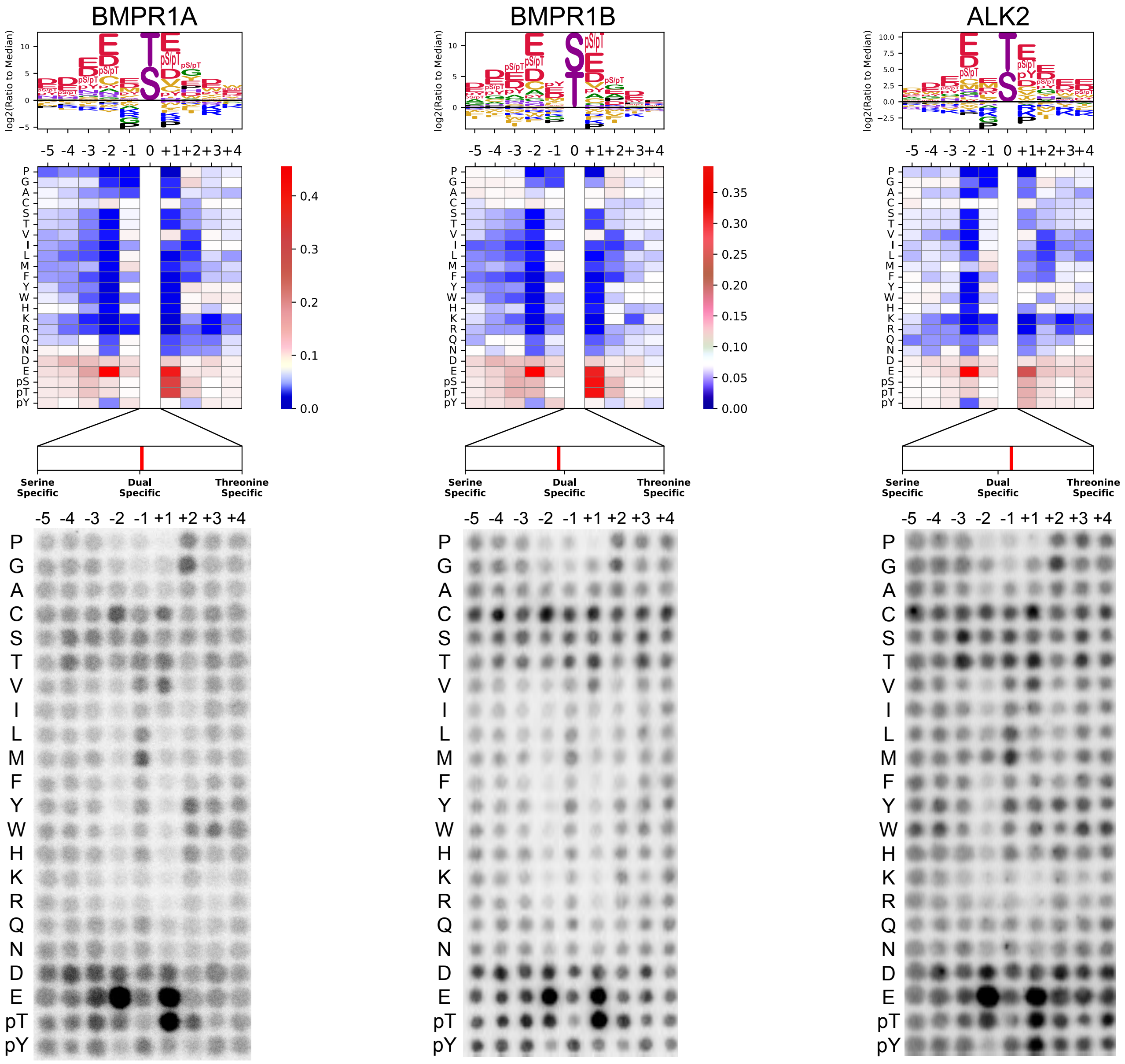
BMPR1A
BMPR1B
ALK2
log2(Ratio to Median)
-5 -4 -3 -2 -1 0 +1 +2 +3 +4
P G A C S T V I L M F Y W H K R Q N D E pS pT pY
Serine Specific
Dual Specific
Threonine Specific
-5 -4 -3 -2 -1 +1 +2 +3 +4
P G A C S T V I L M F Y W H K R Q N D E pT pY

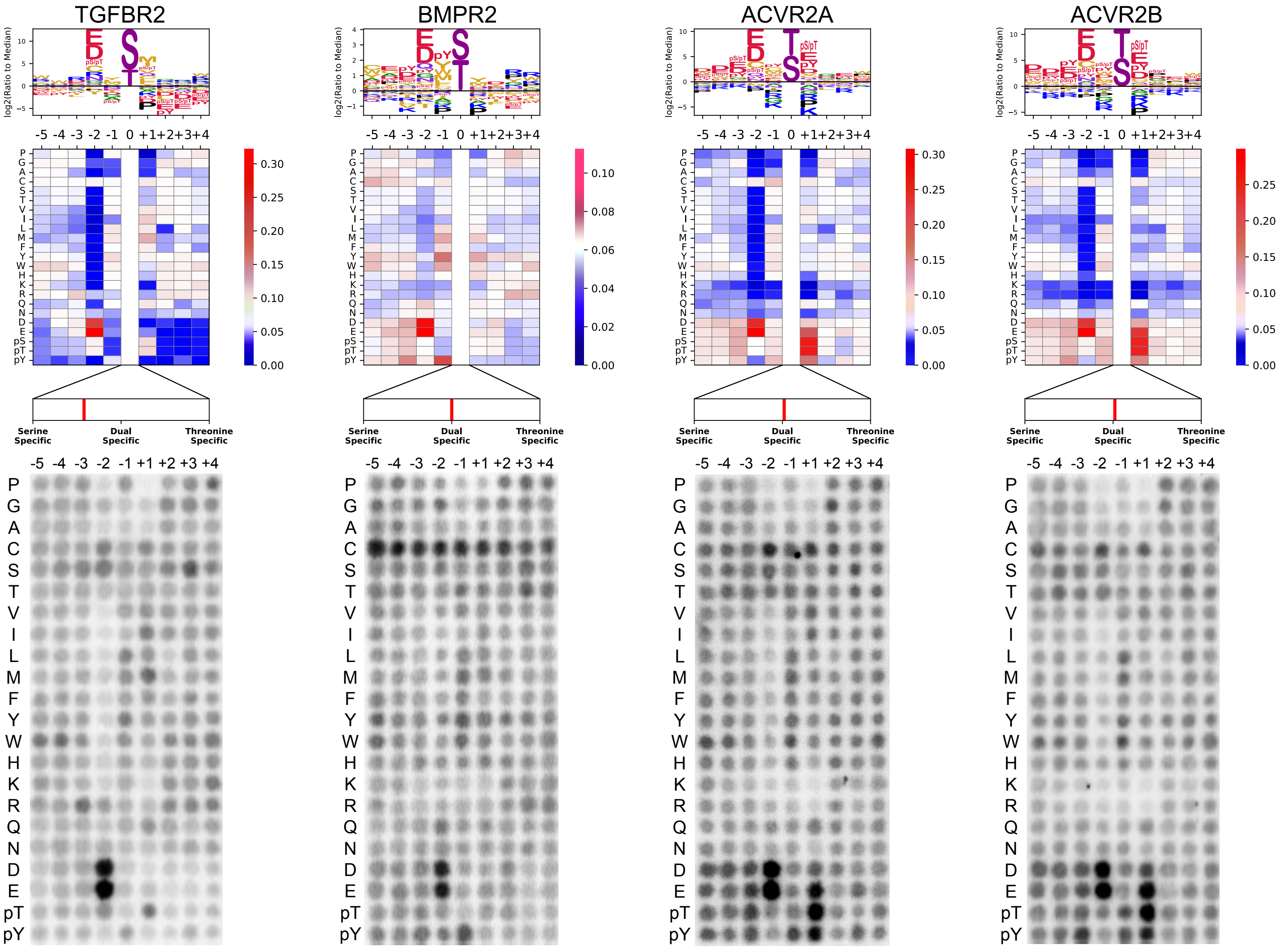

TGFBR2
BMPR2
ACVR2A
ACVR2B
log2(Ratio to Median)
-5 -4 -3 -2 -1 0 +1 +2 +3 +4
P G A C S T V I L M F Y W H K R Q N D E pS pT pY
Serine Specific
Dual Specific
Threonine Specific
-5 -4 -3 -2 -1 +1 +2 +3 +4
P G A C S T V I L M F Y W H K R Q N D E pT pY

OTHER

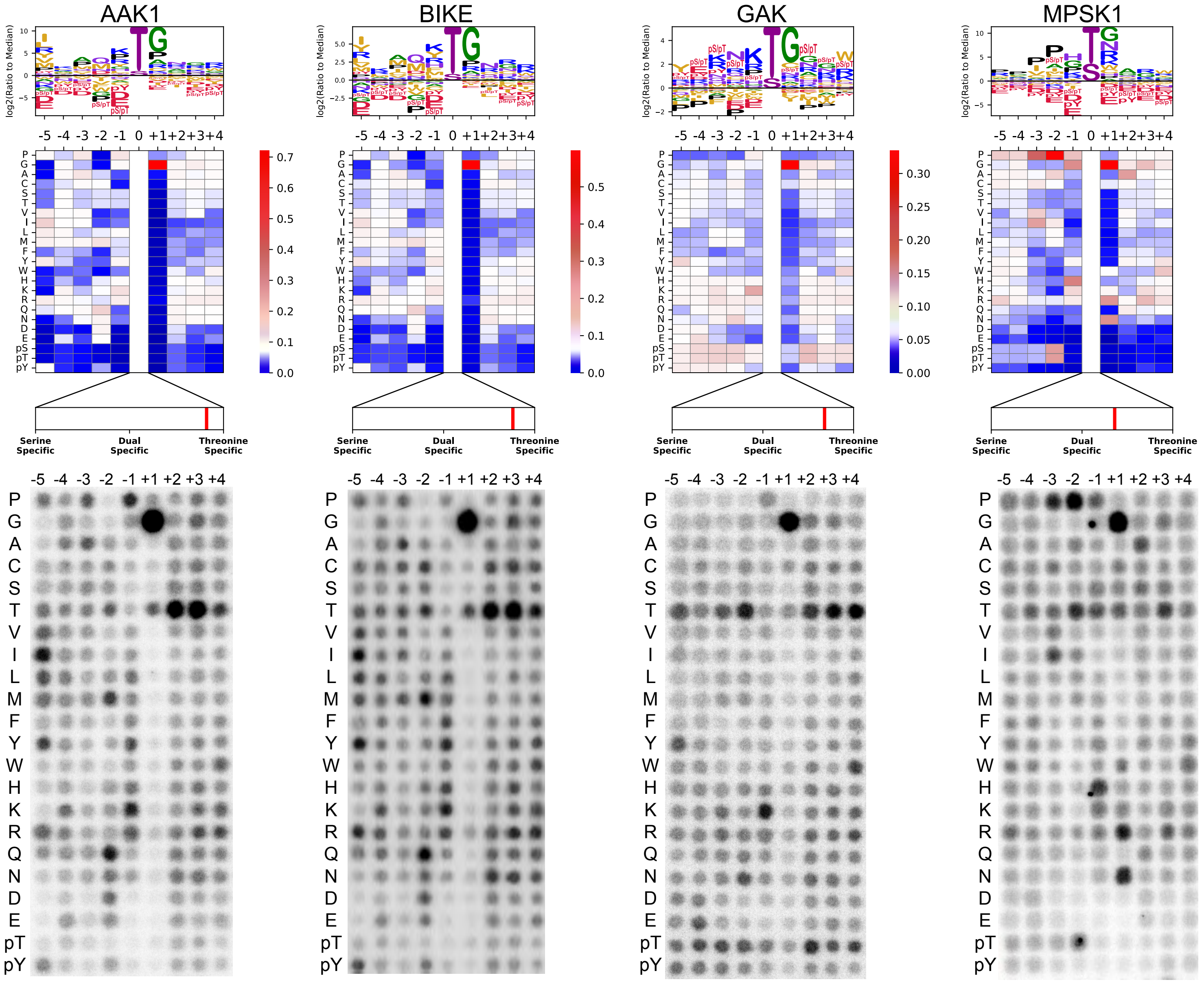
AAK1
BIKE
GAK
MPSK1
log2(Ratio to Median)
-5 -4 -3 -2 -1 0 +1 +2 +3 +4
P G A C S T V I L M F Y W H K R Q N D E pS pT pY
Serine Specific
Dual Specific
Threonine Specific
-5 -4 -3 -2 -1 +1 +2 +3 +4
P G A C S T V I L M F Y W H K R Q N D E pT pY

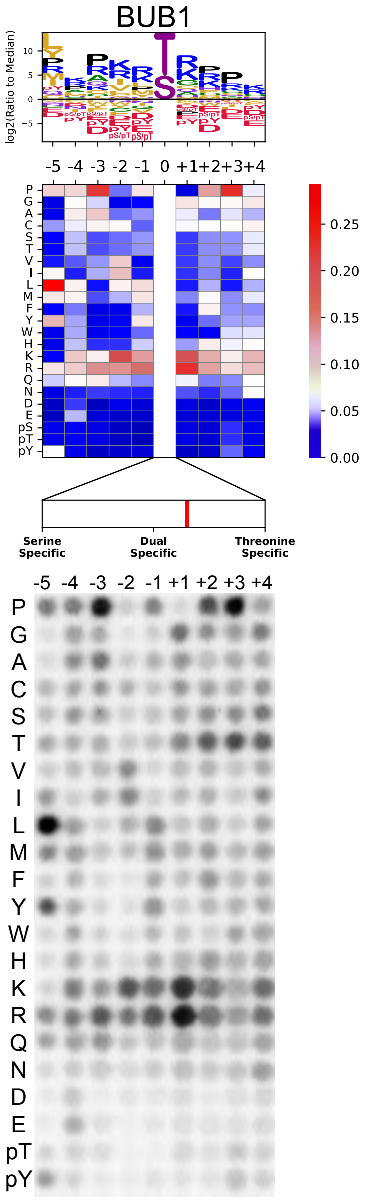
BUB1
log2(Ratio to Median)
-5 -4 -3 -2 -1 0 +1 +2 +3 +4
P G A C S T V I L M F Y W H K R Q N D E pS pT pY
0.25 0.20 0.15 0.10 0.05 0.00
Serine Specific
Dual Specific
Threonine Specific
-5 -4 -3 -2 -1 +1 +2 +3 +4
P G A C S T V I L M F Y W H K R Q N D E pT pY

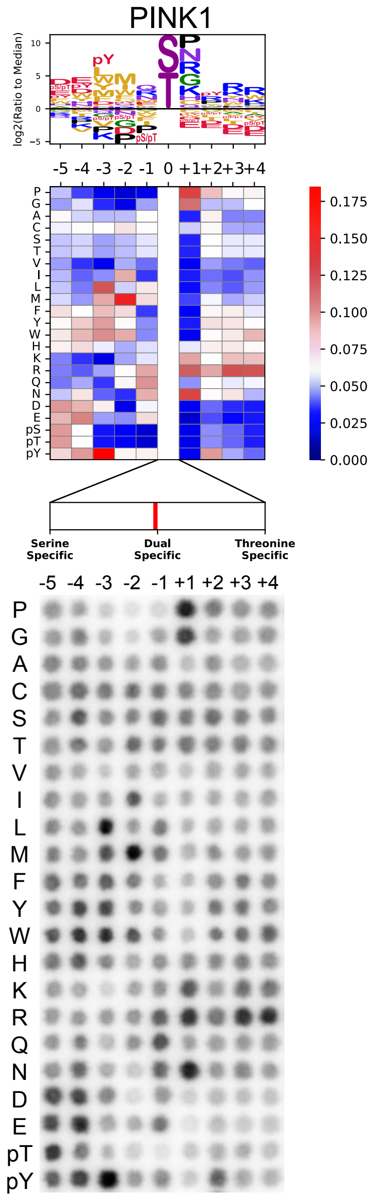

PINK1
log2(Ratio to Median)
-5 -4 -3 -2 -1 0 +1 +2 +3 +4
P G A C S T V I L M F Y W H K R Q N D E pS pT pY
0.175 0.150 0.125 0.100 0.075 0.050 0.025 0.000
Serine Specific
Dual Specific
Threonine Specific
-5 -4 -3 -2 -1 +1 +2 +3 +4
P G A C S T V I L M F Y W H K R Q N D E pT pY

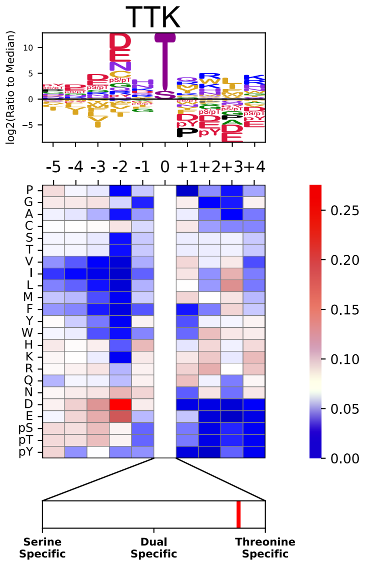
TTK
log2(Ratio to Median)
-5 -4 -3 -2 -1 0 +1 +2 +3 +4
P
G
A
C
S
T
V
I
L
M
F
Y
W
H
K
R
Q
N
D
E
pS
pT
pY
0.25
0.20
0.15
0.10
0.05
0.00
Serine Specific
Dual Specific
Threonine Specific

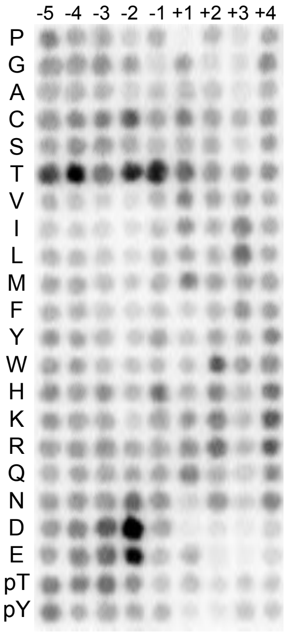
-5 -4 -3 -2 -1 +1 +2 +3 +4
P
G
A
C
S
T
V
I
L
M
F
Y
W
H
K
R
Q
N
D
E
pT
pY

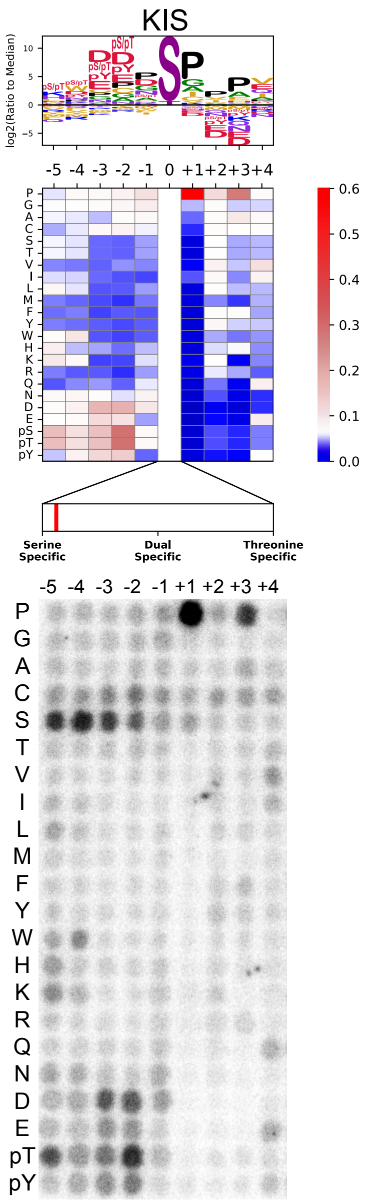
KIS
log2(Ratio to Median)
-5 -4 -3 -2 -1 0 +1 +2 +3 +4
P G A C S T V I L M F Y W H K R Q N D E pS pT pY
Serine Specific
Dual Specific
Threonine Specific
-5 -4 -3 -2 -1 +1 +2 +3 +4
P G A C S T V I L M F Y W H K R Q N D E pT pY

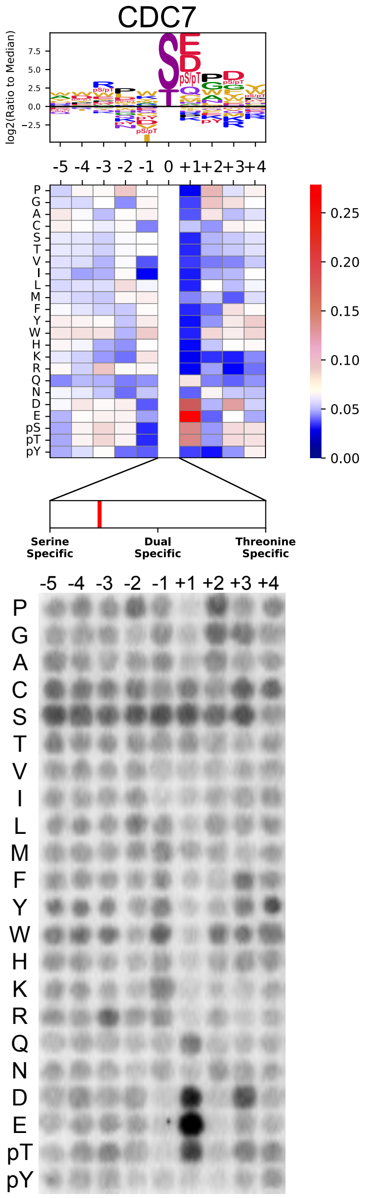

CDC7
log2(Ratio to Median)
-5 -4 -3 -2 -1 0 +1 +2 +3 +4
Serine Specific
Dual Specific
Threonine Specific
P G A C S T V I L M F Y W H K R Q N D E pS pT pY
P G A C S T V I L M F Y W H K R Q N D E pT pY

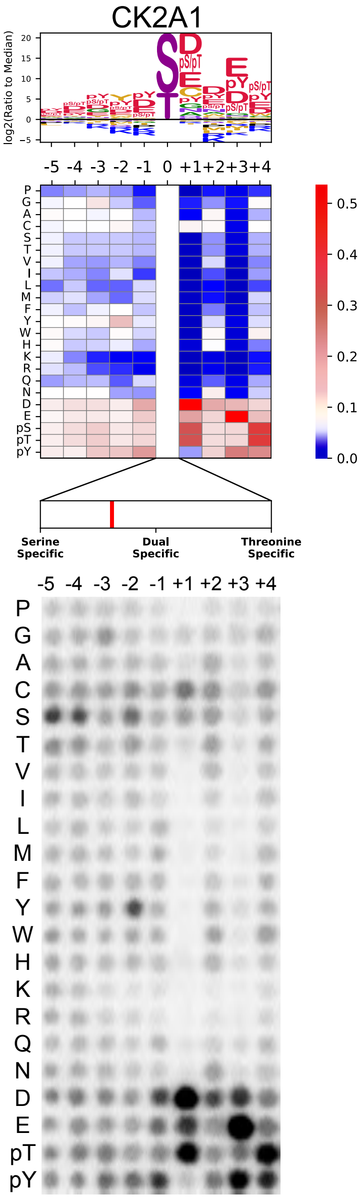
CK2A1
log2(Ratio to Median)
-5 -4 -3 -2 -1 0 +1 +2 +3 +4
P G A C S T V I L M F Y W H K R Q N D E pS pT pY
0.5 0.4 0.3 0.2 0.1 0.0
Serine Specific
Dual Specific
Threonine Specific
-5 -4 -3 -2 -1 +1 +2 +3 +4
P G A C S T V I L M F Y W H K R Q N D E pT pY

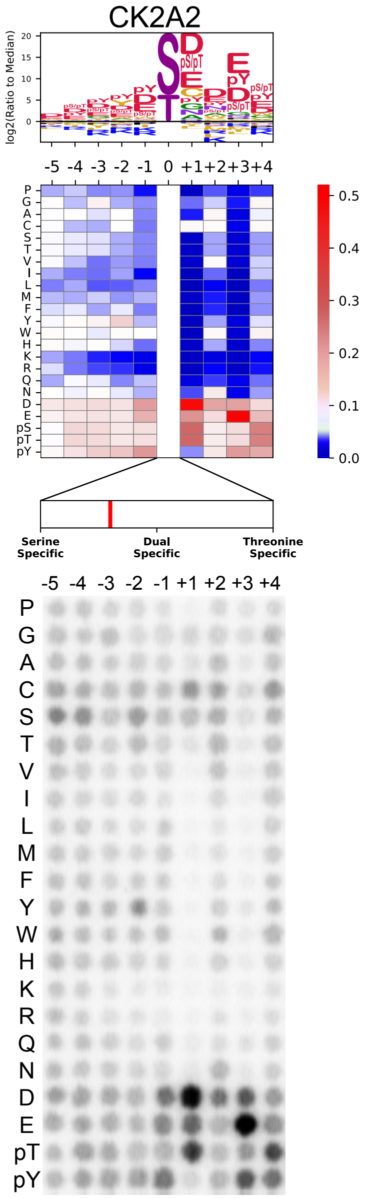
CK2A2
log2(Ratio to Median)
-5 -4 -3 -2 -1 0 +1 +2 +3 +4
P G A C S T V I L M F Y W H K R Q N D E pS pT pY
0.5 0.4 0.3 0.2 0.1 0.0
Serine Specific
Dual Specific
Threonine Specific
-5 -4 -3 -2 -1 +1 +2 +3 +4
P G A C S T V I L M F Y W H K R Q N D E pT pY

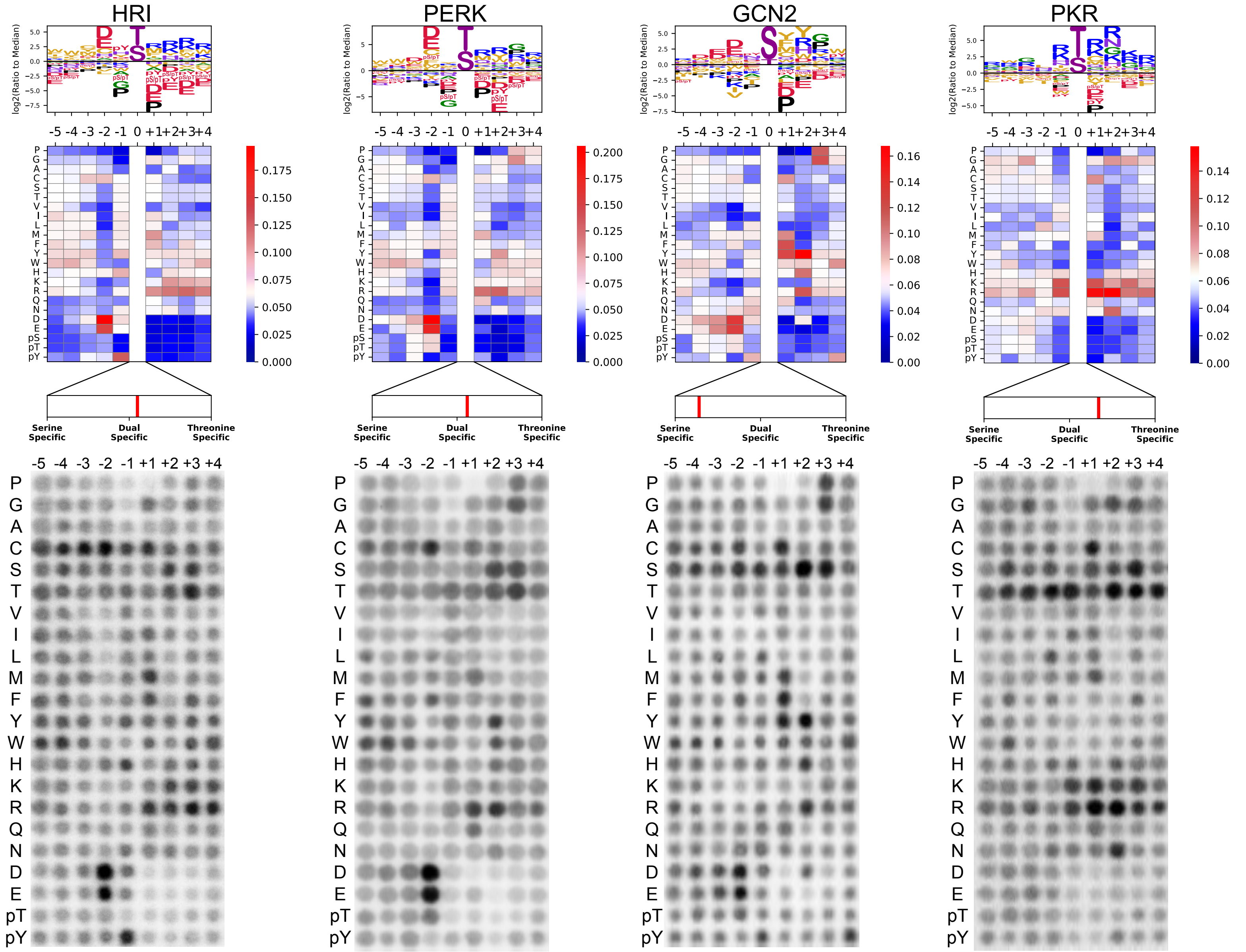

HRI
PERK
GCN2
PKR
log2(Ratio to Median)
-5 -4 -3 -2 -1 0 +1 +2 +3 +4
P G A C S T V I L M F Y W H K R Q N D E pS pT pY
Serine Specific
Dual Specific
Threonine Specific
-5 -4 -3 -2 -1 +1 +2 +3 +4
P G A C S T V I L M F Y W H K R Q N D E pT pY

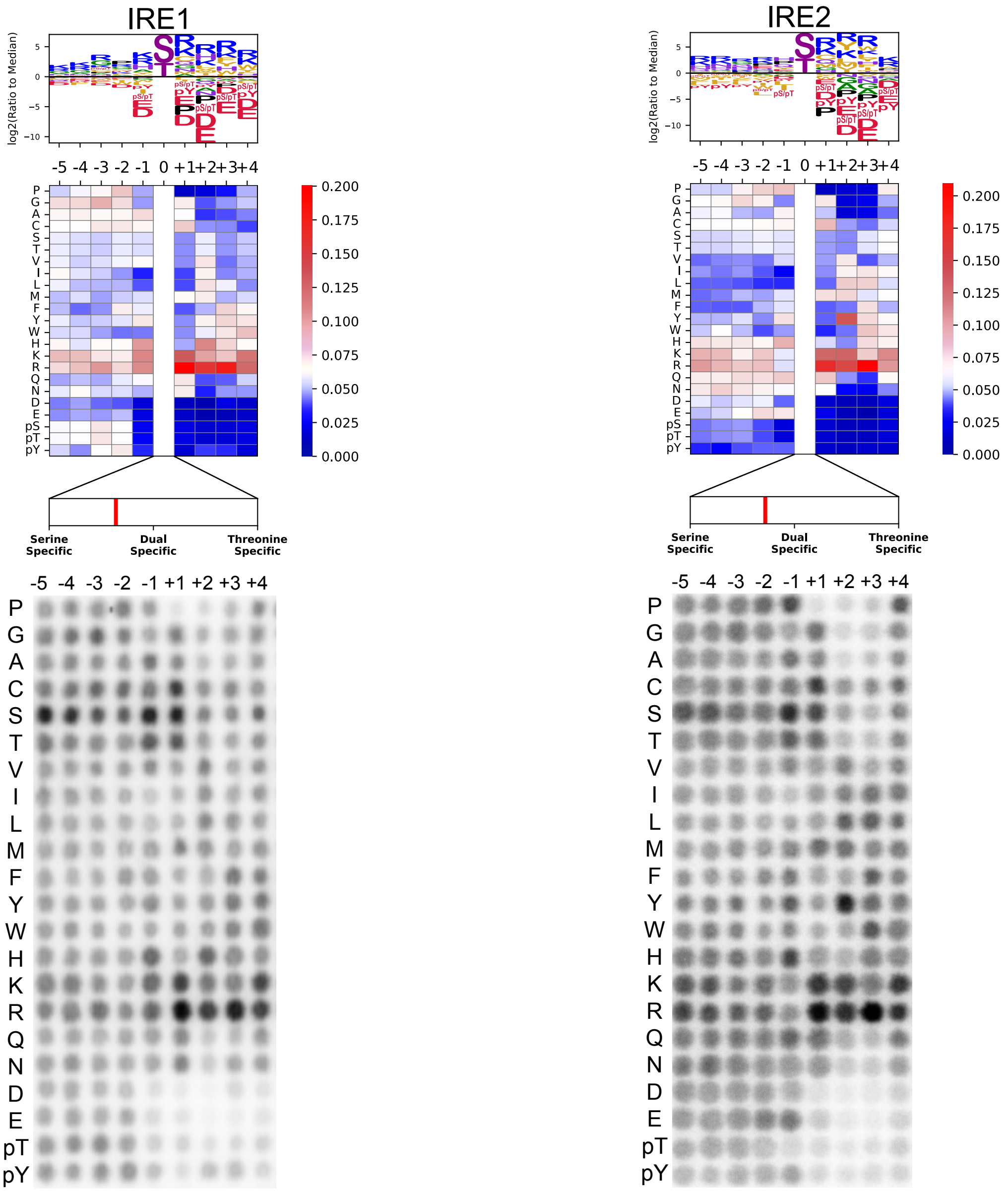
IRE1
IRE2
log2(Ratio to Median)
-5 -4 -3 -2 -1 0 +1 +2 +3 +4
P G A C S T V I L M F Y W H K R Q N D E pS pT pY
0.200 0.175 0.150 0.125 0.100 0.075 0.050 0.025 0.000
Serine Specific
Dual Specific
Threonine Specific
-5 -4 -3 -2 -1 +1 +2 +3 +4
P G A C S T V I L M F Y W H K R Q N D E pT pY

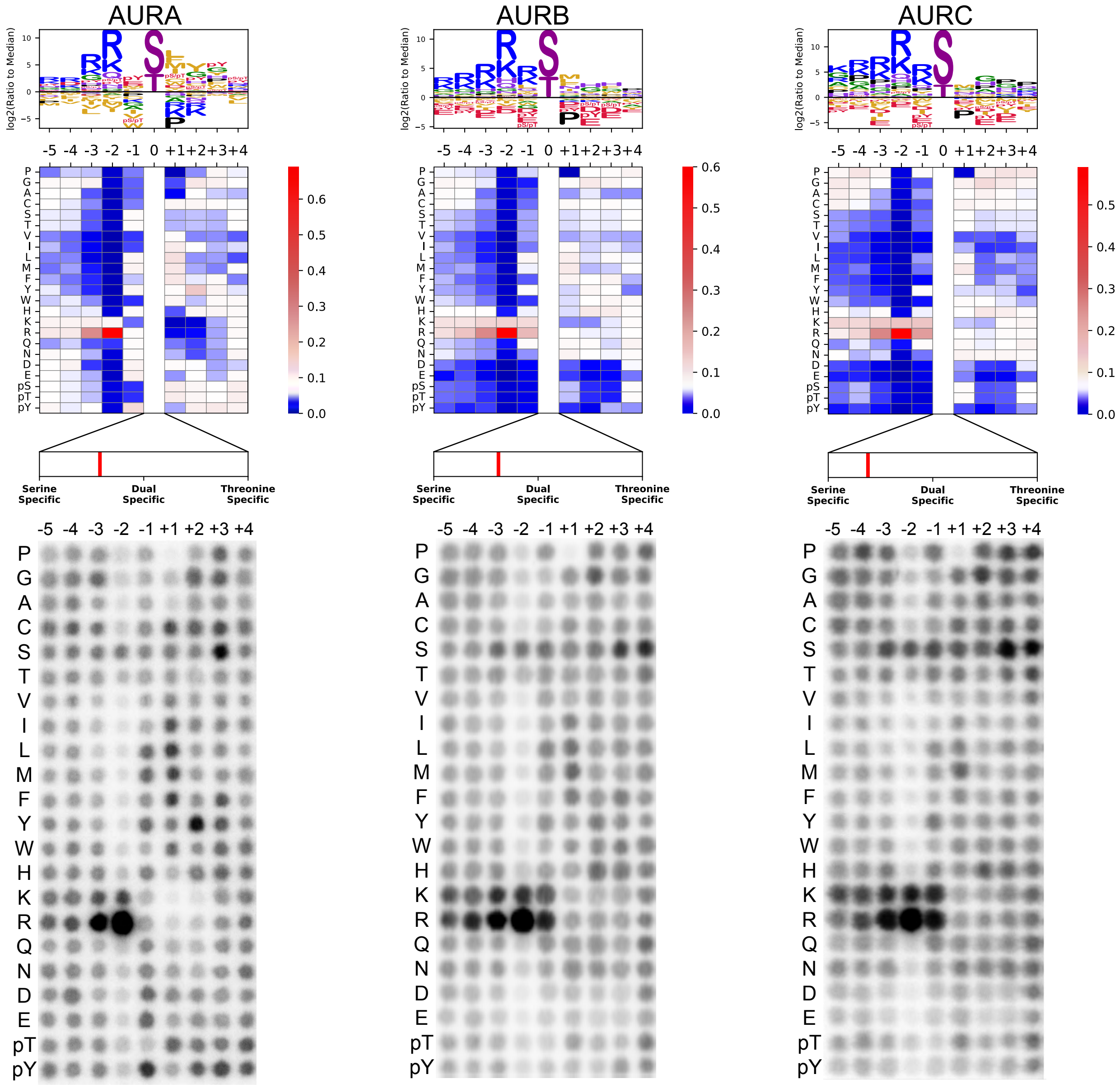
AURA
AURB
AURC
log2(Ratio to Median)
-5 -4 -3 -2 -1 0 +1 +2 +3 +4
P G A C S T V I L M F Y W H K R Q N D E pS pT pY
Serine Specific
Dual Specific
Threonine Specific
-5 -4 -3 -2 -1 +1 +2 +3 +4
P G A C S T V I L M F Y W H K R Q N D E pT pY

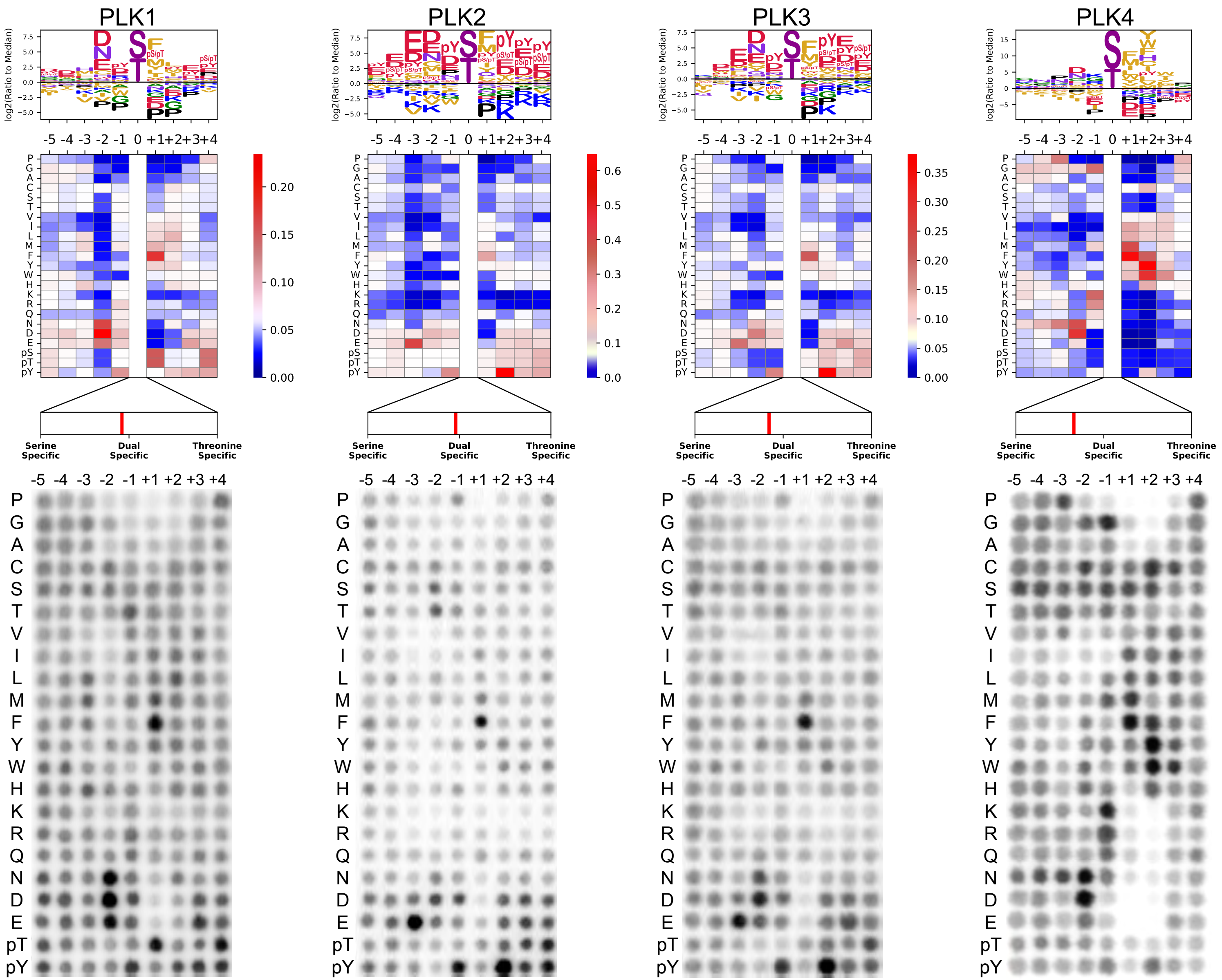
PLK1
PLK2
PLK3
PLK4
log2(Ratio to Median)
-5 -4 -3 -2 -1 0 +1 +2 +3 +4
P G A C S T V I L M F Y W H K R Q N D E pS pT pY
Serine Specific
Dual Specific
Threonine Specific
-5 -4 -3 -2 -1 +1 +2 +3 +4
P G A C S T V I L M F Y W H K R Q N D E pT pY

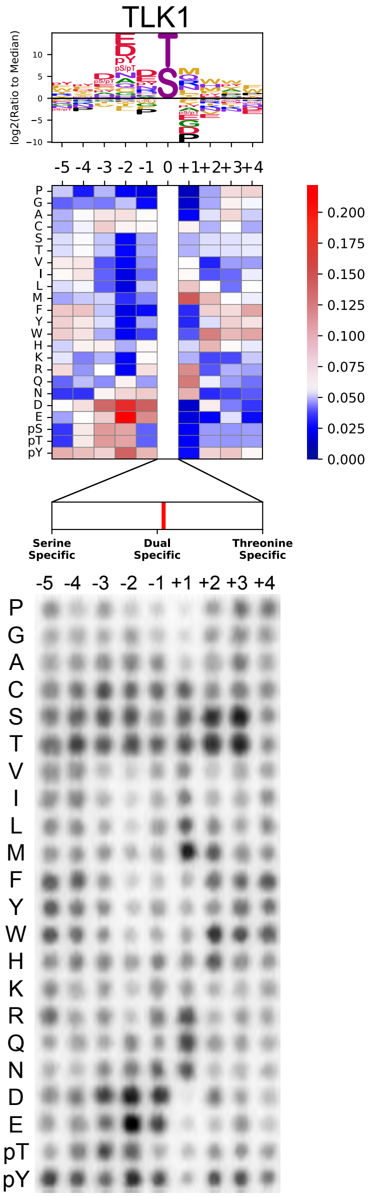
TLK1
log2(Ratio to Median)
-5 -4 -3 -2 -1 0 +1 +2 +3 +4
P G A C S T V I L M F Y W H K R Q N D E pS pT pY
0.200 0.175 0.150 0.125 0.100 0.075 0.050 0.025 0.000
Serine Specific
Dual Specific
Threonine Specific
-5 -4 -3 -2 -1 +1 +2 +3 +4
P G A C S T V I L M F Y W H K R Q N D E pT pY

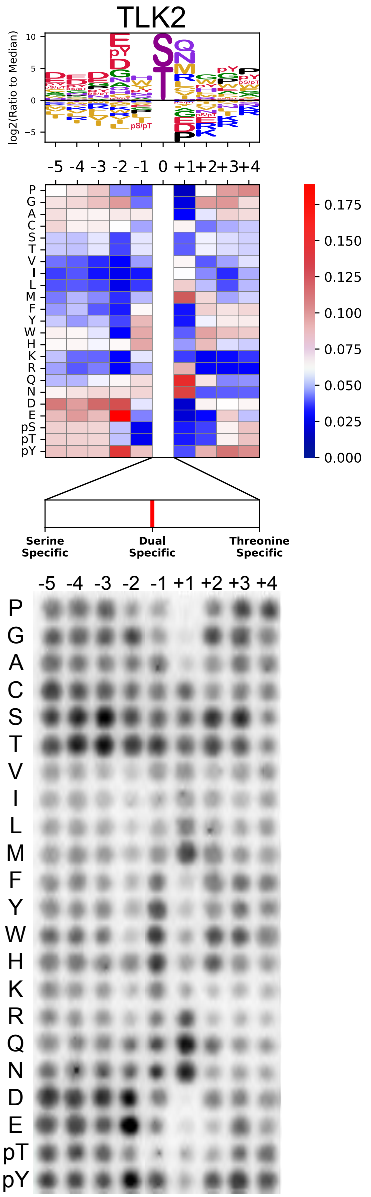
TLK2
log2(Ratio to Median)
-5 -4 -3 -2 -1 0 +1 +2 +3 +4
P G A C S T V I L M F Y W H K R Q N D E pS pT pY
0.175 0.150 0.125 0.100 0.075 0.050 0.025 0.000
Serine Specific
Dual Specific
Threonine Specific
-5 -4 -3 -2 -1 +1 +2 +3 +4
P G A C S T V I L M F Y W H K R Q N D E pT pY

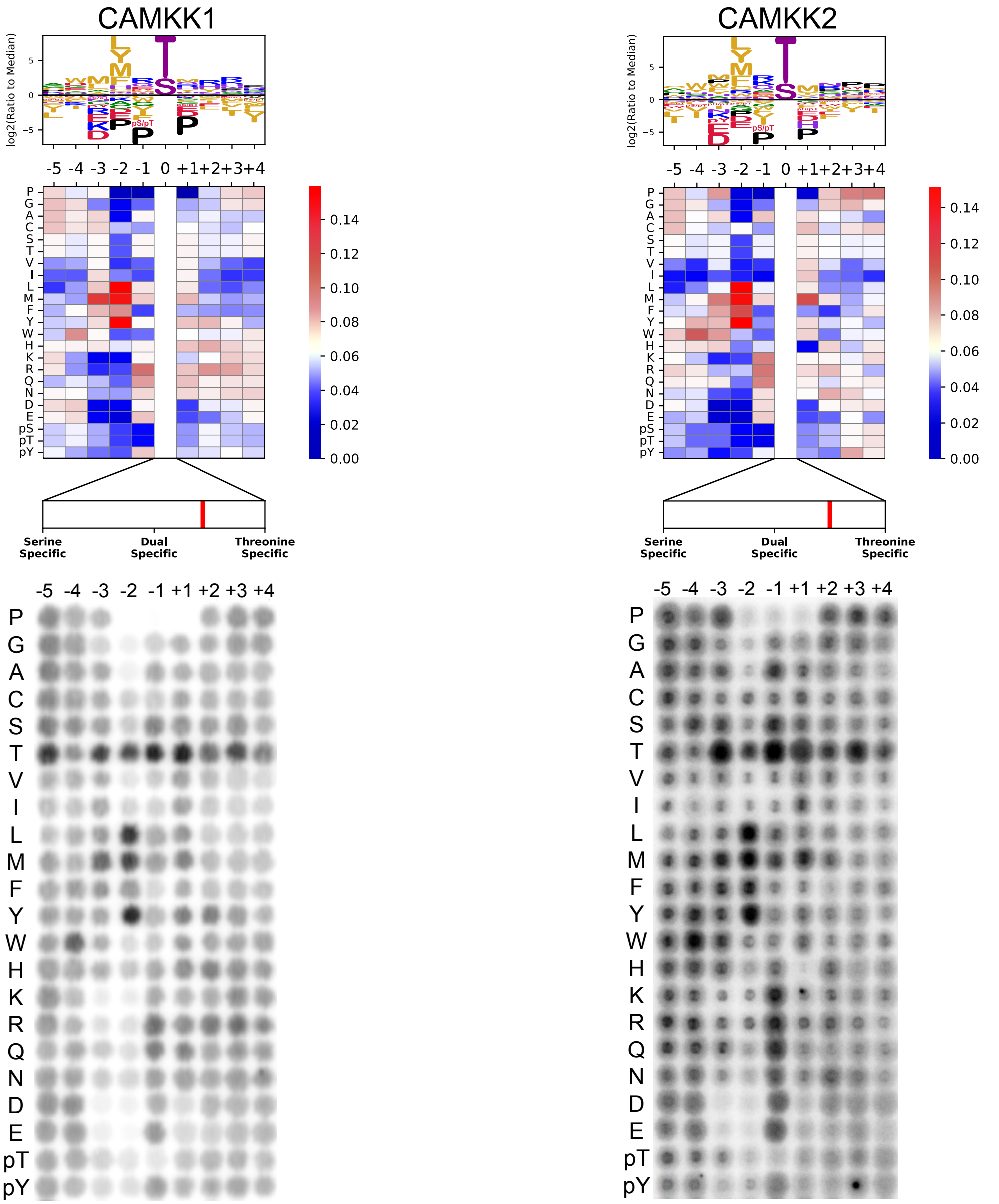
CAMKK1
CAMKK2
log2(Ratio to Median)
5
0
-5
pS/pT
-5 -4 -3 -2 -1 0 +1+2+3+4
P G A C S T V I L M F Y W H K R Q N D E pS pT pY
0.14
0.12
0.10
0.08
0.06
0.04
0.02
0.00
Serine Specific
Dual Specific
Threonine Specific
-5 -4 -3 -2 -1 +1 +2 +3 +4
P G A C S T V I L M F Y W H K R Q N D E pT pY

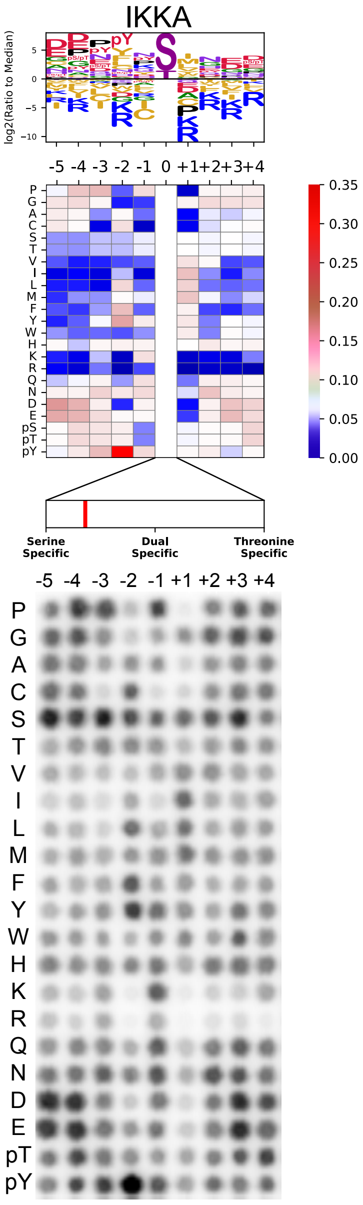
IKKA
log2(Ratio to Median)
-5 -4 -3 -2 -1 0 +1 +2 +3 +4
P G A C S T V I L M F Y W H K R Q N D E pS pT pY
0.35 0.30 0.25 0.20 0.15 0.10 0.05 0.00
Serine Specific
Dual Specific
Threonine Specific
-5 -4 -3 -2 -1 +1 +2 +3 +4
P G A C S T V I L M F Y W H K R Q N D E pT pY

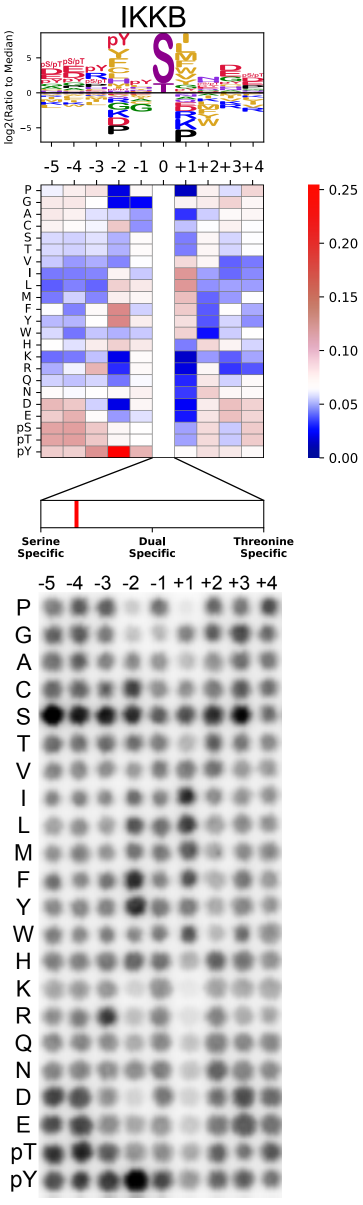
IKKB
log2(Ratio to Median)
-5 -4 -3 -2 -1 0 +1 +2 +3 +4
P G A C S T V I L M F Y W H K R Q N D E pS pT pY
0.25 0.20 0.15 0.10 0.05 0.00
Serine Specific
Dual Specific
Threonine Specific
-5 -4 -3 -2 -1 +1 +2 +3 +4
P G A C S T V I L M F Y W H K R Q N D E pT pY

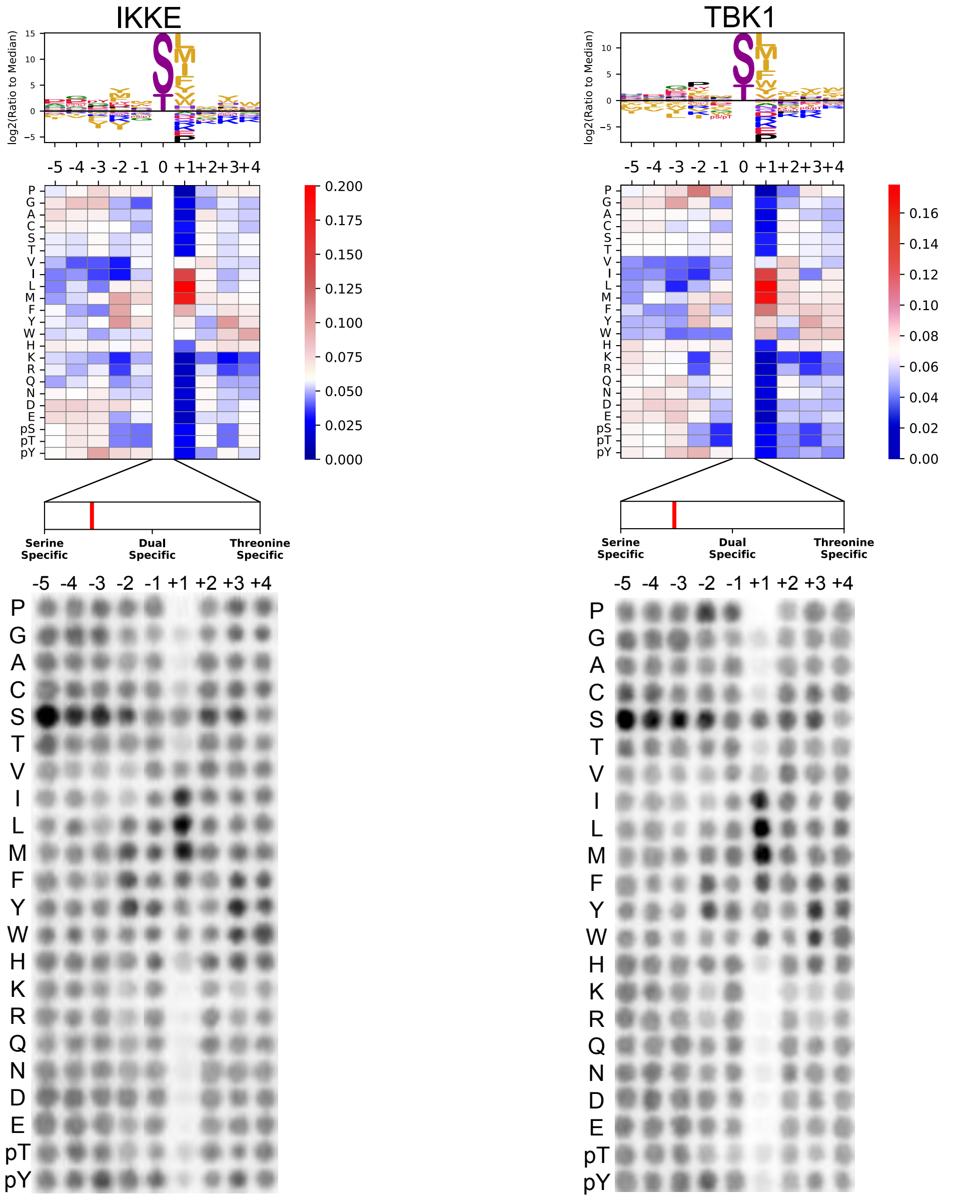
IKKE
TBK1
log2(Ratio to Median)
-5 -4 -3 -2 -1 0 +1 +2 +3 +4
P G A C S T V I L M F Y W H K R Q N D E pS pT pY
Serine Specific
Dual Specific
Threonine Specific
P G A C S T V I L M F Y W H K R Q N D E pT pY

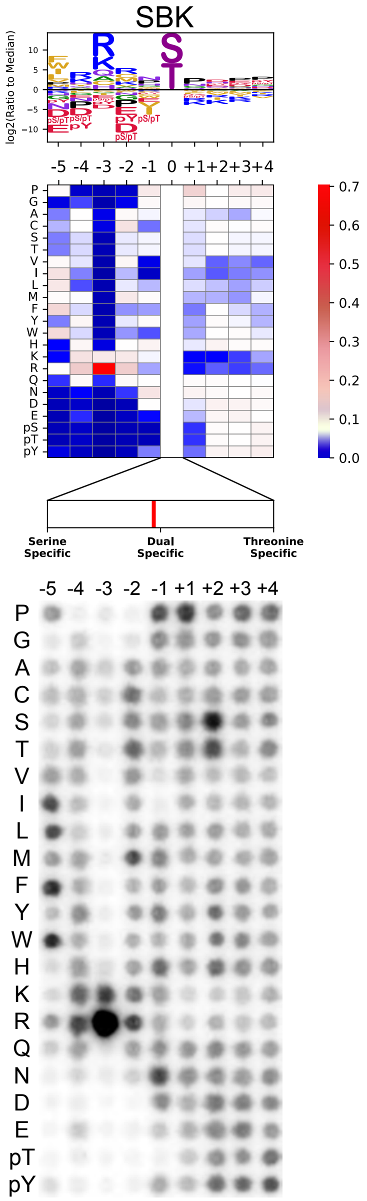
SBK
log2(Ratio to Median)
-5 -4 -3 -2 -1 0 +1 +2 +3 +4
P G A C S T V I L M F Y W H K R Q N D E pS pT pY
0.7 0.6 0.5 0.4 0.3 0.2 0.1 0.0
Serine Specific
Dual Specific
Threonine Specific
-5 -4 -3 -2 -1 +1 +2 +3 +4
P G A C S T V I L M F Y W H K R Q N D E pT pY

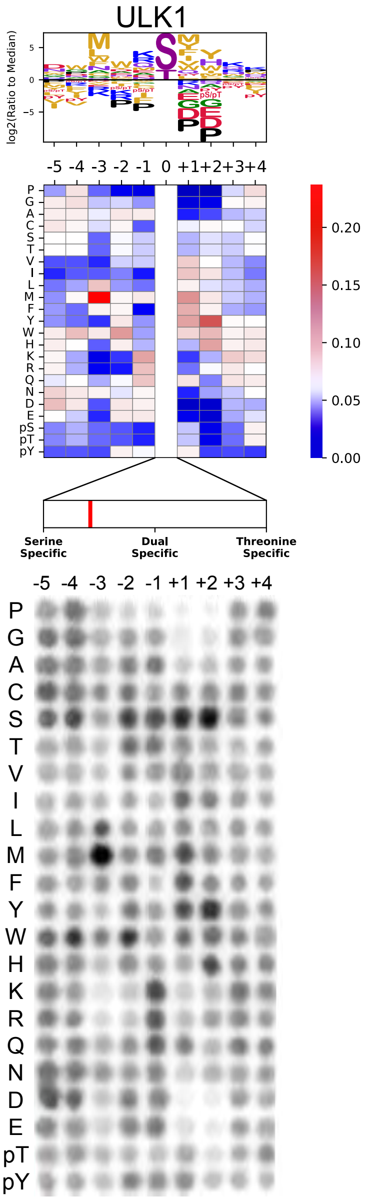
ULK1
log2(Ratio to Median)
5
0
−5
-5 -4 -3 -2 -1 0 +1 +2 +3 +4
P G A C S T V I L M F Y W H K R Q N D E pS pT pY
0.20
0.15
0.10
0.05
0.00
Serine Specific
Dual Specific
Threonine Specific
-5 -4 -3 -2 -1 +1 +2 +3 +4
P G A C S T V I L M F Y W H K R Q N D E pT pY

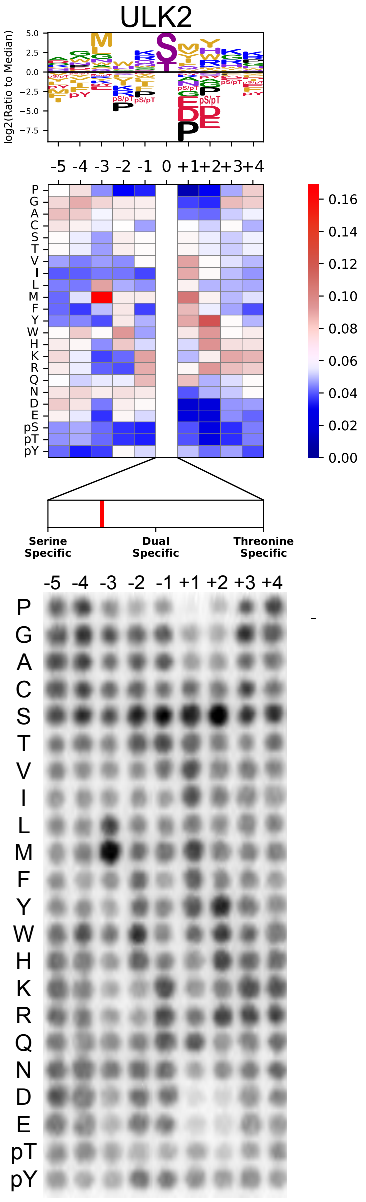
ULK2
log2(Ratio to Median)
5.0
2.5
0.0
−2.5
−5.0
−7.5
-5 -4 -3 -2 -1 0 +1 +2 +3 +4
P G A C S T V I L M F Y W H K R Q N D E pS pT pY
0.16
0.14
0.12
0.10
0.08
0.06
0.04
0.02
0.00
Serine Specific
Dual Specific
Threonine Specific
-5 -4 -3 -2 -1 +1 +2 +3 +4
P G A C S T V I L M F Y W H K R Q N D E pT pY

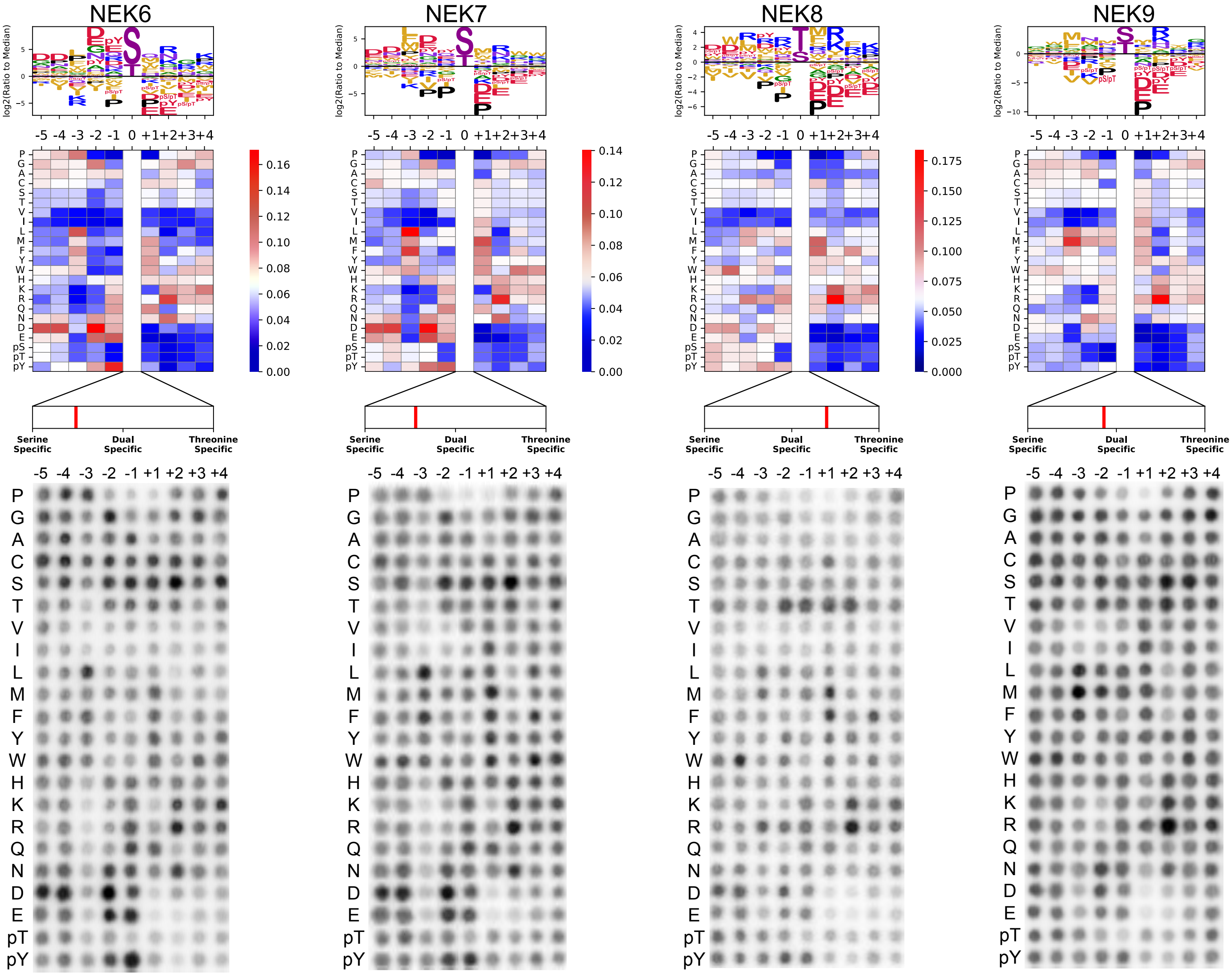
NEK6
NEK7
NEK8
NEK9
log2(Ratio to Median)
-5 -4 -3 -2 -1 0 +1 +2 +3 +4
P G A C S T V I L M F Y W H K R Q N D E pS pT pY
Serine Specific
Dual Specific
Threonine Specific
-5 -4 -3 -2 -1 +1 +2 +3 +4
P G A C S T V I L M F Y W H K R Q N D E pT pY

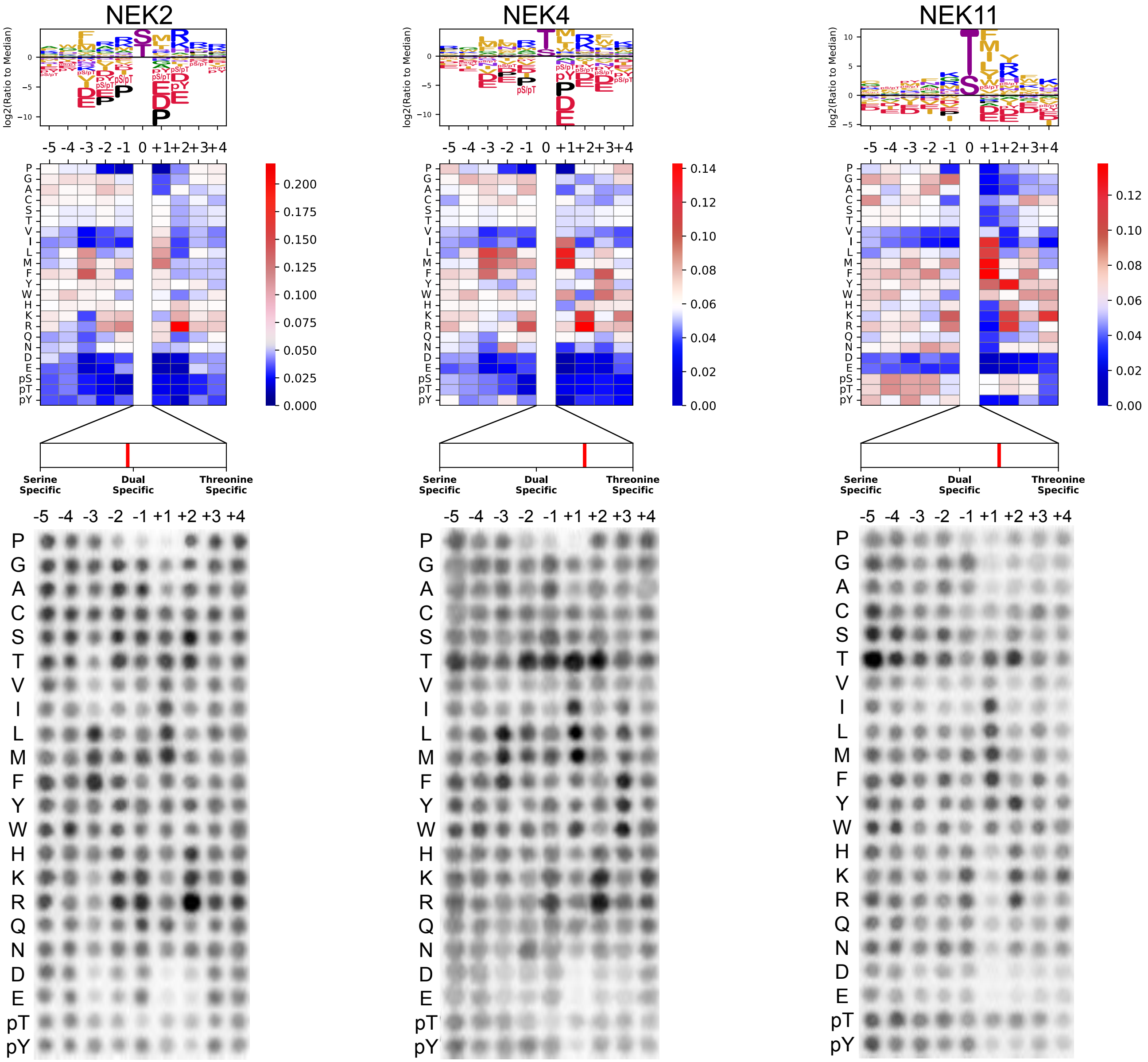
NEK2
NEK4
NEK11
log2(Ratio to Median)
-5 -4 -3 -2 -1 0 +1 +2 +3 +4
P G A C S T V I L M F Y W H K R Q N D E pS pT pY
0.200 0.175 0.150 0.125 0.100 0.075 0.050 0.025 0.000
0.14 0.12 0.10 0.08 0.06 0.04 0.02 0.00
0.12 0.10 0.08 0.06 0.04 0.02 0.00
Serine Specific
Dual Specific
Threonine Specific
-5 -4 -3 -2 -1 +1 +2 +3 +4
P G A C S T V I L M F Y W H K R Q N D E pT pY

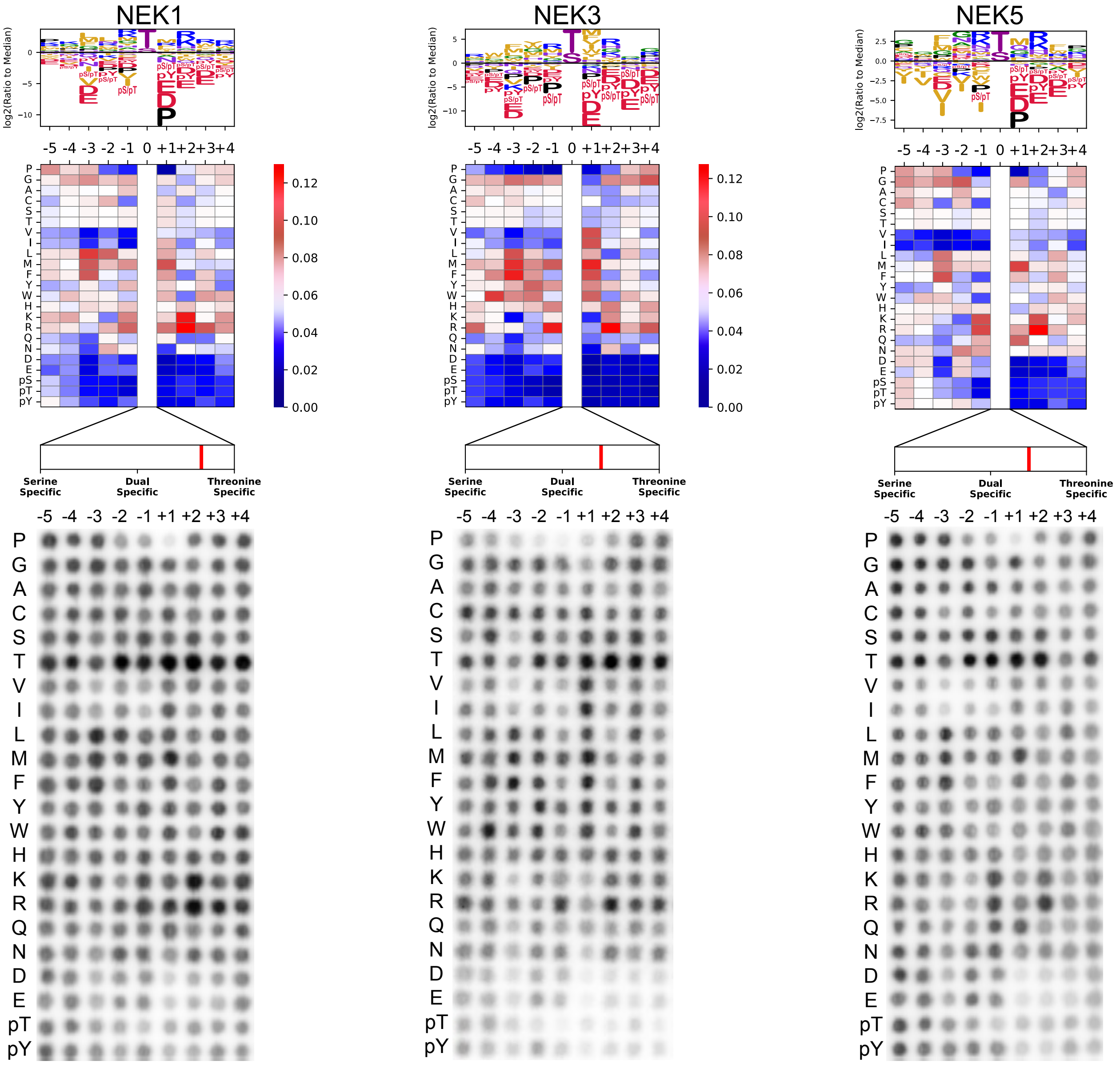
NEK1
NEK3
NEK5
log2(Ratio to Median)
-5 -4 -3 -2 -1 0 +1 +2 +3 +4
P G A C S T V I L M F Y W H K R Q N D E pS pT pY
0.12
0.10
0.08
0.06
0.04
0.02
0.00
Serine Specific
Dual Specific
Threonine Specific
-5 -4 -3 -2 -1 +1 +2 +3 +4
P G A C S T V I L M F Y W H K R Q N D E pT pY

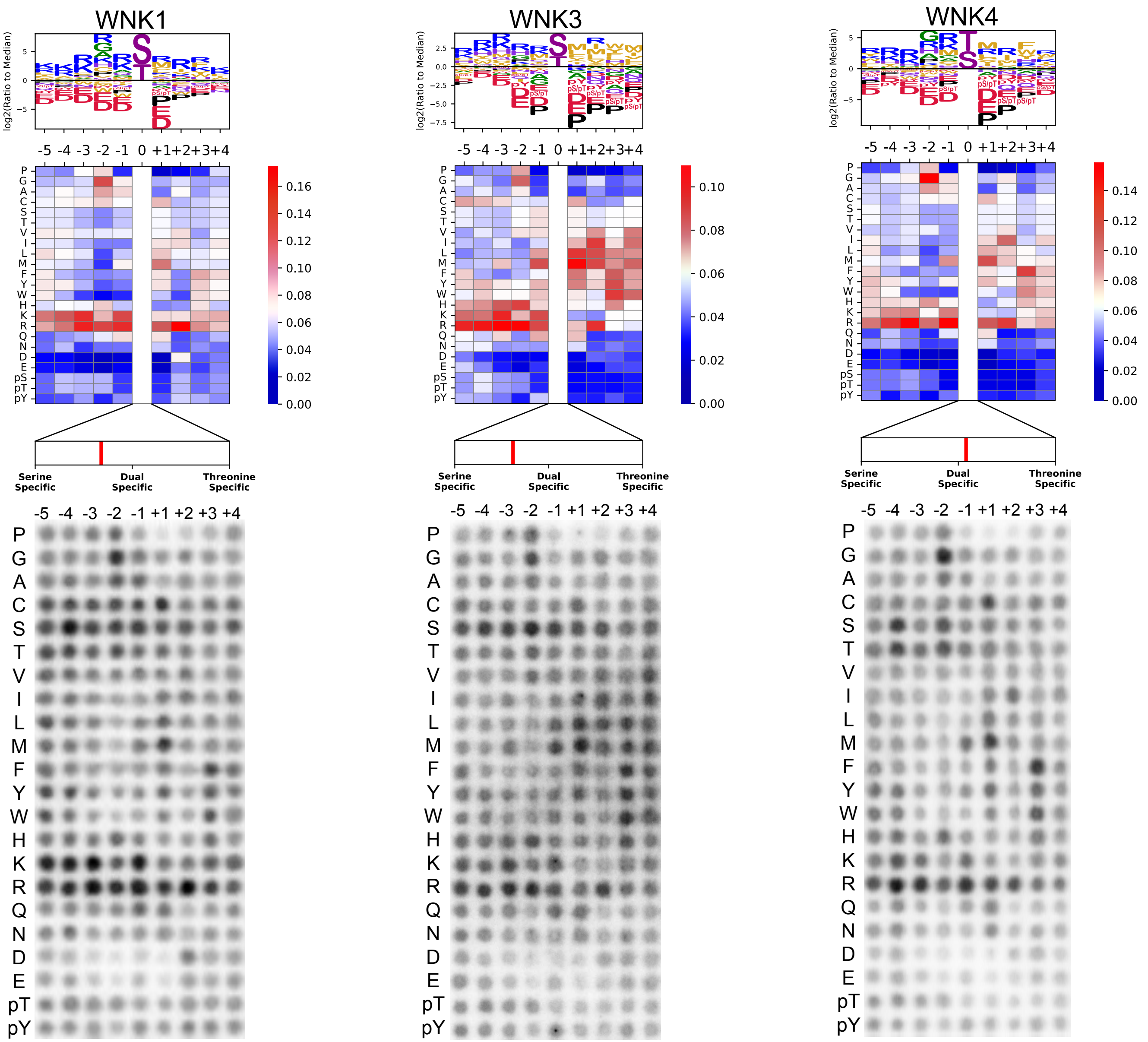

WNK1
WNK3
WNK4
log2(Ratio to Median)
-5 -4 -3 -2 -1 0 +1 +2 +3 +4
P G A C S T V I L M F Y W H K R Q N D E pS pT pY
Serine Specific
Dual Specific
Threonine Specific
-5 -4 -3 -2 -1 +1 +2 +3 +4
P G A C S T V I L M F Y W H K R Q N D E pT pY

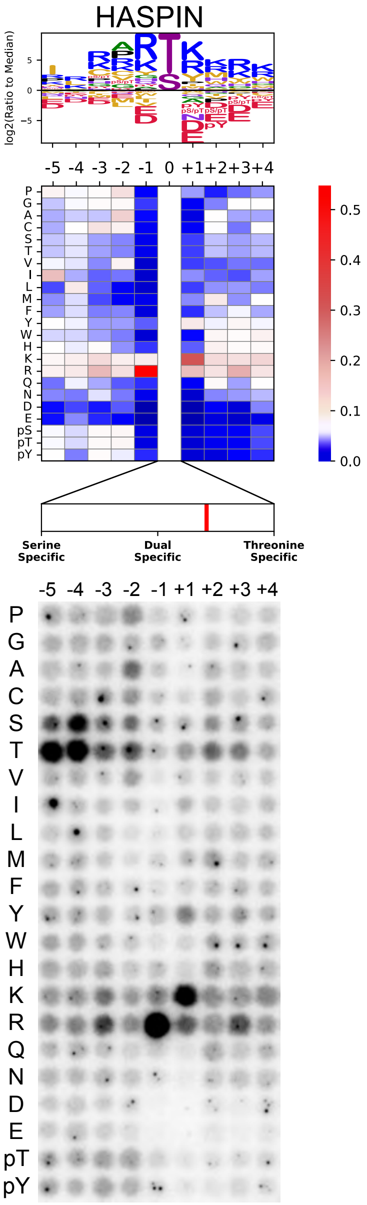
HASPIN
log2(Ratio to Median)
-5 -4 -3 -2 -1 0 +1 +2 +3 +4
P G A C S T V I L M F Y W H K R Q N D E pS pT pY
0.5 0.4 0.3 0.2 0.1 0.0
Serine Specific
Dual Specific
Threonine Specific
-5 -4 -3 -2 -1 +1 +2 +3 +4
P G A C S T V I L M F Y W H K R Q N D E pT pY

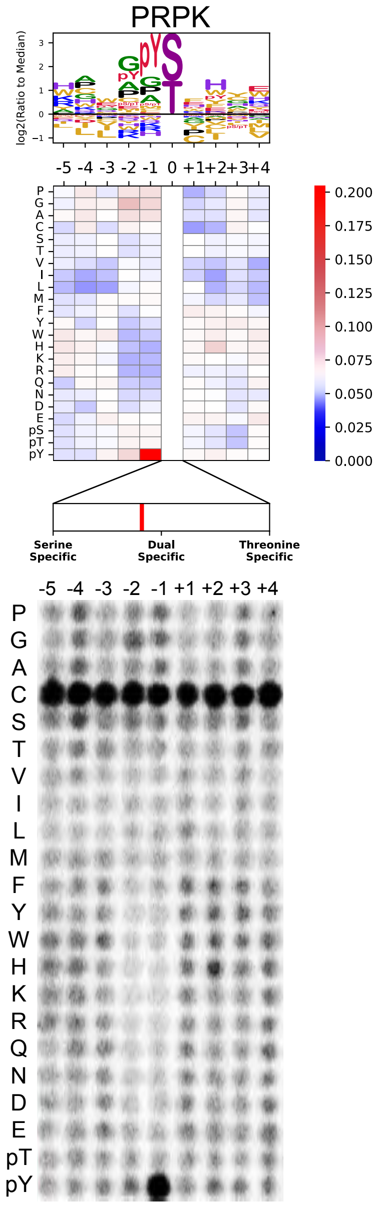
PRPK
log2(Ratio to Median)
-5 -4 -3 -2 -1 0 +1 +2 +3 +4
P G A C S T V I L M F Y W H K R Q N D E pS pT pY
0.200 0.175 0.150 0.125 0.100 0.075 0.050 0.025 0.000
Serine Specific
Dual Specific
Threonine Specific
-5 -4 -3 -2 -1 +1 +2 +3 +4
P G A C S T V I L M F Y W H K R Q N D E pT pY

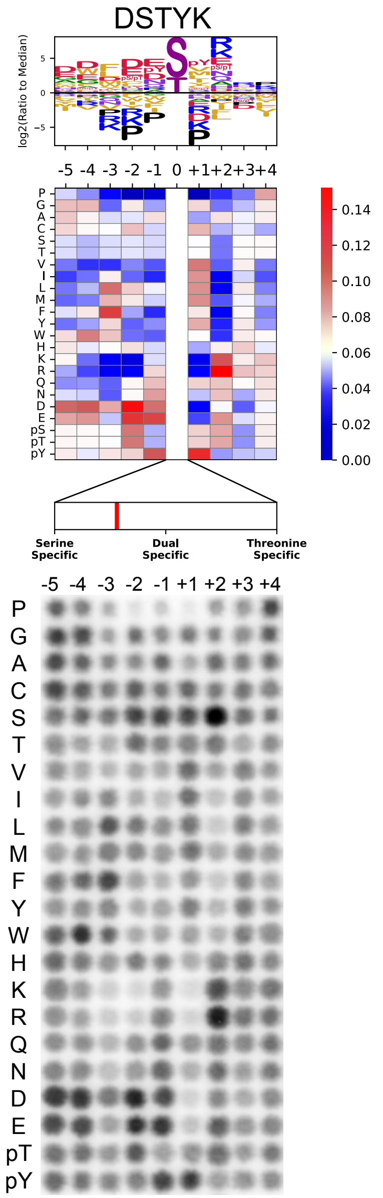
DSTYK
log2(Ratio to Median)
-5 -4 -3 -2 -1 0 +1 +2 +3 +4
P G A C S T V I L M F Y W H K R Q N D E pS pT pY
0.14 0.12 0.10 0.08 0.06 0.04 0.02 0.00
Serine Specific
Dual Specific
Threonine Specific
-5 -4 -3 -2 -1 +1 +2 +3 +4
P G A C S T V I L M F Y W H K R Q N D E pT pY

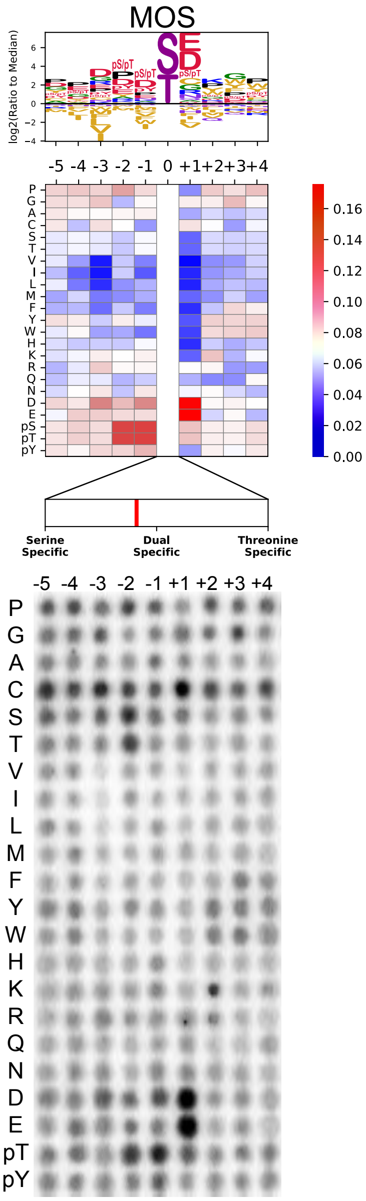
MOS
log2(Ratio to Median)
-5 -4 -3 -2 -1 0 +1 +2 +3 +4
P G A C S T V I L M F Y W H K R Q N D E pS pT pY
0.16 0.14 0.12 0.10 0.08 0.06 0.04 0.02 0.00
Serine Specific
Dual Specific
Threonine Specific
-5 -4 -3 -2 -1 +1 +2 +3 +4
P G A C S T V I L M F Y W H K R Q N D E pT pY

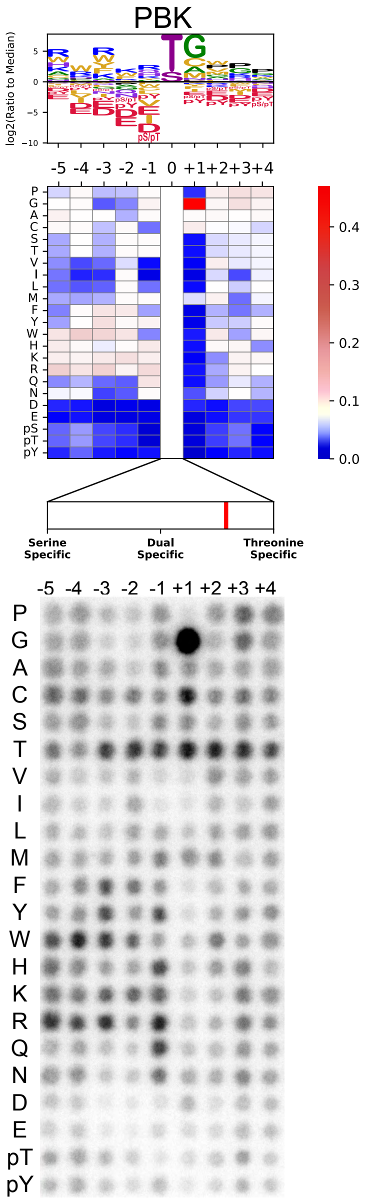
PBK
log2(Ratio to Median)
-5 -4 -3 -2 -1 0 +1 +2 +3 +4
P G A C S T V I L M F Y W H K R Q N D E pS pT pY
0.0 0.1 0.2 0.3 0.4
Serine Specific
Dual Specific
Threonine Specific
-5 -4 -3 -2 -1 +1 +2 +3 +4
P G A C S T V I L M F Y W H K R Q N D E pT pY

ATYPICAL

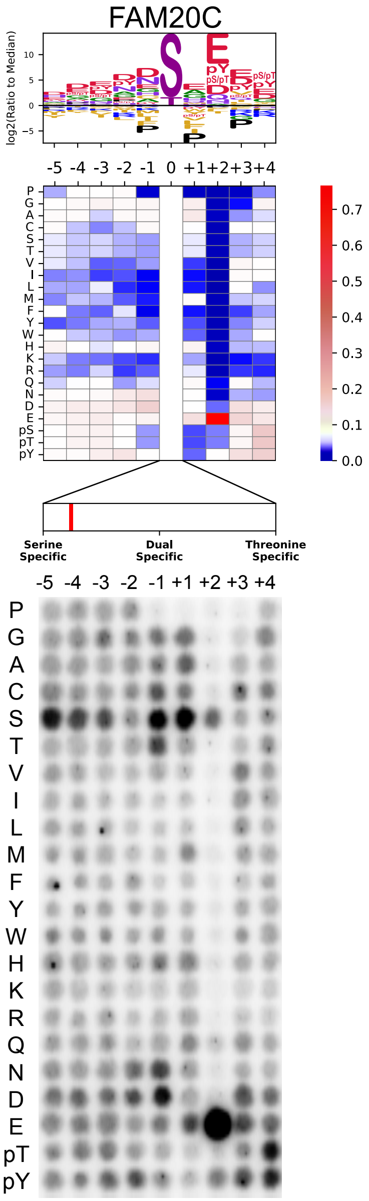
FAM20C
log2(Ratio to Median)
-5 -4 -3 -2 -1 0 +1 +2 +3 +4
P G A C S T V I L M F Y W H K R Q N D E pS pT pY
0.7 0.6 0.5 0.4 0.3 0.2 0.1 0.0
Serine Specific
Dual Specific
Threonine Specific
-5 -4 -3 -2 -1 +1 +2 +3 +4
P G A C S T V I L M F Y W H K R Q N D E pT pY

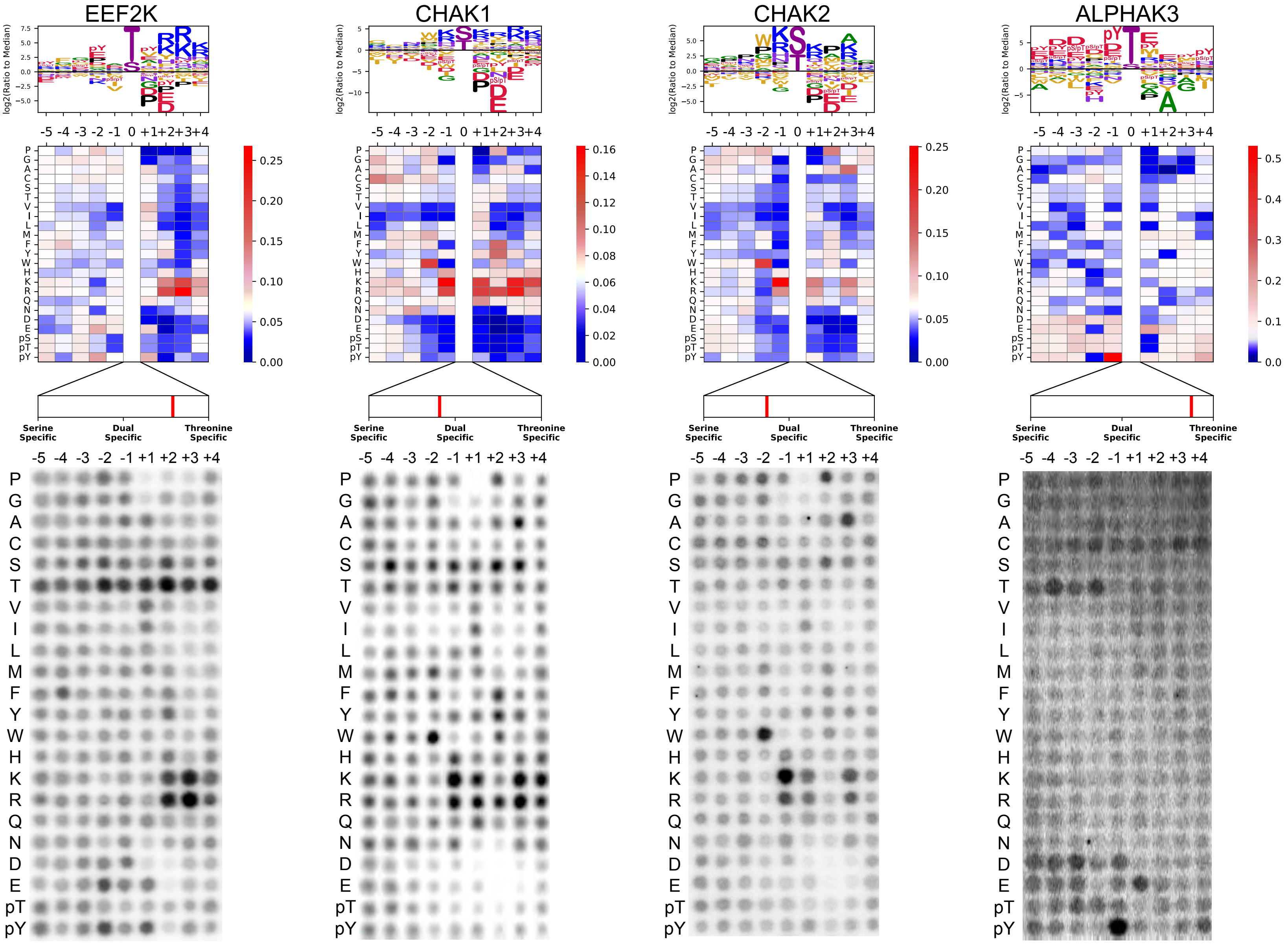
EEF2K
CHAK1
CHAK2
ALPHAK3
log2(Ratio to Median)
-5 -4 -3 -2 -1 0 +1 +2 +3 +4
P G A C S T V I L M F Y W H K R Q N D E pS pT pY
Serine Specific
Dual Specific
Threonine Specific
-5 -4 -3 -2 -1 +1 +2 +3 +4
P G A C S T V I L M F Y W H K R Q N D E pT pY

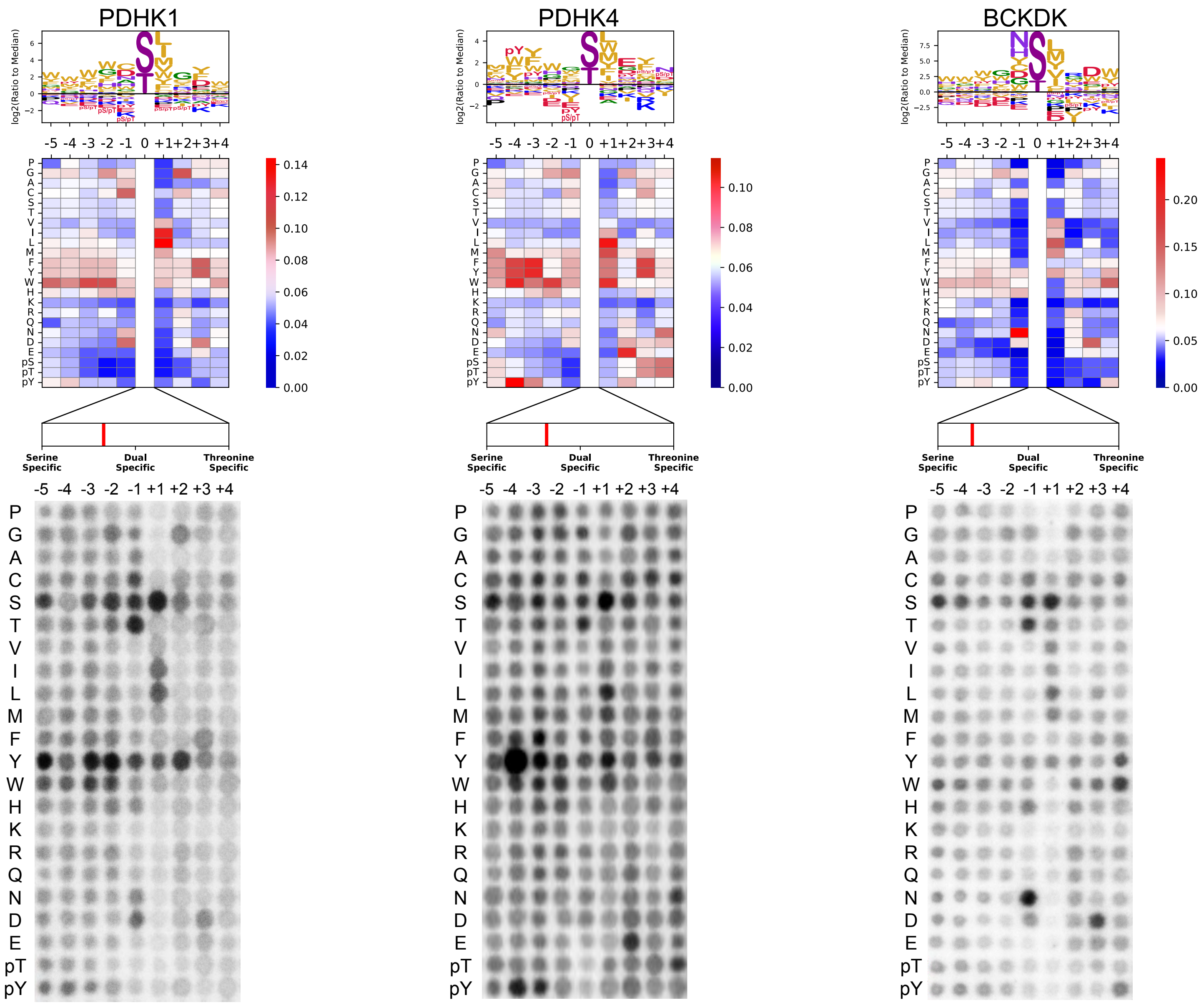
PDHK1
PDHK4
BCKDK
log2(Ratio to Median)
-5 -4 -3 -2 -1 0 +1 +2 +3 +4
P G A C S T V I L M F Y W H K R Q N D E pS pT pY
Serine Specific
Dual Specific
Threonine Specific
-5 -4 -3 -2 -1 +1 +2 +3 +4
P G A C S T V I L M F Y W H K R Q N D E pT pY

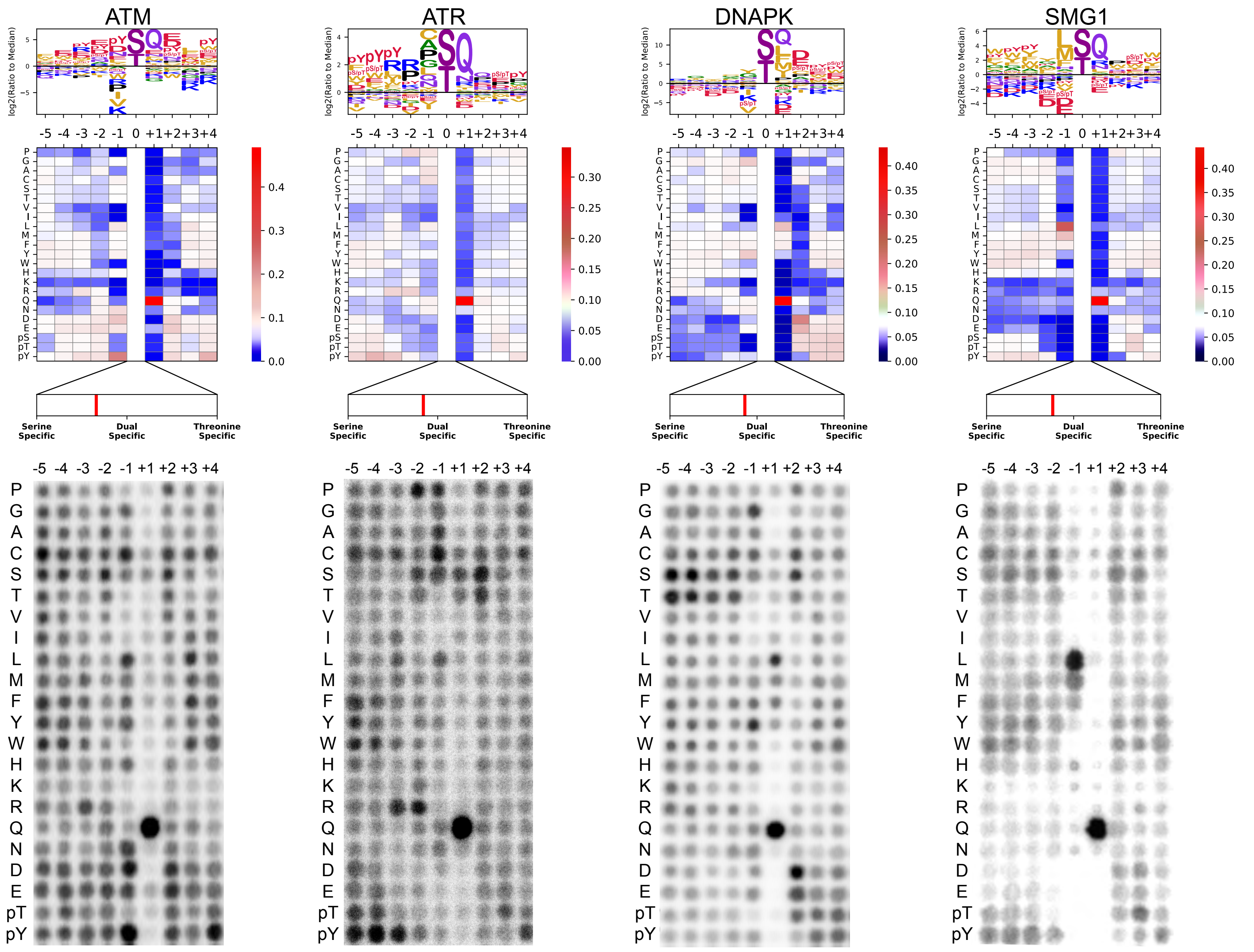
ATM
ATR
DNAPK
SMG1
log2(Ratio to Median)
-5 -4 -3 -2 -1 0 +1+2+3+4
P G A C S T V I L M F Y W H K R Q N D E pS pT pY
Serine Specific
Dual Specific
Threonine Specific
-5 -4 -3 -2 -1 +1 +2 +3 +4
P G A C S T V I L M F Y W H K R Q N D E pT pY

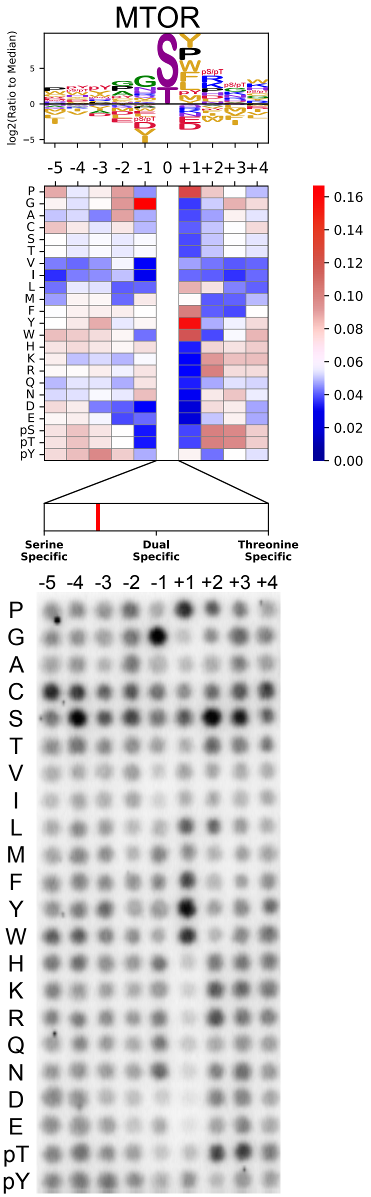

MTOR
log2(Ratio to Median)
-5 -4 -3 -2 -1 0 +1 +2 +3 +4
P G A C S T V I L M F Y W H K R Q N D E pS pT pY
0.16 0.14 0.12 0.10 0.08 0.06 0.04 0.02 0.00
Serine Specific
Dual Specific
Threonine Specific
-5 -4 -3 -2 -1 +1 +2 +3 +4
P G A C S T V I L M F Y W H K R Q N D E pT pY

Supplementary Figure 2: Fully annotated volcano plots presented in Figs. 4b-g.

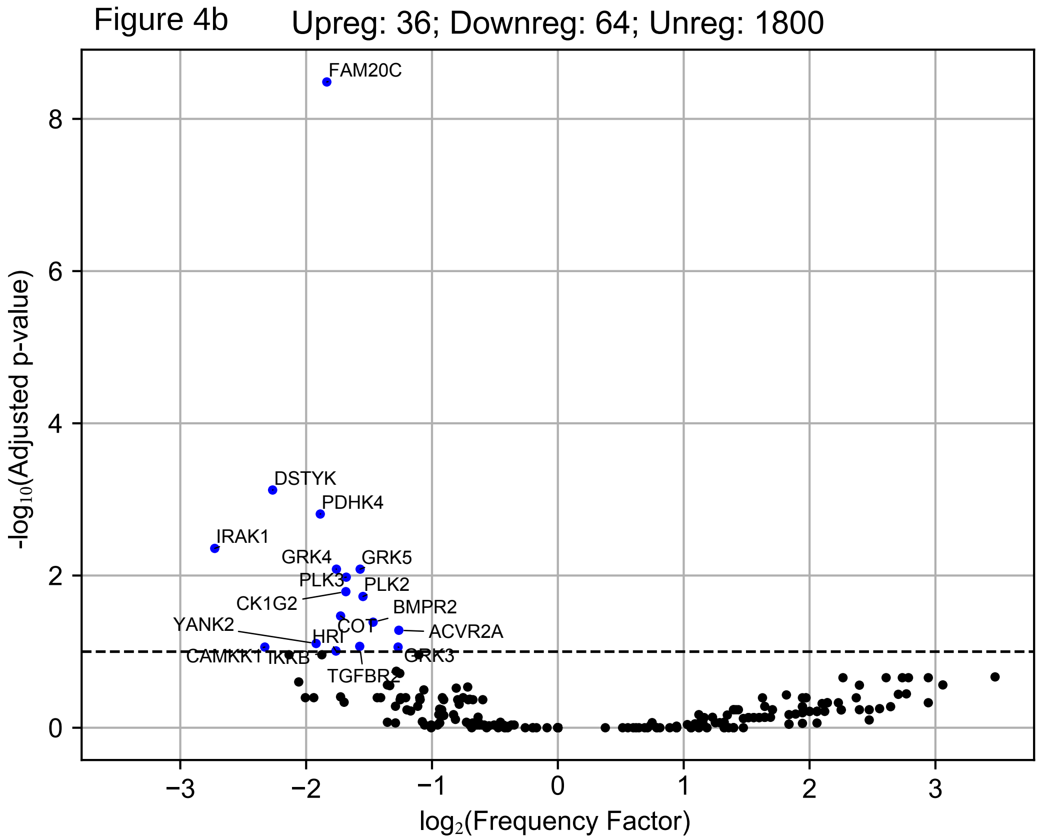

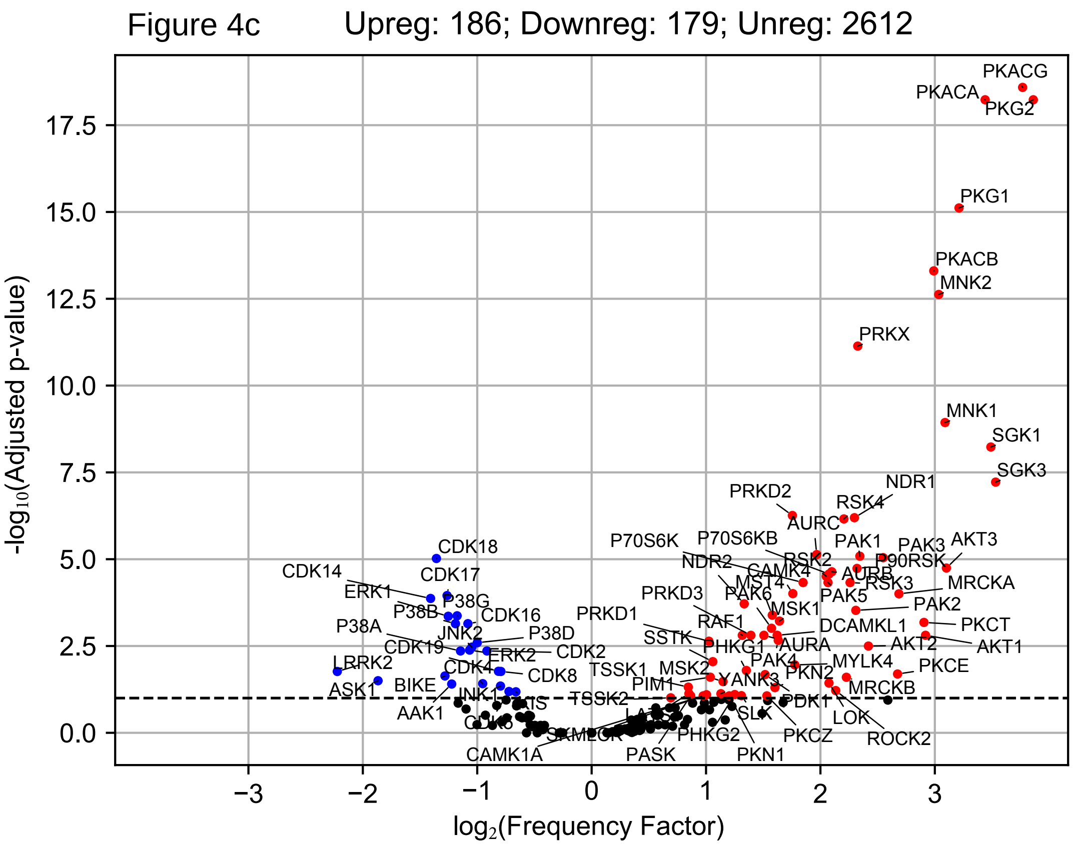
Figure 4c
Upreg: 186; Downreg: 179; Unreg: 2612
-log10(Adjusted p-value)
log2(Frequency Factor)
17.5
15.0
12.5
10.0
7.5
5.0
2.5
0.0
−3
−2
−1
0
1
2
3
PKACG
PKACA
PKG2
PKG1
PKACB
MNK2
PRKX
MNK1
SGK1
SGK3
NDR1
PRKD2
RSK4
AURC
P70S6K
P70S6KB
PAK1
PAK3
AKT3
NDR2
RSK2
P90RSK
CAMK4
AURB
MST4
RSK3
MRCKA
PRKD3
PAK6
PAK5
MSK1
PAK2
PRKD1
RAF1
DCAMKL1
PKCT
SSTK
PHKG1
AURA
AKT2
AKT1
PAK4
MYLK4
TSSK1
MSK2
PKN2
PKCE
PIM1
YANK3
MRCKB
TSSK2
PDK1
SLK
LOK
PKCZ
ROCK2
PHKG2
PASK
PKN1
SKMLCK
CAMK1A
CDK18
CDK14
CDK17
ERK1
P38G
P38B
CDK16
P38A
JNK2
P38D
CDK19
ERK2
CDK2
LRRK2
CDK4
CDK8
ASK1
BIKE
JNK1
AAK1
CDK6

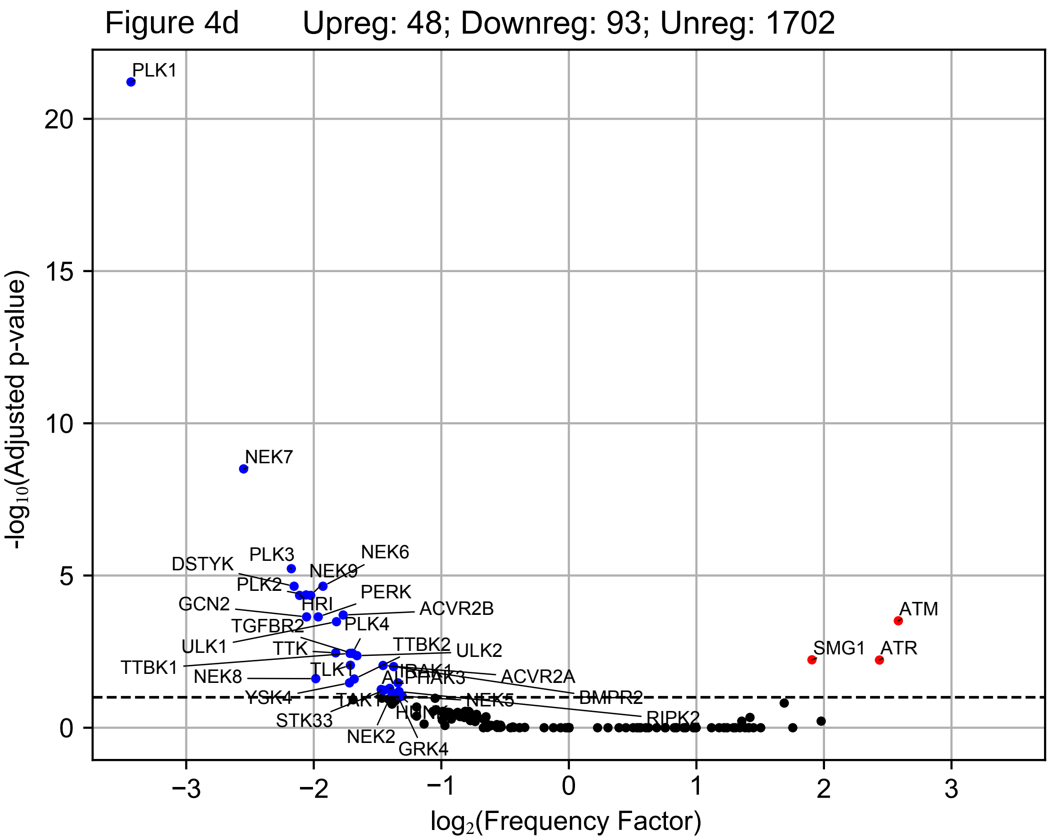
Figure 4d
Upreg: 48; Downreg: 93; Unreg: 1702
-log10(Adjusted p-value)
log2(Frequency Factor)
20
15
10
5
0
−3
−2
−1
0
1
2
3
PLK1
NEK7
PLK3
NEK6
DSTYK
NEK9
PLK2
PERK
GCN2
HRI
ACVR2B
TGFBR2
PLK4
ULK1
TTK
TTBK2
ULK2
TTBK1
TLK1
IRAK1
ACVR2A
NEK8
IRAK3
YSK4
BMPR2
NEK5
STK33
RIPK2
NEK2
GRK4
ATM
SMG1
ATR

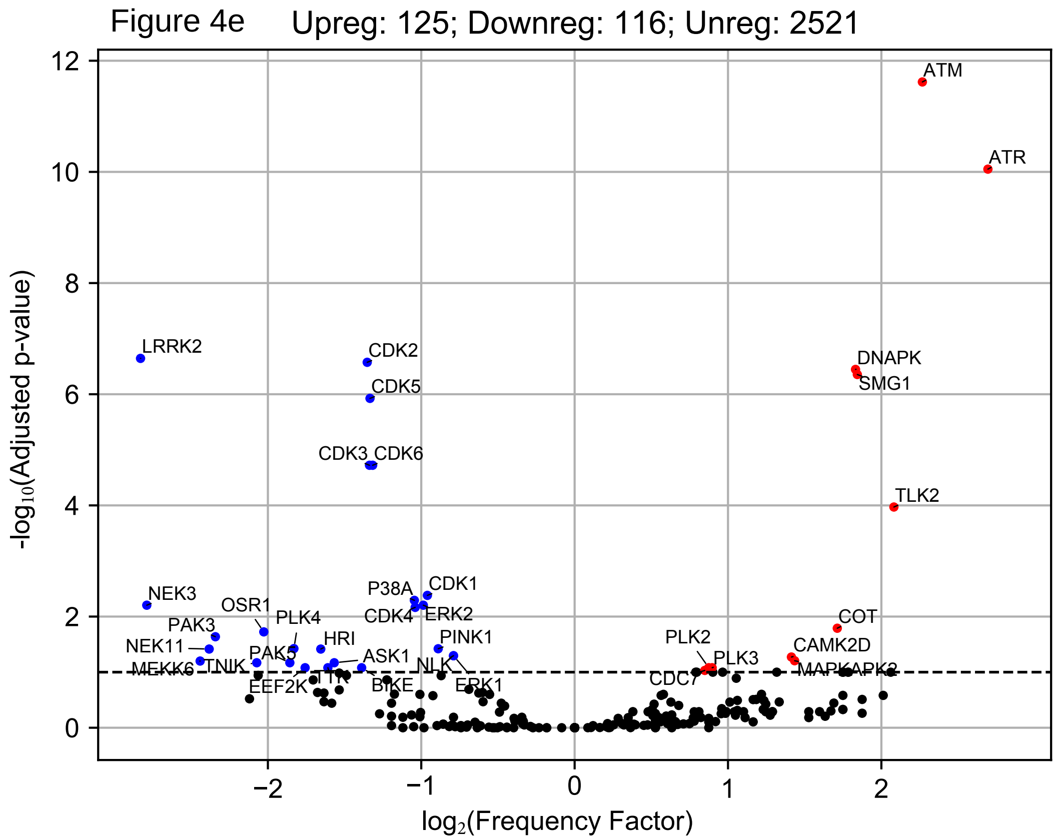
Figure 4e
Upreg: 125; Downreg: 116; Unreg: 2521
-log10(Adjusted p-value)
log2(Frequency Factor)
12
10
8
6
4
2
0
−2
−1
0
1
2
ATM
ATR
LRRK2
CDK2
CDK5
CDK3
CDK6
DNAPK
SMG1
TLK2
NEK3
OSR1
PAK3
PLK4
NEK11
HRI
PAK5
MEKK6
TNIK
EEF2K
TTK
ASK1
BIKE
P38A
CDK1
CDK4
ERK2
PINK1
NLK
ERK1
PLK2
PLK3
CDC7
COT
CAMK2D
MAPKAPK2

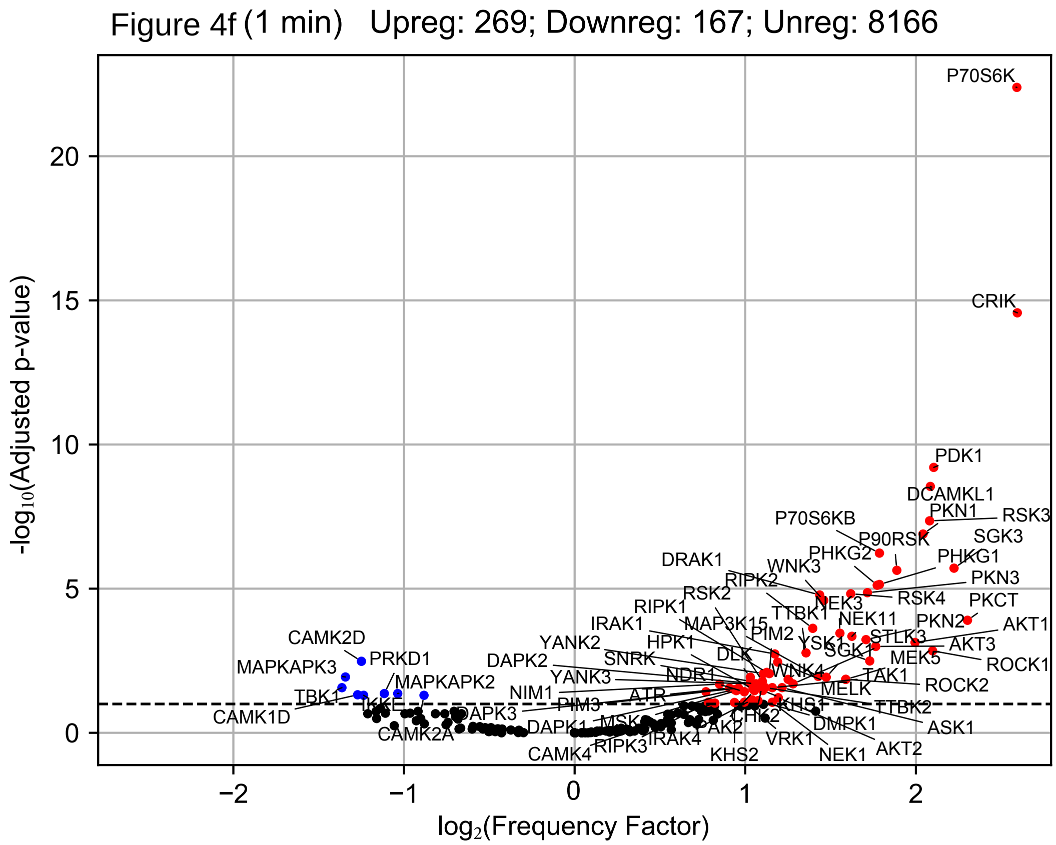

Figure 4f (1 min) Upreg: 269; Downreg: 167; Unreg: 8166
-log10(Adjusted p-value)
log2(Frequency Factor)
P70S6K
CRIK
PDK1
DCAMKL1
PKN1
RSK3
P70S6KB
P90RSK
SGK3
PHKG2
PHKG1
DRAK1
WNK3
PKN3
RIPK2
RSK4
PKCT
NEK3
RSK2
TTBK1
NEK11
PKN2
AKT1
RIPK1
MAP3K15
PIM2
YSK1
STLK3
AKT3
IRAK1
SGK1
MEK5
ROCK1
HPK1
YANK2
DLK
SNRK
WNK4
TAK1
DAPK2
NDR1
ROCK2
YANK3
MELK
NIM1
ATR
TTBK2
PIM3
KHS1
DAPK3
CHK2
ASK1
DMPK1
DAPK1
MSK
PAK2
VRK1
IRAK4
AKT2
RIPK3
KHS2
NEK1
CAMK4
CAMK2D
PRKD1
MAPKAPK3
MAPKAPK2
TBK1
IKKE
CAMK1D
CAMK2A

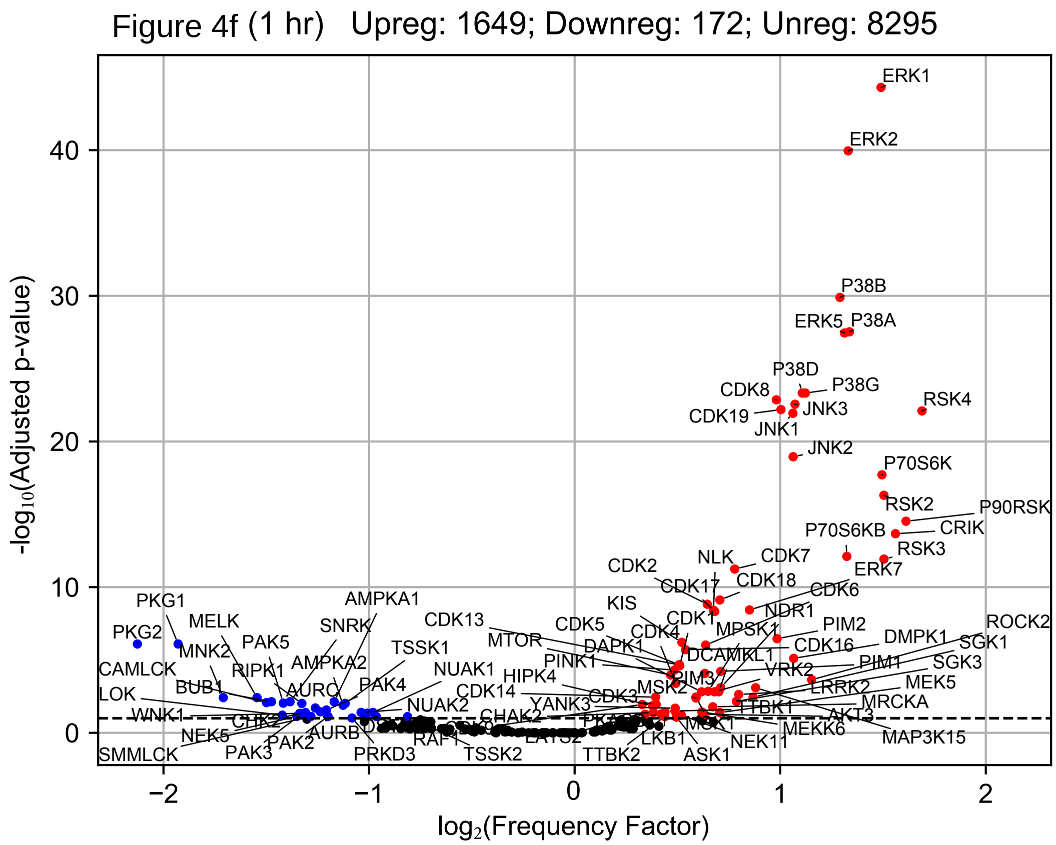
Figure 4f (1 hr) Upreg: 1649; Downreg: 172; Unreg: 8295
-log10(Adjusted p-value)
log2(Frequency Factor)
ERK1
ERK2
P38B
ERK5
P38A
P38D
P38G
CDK8
JNK3
CDK19
JNK1
RSK4
JNK2
P70S6K
RSK2
P90RSK
CRIK
P70S6KB
RSK3
ERK7
NLK
CDK7
CDK2
CDK18
CDK17
CDK6
KIS
NDR1
CDK1
PIM2
MPSK1
CDK4
ROCK2
CDK5
DMPK1
SGK1
CDK16
DAPK1
DCAMKL1
PIM1
SGK3
PINK1
VRK2
PIM3
MEK5
LRRK2
MSK2
CDK3
MRCKA
TTBK1
AKT3
MSK1
MEKK6
NEK11
MAP3K15
LKB1
ASK1
TTBK2
TSSK2
RAF1
PRKD3
CDK13
MTOR
HIPK4
CDK14
YANK3
CHAK2
PKG1
PKG2
MELK
MNK2
PAK5
SNRK
AMPKA1
CAMLCK
RIPK1
AMPKA2
TSSK1
NUAK1
BUB1
AURC
PAK4
LOK
NUAK2
WNK1
CHK2
NEK5
AURB
SMMLCK
PAK3
PAK2
−2
−1
0
1
2
0
10
20
30
40

Figure 4g (30 min)   Upreg: 2147; Downreg: 600; Unreg: 9408

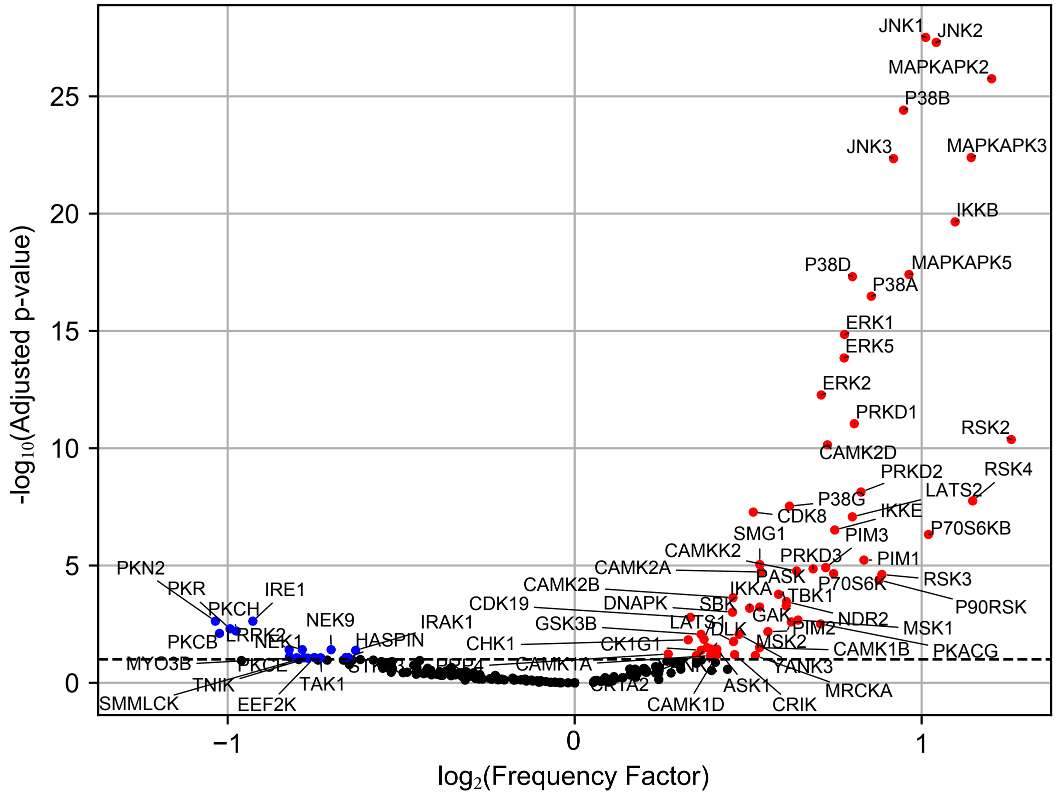

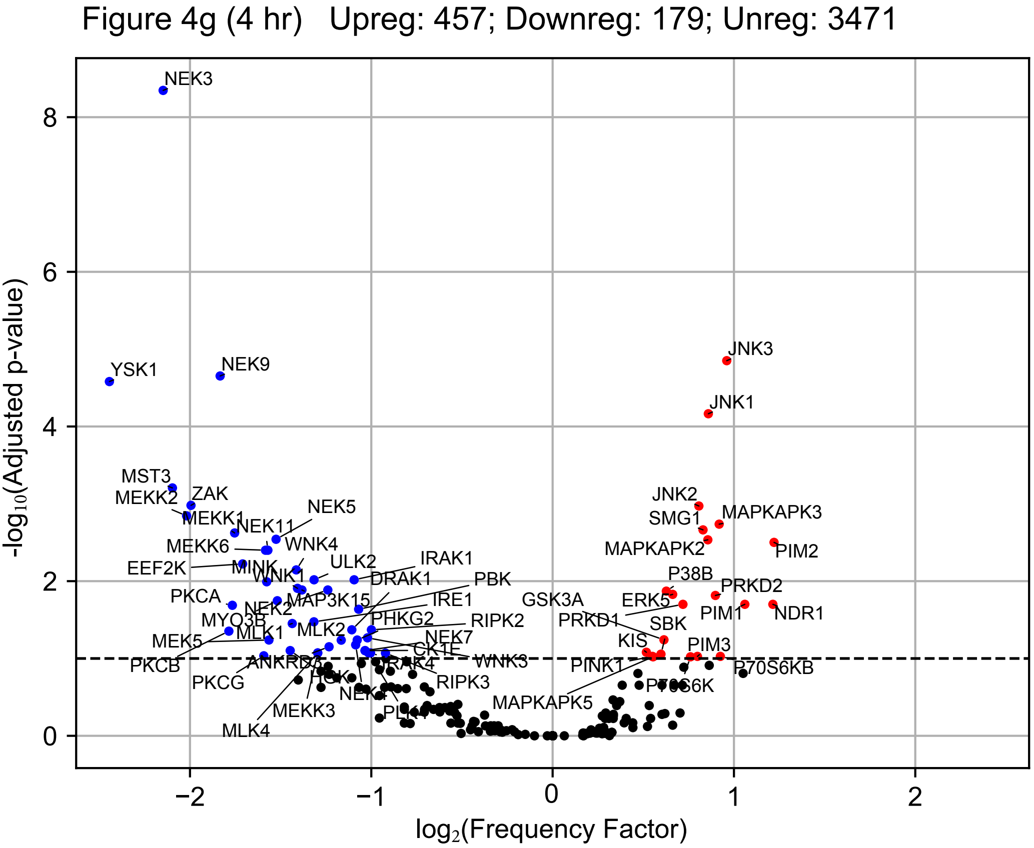
Figure 4g (4 hr) Upreg: 457; Downreg: 179; Unreg: 3471
-log10(Adjusted p-value)
log2(Frequency Factor)
NEK3
YSK1
NEK9
JNK3
JNK1
MST3
MEKK2
ZAK
MEKK1
NEK11
NEK5
MEKK6
WNK4
EEF2K
MINK
ULK2
IRAK1
WNK1
DRAK1
PBK
PKCA
MAP3K15
IRE1
NEK2
MYO3B
PHKG2
RIPK2
MLK1
MLK2
NEK7
MEK5
CK1E
WNK3
PKCB
ANKRD3
IRAK4
PKCG
RIPK3
MEKK3
PLK4
MLK4
JNK2
SMG1
MAPKAPK3
MAPKAPK2
PIM2
P38B
PRKD2
GSK3A
ERK5
PIM1
NDR1
PRKD1
SBK
KIS
PIM3
PINK1
P70S6KB
P70S6K
MAPKAPK5

Supplementary Note 1

$$S_S = \sum_{p=-5}^{+4} M_{S,p}\,; S_T = \sum_{p=-5}^{+4} M_{T,p}$$

$$S_{ctrl} = 0.75 * S_S - 0.25 * S_T; \;\; T_{ctrl} = 0.75 * S_T - 0.25 * S_S$$

$$S_0 = \frac{S_{ctrl}}{\max\,(S_{ctrl}, T_{ctrl})}\,; T_0 = \frac{T_{ctrl}}{\max\,(S_{ctrl}, T_{ctrl})}$$

Supplementary Note 2

$$Raw\ Score_{Kin\ X} = \frac{\prod_{Pos} P_{Kin\ X}(AA, Position)}{\left(\frac{1}{\#Random\ AA}\right)^{length(positions)}}$$
